# Risk Behaviours among Female Sex Workers in China: A Systematic Review and Data Synthesis

**DOI:** 10.1371/journal.pone.0120595

**Published:** 2015-03-27

**Authors:** Eric P. F. Chow, Kathryn E. Muessig, Lei Yuan, Yanjie Wang, Xiaohu Zhang, Rui Zhao, Peng Sun, Xiaoshu Sun, Joseph D. Tucker, Jun Jing, Lei Zhang

**Affiliations:** 1 Research Center for Public Health, School of Medicine, Tsinghua University, Beijing, China; 2 Melbourne Sexual Health Centre, Alfred Health, Melbourne, Victoria, Australia; 3 Central Clinical School, Faculty of Medicine, Nursing and Health Sciences, Monash University, Melbourne, Victoria, Australia; 4 The Kirby Institute, UNSW Australia, Sydney, New South Wales, Australia; 5 The University of North Carolina Project-China, Guangzhou, China; 6 Department of Health Behavior, Gillings School of Global Public Health at the University of North Carolina at Chapel Hill, Chapel Hill, North Carolina, United States of America; 7 Institute for Global Health and Infectious Diseases, UNC Chapel Hill, Chapel Hill, North Carolina, United States of America,; 8 The School of Sociology and Population Studies, Remin University of China, Beijing, China; Brown University, UNITED STATES

## Abstract

**Background:**

Commercial sex is one of the major modes of HIV transmission in China. Understanding HIV risk behaviours in female sex workers (FSW) is of great importance for prevention. This study aims to assess the magnitude and temporal changes of risk behaviours in Chinese FSW.

**Method:**

Five electronic databases were searched to identify peer-reviewed English and Chinese language articles published between January 2000 and December 2012 that reported risk behaviours among FSW in China, including condom use, HIV testing, and drug use. Linear regression and Spearman's rank correlation were used to examine temporal trends in these risk factors. The study followed PRISMA guidelines for meta-analyses and was registered in the PROSPERO database for systematic reviews.

**Results:**

A total of 583 articles (44 English, 539 Chinese) investigating 594,583 Chinese FSW were included in this review. At last sex, condom use was highest with commercial partners (clients), increasing from 53.7% in 2000 to 84.9% in 2011. During this same time period, condom use increased with regular partners from 15.2% to 40.4% and with unspecified partners from 38.6% to 82.5%. Increasing trends were also found in the proportion of sampled FSW who reported testing for HIV in the past 12 months (from 3.2% in 2000 to 48.0% in 2011), while drug use behaviours decreased significantly from 10.9% to 2.6%.

**Conclusion:**

During the first decade of 2000, Chinese FSWs’ self-reported risk behaviours have decreased significantly while HIV testing has increased. Further outreach and intervention efforts are needed to encourage condom use with regular partners, continue promotion of HIV testing, and provide resources for the most vulnerable FSW, particularly low tier FSW, who may have limited access to sexual health and prevention programs.

## Introduction

Compared to the general female population, female sex workers (FSW) are 13.5 times more likely to be infected with HIV [1]. Based on the effectiveness of consistent condom use for preventing the transmission of HIV and other sexually transmitted infections (STI) [2–7], numerous countries have adopted intervention programs for FSW that focus on increasing condom use as a central strategy [8]. In spite of these efforts, as of 2012, the average HIV prevalence among FSW in 50 low and middle-income countries was 11.8% and 15 of these countries had FSW HIV prevalence over 23% [1].

Wide ranging reports have estimated the number of FSW in China between 2 and 20 million women [9–11]. In 2000, China implemented the 100% Condom Use Programme (CUP) in response to a noted increase in HIV prevalence among FSW [12, 13]. This campaign had measured success in some areas of the country, achieving increased condom use between FSW and their male clients [14, 15]. A 2013 analysis that integrated China’s national surveillance data with a systematic review of prevalence studies found that HIV prevalence among FSW had significantly declined, from 0.46% in 2000 to 0.36% in 2010 [16]. However, the prevalence of other STI remains high (e.g. syphilis [0.8–12.5%]; chlamydia [3.9–58.6%]; gonorrhoea [2.0–85.4%], herpes simplex virus 2 [29.7–70.8%]) [17], suggesting a continued need for public health intervention. Furthermore, intervention efforts among Chinese FSW have not adequately addressed the overlap of drug-use and high-risk sexual behaviours [17–20].

Commercial sex work is illegal in China [21] and there has been no overt law or regulation to comprehensively improve HIV/STI services among non-incarcerated Chinese FSW [22]. Free HIV voluntary counselling and testing (VCT) services have been widely available to the general public (including FSW) since the 2003 launch of the national “Four Frees, One Care” policy. This policy provides a range of free prevention and care services including VCT, antiretroviral drugs, prevention of mother-to-child transmission, education for AIDS orphans, and care for people living with HIV/AIDS (PLHIV) [23]. Increasing FSWs’ uptake of free HIV testing could enhance early diagnosis and treatment of HIV/STIs to improve care outcomes and prevent onward transmission of infections to FSWs’ sexual partners [24].

While individual studies have reported a reduction of risk behaviours among FSW in some regions of China [25, 26], a systematic review of all available data could better inform national level policies. Second generation behavioural surveillance for HIV/STIs is an essential part of public health surveillance and informs the best practice of interventions. Given the extensive rollout of condom and VCT programmes over the past decade, it is of great importance to systematically review available information to quantify the magnitude and trends of HIV/STI-related risk behaviours among FSW in China. We conducted a comprehensive literature review of English and Chinese language peer-reviewed articles published between January 2000 and December 2012 to examine temporal trends in FSW condom use in various types of partnerships (regular, non-commercial casual, and clients), HIV testing rate, and drug-use. The findings provide important insights to inform HIV health policies and future outreach efforts for Chinese FSW.

## Methods

This review was reported according to the Preferred Reporting Items for Systematic Reviews and Meta-Analyses (PRISMA) Statement (S1 Checklist) [27]. The protocol for this review has been prospectively registered (CRD42014010774) with the International Prospective Register of Systematic Reviews (PROSPERO) [28].

### Search strategy

Two investigators (EPFC, XZ) performed a literature search within five electronic databases: PubMed, EMBASE, Chinese Scientific Journals Fulltext Database (CQVIP), China National Knowledge Infrastructure (CNKI) and Wanfang Data. Search parameters included Chinese and English language manuscripts published between January 1, 2000 and December 31, 2012. The search strategy employed a combination of Medical Subject Headings (MeSH) terms and keywords: (“China [MeSH]” OR “Chinese [MeSH]”) AND (“sex workers [MeSH]” OR “prostitute” OR “women who sell sex” OR “sex industry” OR “commercial sex”) AND (“risk behaviour” OR “risk behavior” OR “condom” OR “HIV test” OR “drug use” OR “unprotected sex”).

### Selection criteria

#### Type of studies

This review included all types of quantitative, observational (e.g. cohort, cross-sectional, and serial cross-sectional), and intervention studies (e.g. before-and-after). Interventions might have influenced the behaviours of FSW; hence, only baseline data prior to the interventions were included, and all follow-up data were excluded. The following types of publications were excluded: non peer-reviewed literature, review articles, mathematical modelling studies, case-reported studies, studies not conducted in mainland China (e.g. Hong Kong, Macau, Taiwan), and studies with a sample size less than 30.

#### Type of participants

We included studies with women who reported exchanging sex for goods or money (commercial sex) at the time of the interview. We excluded studies that targeted HIV-positive FSW. No additional sample restrictions were applied. There were no restrictions on the demographic characteristics (e.g. age, marital status, education level, ethnicity, and years of selling sex) of the participants.

#### Type of outcome measures

This review assessed risk and protective behaviours among FSW including the following outcomes: condom use with different types of partners (regular, non-commercial casual, clients, and unspecified) at last episode of sex and consistent condom use (i.e. 100% condom use) in the past one month, the uptake of HIV testing in the past 12 months, and the use of illicit drugs. Types of partnerships were based on the definition reported in the studies. Studies without a clear description on the types of partnerships were categorized as ‘unspecified’. Regular partners refer to boyfriends, husbands or stable partners. Non-commercial casual partners mean extra-marital sexual partners, partners with a single sexual encounter. Casual partnership does not involve any money or gifts for sex in exchange. Commercial partners are referred to those who have paid money or gift for sex service from FSW. Studies that reported at least one of these indicators were eligible for this review.

### Quality assessment

Each study was assessed for risk of bias [29] by two independent investigators (EPFC, XZ). Any disagreements were resolved by a third reviewer (LZ). Six domains were used to access the risk of bias: participant selection, measurement of exposure and outcome variables, study design, method of control confounding, methods of statistical analysis, and other sources of bias (S2 Checklist). Each item was scored one point for “low risk of bias” or zero points for “high or unclear risk of bias” for a total quality score ranging from zero to six.

### Data extraction and statistical analysis

Each included study was given a unique identification number and data were abstracted into a predesigned electronic data collection form in a Microsoft Access database (version 2010, Microsoft Corp., Redmond, WA, USA). The data collection form included information on: (1) study design (study location, type of study, sample size, study year); (2) FSW demographic characteristics (age, marital status, level of education); and (3) indicators of risk behaviour as described above.

Meta-regression using the random effect model of Mantel-Haenszel was performed to observe changes in reported risk behaviours over time. The best fit of the regression and its 95% confidence intervals (CIs) of the outcome were reported. Significance level of 0.05 was used. All analyses were performed in STATA software (version 12.0, Stata Corporation, College Station, Texas, USA).

## Results

**Fig 1:**
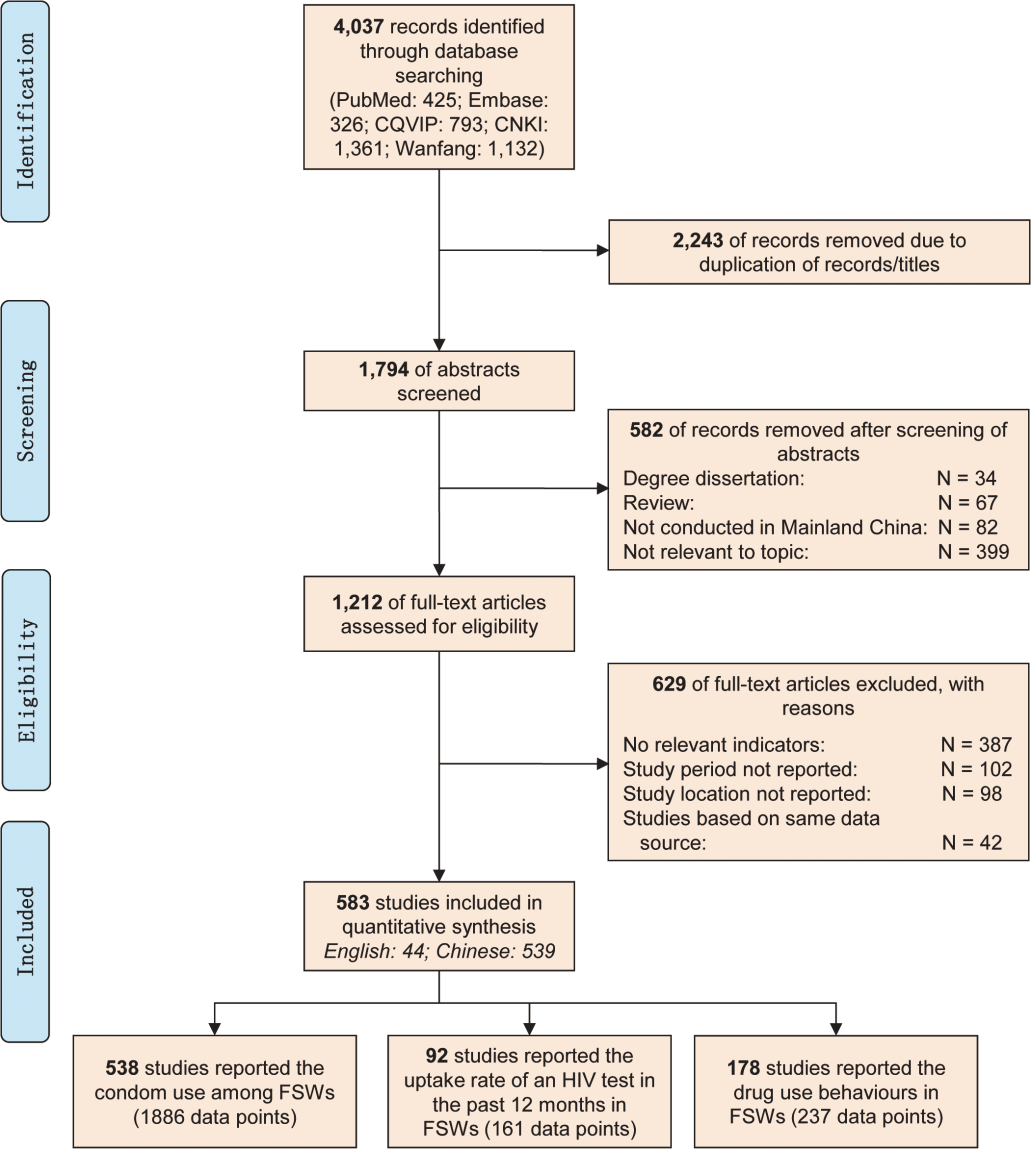
PRISMA flow chart for selection of studies.

Initial keyword searches identified 4,037 records. Removing duplicate records resulted in 1,794 unique articles (Fig. 1) for initial abstract review. Among these 1,794 abstracts reviewed, 582 articles were excluded for the following reasons: not peer-reviewed (N = 34), not primary data (N = 67), not conducted in mainland China (N = 82), and not relevant to the research topic (N = 399). The remaining 1,212 articles were eligible for full-text screening, and 629 were further excluded due to: not reporting any pre-defined outcome measures (N = 387), no study period or location reported (N = 200) and identical data previously reported in an included study (N = 42). Finally, a total of 583 records were included in this analysis. Of those, 538 studies reported condom use among FSW with their male sex partners at the last sexual episode or in the previous month, accounting for 1,886 data points. In addition, 92 studies (161 data points) reported the prevalence of HIV testing in the past 12 months and 178 studies (237 data points) reported drug use behaviours (S1–S6 Tables). A total of 594,583 FSW were included in this review. Sample sizes ranged widely from 30 to 15,323, with an average sample size of 614. Of the 583 studies, the median score of the quality assessment was three. Overall, most studies had low risk of bias (score = 1 for quality assessment item) in terms of methods for selecting study participants (79.3%) and methods for measuring exposure and outcome variables (97.0%). However, only 24.2% of studies had low risk of study design-specific sources of bias. Among the 583 studies, only 36.7% (N = 214) of the studies reported the mean age of the participants, the mean age ranged from 18.1 to 35.0 years, with a median of 24.3 (IQR = 22.7–26.0).

### Condom use at the last sexual episode

**Fig 2:**
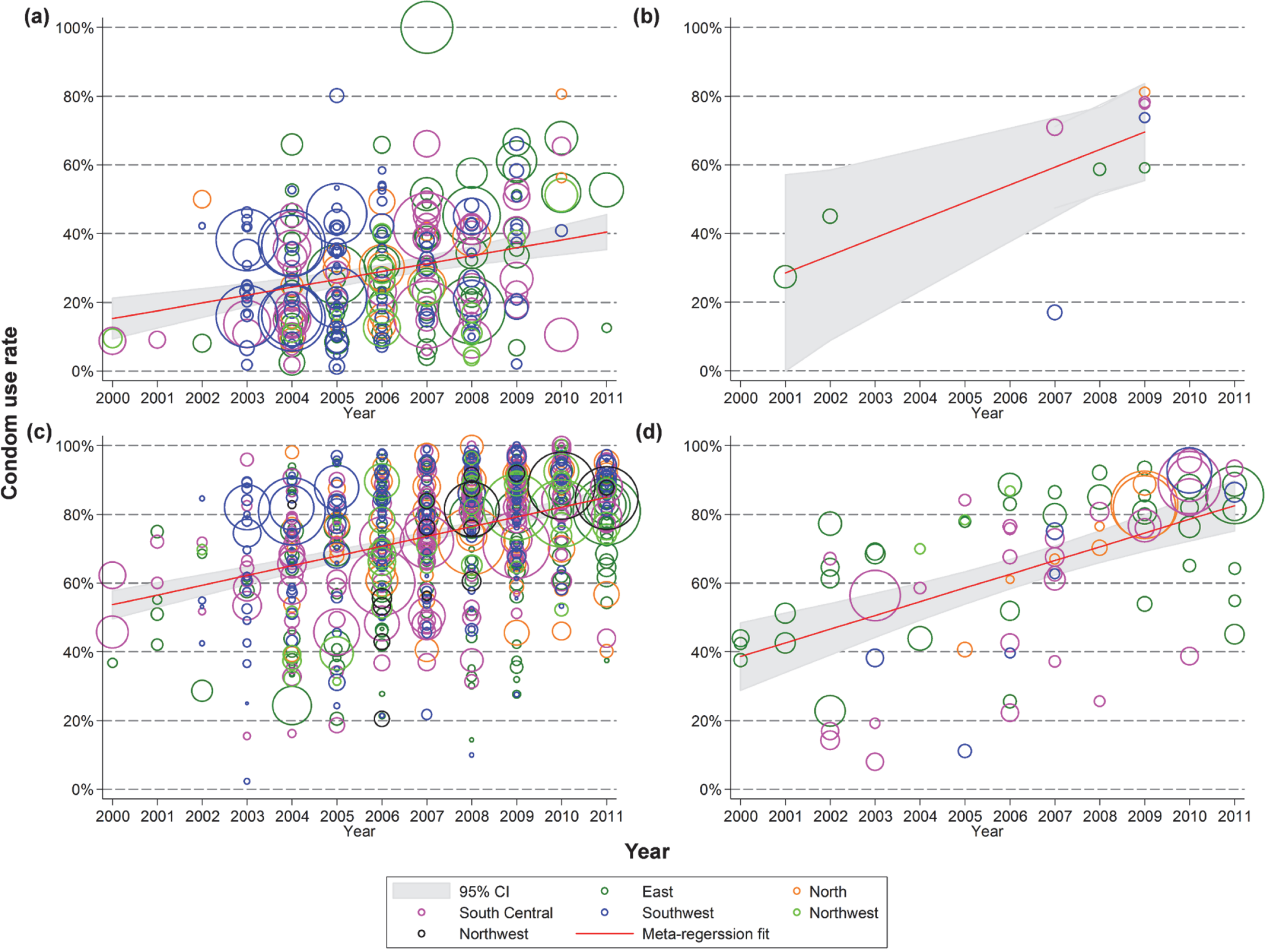
Condom use rate in female sex workers with male (a) regular; (b) non-commercial casual; (c) clients; and (d) unspecified partners during last sexual act, 2000–2011.

Our trend analysis showed that condom use between FSW with unspecified partners at the last sexual episode increased significantly from 38.6% (95% CI: 28.5–48.6%) in 2000 to 82.5% (74.7–89.9%) in 2011 (*p*<0.001), corresponding to an average annual growth rate of 4.0% per year (2.6–5.3%) (Fig. 2). In particular, Chinese FSW had a higher condom use rate with commercial partners (clients) than non-commercial partners at the last sexual episode. Condom use with clients increased significantly from 53.7% (49.4–58.0%) in 2000 to 84.9% (82.5–87.3%) in 2011 (*p*<0.001); while condom use with regular partners also increased from 15.2% (9.2–21.3%) to 40.4% (35.4–45.9%) over the same period (*p*<0.001). The corresponding annual growth rates for commercial and regular partners were 2.8% (2.3–3.4%) and 2.3% (95% CI: 1.3–3.2%), respectively.

### Consistent condom use in the past one month

**Fig 3:**
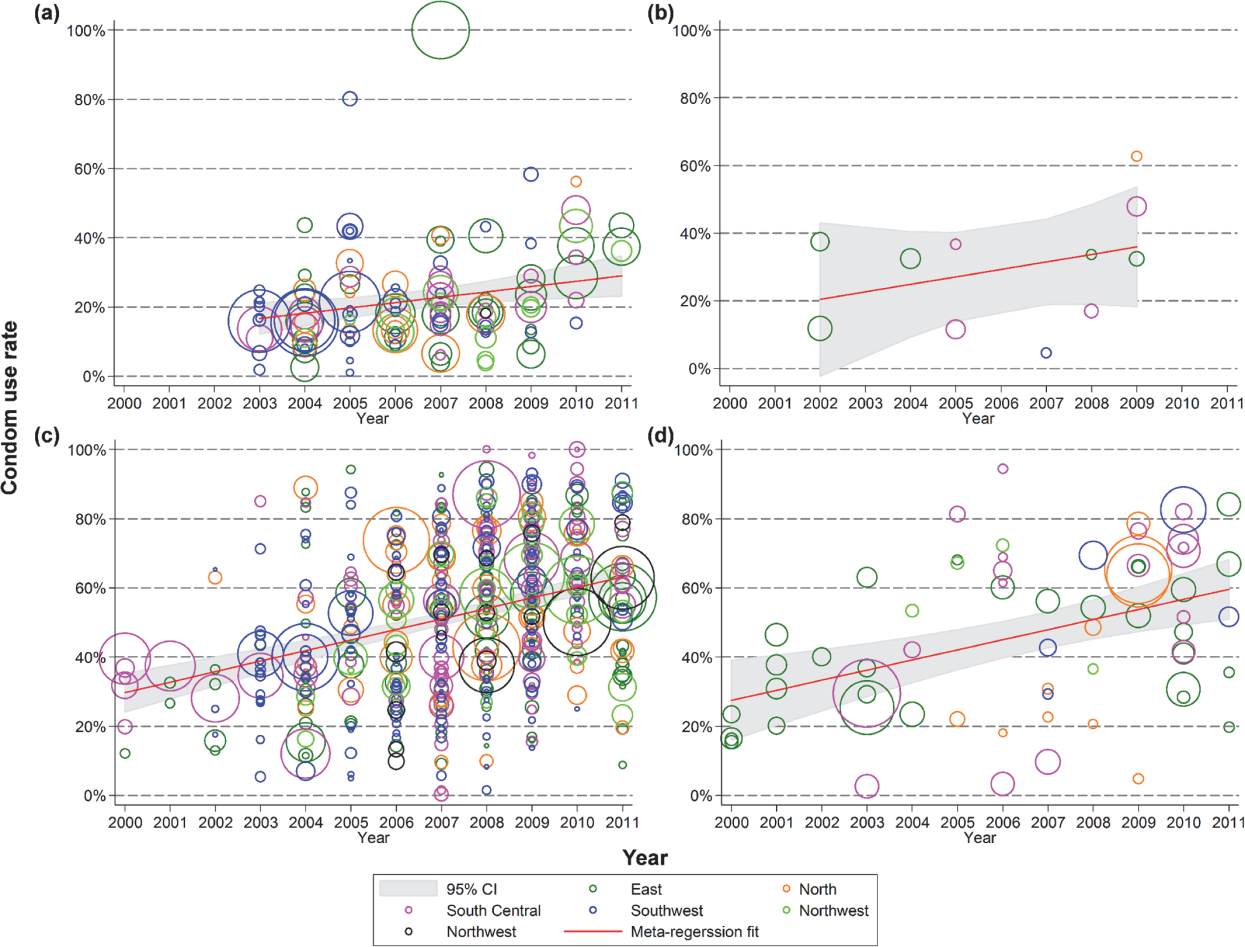
Consistent condom use rate in female sex workers with male (a) regular; (b) non-commercial casual; (c) clients; and (d) unspecified partners in the last one month, 2000–2011.

We observed similar trends in consistent condom use in the previous month among Chinese FSW (Fig. 3). Consistent condom use was most frequent in sexual activities with clients and the rate significantly increased from 29.7% (24.0–35.4%) in 2000 to 63.2% (59.7–66.6%) in 2011 (*p*<0.001), an annual increase of 3.0% (2.3–3.8%). Consistent condom use with regular partners in the past month significantly increased from 16.7% (11.9–21.5%) in 2003 to 29.0% (22.9–34.9%) in 2011 (*p* = 0.009), which had an annual growth rate of 1.5% (0.4–2.7%). Only 12 studies reported consistent condom use with non-commercial casual partners and no significant temporal trends were observed (*p* = 0.296), though the rate increased from 20.4% to 35.9% during 2002–2009.

**Fig 4:**
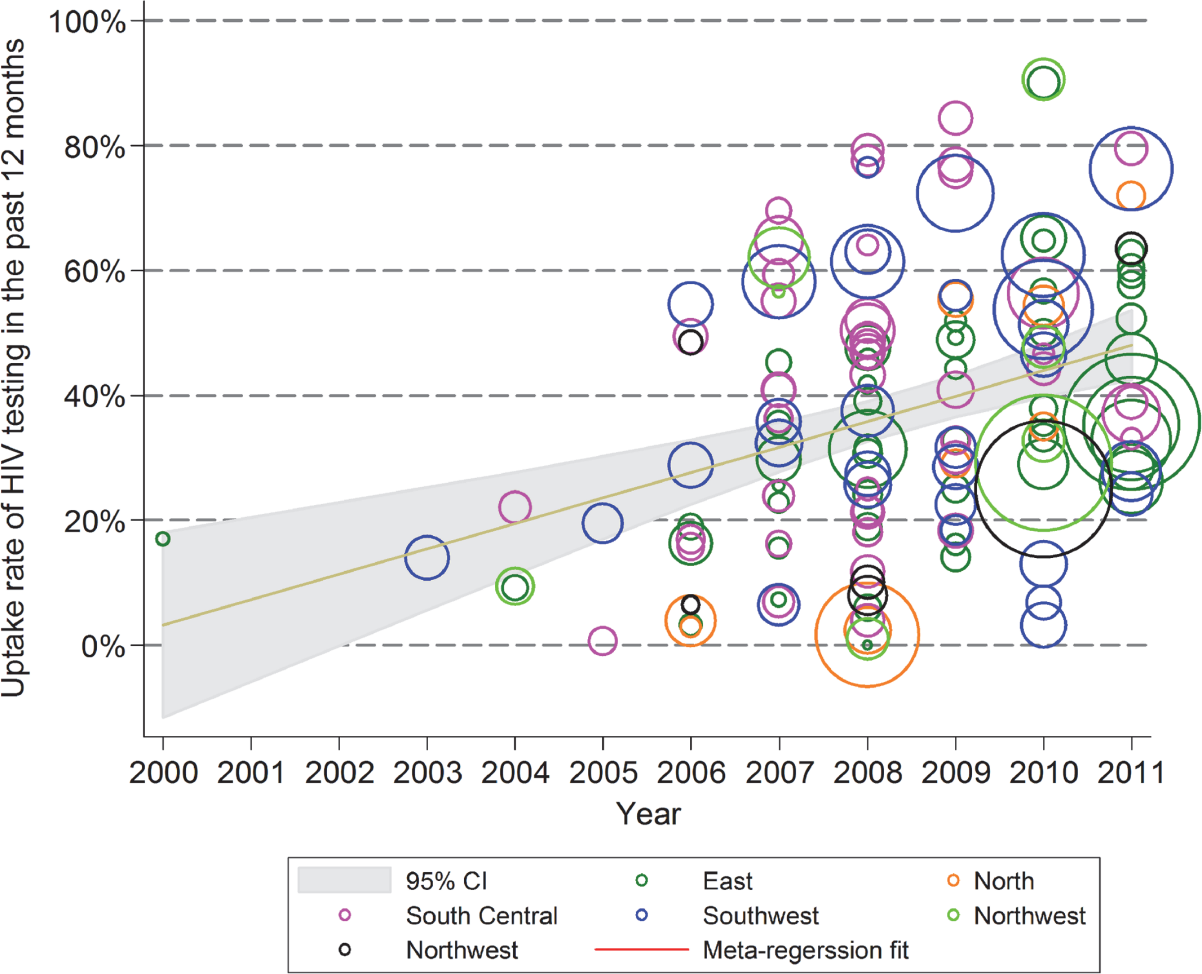
Uptake rate of HIV testing in female sex workers in the past 12 months, 2000–2011.

**Fig 5:**
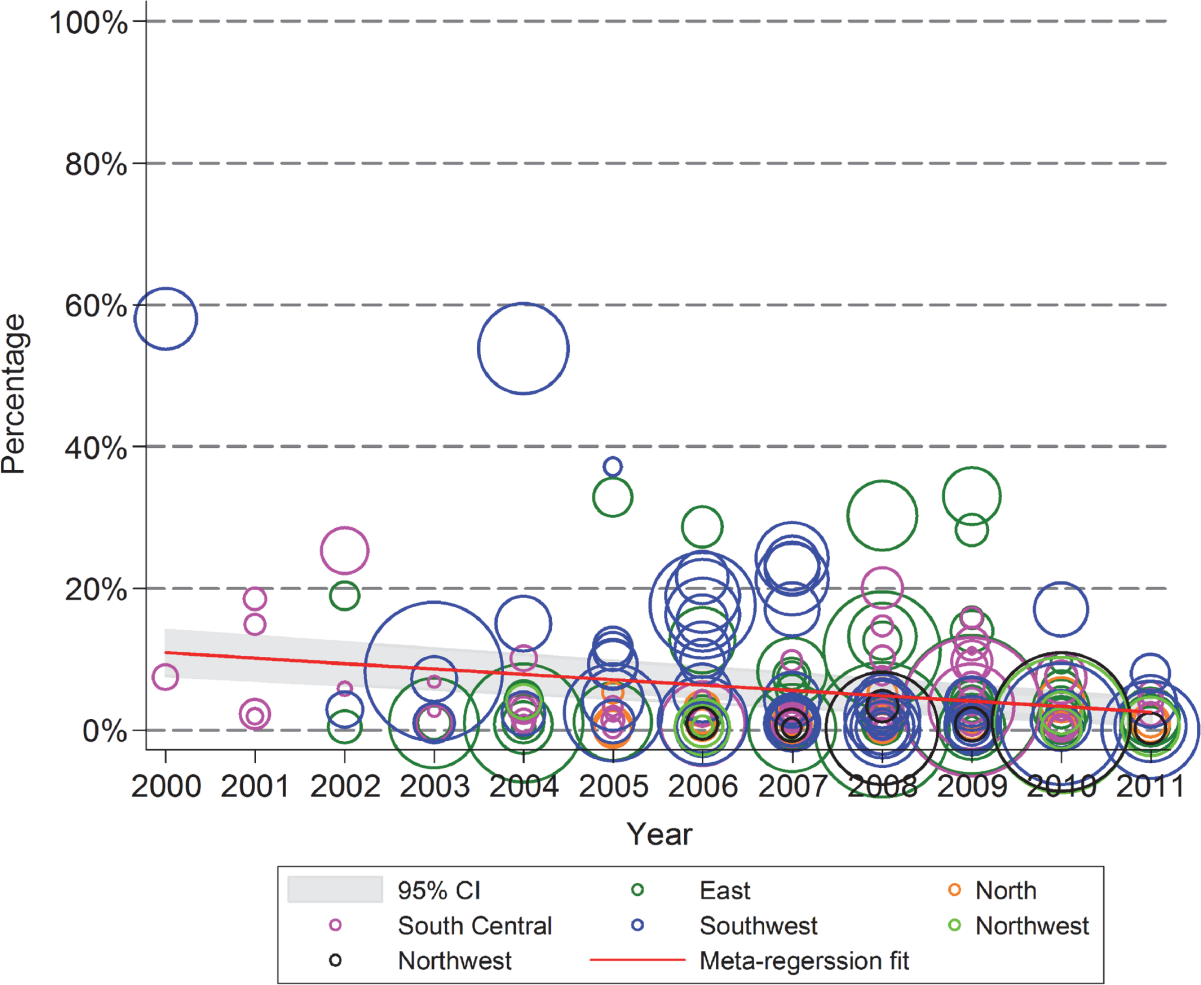
Proportion of female sex workers who have ever used drugs, 2000–2011.

The proportion of sampled FSW who reported testing for HIV in the past 12 months increased from 3.2% (0.0–18.2%) in 2000 to 48.0% (42.1–53.7%) in 2011 (*p*<0.001), with an annual growth of 4.1% (2.3–5.8%) (Fig. 4). The proportion of FSW who reported ever using drugs decreased significantly from 10.9% (7.6–14.4%) in 2000 to 2.6% (0.6–4.9%) in 2011 (*p* = 0.001), for an annual decline of -0.8% (−0.3 to −1.2%) (Fig. 5).

## Discussion

This systematic review of 583 studies of FSW in China fills a critical gap in the field. Past reviews on Chinese FSW have focused on HIV/STI prevalence [17, 30] and only two have included condom use: a 2005 synthesis of 11 studies [31] and a 2008 review of 20 studies [32]. Our analysis contributes significantly by including a large number of English and Chinese language peer-reviewed articles and by presenting temporal trends and differentiation in condom use by partner type. Overall, the analyses identified a number of positive trends in improved sexual health and reduced risk behaviours including increased condom use across all partner types, increased HIV testing, and decreased drug use.

According to 2011 data from China’s national sentinel surveillance sites, 87.5% of FSW reported using a condom at the last sexual episode (with unspecified partner type) [25]. This rate is higher than what our synthesis found with either commercial, unspecified, or regular partners (84.9%, 82.5%, and 40.4%, respectively) in 2011, and may suggest over-reporting or over-representation of higher tier commercial sex venues among Chinese national surveillance sites. FSW’s ability to use condoms with clients is influenced by individual and venue-level factors where women who work in the lower socioeconomic tiers of sex work, including on the streets and in smaller venues, are at greater risk of unprotected sex and HIV/STI [33, 34]. In fact, 2012 data from the sentinel surveillance sites showed that although consistent condom use among low tier (street-based) FSW increased from 2010 to 2012, it still remains lower than condom use among high tier FSW (59% versus 72%) [30, 35]. FSW who do not use condoms with clients are at higher risk of HIV/STI [36]. A nationwide probability sample has shown that about 6.9% men aged 18–49 years have ever patronized FSW during their lifetime, and about 19.4% of men who did not have consistent condom use with FSW reported having STI in the past 12 months [37]. This study shows that men who did not always use condom with FSW were 3.71 (95% CI 1.18–11.66) times more likely to report past STI in comparison with those who use condom consistently.

Consistent with other Chinese [32, 38] and international [39, 40] literature, we found that condom use among FSW was higher with clients compared to regular and non-commercial casual partners. Chinese FSW may avoid condom use with regular partners in order to promote trust and intimacy, and to keep sex work hidden [41]. Comprehensive, multi-level interventions with FSW in China [42] and internationally [43–45] have effectively increased condom use with both clients and regular partners. Addressing low condom use with regular partners among Chinese FSW reveals a missed opportunity within current intervention efforts.

We found a dramatic overall increase in the prevalence of recent HIV testing. Our pooled 2011 estimate of 48.0% FSW testing in the past 12 months is somewhat higher than the 38.2% reported by the 2011 data from the Chinese national sentinel surveillance sites [25]. Free HIV testing, the government’s large scale testing campaigns which include FSW as a key population, and increased awareness have likely all contributed to this public health success [25]. Yet with over half of FSW still not testing regularly, renewed efforts are needed. In particular, street-based FSW have higher prevalence of HIV [35, 46], yet testing has reached only 37% nationally [25]. Government supported, enhanced community-based syphilis screening and treatment programs for FSW have significantly reduced incidence density and could be used as one model for continued expansion of HIV testing uptake [47]. As lifetime HIV testing among other key populations, such as men who have sex with men also remains low [48, 49], widespread health communication campaigns as well as targeted outreach efforts are warranted. Increased use of on-site rapid testing via finger prick and oral swab may provide an acceptable supplement to current testing options which typically involve venous blood draws [50]. Alternative models that promote confidential, non-stigmatizing testing environments via women’s health clinics and FSW-specific health clinics have had some success in China and should also be considered [51, 52].

While the prevalence of injection drug use among FSW in China appears to have decreased, the burden of HIV among these women remains substantial [53–55]. A review of HIV/STI among FSW found 12 to 49% HIV prevalence among samples of women who also inject drugs [17]. Whether these women are primarily sex workers who also inject drugs, or drug users who sell sex, in either case they are underserved within the public health system and face additional barriers to safe sex. However, these barriers are not insurmountable. For example, among injection drug using FSW in Southern China, those women who had experienced violence and lacked social support were less likely to use condoms, while those who had received HIV/STI supportive services or had clients who were willing to use condoms had more consistent condom use [55].

Several limiting factors contextualize this analysis. First, there were few studies published in 2000 and 2001. The number of FSW-focused studies has increased exponentially over the past decade and a half. This weakens the baseline comparison measure but would suggest that the pooled estimates become more reliable in later years. Second, the majority of published studies drew samples from urban areas, thus FSW in rural areas were underrepresented. The few studies that have been conducted among rural and peri-urban FSW suggest that these women may be at even higher risk for HIV/STI and report more injection drug use [53] than their urban counterparts. For example, a study among 102 male clients in rural Guangxi found that only 16.3% reported condom use at last sex with FSW [34]. The coverage of 100% CUP campaigns is likely to have been most focused on urban areas where health departments and community organizations have more resources to expend. As such, the magnitude of increased condom use found in our meta-analysis may overestimate improvements in risk behaviours among FSW in rural and peri-urban areas. Third, the quality of the published evidence varies widely across studies. The majority of Chinese language studies did not describe potential sources of design-specific bias as these are not commonly required or reported in Chinese publications. Fourth, it is suggested that some demographic characteristics such as age and education level may be associated with condom use [56]. However, due to limited data reported in the articles, we were not able to investigate such relationships. Fifth, other factors, such as alcohol use prior to or during sexual intercourse [57], may be associated with condom use of FSW with clients. However, only very few studies reported and examined this in the Chinese setting. Future studies on sexual behaviour of FSW under the influence of alcohol are essential to understand the relationship between alcohol consumption and condom use. Sixth, a substantial proportion of studies did not differentiate condom use according to the type of their sexual partners. Information on condom uses with unspecified partners has limited implications to HIV prevention and control for FSW in China. Seventh, the majority of studies did not report the condom use rate according to the specific types of venues, and the definition of tiers of venues also varies substantially across studies. Hence, we were not able to examine the trends of condom use by venue tiers.

This study yields several key implications for research, implementation, and policy. In general, condom use, HIV testing, and drug use among FSW in China are improving, but more work is needed. In particular, more focus is needed on the most vulnerable FSW—those who live in rural areas, use drugs, or work on the streets and lower tier of the sex industry [16, 30, 58, 59]. These women are least likely to be reached by the majority of existing public health efforts for testing, education, and 100% CUP and have the least resources for seeking care. In addition, while continuing to emphasize the importance of condom use with clients, targeted interventions are needed to promote condom use with regular and other non-commercial partners. In fact, significant changes in condom use are unlikely to be achieved through further increasing distribution and promotion of condoms to FSW without simultaneous efforts to shift condom use norms with regular partners and increase legal protections to reduce violence against FSW [60]. A recent systematic review has demonstrated that overlapping risk behaviours among key populations are common in China, such as FSW who also use drugs [16]. Previous study has also shown that FSW who also use drugs are less likely (OR = 0.25) to have consistent condom use than otherwise [56]. Currently, most behavioural interventions for FSW in China only focus on condom distribution; while the components for HIV testing and drug uses are always being neglected. Given that drug use may be associated with condom use and hence increase the risk of HIV/STI acquisition, it is important to integrate drug control and treatment as part of the interventions for FSW. Furthermore, adoption of a women’s reproductive health framework (rather than infectious disease prevention framework) may offer a more attractive approach for FSW and result in higher engagement [38, 51, 52].

## Supporting Information

S1 Checklist PRISMA Checklist. (PDF)

S2 Checklist Quality Assessment Checklist. (PDF)

S1 Table Studies reported the rate of condom use in female sex workers with male regular partners. (PDF)

S2 Table Studies reported the rate of condom use in female sex workers with male non-commercial casual partners. (PDF)

S3 Table Studies reported the rate of condom use in female sex workers with male clients. (PDF)

S4 Table Studies reported the rate of condom use in female sex workers with any male partners. (PDF)

S5 Table Studies reported the uptake rate of an HIV test in the past 12 months in female sex workers. (PDF)

S6 Table Studies reported the drug use behaviours in female sex workers. (PDF)
