# Supplementary material for: Risk Behaviours among Female Sex Workers in China: A Systematic Review and Data Synthesis
Source: PLoS One. 2015 Mar 27;10(3):e0120595. doi: 10.1371/journal.pone.0120595 (PMC4376708; doi:10.1371/journal.pone.0120595)
Supplement: S2 Checklist — (PDF) [file pone.0120595.s002.pdf]

Checklist S2. Quality Assessment Checklist

	Bias	Decision criteria	Score
1	**Methods for selecting study participants**	Appropriate source population (cases, controls and cohorts) and inclusion or exclusion criteria	1
		Inappropriate source population (cases, controls and cohorts) and inclusion or exclusion criteria	0
		Not reported	0
2	**Methods for measuring exposure and outcome variables**	Appropriate measurement methods for both exposure(s) and/or outcome(s)	1
		Inappropriate measurement methods for both exposure(s) and/or outcome(s), e.g. did not report the duration of measurement (condom use in past x months)	0
		Not reported	0
3	**Design-specific sources of bias (excluding confounding)**	Appropriate methods outlined to deal with any design-specific issues such as recall bias, interviewer bias, biased loss to follow or blinding	1
		Inappropriate methods outlined to deal with any design-specific issues such as selection bias, performance bias, attrition bias, detection bias, or reporting bias<br>• Did not apply inclusion/exclusion criteria uniformly to all comparison groups<br>• Did not rule out any impact from a concurrent intervention or an unintended exposure that might bias results<br>• The study did not maintain fidelity to the intervention protocol<br>• Outcomes were not assessed and implemented consistently across all study participants	0
		Not reported	0
4	**Methods to control confounding**	Appropriate design and/or analytical methods	1
		Inappropriate design and/or analytical methods, and did not consider possible confounding	0
		Not reported	0
5	**Statistical methods (excluding control of confounding)**	Appropriate use of statistics for primary analysis of effect	1
		Inappropriate use of statistics for primary analysis of effect	0
		Not reported	0
6	**Conflict of interest**	Declarations of no conflict of interest or identification of funding sources	1
		Reported conflict of interest	0
		Not reported	0
