# Supplementary material for: Risk Behaviours among Female Sex Workers in China: A Systematic Review and Data Synthesis
Source: PLoS One. 2015 Mar 27;10(3):e0120595. doi: 10.1371/journal.pone.0120595 (PMC4376708; doi:10.1371/journal.pone.0120595)
Supplement: S1 Table — (PDF) [file pone.0120595.s003.pdf]

Table S1. Studies reported the rate of condom use in female sex workers with male regular partners.

First author, published year	Study period	Location	Province	Region	Recruitment venue	Sampling method	Measurement period*	Number of FSW used condom	Total number of FSW	Condom Usage (%)	QA Score
Yang P, 2005 [1]	2002/12	Fuzhou	Fujian	East	Entertainment	Random sampling	LA	12	149	8.1%	3
He JG, 2005 [2]	2004/10-2004/12	Wuhu	Anhui	East	Entertainment	--	LA	21	207	10.1%	5
Zhu CQ, 2006 [3]	2004/08-2004/09	Shaoxing	Zhejiang	East	Entertainment	Random sampling	LA	27	197	13.7%	3
Li XJ, 2005 [4]	2004/10-2004/11	Hefei	Anhui	East	Entertainment	Two-stage cluster sampling	LA	31	130	23.8%	7
Li XJ, 2005 [4]	2004/10-2004/11	Hefei	Anhui	East	Entertainment	Two-stage cluster sampling	LA	49	129	38.0%	7
He JG, 2005 [2]	2004/10-2004/12	Wuhu	Anhui	East	Entertainment	--	LA	14	193	7.3%	5
Li XJ, 2005 [4]	2004/10-2004/11	Hefei	Anhui	East	Entertainment	Two-stage cluster sampling	LA	16	55	29.1%	7
Li XJ, 2005 [4]	2004/10-2004/11	Hefei	Anhui	East	Entertainment	Two-stage cluster sampling	LA	26	55	47.3%	7
Li XF, 2006 [5]	2003/08-2003/10, 2004/05-2004/07	Qingdao	Shandong	East	Entertainment	Outreach	LA	7	281	2.5%	7
Du YP, 2006 [6]	2004/10	Jingjiang	Jiangsu	East	Entertainment	--	LA	130	197	66.0%	4
Jin TL, 2005 [7]	2004/05	Lishui	Zhejiang	East	Entertainment	--	LA	31	87	35.6%	5
Xiao HM, 2007 [8]	2004	Nanchang	Jiangxi	East	Entertainment	Random sampling	LA	41	78	52.6%	4

First author, published year	Study period	Location	Province	Region	Recruitment venue	Sampling method	Measurement period*	Number of FSW used condom	Total number of FSW	Condom Usage (%)	QA Score
Xiao HM, 2007 [8]	2004	Nanchang	Jiangxi	East	Entertainment	Random sampling	LA	41	78	52.6%	4
Xiao HM, 2007 [8]	2004	Nanchang	Jiangxi	East	Entertainment	Random sampling	LA	34	78	43.6%	4
Guan JH, 2009 [9]	2004/08	Fuzhou/putian	Fujian	East	Entertainment	Random sampling	LA	79	501	15.8%	5
He JG, 2005 [2]	2004/10-2004/12	Wuhu	Anhui	East	Entertainment	--	P1M	21	207	10.1%	5
Li XJ, 2005 [4]	2004/10-2004/11	Hefei	Anhui	East	Entertainment	Two-stage cluster sampling	P1M	31	130	23.8%	7
He JG, 2005 [2]	2004/10-2004/12	Wuhu	Anhui	East	Entertainment	--	P1M	14	193	7.3%	5
Li XF, 2006 [5]	2003/08-2003/10, 2004/05-2004/07	Qingdao	Shandong	East	Entertainment	Outreach	P1M	7	281	2.5%	7
Li XJ, 2005 [4]	2004/10-2004/11	Hefei	Anhui	East	Entertainment	Two-stage cluster sampling	P1M	16	55	29.1%	7
Xiao HM, 2007 [8]	2004	Nanchang	Jiangxi	East	Entertainment	Random sampling	P1M	34	78	43.6%	4
Guan JH, 2009 [9]	2004/08	Fuzhou/putian	Fujian	East	Entertainment	Random sampling	P1M	79	501	15.8%	5
Yan HJ, 2007[10]	2005	Zhangjiagang, Liyang, Tongzhou, Nanjing, Yancheng, Huaian	Jiangsu	East	Entertainment	Random sampling	LA	445	1585	28.1%	3
Xu XH, 2007 [11]	2005/11	Jingning	Zhejiang	East	Entertainment	--	LA	17	102	16.7%	4

First author, published year	Study period	Location	Province	Region	Recruitment venue	Sampling method	Measurement period*	Number of FSW used condom	Total number of FSW	Condom Usage (%)	QA Score
Ruan SM, 2007 [12]	2005/09	Jinan	Shandong	East	Entertainment	Convenience sampling	LA	34	128	26.6%	2
Zhao XP, 2006 [13]	2005/03	Suzhou	Jiangsu	East	Entertainment	--	LA	6	61	9.8%	4
Wang J, 2008 [14]	2005	Jianhu	Jiangsu	East	Entertainment	--	LA	22	258	8.5%	6
Zhao XP, 2006 [13]	2005/03	Suzhou	Jiangsu	East	Entertainment	--	LA	37	194	19.1%	4
Ruan SM, 2007 [12]	2005/09	Jinan	Shandong	East	Entertainment	Convenience sampling	P1M	34	128	26.6%	2
Zhu FG, 2009 [15]	2006/05	Yancheng	jiangsu	East	Entertainment	--	LA	67	220	30.5%	4
Xu HQ, 2008 [16]	2006	Jiaxing	Zhejiang	East	Entertainment	Random sampling	LA	81	123	65.9%	5
Wang J, 2008 [14]	2006	Jianhu	Jiangsu	East	Entertainment	--	LA	54	268	20.1%	6
Qi GP, 2007 [17]	2006/04-2006/05	Nanjing	Jiangsu	East	Entertainment	--	LA	59	148	39.9%	4
Sun ZF, 2009 [18]	2006/12	Bozhou	Anhui	East	Entertainment	--	LA	8	70	11.4%	3
Sun ZF, 2009 [18]	2006/12	Bozhou	Anhui	East	Entertainment	--	LA	16	70	22.9%	3
Gu YB, 2007 [19]	2006	Funan	Anhui	East	Entertainment	Random sampling	LA	32	82	39.0%	4
Zhang XJ, 2012 [20]	2006	Qingdao, Yantai, Dezhou	Shandong	East	Entertainment	--	LA	176	566	31.1%	4
Yu X, 2007 [21]	2006/05	Haimen	Jiangsu	East	Entertainment	--	LA	31	121	25.6%	5
Wang YF, 2008 [22]	2005-2006	Qingdao	Shandong	East	Entertainment	--	LA	23	68	33.8%	4
Luo Z, 2007 [23]	2006	Shanghai	Shanghai	East	Entertainment	--	LA	20	181	11.0%	4
Zhang XJ, 2012 [20]	2006	Qingdao, Yantai, Dezhou	Shandong	East	Entertainment	--	P1M	102	566	18.0%	4

First author, published year	Study period	Location	Province	Region	Recruitment venue	Sampling method	Measurement period*	Number of FSW used condom	Total number of FSW	Condom Usage (%)	QA Score
Liao M, 2012 [24]	2006	Dezhu, Yantai, Qingdao	Shandong	East	Entertainment	Venue-based sampling	P1M	102	566	18.0%	5
Sun ZF, 2009 [18]	2006/12	Bozhou	Anhui	East	Entertainment	--	P1M	8	70	11.4%	3
Luo Z, 2007 [23]	2006	Shanghai	Shanghai	East	Entertainment	--	P1M	20	181	11.0%	4
Luo Y, 2008 [25]	2007/02-2007/07	Hangzhou	Zhejiang	East	Entertainment	--	LA	12	188	6.4%	5
Luo Y, 2008 [25]	2007/02-2007/07	Hangzhou	Zhejiang	East	Entertainment	--	LA	56	188	29.8%	5
Liao MZ, 2008 [26]	2007	-	Shandong	East	VCT	--	LA	1197	1197	100.0%	4
Chen SP, 2010 [27]	2007/03-2007/04	Xunyang	Jiangxi	East	Entertainment	Convenience sampling	LA	27	154	17.5%	7
Chen SP, 2010 [27]	2007/03-2007/04	Xunyang	Jiangxi	East	Entertainment	Convenience sampling	LA	48	156	30.8%	7
Wang L, 2008 [28]	2007/05-2007/06	Yixing	Jiangsu	East	Entertainment	--	LA	91	235	38.7%	4
Ni YQ, 2008 [29]	2007/07-2007/11	Shanghai	Shanghai	East	Entertainment	--	LA	104	264	39.4%	6
Guo ZY, 2009 [30]	2007/01	Mengcheng	Anhui	East	Entertainment	--	LA	5	121	4.1%	5
Guo ZY, 2009 [30]	2007/01	Mengcheng	Anhui	East	Entertainment	--	LA	13	121	10.7%	5
Wang J, 2008 [14]	2007	Jianhu	Jiangsu	East	Entertainment	--	LA	72	273	26.4%	6
Cheng XL, 2008 [31]	2007/06-2007/11	Funan, Fuyang, Hefei	Anhui	East	Entertainment	Convenience sampling	LA	34	66	51.5%	4
Zhang XJ, 2012 [20]	2007	Qingdao, Yantai, Dezhou	Shandong	East	Entertainment	--	LA	248	482	51.5%	4
Cui W, 2009 [32]	2007/12	Lixin	Anhui	East	Entertainment	Convenience sampling	LA	16	41	39.0%	3
Cui W, 2009 [32]	2007/12	Lixin	Anhui	East	Entertainment	Convenience sampling	LA	20	41	48.8%	3

First author, published year	Study period	Location	Province	Region	Recruitment venue	Sampling method	Measurement period*	Number of FSW used condom	Total number of FSW	Condom Usage (%)	QA Score
Zhang XJ, 2012 [20]	2007	Qingdao, Yantai, Dezhou	Shandong	East	Entertainment	--	P1M	85	482	17.6%	4
Liao MZ, 2008 [26]	2007		Shandong	East	VCT	--	P1M	1197	1197	100.0%	4
Luo Y, 2008 [25]	2007/02-2007/07	Hangzhou	Zhejiang	East	Entertainment	--	P1M	12	188	6.4%	5
Chen SP, 2010 [27]	2007/03-2007/04	Xunyang district	Jiangxi	East	Entertainment	Convenience sampling	P1M	27	154	17.5%	7
Liao M, 2012 [24]	2007	Dezhu, Yantai, Qingdao	Shandong	East	Entertainment	Venue-based sampling	P1M	85	482	17.6%	5
Ni YQ, 2008 [29]	2007/07-2007/11	Shanghai	Shanghai	East	Entertainment	--	P1M	104	264	39.4%	6
Guo ZY, 2009 [24]	2007/01	Mengcheng	Anhui	East	Entertainment	--	P1M	5	121	4.1%	5
Cui W, 2009 [32]	2007/12	Lixin	Anhui	East	Entertainment	Convenience sampling	P1M	16	41	39.0%	3
Jin YL, 2009 [33]	2008/05-2008/06	Wuhu	Anhui	East	Entertainment	Random sampling	LA	15	68	22.1%	4
Jin YL, 2009 [33]	2008/05-2008/06	Wuhu	Anhui	East	Entertainment	Random sampling	LA	22	68	32.4%	4
Jin YL, 2009 [33]	2008/05-2008/06	Wuhu	Anhui	East	Entertainment	Random sampling	LA	80	432	18.5%	4
Jin YL, 2009 [33]	2008/05-2008/06	Wuhu	Anhui	East	Entertainment	Random sampling	LA	148	432	34.3%	4
Wang FH, 2009 [34]	2008	-	Anhui	East	Entertainment	--	LA	348	1973	17.6%	2
Zhang XJ, 2012 [20]	2008	Qingdao, Yantai, Dezhou	Shandong	East	Entertainment	--	LA	237	412	57.5%	4
Yang Y, 2011 [35]	2008/06-2008/10	Shanghai	Shanghai	East	Entertainment	--	LA	44	204	21.6%	5

First author, published year	Study period	Location	Province	Region	Recruitment venue	Sampling method	Measurement period*	Number of FSW used condom	Total number of FSW	Condom Usage (%)	QA Score
Zhang XJ, 2012 [20]	2006-2008	Qingdao, Yantai, Dezhou	Shandong	East	Entertainment	--	LA	661	1460	45.3%	4
He X, 2012 [36]	2008/07	Yu yao	Zhejiang	East	Entertainment	--	LA	78	298	26.2%	4
Zhang XJ, 2012 [20]	2008	Qingdao, Yantai, Dezhou	Shandong	East	Entertainment	--	P1M	167	412	40.5%	4
Liao M, 2012 [24]	2008	Dezhu, Yantai, Qingdao	Shandong	East	Entertainment	Venue-based sampling	P1M	167	412	40.5%	5
Jin YL, 2009 [33]	2008/05-2008/06	Wuhu	Anhui	East	Entertainment	Random sampling	P1M	15	68	22.1%	4
Jin YL, 2009 [33]	2008/05-2008/06	Wuhu	Anhui	East	Entertainment	Random sampling	P1M	80	432	18.5%	4
Liao M, 2012 [37]	2008/02-2008/08	Jinan	Shandong	East	Entertainment	Respondent-Driven Sampling	P1M	50	273	18.3%	6
Cai Y, 2010 [38]	2009/03-2009/12	Shanghai	Shanghai	East	Entertainment	Random sampling	LA	216	324	66.7%	8
Yao Y, 2010 [39]	2009	Fuzhou	Fujian	East	Entertainment	--	LA	8	117	6.8%	3
Wan LJ, 2011 [40]	2008-2009	Shang yu	Zhejiang	East	Entertainment	Two-stage cluster sampling	LA	118	288	41.0%	4
Kang D, 2011 [41]	2006-2009	Qingdao	Shandong	East	Entertainment	Venue-based, community outreach and peer-referral	LA	32	90	35.6%	6
Sun L, 2012 [42]	2009/09-2009/12	3 counties	Jiangsu	East	--	Cluster sampling	LA	441	721	61.2%	2
Chen SX, 2011 [43]	2009/07-2009/09	Gao mi	Shandong	East	Entertainment	--	LA	19	37	51.4%	4

First author, published year	Study period	Location	Province	Region	Recruitment venue	Sampling method	Measurement period*	Number of FSW used condom	Total number of FSW	Condom Usage (%)	QA Score
Yang Y, 2011 [35]	2009/05-2009/08	Shanghai	Shanghai	East	Entertainment	--	LA	192	327	58.7%	5
Kang D, 2011 [41]	2006-2009	Qingdao	Shandong	East	Entertainment	Venue-based, community outreach and peer-referral	LA	93	277	33.6%	6
Kang D, 2011 [41]	2006-2009	Qingdao	Shandong	East	Entertainment	Venue-based, community outreach and peer-referral	P1M	18	281	6.4%	6
Kang D, 2011 [41]	2006-2009	Qingdao	Shandong	East	Entertainment	Venue-based, community outreach and peer-referral	P1M	8	92	8.7%	6
Wan LJ, 2011 [40]	2008-2009	Shang yu	Zhejiang	East	Entertainment	Two-stage cluster sampling	P1M	81	288	28.1%	4
Chen SX, 2011 [43]	2009/07-2009/09	Gao mi	Shandong	East	Entertainment	--	P1M	5	37	13.5%	4
Liao M, 2012 [37]	2009/05-2009/10	Jinan	Shandong	East	Entertainment	Respondent-Driven Sampling	P1M	82	349	23.5%	6
Kang DM, 2011 [44]	2009-2010	Dezhou, dongming, gaomi, lue nan, longkou, pingyi, rushan, yanggu, huaiyin	Shandong	East	Entertainment	Cluster, convenience sampling	LA	351	678	51.8%	3

First author, published year	Study period	Location	Province	Region	Recruitment venue	Sampling method	Measurement period*	Number of FSW used condom	Total number of FSW	Condom Usage (%)	QA Score
Kang DM, 2011 [44]	2009-2010	Heze, caoxian, chengwu, zoucheng, xintai, pingdu	Shandong	East	Entertainment	Cluster, convenience sampling	LA	317	467	67.9%	3
Kang DM, 2011 [44]	2009-2010	Heze, caoxian, chengwu, zoucheng, xintai, pingdu	Shandong	East	Entertainment	Cluster, convenience sampling	P1M	181	481	37.6%	3
Kang DM, 2011 [44]	2009-2010	Dezhou, dongming, gaomi, lue nan, longkou, pingyi, rushan, yanggu, huaiyi	Shandong	East	Entertainment	Cluster, convenience sampling	P1M	194	678	28.6%	3
Chen G, 2012 [45]	2011/07	Dong Yang ,Yu yao, Qu zhou	Zhejiang	East	Entertainment	--	LA	271	514	52.7%	3
Tao SF, 2012 [46]	2011/06-2011/12	Zong yang	Anhui	East	Entertainment	--	LA	5	40	12.5%	2
Sun BJ, 2012 [47]	2011	Zao zhuang	Shandong	East	Entertainment	--	P1M	101	232	43.5%	4
Chen G, 2012 [45]	2011/07	Dong Yang ,Yu yao, Qu zhou	Zhejiang	East	Entertainment	--	P1M	192	514	37.4%	3
Song ZP, 2004 [48]	2002/07-2002/08	Taiyuan	Shanxi	North	Entertainment	--	LA	49	98	50.0%	4
Ren XY, 2006 [49]	2004/07-2004/08	Hehehaote	Inner Mongolia	North	Entertainment	--	LA	16	179	8.9%	4

First author, published year	Study period	Location	Province	Region	Recruitment venue	Sampling method	Measurement period*	Number of FSW used condom	Total number of FSW	Condom Usage (%)	QA Score
Ren XY, 2006 [49]	2004/07-2004/08	Hehehaote	Inner Mongolia	North	Entertainment	--	LA	21	135	15.6%	4
Ren XY, 2006 [49]	2004/07-2004/08	Hehehaote	Inner Mongolia	North	Entertainment	--	LA	37	149	24.8%	4
Ren XY, 2006 [49]	2004/07-2004/08	Hehehaote	Inner Mongolia	North	Entertainment	--	P1M	16	179	8.9%	4
Ren XY, 2006 [49]	2004/07-2004/08	Hehehaote	Inner Mongolia	North	Entertainment	--	P1M	21	135	15.6%	4
Ren XY, 2006 [49]	2004/07-2004/08	Hehehaote	Inner Mongolia	North	Entertainment	--	P1M	37	149	24.8%	4
Li GY, 2008 [50]	2005	Beijing	Beijing	North	Entertainment	Cluster sampling	LA	8	33	24.2%	6
Lin Z, 2007 [51]	2005/08-2005/09	Tongliao	Inner Mongolia	North	Entertainment	Convenience sampling	LA	7	53	13.2%	5
Liu YJ, 2007 [52]	2005/09	Beijing	Beijing	North	Entertainment	--	LA	67	226	29.6%	4
Liu YJ, 2007 [52]	2005/09	Beijing	Beijing	North	Entertainment	--	LA	74	226	32.7%	4
Li GY, 2008 [50]	2005	Beijing	Beijing	North	Entertainment	Cluster sampling	P1M	8	33	24.2%	6
Lin Z, 2007 [51]	2005/08-2005/09	Tongliao	Inner Mongolia	North	Entertainment	Convenience sampling	P1M	7	53	13.2%	5
Liu YJ, 2007 [52]	2005/09	Beijing	Beijing	North	Entertainment	--	P1M	74	226	32.7%	4
Bai JM, 2007 [53]	2006/10-2006/12	Beijing	Beijing	North	Entertainment	Random sampling	LA	31	193	16.1%	4
Shi Y, 2013 [54]	2006/07-2006/10	Hohhot	Inner Mongolia	North	Entertainment	Stratified cluster sampling	LA	188	624	30.1%	5
Ao X, 2008[55]	2006/08-2006/10	Beijing	Beijing	North	Entertainment	Random sampling	LA	14	105	13.3%	7
Liu LR, 2007 [56]	2006/05-2006/10	Beijing	Beijing	North	Entertainment	Venue-based sampling	LA	55	206	26.7%	7

First author, published year	Study period	Location	Province	Region	Recruitment venue	Sampling method	Measurement period*	Number of FSW used condom	Total number of FSW	Condom Usage (%)	QA Score
Liu LR, 2007 [56]	2006/05-2006/10	Beijing	Beijing	North	Entertainment	Venue-based sampling	LA	103	209	49.3%	7
Bo FB, 2007 [57]	2006/08-2006/09	Huhehaote	Inner Mongolia	North	Entertainment	Random sampling	LA	75	246	30.5%	3
Bo FB, 2007 [57]	2006/08-2006/09	Huhehaote	Inner Mongolia	North	Entertainment	Random sampling	LA	33	246	13.4%	3
Shi Y, 2013 [54]	2006/07-2006/10	Hohhot	Inner Mongolia	North	Entertainment	Stratified cluster sampling	P1M	83	624	13.3%	5
Ao X, 2008 [55]	2006/08-2006/10	Beijing	Beijing	North	Entertainment	Random sampling	P1M	14	105	13.3%	7
Liu LR, 2007 [56]	2006/05-2006/10	Beijing	Beijing	North	Entertainment	Venue-based sampling	P1M	55	206	26.7%	7
Bo FB, 2007 [57]	2006/08-2006/09	Huhehaote	Inner Mongolia	North	Entertainment	Random sampling	P1M	33	246	13.4%	3
Shi Y, 2013 [54]	2007/07-2007/10	Hohhot	Inner Mongolia	North	Entertainment	Stratified cluster sampling	LA	111	444	25.0%	5
Liu YQ, 2008 [58]	2006/07-2007/09	Beijing	Beijing	North	Entertainment	Random sampling	LA	41	101	40.6%	4
Shi Y, 2013 [54]	2007/07-2007/10	Hohhot	Inner Mongolia	North	Entertainment	Stratified cluster sampling	P1M	29	444	6.5%	5
Liu YQ, 2008 [58]	2006/07-2007/09	Beijing	Beijing	North	Entertainment	Random sampling	P1M	41	101	40.6%	4
Shi Y, 2013 [54]	2008/07-2008/10	Hohhot	Inner Mongolia	North	Entertainment	Stratified cluster sampling	LA	176	451	39.0%	5
Shi Y, 2013 [54]	2008/07-2008/10	Hohhot	Inner Mongolia	North	Entertainment	Stratified cluster sampling	P1M	81	451	18.0%	5

First author, published year	Study period	Location	Province	Region	Recruitment venue	Sampling method	Measurement period*	Number of FSW used condom	Total number of FSW	Condom Usage (%)	QA Score
Cao H, 2010 [59]	2009/07-2010/06	Tianjin	Tianjin	North	Entertainment	Random sampling	LA	25	31	80.6%	5
Cao H, 2010 [59]	2009/07-2010/06	Tianjin	Tianjin	North	Entertainment	Random sampling	LA	18	32	56.3%	5
Cao H, 2010 [59]	2009/07-2010/06	Tianjin	Tianjin	North	Entertainment	Random sampling	P1M	18	32	56.3%	5
Shao B, 2011 [60]	2008	13 cities	Heilongjiang	Northeast	Entertainment	--	P1M	916	5055	18.1%	4
Lin B, 2002 [61]	2000/08-2000/11	Karamay	Xinjiang	Northwest	Entertainment	--	LA	19	198	9.6%	4
Zhang M, 2006 [62]	1999-2004	Wulumuqi	Xinjiang	Northwest	Detention Center	--	LA	13	93	14.0%	3
Song YR, 2005 [63]	2004/08	Alaer	Xinjiang	Northwest	Entertainment	--	LA	22	200	11.0%	4
Song YR, 2005 [63]	2004/08	Alaer	Xinjiang	Northwest	Entertainment	--	P1M	22	200	11.0%	4
Lin L, 2009 [64]	2005	Karamay	Xinjiang	Northwest	Sentinel sites	Continuous sampling	LA	9	55	16.4%	4
Lin L, 2009 [64]	2005	Karamay	Xinjiang	Northwest	Sentinel sites	Continuous sampling	P1M	9	55	16.4%	4
Liu YX, 2007 [65]	2006/08-2006/11	Yinchuan	Ningxia	Northwest	Entertainment	--	LA	74	184	40.2%	4
Zhang L, 2007 [66]	2005/11-2006/05	Lanzhou	Gansu	Northwest	Entertainment	--	LA	110	376	29.3%	1
Zhang L, 2007 [66]	2005/11-2006/05	Lanzhou	Gansu	Northwest	Entertainment	--	LA	99	376	26.3%	1
Lin L, 2009 [64]	2006	Karamay	Xinjiang	Northwest	Sentinel sites	Continuous sampling	LA	95	519	18.3%	4
Zeng KF, 2008 [67]	2006/01-2006/02	Kelamayi	Xinjiang	Northwest	Entertainment	--	LA	94	747	12.6%	3
Lin L, 2009 [64]	2006	Karamay	Xinjiang	Northwest	Sentinel sites	Continuous sampling	P1M	95	519	18.3%	4

First author, published year	Study period	Location	Province	Region	Recruitment venue	Sampling method	Measurement period*	Number of FSW used condom	Total number of FSW	Condom Usage (%)	QA Score
Zeng KF, 2008 [67]	2006/01-2006/02	Kelamayi	Xinjiang	Northwest	Entertainment	--	P1M	94	747	12.6%	3
Lin L, 2009 [64]	2007	Karamay	Xinjiang	Northwest	Sentinel sites	Continuous sampling	LA	38	178	21.3%	4
Lin B, 2009 [68]	2007/01	Karamay	Xinjiang	Northwest	Entertainment	--	LA	38	178	21.3%	1
Amydam MAYT, 2008 [69]	2007	Wulumuqi	Xinjiang	Northwest	Entertainment	Convenience sampling	LA	157	650	24.2%	7
Lin L, 2009 [64]	2007	Karamay	Xinjiang	Northwest	Sentinel sites	Continuous sampling	P1M	38	178	21.3%	4
Amydam MAYT, 2008 [69]	2007	Wulumuqi	Xinjiang	Northwest	Entertainment	Convenience sampling	P1M	157	650	24.2%	7
Lin B, 2009 [68]	2007/01	Karamay	Xinjiang	Northwest	Entertainment	--	P1M	38	178	21.3%	1
Liu SH, 2009 [70]	2008/04-2008/05	Huinong	Ningxia	Northwest	Entertainment	Random sampling	LA	5	130	3.8%	7
Zhang XQ, 2010 [71]	2008/01	Gaolan	Gansu	Northwest	Entertainment	--	LA	7	150	4.7%	3
Zhang XQ, 2010 [71]	2008/01	Gaolan	Gansu	Northwest	Entertainment	--	LA	15	150	10.0%	3
Liu SH, 2009 [70]	2008/04-2008/05	Huinong	Ningxia	Northwest	Entertainment	Random sampling	LA	26	236	11.0%	7
Lin L, 2009 [64]	2008	Karamay	Xinjiang	Northwest	Sentinel sites	Continuous sampling	LA	36	212	17.0%	4
Liu SH, 2009 [70]	2008/04-2008/05	Huinong	Ningxia	Northwest	Entertainment	Random sampling	P1M	5	130	3.8%	7
Zhang XQ, 2010 [71]	2008/01	Gaolan	Gansu	Northwest	Entertainment	--	P1M	7	150	4.7%	3
Liu SH, 2009 [70]	2008/04-2008/05	Huinong	Ningxia	Northwest	Entertainment	Random sampling	P1M	26	236	11.0%	7
Lin L, 2009 [64]	2008	Karamay	Xinjiang	Northwest	Sentinel sites	Continuous sampling	P1M	36	212	17.0%	4

First author, published year	Study period	Location	Province	Region	Recruitment venue	Sampling method	Measurement period*	Number of FSW used condom	Total number of FSW	Condom Usage (%)	QA Score
Gao W, 2012 [72]	2008/11-2009/01	Chenguan (lanzhou)	Gansu	Northwest	Entertainment	Proportional stratified random sampling	LA	63	164	38.4%	7
Gao W, 2012 [72]	2008/11-2009/01	Chenguan (lanzhou)	Gansu	Northwest	Entertainment	Proportional stratified random sampling	P1M	32	164	19.5%	7
Ma L, 2011 [73]	2009/06	Hu tu bi	Xinjiang	Northwest	Entertainment	--	P1M	30	146	20.5%	4
Tao LD, 2011 [74]	2010/04-2010/10	Lan zhou	Gansu	Northwest	Entertainment	--	LA	305	596	51.2%	4
Tao LD, 2011 [74]	2010/04-2010/10	Lan zhou	Gansu	Northwest	Entertainment	--	P1M	259	596	43.5%	4
Liu DF, 2012 [75]	2011	Yan an	Shaanxi	Northwest	Entertainment	--	P1M	85	235	36.2%	4
Qu S, 2002 [76]	2000/10-2000/12	Baise city	Guangxi	South Central	Entertainment	--	LA	27	305	8.9%	4
Zhu GR, 2003 [77]	2001/07	-	Guangxi	South Central	Entertainment	--	LA	11	120	9.2%	3
Wei MG, 2004 [78]	2003	Haikou,sanya	Hainan	South Central	Entertainment	--	LA	37	338	10.9%	3
Li N, 2005 [79]	2003/04	Henan	Henan	South Central	--	Cluster Random sampling	LA	126	922	13.7%	5
Li N, 2005 [79]	2003/04	Henan	Henan	South Central	--	Cluster Random sampling	P1M	126	922	13.7%	5
Wei MG, 2004 [78]	2003	Haikou,sanya	Hainan	South Central	Entertainment	--	P1M	37	338	10.9%	3
Yang BF, 2006 [80]	2004/08-2004/11	-	Hubei	South Central	Entertainment	--	LA	88	588	15.0%	2

First author, published year	Study period	Location	Province	Region	Recruitment venue	Sampling method	Measurement period*	Number of FSW used condom	Total number of FSW	Condom Usage (%)	QA Score
Yang BF, 2006 [81]	2004/09	-	Hubei	South Central	Entertainment	--	LA	53	374	14.2%	2
Yang BF, 2006 [80]	2004/08-2004/11	-	Hubei	South Central	Entertainment	--	LA	209	588	35.5%	2
Yang BF, 2006 [81]	2004/09	-	Hubei	South Central	Entertainment	--	LA	124	374	33.2%	2
LI Q, 2011 [82]	2004	Guangxi	Guangxi	South Central	Entertainment	Ethnographic target sampling	LA	60	395	15.2%	5
Yang BF, 2006 [83]	2004/08-2004/11	Wuhan	Hubei	South Central	Entertainment	--	LA	86	473	18.2%	3
Yang BF, 2006 [83]	2004/08-2004/11	Wuhan	Hubei	South Central	Entertainment	--	LA	197	473	41.6%	3
Yang F, 2006 [84]	2004	Doumen	Guangdong	South Central	--	Convenience sampling	LA	5	76	6.6%	3
Yang F, 2006 [84]	2004	Yangdong	Guangdong	South Central	--	Convenience sampling	LA	2	110	1.8%	3
Yang F, 2006 [84]	2004	Taishan	Guangdong	South Central	--	Convenience sampling	LA	24	121	19.8%	3
Yang F, 2006 [84]	2004	Taishan, yangdong, doumen	Guangdong	South Central	--	Convenience sampling	LA	31	307	10.1%	3
Jiang M, 2005 [85]	2004/08-2004/10	Jingzhou	Hubei	South Central	Entertainment	Random sampling	LA	41	139	29.5%	4
Wang H, 2005 [86]	2004/03-2004/05	H county (Nanning)	Guangxi	South Central	Entertainment	Ethnographic target sampling	LA	43	308	14.0%	4
Luo J, 2005 [87]	2004/11	Liuzhou	Guangxi	South Central	Entertainment	--	LA	106	234	45.3%	6
Yang BF, 2006 [81]	2004/09	-	Hubei	South Central	Entertainment	--	P1M	53	374	14.2%	2

First author, published year	Study period	Location	Province	Region	Recruitment venue	Sampling method	Measurement period*	Number of FSW used condom	Total number of FSW	Condom Usage (%)	QA Score
Yang BF, 2006 [80]	2004/08-2004/11	-	Hubei	South Central	Entertainment	--	P1M	88	588	15.0%	2
Yang BF, 2006 [83]	2004/08-2004/11	Wuhan	Hubei	South Central	Entertainment	--	P1M	86	473	18.2%	3
Zhou YJ, 2006 [88]	2005	-	Guangxi	South Central	Entertainment	--	LA	7	32	21.9%	4
Lu F, 2009 [18]	2005	Liuzhou	Guangxi	South Central	--	Snowball sampling	LA	57	198	28.8%	3
Xu YF, 2007 [89]	2005/07-2005/09	Nanning	Guangxi	South Central	Entertainment	Random sampling	LA	40	193	20.7%	7
Lu F, 2009 [18]	2005	Liuzhou	Guangxi	South Central	--	Snowball sampling	P1M	57	198	28.8%	3
Xu XY, 2007 [90]	2006/04-2006/06	Guangzhou	Guangdong	South Central	Entertainment	--	LA	82	355	23.1%	3
Zhou YJ, 2008 [91]	2007/04-2007/05	-	Guangxi	South Central	--	--	LA	11	34	32.4%	7
Wen XQ, 2009 [92]	2007/04-2007/05	Guilin	Guangxi	South Central	Entertainment	Stratified cluster and random sampling	LA	83	360	23.1%	3
Wen XQ, 2009 [92]	2007/04-2007/05	Guilin	Guangxi	South Central	Entertainment	Stratified cluster and random sampling	LA	173	360	48.1%	3
Tan WW, 2008 [93]	2007/07-2007/09	Nanning	Guangxi	South Central	Entertainment	--	LA	30	204	14.7%	4
Tan WW, 2008 [93]	2007/07-2007/09	Nanning	Guangxi	South Central	Entertainment	--	LA	77	204	37.7%	4
Lu WJ, 2008 [94]	2007	20 cities/counties of Guangxi	Guangxi	South Central	Entertainment	--	LA	309	1882	16.4%	6

First author, published year	Study period	Location	Province	Region	Recruitment venue	Sampling method	Measurement period*	Number of FSW used condom	Total number of FSW	Condom Usage (%)	QA Score
Lu WJ, 2008 [94]	2007	20 cities/counties of Guangxi	Guangxi	South Central	Entertainment	--	LA	792	1882	42.1%	6
Li Y, 2009 [95]	2006/08-2007/01	Guangdong	Guangdong	South Central	Entertainment	Respondent-Driven Sampling	LA	124	320	38.8%	6
He Y, 2009 [96]	2007/07	Nanning	Guangxi	South Central	Entertainment	Random sampling	LA	38	206	18.4%	5
He Y, 2009 [96]	2007/07	Nanning	Guangxi	South Central	Entertainment	Random sampling	LA	88	206	42.7%	5
He Y, 2009 [96]	2007/07	Nanning	Guangxi	South Central	Entertainment	Random sampling	LA	38	206	18.4%	5
Zhang YX, 2011 [97]	2007/07-2007/09	Liu zhou	Guangxi	South Central	Entertainment	--	LA	28	67	41.8%	4
Zhang YX, 2011 [97]	2007/07-2007/09	Liu zhou	Guangxi	South Central	Entertainment	--	LA	142	314	45.2%	4
Zhang YX, 2011 [97]	2007/07-2007/09	Liu zhou	Guangxi	South Central	Entertainment	--	LA	114	247	46.2%	4
Jiang M, 2012 [98]	2007/07-2007/10	Jing zhou	Hubei	South Central	Entertainment	--	LA	41	139	29.5%	4
Zhou JH, 2010 [99]	2007/07-2007/09	Shenzhen	Guangdong	South Central	Entertainment	--	LA	188	284	66.2%	4
Chen Y, 2010 [100]	2006/12-2007/02	N/a	Guangxi	South Central	Entertainment	Stratified cluster sampling, convenience sampling	LA	3	49	6.1%	2
Jiang M, 2012 [98]	2007/07-2007/10	Jing zhou	Hubei	South Central	Entertainment	--	P1M	11	139	7.9%	4

First author, published year	Study period	Location	Province	Region	Recruitment venue	Sampling method	Measurement period*	Number of FSW used condom	Total number of FSW	Condom Usage (%)	QA Score
Wen XQ, 2009 [92]	2007/4-2007/5	Guilin	Guangxi	South Central	Entertainment	Stratified cluster and random sampling	P1M	83	360	23.1%	3
Tan WW, 2008 [93]	2007/7-2007/9	Nanning	Guangxi	South Central	Entertainment	--	P1M	30	204	14.7%	4
Chen Y, 2010 [100]	2006/12-2007/02	N/a	Guangxi	South Central	Entertainment	Stratified cluster sampling, convenience sampling	P1M	3	49	6.1%	2
He Y, 2009 [96]	2007/07	Nanning	Guangxi	South Central	Entertainment	Random sampling	P1M	38	206	18.4%	5
He Y, 2009 [96]	2007/07	Nanning	Guangxi	South Central	Entertainment	Random sampling	P1M	38	206	18.4%	5
Zhang YX, 2011 [97]	2007/07-2007/09	Liu zhou	Guangxi	South Central	Entertainment	--	P1M	13	67	19.4%	4
Zhang YX, 2011 [97]	2007/07-2007/09	Liu zhou	Guangxi	South Central	Entertainment	--	P1M	71	249	28.5%	4
Zhang YX, 2011 [97]	2007/07-2007/09	Liu zhou	Guangxi	South Central	Entertainment	--	P1M	84	316	26.6%	4
Zhao YY, 2010 [101]	2008/10-2008/12	Guangzhou	Guangdong	South Central	Entertainment	--	LA	58	622	9.3%	4
Tan JG, 2009 [102]	2008/4-2008/5	Shenzhen	Guangdong	South Central	Entertainment	Stratified cluster sampling	LA	20	139	14.4%	5
Tan JG, 2009 [102]	2008/4-2008/5	Shenzhen	Guangdong	South Central	Entertainment	Stratified cluster sampling	LA	57	139	41.0%	5
Huang XT, 2009 [103]	2008	Shantou	Guangdong	South Central	Entertainment	--	LA	46	127	36.2%	4

First author, published year	Study period	Location	Province	Region	Recruitment venue	Sampling method	Measurement period*	Number of FSW used condom	Total number of FSW	Condom Usage (%)	QA Score
Zhang L, 2010 [104]	2008/12	Shangcai	Henan	South Central	Entertainment	--	LA	20	106	18.9%	3
Zhang L, 2010 [104]	2008/12	Shangcai	Henan	South Central	Entertainment	--	LA	46	106	43.4%	3
BaiY, 2010 [105]	2008/04-2008/07	Liuzhou	Guangxi	South Central	--	Continuous sampling	LA	85	353	24.1%	5
He Y, 2009 [96]	2007/07	Nanning	Guangxi	South Central	Entertainment	Random sampling	LA	88	206	42.7%	5
BaiY, 2010 [105]	2008/04-2008/07	Liuzhou	Guangxi	South Central	--	Continuous sampling	LA	145	353	41.1%	5
Tan JG, 2009 [102]	2008/04-2008/05	Shenzhen	Guangdong	South Central	Entertainment	Stratified cluster sampling	P1M	20	139	14.4%	5
Zhang L, 2010 [104]	2008/12	Shangcai	Henan	South Central	Entertainment	--	P1M	20	106	18.9%	3
Wang JY, 2010 [106]	2009/05	Zhongshan	Guangdong	South Central	Community	Convenience sampling	LA	129	245	52.7%	4
Zeng XL, 2011 [107]	2009/11	Nanyang	Henan	South Central	Entertainment	--	LA	123	457	26.9%	4
Wang JY, 2010 [108]	2009/05	Zhongshan	Guangdong	South Central	Community	Convenience sampling	LA	46	242	19.0%	4
Hu SX, 2010 [109]	2009/10-2009/12	Qingyuan	Guangdong	South Central	Entertainment	Random sampling	LA	143	282	50.7%	4
Hu SX, 2010 [109]	2009/10-2009/12	Qingyuan	Guangdong	South Central	Entertainment	Random sampling	LA	116	282	41.1%	4
Dun ZJ, 2011 [110]	2007-2009	Guang zhou	Guangdong	South Central	Entertainment	--	LA	47	204	23.0%	3
Zeng XL, 2011 [107]	2009/11	Nanyang	Henan	South Central	Entertainment	--	P1M	91	457	19.9%	4
Liao S, 2011 [111]	2008-2009		Hainan	South Central	Entertainment	Venue-based sampling	P1M	26	90	28.9%	4

First author, published year	Study period	Location	Province	Region	Recruitment venue	Sampling method	Measurement period*	Number of FSW used condom	Total number of FSW	Condom Usage (%)	QA Score
Xiong CS, 2012 [112]	2010	Shi yan	Hubei	South Central	Entertainment	Two-stage sampling	LA	89	136	65.4%	4
Jiang N, 2012 [113]	2009-2010	Zhu ma dian	Henan	South Central	--	--	LA	50	475	10.5%	3
He B, 2011 [114]	2010/03-2010/08	Qiong hai	Hainan	South Central	Entertainment	--	P1M	24	109	22.0%	3
Zhu L, 2011 [115]	2010/04-2010/06	Xiang yang	Hubei	South Central	Sentinel sites	--	P1M	193	402	48.0%	4
Liao S, 2011 [111]	2009 - 2010		Guangxi	South Central	Entertainment	Venue-based sampling	P1M	34	99	34.3%	4
Yang HW, 2003 [116]	2002/04-2002/05	Mianyang	Sichuan	Southwest	Entertainment	--	LA	30	71	42.3%	2
Huang ZM, 2006 [117]	2003/08	Luxi	Yunnan	Southwest	Entertainment	The proportion of two-stage probability sampling	LA	20	91	22.0%	3
Huang ZM, 2006 [117]	2003/08	Luxi	Yunnan	Southwest	Entertainment	The proportion of two-stage probability sampling	LA	38	91	41.8%	3
Li JE, 2006 [118]	2003/07	Yuxi	Yunnan	Southwest	Entertainment	--	LA	15	90	16.7%	5
Li JE, 2006 [118]	2003/07	Yuxi	Yunnan	Southwest	Entertainment	--	LA	22	90	24.4%	5
Huang LH, 2006 [119]	2003	Dali	Yunnan	Southwest	Entertainment	Random sampling	LA	58	234	24.8%	4
Huang LH, 2006 [119]	2003	Dali	Yunnan	Southwest	Entertainment	Random sampling	LA	108	234	46.2%	4
Wang LL, 2004 [120]	2003/06-2003/07	Leshan	Sichuan	Southwest	Entertainment	Convenience sampling	LA	41	192	21.4%	4
Wang LL, 2004 [120]	2003/06-2003/07	Leshan	Sichuan	Southwest	Entertainment	Convenience sampling	LA	81	193	42.0%	4

First author, published year	Study period	Location	Province	Region	Recruitment venue	Sampling method	Measurement period*	Number of FSW used condom	Total number of FSW	Condom Usage (%)	QA Score
Jin Y, 2006 [121]	2003/03	Qujing	Yunna	Southwest	Sentinel sites	Random sampling	LA	45	277	16.2%	6
Zhang YL, 2006 [122]	2003	Yuxi	Yunnan	Southwest	Entertainment	--	LA	37	184	20.1%	4
Zhang YL, 2006 [122]	2003	Yuxi	Yunnan	Southwest	Entertainment	--	LA	81	184	44.0%	4
Li SJ, 2004 [123]	2003/06-2003/07	Bazhong	Sichuan	Southwest	Entertainment	--	LA	48	156	30.8%	4
Lau JT, 2011 [124]	2003	Multiple locations In Sichuan	Sichuan	Southwest	Entertainment	Convenience sampling	LA	1123	7063	15.9%	5
Lau JT, 2011 [124]	2003	Multiple locations In Sichuan	Sichuan	Southwest	Entertainment	Convenience sampling	LA	2691	7063	38.1%	5
Lau JT, 2007 [125]	2003	City TC, DC	Sichuan	Southwest	Entertainment	Convenience sampling	LA	27	402	6.7%	4
Lau JT, 2007 [125]	2003	City TC, DC	Sichuan	Southwest	Entertainment	Convenience sampling	LA	93	402	23.1%	4
Jiang HY, 2006 [126]	2003/10	Yuanjiang	Yunnan	Southwest	Entertainment	--	LA	4	216	1.9%	5
Jiang HY, 2006 [126]	2003/10	Yuanjiang	Yunnan	Southwest	Entertainment	--	LA	54	216	25.0%	5
Lai WH, 2009 [127]	2003	-	Sichuan	Southwest	Entertainment	Snowball sampling	LA	488	1421	34.3%	4
Huang ZM, 2006 [117]	2003/08	Luxi	Yunnan	Southwest	Entertainment	The proportion of two-stage probability sampling	P1M	20	91	22.0%	3
Li JE, 2006 [118]	2003/07	Yuxi	Yunnan	Southwest	Entertainment	--	P1M	15	90	16.7%	5
Huang LH, 2006 [119]	2003	Dali	Yunnan	Southwest	Entertainment	Random sampling	P1M	58	234	24.8%	4
Wang LL, 2004 [120]	2003/6-2003/7	Leshan	Sichuan	Southwest	Entertainment	Convenience sampling	P1M	41	192	21.4%	4

First author, published year	Study period	Location	Province	Region	Recruitment venue	Sampling method	Measurement period*	Number of FSW used condom	Total number of FSW	Condom Usage (%)	QA Score
Jin Y, 2006 [121]	2003/03	Qujing	Yunna	Southwest	Sentinel sites	Random sampling	P1M	45	277	16.2%	6
Zhang YL, 2006 [122]	2003	Yuxi	Yunnan	Southwest	Entertainment	--	P1M	37	184	20.1%	4
Jiang HY, 2006 [126]	2003/10	Yuanjiang	Yunnan	Southwest	Entertainment	--	P1M	4	216	1.9%	5
Lau JT, 2007 [125]	2003	City TC, DC	Sichuan	Southwest	Entertainment	Convenience sampling	P1M	27	402	6.7%	4
Lau JT, 2011 [124]	2003	Multiple locations In Sichuan	Sichuan	Southwest	Entertainment	Convenience sampling	P1M	1123	7063	15.9%	5
Su DT, 2005 [128]	2004/3	Chengdu	Sichuan	Southwest	Entertainment	--	LA	59	112	52.7%	4
Zhao R, 2008 [129]	2004/03-2004/05	H country	Guangxi	Southwest	Entertainment	--	LA	82	309	26.5%	3
Lau JT, 2011 [124]	2004	Multiple locations In Sichuan	Sichuan	Southwest	Entertainment	Convenience sampling	LA	1107	6875	16.1%	5
Lau JT, 2011 [124]	2004	Multiple locations In Sichuan	Sichuan	Southwest	Entertainment	Convenience sampling	LA	2509	6875	36.5%	5
Lau JT, 2007 [125]	2004	City TC, DC	Sichuan	Southwest	Entertainment	Convenience sampling	LA	79	380	20.8%	4
Lau JT, 2007 [125]	2004	City TC, DC	Sichuan	Southwest	Entertainment	Convenience sampling	LA	125	380	32.9%	4
Lau JT, 2007 [130]	2002-2004	Multiple locations In Sichuan	Sichuan	Southwest	Entertainment	Convenience sampling	LA	1287	8300	15.5%	4
Lau JT, 2007 [130]	2002-2004	Multiple locations In Sichuan	Sichuan	Southwest	Entertainment	Convenience sampling	LA	3086	8313	37.1%	4
Jiang M, 2005 [85]	2004/08-2004/10	Jingzhou	Hubei	Southwest	Entertainment	Random sampling	LA	11	139	7.9%	4

First author, published year	Study period	Location	Province	Region	Recruitment venue	Sampling method	Measurement period*	Number of FSW used condom	Total number of FSW	Condom Usage (%)	QA Score
Zou YD, 2006 [131]	2004/04	Chuxiong	Yunnan	Southwest	Entertainment	--	LA	50	233	21.5%	6
Zou YD, 2006 [131]	2004/04	Chuxiong	Yunnan	Southwest	Entertainment	--	LA	84	233	36.1%	6
Wen Y, 2006 [132]	2004/03	Gejiu	Yunnan	Southwest	Mixed venues	--	LA	38	415	9.2%	4
Wen Y, 2006 [132]	2004/03	Gejiu	Yunnan	Southwest	Mixed venues	--	LA	73	415	17.6%	4
Lau JT, 2007 [130]	2002-2004	Multiple locations In Sichuan	Sichuan	Southwest	Entertainment	Convenience sampling	P1M	1287	8300	15.5%	4
Lau JT, 2007 [125]	2004	City TC, DC	Sichuan	Southwest	Entertainment	Convenience sampling	P1M	79	380	20.8%	4
Lau JT, 2011 [124]	2004	Multiple locations In Sichuan	Sichuan	Southwest	Entertainment	Convenience sampling	P1M	1107	6875	16.1%	5
Jiang M, 2005 [85]	2004/08-2004/10	Jingzhou	Hubei	Southwest	Entertainment	Random sampling	P1M	11	139	7.9%	4
Wen Y, 2006 [132]	2004/03	Gejiu	Yunnan	Southwest	Mixed venues	--	P1M	38	415	9.2%	4
Tan XJ, 2007 [133]	2005/8-2005/9	Chongqing	Chongqing	Southwest	Entertainment	--	LA	33	229	14.4%	5
Zeng K, 2007 [134]	2002-2005	Aba	Sichuan	Southwest	Entertainment	--	LA	56	872	6.4%	2
Zeng K, 2007 [134]	2002-2005	Aba	Sichuan	Southwest	Entertainment	--	LA	186	872	21.3%	2
Ruan Y, 2006 [135]	2004/12-2005/01	Xichang	Sichuan	Southwest	Entertainment	--	LA	144	343	42.0%	5
Ding XB, 2006 [136]	2005/08-2005/09	Chongqing	Chongqing	Southwest	Entertainment	Convenience sampling	LA	6	426	1.4%	4
Zhang Q, 2006 [137]	2005	Nanchong	Sichuan	Southwest	Entertainment	Random sampling	LA	16	162	9.9%	2
Zhang Q, 2006 [137]	2005	Nanchong	Sichuan	Southwest	Entertainment	Random sampling	LA	36	162	22.2%	2

First author, published year	Study period	Location	Province	Region	Recruitment venue	Sampling method	Measurement period*	Number of FSW used condom	Total number of FSW	Condom Usage (%)	QA Score
Yuzhen LC, 2006 [138]	2005/06-2005/08	Lasa	Tibet	Southwest	Entertainment	--	LA	76	892	8.5%	2
Yuan MJ, 2006 [139]	2005/03	Deyang	Sichuan	Southwest	Entertainment	--	LA	10	30	33.3%	4
Yuan MJ, 2006 [139]	2005/03	Deyang	Sichuan	Southwest	Entertainment	--	LA	16	30	53.3%	4
Wang JH, 2006 [140]	2005/02	Huaning	Yunnan	Southwest	Entertainment	--	LA	1	100	1.0%	6
Wang JH, 2006 [140]	2005/02	Huaning	Yunnan	Southwest	Entertainment	--	LA	13	100	13.0%	6
Chen CL, 2006 [141]	2005/03	Malong	Yunnan	Southwest	Entertainment	Random sampling	LA	20	89	22.5%	7
Chen CL, 2006 [141]	2005/03	Malong	Yunnan	Southwest	Entertainment	Random sampling	LA	4	89	4.5%	7
Luo GY, 2006 [142]	2005/08-2005/09	Meishan	Sichuan	Southwest	Entertainment	Convenience sampling	LA	9	50	18.0%	3
Luo GY, 2006 [142]	2005/08-2005/09	Meishan	Sichuan	Southwest	Entertainment	Convenience sampling	LA	18	50	36.0%	3
Zhang Q, 2006 [137]	2005	Nanchong	Sichuan	Southwest	Entertainment	Random sampling	LA	14	72	19.4%	2
Zhang Q, 2006 [137]	2005	Nanchong	Sichuan	Southwest	Entertainment	Random sampling	LA	22	72	30.6%	2
Luo GY, 2006 [142]	2005/08-2005/09	Meishan	Sichuan	Southwest	Entertainment	Convenience sampling	LA	13	109	11.9%	3
Luo GY, 2006 [142]	2005/08-2005/09	Meishan	Sichuan	Southwest	Entertainment	Convenience sampling	LA	38	109	34.9%	3
Jiang ZQ, 2006 [143]	2004/12-2005/01	Xichang	Sichuan	Southwest	Entertainment	--	LA	44	105	41.9%	4
Lau JT, 2011 [124]	2005	Multiple locations In Sichuan	Sichuan	Southwest	Entertainment	Convenience sampling	LA	1473	6853	21.5%	5
Lau JT, 2011 [124]	2005	Multiple locations In Sichuan	Sichuan	Southwest	Entertainment	Convenience sampling	LA	3139	6853	45.8%	5

First author, published year	Study period	Location	Province	Region	Recruitment venue	Sampling method	Measurement period*	Number of FSW used condom	Total number of FSW	Condom Usage (%)	QA Score
Lau JT, 2007 [125]	2005	City TC, DC	Sichuan	Southwest	Entertainment	Convenience sampling	LA	66	371	17.8%	4
Lau JT, 2007 [125]	2005	City TC, DC	Sichuan	Southwest	Entertainment	Convenience sampling	LA	131	371	35.3%	4
Wang Y, 2007 [144]	2005	Mianyang	Sichuan	Southwest	Entertainment	Convenience sampling	LA	77	657	11.7%	4
Wang Y, 2007 [144]	2005	Mianyang	Sichuan	Southwest	Entertainment	Convenience sampling	LA	236	657	35.9%	4
Zou YD, 2006 [131]	2005/05	Chuxiong	Yunnan	Southwest	Entertainment	--	LA	46	156	29.5%	6
Zou YD, 2006 [131]	2005/05	Chuxiong	Yunnan	Southwest	Entertainment	--	LA	74	156	47.4%	6
Chen XH, 2006 [145]	2004/12-2005/01	Xichang	Sichuan	Southwest	Entertainment	--	LA	44	105	41.9%	6
Wang YH, 2009 [146]	2005/8	Shangri-la	Yunnan	Southwest	Entertainment	--	LA	67	191	35.1%	3
Wang C, 2007 [147]	2005/12	Simao	Yunnan	Southwest	Entertainment	Random sampling	LA	103	250	41.2%	4
Tian LG, 2006 [148]	2004/12-2005/1	Xichang	Sichuan	Southwest	Community	--	LA	275	343	80.2%	4
Cao XY, 2007 [149]	2004/12-2005/01	Xichang	Sichuan	Southwest	Entertainment	--	LA	138	330	41.8%	5
Cao XY, 2006 [150]	2004/12-2005/1	Xichang	Sichuan	Southwest	Entertainment	--	LA	168	402	41.8%	4
Li L, 2007 [151]	2005/12	Kunming	Yunnan	Southwest	Entertainment	Random sampling	LA	529	1222	43.3%	5
Ruan Y, 2006 [135]	2004/12-2005/01	Xichang	Sichuan	Southwest	Entertainment	--	P1M	144	343	42.0%	5
Chen CL, 2006 [141]	2005/03	Malong	Yunnan	Southwest	Entertainment	Random sampling	P1M	4	89	4.5%	7
Luo GY, 2006 [142]	2005/08-2005/09	Meishan	Sichuan	Southwest	Entertainment	Convenience sampling	P1M	9	50	18.0%	3
Wang JH, 2006 [140]	2005/02	Huaning	Yunnan	Southwest	Entertainment	--	P1M	1	100	1.0%	6

First author, published year	Study period	Location	Province	Region	Recruitment venue	Sampling method	Measurement period*	Number of FSW used condom	Total number of FSW	Condom Usage (%)	QA Score
Yuan MJ, 2006 [139]	2005/03	Deyang	Sichuan	Southwest	Entertainment	--	P1M	10	30	33.3%	4
Zhang Q, 2006 [137]	2005	Nanchong	Sichuan	Southwest	Entertainment	Random sampling	P1M	16	162	9.9%	2
Luo GY, 2006 [142]	2005/08-2005/09	Meishan	Sichuan	Southwest	Entertainment	Convenience sampling	P1M	13	109	11.9%	3
Zhang Q, 2006 [137]	2005	Nanchong	Sichuan	Southwest	Entertainment	Random sampling	P1M	14	72	19.4%	2
Jiang ZQ, 2006 [143]	2004/12-2005/01	Xichang	Sichuan	Southwest	Entertainment	--	P1M	44	105	41.9%	4
Lau JT, 2007 [125]	2005	City TC, DC	Sichuan	Southwest	Entertainment	Convenience sampling	P1M	66	371	17.8%	4
Lau JT, 2011 [124]	2005	Multiple locations In Sichuan	Sichuan	Southwest	Entertainment	Convenience sampling	P1M	1473	6853	21.5%	5
Wang Y, 2007 [144]	2005	Mianyang	Sichuan	Southwest	Entertainment	Convenience sampling	P1M	77	657	11.7%	4
Cao XY, 2006 [150]	2004/12-2005/1	Xichang	Sichuan	Southwest	Entertainment	--	P1M	168	402	41.8%	4
Cao XY, 2007 [149]	2004/12-2005/01	Xichang	Sichuan	Southwest	Entertainment	--	P1M	138	330	41.8%	5
Chen XH, 2006 [145]	2004/12-2005/01	Xichang	Sichuan	Southwest	Entertainment	--	P1M	44	105	41.9%	6
Li L, 2007 [151]	2005/12	Kunming	Yunnan	Southwest	Entertainment	Random sampling	P1M	529	1222	43.3%	5
Tian LG, 2006 [148]	2004/12-2005/1	Xichang	Sichuan	Southwest	Community	--	P1M	275	343	80.2%	4
Li JE, 2006 [118]	2003/10-2006/04	Yuxi	Yunnan	Southwest	Entertainment	--	LA	52	96	54.2%	5
Li DM, 2007 [152]	2006	Guiyang	Guizhou	Southwest	Entertainment	Random sampling	LA	31	166	18.7%	7

First author, published year	Study period	Location	Province	Region	Recruitment venue	Sampling method	Measurement period*	Number of FSW used condom	Total number of FSW	Condom Usage (%)	QA Score
Li DM, 2007 [152]	2006	Guiyang	Guizhou	Southwest	Entertainment	Random sampling	LA	82	166	49.4%	7
Wang H, 2009 [153]	2006/03-2006/05	Kaiyuan	Yunnan	Southwest	Entertainment	Convenience sampling	LA	41	458	9.0%	4
Du JQ, 2008 [154]	2006	Kaiyuan	Yunnan	Southwest	Entertainment	--	LA	23	99	23.2%	4
Du JQ, 2008 [154]	2006	Kaiyuan	Yunnan	Southwest	Entertainment	--	LA	53	101	52.5%	4
Sun JY, 2012 [155]	2006	Jie li	Guizhou	Southwest	Entertainment	--	LA	59	110	53.6%	4
Wu Q, 2011 [156]	2006	Si mao, Qin lin, Da li, Lu feng, Meng zi	Yunnan	Southwest	Entertainment	Cluster sampling	LA	450	1065	42.3%	4
Li DM, 2007 [152]	2006	Guiyang	Guizhou	Southwest	Entertainment	Random sampling	LA	18	143	12.6%	7
Li DM, 2007 [152]	2006	Guiyang	Guizhou	Southwest	Entertainment	Random sampling	LA	51	143	35.7%	7
Wang H, 2009 [153]	2006/03-2006/05	Kaiyuan	Yunnan	Southwest	Entertainment	Convenience sampling	LA	20	279	7.2%	4
Li QH, 2010 [157]	2006/03-2006/04	Kaiyuan	Yunnan	Southwest	Entertainment	--	LA	58	375	15.5%	6
Huang Y, 2006 [158]	2006	Leshan	Sichuan	Southwest	Entertainment	--	LA	20	223	9.0%	4
Huang Y, 2006 [158]	2006	Leshan	Sichuan	Southwest	Entertainment	--	LA	64	223	28.7%	4
Lei JH, 2012 [159]	2006	Kai li	Guizhou	Southwest	--	--	LA	59	101	58.4%	2
Li YY, 2009 [160]	2004/10-2006/12	Gejiu	Yunnan	Southwest	Entertainment	Random sampling	LA	38	181	21.0%	4
Li YY, 2009 [160]	2004/10-2006/12	Gejiu	Yunnan	Southwest	Entertainment	Random sampling	LA	73	181	40.3%	4
Lei JH, 2012 [159]	2006	Kai li	Guizhou	Southwest	--	--	P1M	28	110	25.5%	2
Li DM, 2007 [152]	2006	Guiyang	Guizhou	Southwest	Entertainment	Random sampling	P1M	31	166	18.7%	7
Sun JY, 2012 [155]	2006	Jie li	Guizhou	Southwest	Entertainment	--	P1M	28	110	25.5%	4

First author, published year	Study period	Location	Province	Region	Recruitment venue	Sampling method	Measurement period*	Number of FSW used condom	Total number of FSW	Condom Usage (%)	QA Score
Wu Q, 2011 [156]	2006	Si mao, Qin lin, Da li, Lu feng, Meng zi	Yunnan	Southwest	Entertainment	Cluster sampling	P1M	232	1065	21.8%	4
Li DM, 2007 [152]	2006	Guiyang	Guizhou	Southwest	Entertainment	Random sampling	P1M	18	143	12.6%	7
Huang Y, 2006 [158]	2006	Leshan	Sichuan	Southwest	Entertainment	--	P1M	20	223	9.0%	4
Li YY, 2009 [160]	2004/10-2006/12	Gejiu	Yunnan	Southwest	Entertainment	Random sampling	P1M	38	181	21.0%	4
Peng HB, 2007 [161]	2007/4-2007/5	Nanchong	Sichuan	Southwest	Entertainment	Stratified random sampling	LA	67	419	16.0%	5
Peng HB, 2007 [161]	2007/4-2007/5	Nanchong	Sichuan	Southwest	Entertainment	Stratified random sampling	LA	160	420	38.1%	5
Feng J, 2009 [162]	2007/11	Jiayu	Hubei	Southwest	Entertainment	Random sampling	LA	86	238	36.1%	5
Wu MR, 2004 [163]	2003-2007	Luzhou	Sichuan	Southwest	Entertainment	--	LA	44	248	17.7%	2
Wen TJ, 2009 [164]	2007/4-2007/5	Panzhihua	Sichuan	Southwest	Entertainment	Convenience and snowball sampling	LA	117	358	32.7%	3
Wu CL, 2010 [165]	2007/10-2007/11	Xichang	Sichuan	Southwest	Entertainment	Cluster sampling	LA	67	444	15.1%	5
Wu CL, 2010 [165]	2007/10-2007/11	Xichang	Sichuan	Southwest	Entertainment	Cluster sampling	LA	133	444	30.0%	5
Peng HB, 2007 [161]	2007/4-2007/5	Nanchong	Sichuan	Southwest	Entertainment	Stratified random sampling	P1M	67	419	16.0%	5
Guo L, 2011 [166]	2007	--	Guizhou	Southwest	Entertainment	--	P1M	47	197	23.9%	4

First author, published year	Study period	Location	Province	Region	Recruitment venue	Sampling method	Measurement period*	Number of FSW used condom	Total number of FSW	Condom Usage (%)	QA Score
Wen TJ, 2009 [164]	2007/4-2007/5	Panzhihua	Sichuan	Southwest	Entertainment	Convenience and snowball sampling	P1M	117	358	32.7%	3
Wu CL, 2010 [165]	2007/10-2007/11	Xichang	Sichuan	Southwest	Entertainment	Cluster sampling	P1M	67	444	15.1%	5
Li L, 2009 [167]	2008/12	Kunming	Yunnan	Southwest	Entertainment	--	LA	522	2472	21.1%	4
Li L, 2009 [167]	2008/12	Kunming	Yunnan	Southwest	Entertainment	--	LA	1112	2472	45.0%	4
Zhu Q, 2009 [168]	2008	Chuxiong	Yunnan	Southwest	Entertainment	--	LA	190	394	48.2%	4
Sun JY, 2012 [155]	2008	Jie li	Guizhou	Southwest	Entertainment	--	LA	55	160	34.4%	4
Gan GC, 2010 [169]	2008	Jiangyou	Sichuan	Southwest	Entertainment	Convenience sampling	LA	38	264	14.4%	5
Fu JB, 2010 [170]	2008/09-2008/12	Zhoushan	Zhejiang	Southwest	Entertainment	Convenience sampling	LA	13	100	13.0%	3
Fu JB, 2010 [170]	2008/09-2008/12	Zhoushan	Zhejiang	Southwest	Entertainment	Convenience sampling	LA	10	100	10.0%	3
Dong LM, 2010 [171]	2008/04-2008/06	A district zigong	Sichuan	Southwest	Entertainment	--	LA	57	536	10.6%	6
Dong LM, 2010 [171]	2008/04-2008/06	A district zigong	Sichuan	Southwest	Entertainment	--	LA	144	536	26.9%	6
Lei JH, 2012 [159]	2008	Kai li	Guizhou	Southwest	--		LA	55	160	34.4%	2
Yang ZJ, 2010 [172]	2007-2008	Ruili	Yunnan	Southwest	Entertainment	--	LA	129	303	42.6%	4
Li WZ, 2009 [173]	2008	Jianshui	Yunnan	Southwest	Entertainment	Probability proportional to size sampling	LA	79	183	43.2%	6
Lei JH, 2012 [159]	2008	Kai li	Guizhou	Southwest	--	--	P1M	20	160	12.5%	2
Sun JY, 2012 [155]	2008	Jie li	Guizhou	Southwest	Entertainment	--	P1M	20	160	12.5%	4
Fu JB, 2010 [170]	2008/09-2008/12	Zhoushan	Zhejiang	Southwest	Entertainment	Convenience sampling	P1M	13	100	13.0%	3

First author, published year	Study period	Location	Province	Region	Recruitment venue	Sampling method	Measurement period*	Number of FSW used condom	Total number of FSW	Condom Usage (%)	QA Score
Gan GC, 2010 [169]	2008	Jiangyou	Sichuan	Southwest	Entertainment	Convenience sampling	P1M	38	264	14.4%	5
Li WZ, 2009 [173]	2008	Jianshui	Yunnan	Southwest	Entertainment	Probability proportional to size sampling	P1M	79	183	43.2%	6
Sun JY, 2012 [155]	2009	Jie li	Guizhou	Southwest	Entertainment	--	LA	80	214	37.4%	4
Dong LM, 2010 [171]	2009/04-2009/06	B district zigong	Sichuan	Southwest	Entertainment	--	LA	207	355	58.3%	6
Dong LM, 2010 [171]	2009/04-2009/06	B district zigong	Sichuan	Southwest	Entertainment	--	LA	235	355	66.2%	6
Huang JF, 2010 [174]	2009/08	Qingyuan	Yunnan	Southwest	Entertainment	Random sampling and Convenience sampling	LA	54	259	20.8%	5
Huang JF, 2010 [174]	2009/08	Qingyuan	Yunnan	Southwest	Entertainment	Random sampling and Convenience sampling	LA	110	259	42.5%	5
Dong CL, 2012 [175]	2009/12	Pu er	Yunnan	Southwest	Entertainment		LA	102	201	50.7%	4
Xue HM, 2011 [176]	2009/12	Hekou	Yunnan	Southwest	Entertainment	--	LA	4	200	2.0%	5
Wang H, 2011 [177]	2006/03-2009/11	Kaiyuan	Yunnan	Southwest	Entertainment	Convenience sampling	LA	175	955	18.3%	4
Yang YH, 2012 [178]	2009/01	Pu er	Yunnan	Southwest	Entertainment	Probability proportional to size sampling	LA	103	250	41.2%	2
Dong CL, 2012 [175]	2009/12	Pu er	Yunnan	Southwest	Entertainment	--	P1M	77	201	38.3%	4
Lei JH, 2012 [159]	2009	Kai li	Guizhou	Southwest	--	--	P1M	27	214	12.6%	2
Sun JY, 2012 [155]	2009	Jie li	Guizhou	Southwest	Entertainment	--	P1M	27	214	12.6%	4
Dong LM, 2010 [171]	2009/04-2009/06	B district zigong	Sichuan	Southwest	Entertainment	--	P1M	207	355	58.3%	6

First author, published year	Study period	Location	Province	Region	Recruitment venue	Sampling method	Measurement period*	Number of FSW used condom	Total number of FSW	Condom Usage (%)	QA Score
Sun JY, 2012 [155]	2010	Jie li	Guizhou	Southwest	Entertainment	--	LA	99	242	40.9%	4
Lei JH, 2012 [159]	2010	Kai li	Guizhou	Southwest	--	--	P1M	37	242	15.3%	2
Sun JY, 2012 [155]	2010	Jie li	Guizhou	Southwest	Entertainment	--	P1M	37	242	15.3%	4

*LA: last sex act; P1M: in the past one month prior to the survey

References

1. Yang P, Su X, Yin Y, Wei X, Xia Q, Yu Y, et al. [Investigation of STD intervention modes among female sex workers in China]. Chinese Journal of AIDS & STD. 2005;11(3):195-7.
2. He J, Wu Z, Dou Z. [A survey on HIV awareness and characteristics of sexual behavior among CSWs in public places of entertainments in Wuhu city]. Anhui Journal of Preventive Medicine. 2005;11(5):274-7.
3. Zhu CQ, Wu JH, Fu LJ, Guo TY, Pan NY, Lu QL, et al. [A survey on AIDS knowledge and related risk behavior among female sex workers in public entertainment venues in Shaoxing City, Zhejiang Province]. Chinese Journal of AIDS & STD. 2006;12(2):166,8.
4. Li X, Han H, Zhang X, Li Z. [Analysis on the results from CSWs integrating surveillance in Hefei city in 2004]. Chinese Journal of Disease Control & Prevention. 2005;9(6):580-3.
5. Li X, Zhang B, Liu M, Zhang N, Sun B, Zhang P. [Survey on the behavioral characteristics related to HIV/AIDS and the sero prevalence of sexually transmitted disease (STD) among 466 female sex workers (FSW) in Qingdao]. Chinese Journal of AIDS & STD. 2006;12(1):16-8.
6. Du Y, Qian W, Shao T. [Analysis on results of behavior surveillance and serum examination among female sex workers in entertainment establishments]. Jiangsu Journal of Preventive Medicine. 2006;17(03):12-5.
7. Jin T, Yan J, Ma Q, Pan X, Chen W. [Survey on status Quo of Knowledge about and behaviors of AIDS prevention and control of unlicensed prostitutes in the entertainment establishments in the city of Lishui]. Disease Surveillance. 2005;20(12):621-4.
8. Xiao H, Fu Y, Xiong Z, Xu D, Li J, Tang X. [Evaluation on Effects of Venerism Prevention and AIDS Interference on Female Sex Workers]. Mod Diagn Treat. 2007;18(5):277-81.
9. Guan J, Zheng HQ, Lin CQ, Zhang HH. [The effectiveness of high-risk behavior intervention among sex workers in Fuzhou and Putian City]. Morden Preventive Medicine. 2009;36(9):1727-9.
10. Yan H, Chen G, Cao G, Ding P, Shi P. [Analysis of baseline survey on expanding behavior interventions programme at entertainment establishments in Jiangsu province]. Jiangsu Journal of Preventive Medicine. 2007;18(1):12-5.
11. Xu X, Lan J, Chen X, Lan L, Ban X. [A survey on AIDS- related knowledge and sexual behavior s among female sex workers in Jingning County of Zhejiang Province]. Disease Surveillance. 2007;22(4):240-1,50.
12. Ruan S, Zhang C, Shi Z, Wang C, Pan R, Yang H, et al. [Evaluation on the Effect of Health Education on AIDS Prevention among Commercial Sexual Workers in Public Places of Entertainment in Ji'nan City]. Preventive Medicine Tribune. 2007;13(11):974-5, 80.
13. Zhao XP, Que JL, Liu YC, Mi HH, Zhao YQ, Cao XP, et al. [An investigation of HIV/AIDS prevention and knowledge among female working in entertainment venues in Suzhou City, Jiangsu Province]. Shanghai Journal of Preventive Medicine. 2006;18(11):561-2.
14. Wang J, Liu ZL, Wang B, Yu WX, Wang WB. [Investigation on HIV/STD knowledge, attitude, practice among female sex workers in entertainment venues in Jianhu County, Jiangsu Province]. Jiangsu Journal of Preventive Medicine. 2008;19(3):24-7.
15. Zhu F, Ji K, Li Z, Xu x, Wang R. [Evaluation on the Effect fo AIDS/STD Related Knowledge Training and Behavior Intervention among Female Sex Workers in Entertainment Places]. Preventive Medicine Tribune. 2009;v.15(05):420-2.

16. Xu HQ, Wang JW. [Analysis on HIV/AIDS intervention for commercial sex workers at public venues in Xiuzhou District, Zhejiang Province]. Zhejiang Journal of Preventive Medicine. 2008;20(7):72-3.
17. Qi G, Zhou X, Yan H, Yu R, Hu X. [Baseline survey on STD/ AIDS knowledge, attitude and practice among female sex workers]. Chinese Journal of Public Health. 2007;23(7):861-2.
18. Lu F, Jia Y, Sun X, Wang L, Liu W, Xiao Y, et al. Prevalence of HIV infection and predictors for syphilis infection among female sex workers in southern China. Southeast Asian J Trop Med Public Health. 2009;40(2):263-72.
19. Gu Y, Ding X, Chen X. [Survey on attitude to voluntary HIV test results disclosure among female sex workers in Funan County of Anhui Province, China]. Chinese Journal of Health Education. 2007;23(7):502-4.
20. Zhang XJ, Liao MZ, Kang DM, Tao XR, Qian YS, Wang GR, et al. [Condom Use and Correlates Among Female Sex Workers in Shandong Province,2006-2008]. Preventive Medicine Tribune. 2012;18(6):405-7+10.
21. Yu X. [Survey on the knowledge, attitude, and behavior related to HIV/AIDS among prostitutes in the entertainment establishments in Haimen city]. Journal of Public Health and Preventive Medicine. 2007;18(2):104-5.
22. Wang Y, Li X, Zhang B, Wang L, Liu M, Wu D. [Factors associated with HIV/AIDS STD among young female sex workers in Qingdao]. China J Lepr Skin Dis. 2008;24(06):424-6.
23. Luo Z, Zha YF, Huang ZM. [Analysis on surveillance of secret prostitutes in Songjiang district in year 2006 Shanghai]. Shanghai Journal of Preventive Medicine. 2007;19(4):184-5.
24. Liao M, Bi Z, Liu X, Kang D, Fu J, Song Q, et al. Condom use, intervention service utilization and HIV knowledge among female sex workers in China: results of three consecutive cross-sectional surveys in Shandong Province with historically low HIV prevalence. International journal of STD & AIDS. 2012;23(3):e23-9.
25. Luo Y, Chen S, Xu K, Yuan H, Chen J, Hu J, et al. [Survey of STD/AIDS-related knowledge, behaviors and infection rates of sex workers in entertainment places in Hangzhou]. Disease Surveillance. 2008;23(10):607-9.
26. Liao MZ, Liu XZ, Fu JH, Qian YS, Wang TZ. [Analysis of HIV/ AIDS Surveillance Data in Shandong Province in 2007]. Preventive Medicine Tribune 2008;14(12):1143-5.
27. Chen SP, Tu BY, Wang X. [Survey and Analysis on HIV/AIDS-Related Behavior and Recognition among HIV/AIDS High-risk Population of Xunyang District Jiujiang City]. Chinese Journal of Evidence-Based Medicine. 2010;10(7):817-21.
28. Wang L, Gu B, Yang H. [Investigation on HIV/AIDS knowledge and behaviors among female sex workers in entertainment venues in Yixing City, Jiangsu Province]. Shanghai Journal of Preventive Medicine. 2008;20(10):493-4.
29. Ni YQ, Wang ZY, Shi CL. [Investigation on HIV/AIDS knowledge, attitude and practice (KAP) among female sex workers in three small-sized entertainment venues in Changning District, Shanghai]. Shanghai Journal of Preventive Medicine. 2008;20(11):537-8.
30. Guo Z, Wan D, Feng C, Cai L, Fan Y. [Trend analysis on knowledge and behavior of AIDS prevention among the CSWs in Mengcheng County]. Chinese Journal of Disease Control & Prevention. 2009;13(04):411-3.

31. Cheng X, Xiao Y, Wang H. [Analysis on HIV/AIDS knowledge and condom use of mid-low-end female sex workers]. Anhui Journal of Preventive Medicine. 2008;20(14):410-2.
32. Cui W, Liu J, Yang L. [Changes of AIDS related knowledge and behaviors among FSWs in Lixin County from 2007 to 2008]. Anhui Journal of Preventive Medicine. 2009;30(9):1048-50.
33. Jin Y, Yao Y, Ye D, He J, Dou Z, Qi S, et al. [Investigation and analysis on condom use status female commercial sex workers]. Chinese Journal of Disease Control & Prevention. 2009;13(01):20-2.
34. Wang F, Chen X, Su B, Ji G. [Analysis of the results of the comprehensive HIV/AIDS surveillance among sex workers in Anhui]. Anhui Journal of Preventive Medicine. 2009;15(06):407-8.
35. Yang Y, Yao J, Gao M, Su H, Zhang T, He N. Herpes simplex virus type 2 infection among female sex workers in Shanghai, China. AIDS Care. 2011;23 Suppl 1:37-44.
36. He X. [Evaluation on AIDS comprehensive intervention program among CSWs in Yuyao City]. Zhejiang Journal of Preventive Medicine. 2012;24(11):21-3.
37. Liao M, Nie X, Pan R, Wang C, Ruan S, Zhang C, et al. Consistently low prevalence of syphilis among female sex workers in Jinan, China: findings from two consecutive respondent driven sampling surveys. PLoS One. 2012;7(4):e34085.
38. Cai Y, Shang M, Shen T, Pei B, Jiang X, Huang H, et al. [Awareness and behavioral research of AIDS among female sex workers in small entertainment venues in Shanghai]. Journal of Shanghai Jiaotong University (Medical Science). 2010;30(8):890-3.
39. Yao X, Ying YL, Xu S, Chen ZW, Huang CW, Lu Y, et al. [Evaluation on AIDS high-risk behaviors intervention among female sex workers in entertainment places of Fuzhou]. Modern Preventive Medicine. 2010;37(21):4094-6.
40. Wan LJ, Zhang XX, Gu XM. [A study on HIV/AIDS-related knowledge, attitudes and practices among female sex workers working at different venues]. Zhejiang Journal of Preventive Medicine. 2011;23(5):81-2, 5.
41. Guo HJ, Feng D, Chen ZY, Zhou CX, Sun X, Chen ZM. [AIDS Knowledge Levels and Behavior Characteristics of FSWs in Places of Entertainment in Zunyi City]. Occupation and Health. 2011;27(22):2603-4.
42. Sun L, Wen Y, Zhang MH, Liu XX, Huan XP, Yang HT, et al. [Investigation and analysis of the related knowledge and behavior on AIDS of the FSWs in Jiangsu province]. Acta Universitatis Medicinalis Nanjing (Natural Science). 2012;32(1):10-5.
43. Chen SX, Zhang ML, Han XM. [Survey on AIDS Related Knowledge, Behavior and the HIV Infection Status Among Commercial Sex Workers in Gaomi City]. Preventive Medicine Tribune 2011;17(12):1119-20, 23.
44. Kang DM, Tao XR, Li JZ, Liao WZ, Zhu XH, Zhang H, et al. [Evaluation of AIDS intervention among female sex workers in Global Fund Project counties in Shandong Province]. Journal of Shandong University (Health Sciences). 2011;49(10):155-9.
45. Chen G, Wu ZH, He X, Wang W. [Effect evaluation of the AIDS health education for CSW in 3 cities]. Chinese Journal of Health Education. 2012;v.28(08):682-4+7.
46. Tao SF, Zheng YJ. [Analysis of the characteristics of sexual behavior and syphilis infection among high risk population in Zongyang county, Anhui province]. Anhui Journal of Preventive Medicine. 2012;18(3):195-7.

47. Sun BJ. [Survey on AIDS-related Knowledge, Behavior and HIV-infection Among Commercial Sex Workers in Shizhong District, Zaozhuang City, 2011]. Preventive Medicine Tribune. 2012;18(9):654-6.
48. Song ZP. [Evaluation on the effect of HIV/AIDS prevention intervention programs among bargirls in Yingze District of Taiyuan City, Shanxi] Chinese Journal of Public Health Management. 2004;20(5):457-9.
49. Ren X, Bo F, Bao Z, Xu R, Zhou B, Liu X, et al. [A study of HIV and syphilis infection and related behaviours among female sex workers in Hohhot, China]. Chinese Journal of AIDS & STD. 2006;12(06):551-2.
50. Li G, Xia D, Lu H, Yang Y, Gao J, Zhang M, et al. [Study on AIDS related risk factors and behaviors among female commercial sex workers]. China Preventive Medicine. 2008;9(1):1-4.
51. Lin Z, Liu GY, Li ZM, Zhao RL, Zhang XM, Chen Y, et al. [Analysis of HIV surveillance among 364 female sex workers in Tongliao City, Inner Mongolia]. Chinese Journal of Public Health. 2007;23(3):355.
52. Liu Y, Ding H, Yu S. [The effects of sexually transmitted infections(STI)/AIDS behavioral intervention among female sex workers]. Chin J Prev Med. 2007;41(06):492-5.
53. Bai JM, Shi WY, Xie HY, Qu YM, Zhai CX, Tian ML, et al. [Prevalence of high-risk behavior and HIV infection among bargirls in Fengtai District of Beijing]. The Chinese Journal of Human Sexuality 2007;16(6):45-6, 8.
54. Shi Y, Guo S, Bo F, Zhang X, Cao W, Wang P. Impact evaluation of a sexually transmitted disease preventive intervention among female sex workers in Hohhot, China. Int J Infect Dis. 2013;17(1):e59-64.
55. AO X, Han Q. [Survey of AIDS-related knowledge, behaviors and infection HIV and syphilis among 105 sex workers]. Disease Surveillance. 2008;23(11):714-6.
56. Liu L, Liu M, Lu H, Xia D. [Analysis of HIV/AIDS related risk behaviors among female sex workers at entertainment establishments in two districts of Beijing]. Chinese Journal of AIDS & STD. 2007;13(6):532-5.
57. Bao F, Gao P, Yun Z, Ren X, Liu X, Guo S. [An AIDS epidemiological survey of female sex workers crowd in Hohhot]. Inner Mongolia Medical Journal. 2007;39(08):971-3.
58. Liu Y, Zhang M, Zhang W. [STD/AIDS-related risk behaviours and condom usage among 202 female sex workers]. Chinese Journal of AIDS & STD. 2008;14(5):516-8.
59. Cao H. [Analysis of high risk AIDS-related behavior characteristics among 186 female sex workers at Xiqing district of Tianjin]. Port Health Control. 2010;15(5):27-9.
60. Shao B, Yao SP, Wang KL, Yang JQ, Cao B, Wang J, et al. [The survey of AIDS knowledge behaviors and condom use among female sex workers in Heilongjiang Province]. Chinese Journal of Disease Control & Prevention. 2011;v.15(04):318-22.
61. Lin B, Shi F, Guo J, Guo XM, Meng W, Wang F, et al. [Investigation and evaluation of Intervention effect for STD infection in women working in public places for entertainment]. Practical Preventive Medicine. 2002;9(5):440-2.
62. Zhang M, Rui BL, Xue Q, Zhai SH, Wang L. [Study on HIV and Syphilis infection among female sex workers in Urumqi of Xinjiang]. China Preventive Medicine. 2006;7(03):216-7.

63. Song Y, Wang Q. [A study of HIV infection status among entertainment-based female sex workers in Alar City, Xinjiang, 2004]. Endemic Diseases Bulletin. 2005;20(02):45-6.
64. Lin B, Ceng KF, Meng W, Guo J, A Si MGL, Hu J. [Analysis of AIDS sentinel surveillance among female sex workers in Karamay from 2004 to 2008]. Chinese Journal of Public Health. 2009;25(1):75-6.
65. Liu Y, Yang X. [HIV knowledge, behavioural characteristics and condom usage among 415 female sex workers]. Modern Preventive Medicine. 2007;34(21):4144-5.
66. Zhang L, Li Y, Chen JJ. [Analysis on HIV knowledge and awareness among female sex workers in entertainment venues in Lanzhou City of Gansu Province]. Health Vovational Education. 2007;25(20):95-6.
67. Zeng K, Lin B, Wang F, Meng Y, Guo J. [Investigation on Risk Behavior of Sexual Transmitted Diseases/ADIS among Commercial Workers in Entertainment Places in Karamay City]. Preventive Medicine Tribune. 2008;14(02):124-6.
68. Lin B, Luo M, Wang S, Asimu G, Zeng K, Guo J, et al. [Effectiveness evaluation of AIDS related integrated interventions among female sex workers in entertainment settings in Karamay city]. Chinese Journal of AIDS & STD. 2009;v.15;No.82(01):41-3.
69. MAYT A. [HIV Cross-section Study on Female Sex Workers in Xinshi District, Urumqi in 2007]. Endemic Diseases Bulletin. 2008;23(4):27-8.
70. Liu S, Jiang W, Ma S, Li F. [A study of HIV-related knowledge and high-risk behaviours among community female sex workers in Huinong district, Ningxia, China]. Ningxia Med J. 2009;31(9):833-4.
71. Zhang X, Luo X. [Effect Analysis on AIDS Know ledge and Behavior Intervened among Sex Workers in Gaolan County of Gansu Province]. J Diagn Ther Derma Venereo. 2010;v.17(03):238-41.
72. Gao W, Li Z, Yan H, Wang D, Li Y, Dang S, et al. Preventive measures against sexually transmitted infections among female sex workers in Lanzhou, China. Scand J Infect Dis. 2012;44(5):374-80.
73. Wu Y, Luo GY, Wan SP, Zhou H. [Analysis the trends of the prevalence of AIDS and syphilis among female sex workers in Hongya county from 2008 to 2010]. Journal of North Sichuan Medical College. 2012;27(5):441-4.
74. Tao LD, Qi YJ, Wei HW, Chen JJ. [Epidemiology of HIV and Syphilis Among Female Sex Workers in Chenguan County, Gansu Province]. Chinese Primary Health Care. 2011;25(9):65-6.
75. Liu DF, Chen HX. [2011 Epidemiology of HIV among Female Sex Workers in Yanchuan County, Shaanxi Province] Henan Journal of preventive Medicine. 2012;23(5):398+400.
76. Qu S, Liu W, Choi K-H, Li R, Jiang D, Zhou Y, et al. The Potential for Rapid Sexual Transmission of HIV in China: Sexually Transmitted Diseases and Condom Failure Highly Prevalent Among Female Sex Workers. AIDS Behav. 2002;6(3):267-75.
77. Zhu GR, Sun JP, Peng Z, Liu W, Song Y. [Investigation of condom use among commercial sex workers]. Chinese Journal of AIDS & STD. 2003;9(2):95-7.
78. Wei M, Li J, Xu D, Zhang F, Zeng X. [Epidemic survey among female sex workers and drug users in Hainan province in 2003]. Hai Nan Medical Journal. 2004;15(11):107-8.
79. Li N, Wang Z. [The survey of HIV prevalence among commercial sex workers in Henan Province]. Henan J Prev Med. 2005;16(04):213-4.

80. Yang BF, Xu J, Yao ZZ, Chen ZD, Wang X, Liu MQ, et al. [An analysis of the behavior characteristics and the infection of HIV and syphilis among the commercial female sex workers]. Chinese Journal of Disease Control & Prevention. 2006;2006(4):406-8.
81. Yang B, Xu J, Wang X, Yao Z, Liu M, Tang L, et al. [Investigation on high risk behavior and chlamydia trachomatis infection in prostitutes]. J of Pub Health and Prev Med. 2006;(03):31-3.
82. Li Q, Li X, Stanton B, Wang B. Psychometric properties of a pictorial scale measuring correct condom use. AIDS Behav. 2011;15(2):432-40.
83. Yang BF, Ye LX, Yao ZZ, Chen ZD, Xu J, Wang X, et al. [Study on HIV/AIDS knowledge among commercial female sex workers in Wuhan City, Hubei Province]. Central China Medical Journal. 2006;30(5):419-7.
84. Yang F, Lin P, He Q, Xu RH, Yu DN, Mo KS, et al. [A Survey on AIDS-related KABP among 5 target groups in 3 China comprehensive AIDS responses in Guangdong]. South China Journal of Preventive Medicine. 2006;32(1):10-3.
85. Jiang M, Wu JH, Huang JG, Huang GY, Zhu BY, Sun C, et al. [STD/AIDS knowledge, attitude, behaviour and infection status among prostitutes in entertainment sites]. Theory and practice of Chinese Medicine. 2005;15(1):152-3.
86. Yang H, Li X, Stanton B, Fang X, Zhao R, Dong B, et al. Condom use among female sex workers in China: role of gatekeepers. Sex Transm Dis. 2005;32(9):572-80.
87. Luo J. [Analysis of the results from HIV Surveillance of unlicensed prostitutes]. Disease Surveillance. 2005;20(8):409-12.
88. Zhou YJ, Liu W, Guo WG, Zhu QY, Li RJ, Zhou L, et al. [Result of behavioral surveillance of female sex workers in a coastal city in Guangxi, China.]. Journal of Applied Preventive Medicine. 2006;12(4):239-40.
89. Xu YF, Mo XJ, Liang HH, Zhou FH, Li P, Zhou J, et al. [Investigation on STD/AIDS knowledge and risk behaviors among commercial sex women in Nanning City]. Modern Preventive Medicine. 2007;34(21):4007-8, 11.
90. Xu X, Yang Y, Xu H. [Correlation of AIDS and Prostitutes in High-Class Entertainment Centers]. Journal of Tropical Medicine. 2007;7(6):610-1.
91. Zhou YJ. [Analysis of risk behaviors of 105 female sexual workers positive for Treponema pallidum]. China Tropical Medicine. 2008;8(10):1832, 47.
92. Wen X. [Survey on KAB and serology of AIDS among 360 female commercial sex workers in Guilin]. Modern Preventive Medicine. 2009;36(14):2687-9.
93. Tan W, Zhou H, Liu H, Mo X, Luan W. [Comprehensive HIV surveillance results analysis for commercial sex workers in Nanning City, 2007]. Guangxi Medical Journal. 2008;30(11):1727-8.
94. Lu WJ, Liu W, Zhu QY, Lan GH, Li F. [Analysis of the results of comprehensive surveillance in AIDS-related high risk groups in 20 cities/counties of Guangxi in 2007]. Chinese Journal of AIDS & STD. 2008;14(6):583-6.
95. Li Y, Lin P, Detels R, Fu X, Deng Z, Liu Y, et al. [Prevalence of HIV infection and sexually transmitted disease and associated risk factors among female sex workers in Guangdong province]. Disease Surveillance. 2009;24(08):599-602.
96. He Y, Xu Y, Liang F, Zhou F, Mo X. [Results of intervention of AIDS health education and behavior in sex workers in entertainment places in Nanning City]. China Tropical Medicine. 2009;9(02):333-4.
97. Zhang YX, Lin HT, Feng WD, Shan GS, Zhang TJ. [Syphilis and HIV infection status among commercial sexual workers in Liuzhou, Guangxi]. Journal of Tropical Medicine. 2011;11(3):337-9+55.

98. Jiang M, Wu JH, Huang JQ, Huang GY, Zhu BY, Sun C, et al. [Survey of STI/AIDS-related knowledge, attitude and behaviors and infection rates of sex workers in entertainment places in Jingzhou City]. Chinese Journal of Disease Control & Prevention 2012;16(2):175-7.
99. Zhou J, Huang Z, Deng B, Chen Y, Luo R. [Survey on the behavioral characteristics and sexually transmitted infection among 418 female commercial sex workers]. Modern Preventive Medicine. 2010;37(6):1158-9, 61.
100. Chen Y, Liu W, Ted H, Meng D, Xiang S. [Survey of sex workers along Guangxi border region about their HIV related knowledge, behavior and infections]. Chinese Journal of Disease Control & Prevention. 2010;14(7):619-22.
101. Zhao Y, Li M, Li Y, Liang Z, Zhang X, Guo Z. [The status of sexually transmitted infections in the female sex workers at the entertainment venues in Panyu district. International Medicine & Health Guidance News. 2010;16(18):2212-4.
102. Tan J, Chen L, Cai W, Yang Z, Shi X, Wang X. [Study on AIDS/STD related risk behaviors among female sex workers in Shenzhen]. Chinese journal of Social Medicine. 2009;v.26(04):242-4.
103. Huang XT, Lin ZW, Mao XT, Chen SN. [Investigation on HIV/AIDS related risk behaviors among female sex workers in entertainment venues in Chaoyang District of Guangdong Province]. South China Journal of Preventive Medicine. 2009;35(1):33-4.
104. Zhang L, Xue FH, Zhang XQ, Dong SB. [Study on HIV/AIDS related knowledge and behavior among female sex workers in Xincai County of Henan Province]. Henan Journal of Preventive Medicine. 2010;21(3):235-7.
105. Bai Y, Weng YQ, Feng WD. [Sentinel Surveillance of AIDS in Liuzhou, 2008]. Journal of Preventive Medicine Information. 2010;26(7):527-30.
106. Wang J, Wang T, Cen Y, Lai X, Li L, Chen C, et al. [Prevalence of sex transmitted disease or its related symptoms and associated risk factors among female sex workers in Zhongshan]. Journal of Tropical Medicine. 2010;10(4):477-80.
107. Ceng XL, Li P, Ma CH. [The effects of sexually transmitted infections AIDS behavioral intervention among female sex workers in project region]. Henan Journal of preventive Medicine. 2011;22(2):92-4.
108. Wang J, Wang T, Cen Y, Lai X, Li L, Chen C, et al. [Influencing factors of condom use and HIV infection in female sex workers in Zhongshan City]. South China Journal of Preventive Medicine. 2010;36(4):26-8, 34.
109. Hu S, Huang J, Li C, Huang J, Xuan R. [Survey on AIDS related knowledge and behaviors among female sex workers in entertainment places in Qingcheng district of Qingyuan City]. Occupation and Health. 2010;26(19):2218-30.
110. Dun ZJ, Ling L, Xia HY, Wang C, Lin AH, Lu CY. [Survey on HIV/AIDS related KABP of four high-risk population in Guangzhou City of Guangdong Province]. Chinese Journal of Health Education. 2011;v.27(11):843-6.
111. Liao S, Weeks MR, Wang Y, Nie L, Li F, Zhou Y, et al. Inclusion of the female condom in a male condom-only intervention in the sex industry in China: a cross-sectional analysis of pre- and post-intervention surveys in three study sites. Public health. 2011;125(5):283-92.
112. Xiong CS, Mao LF, Peng J, Zeng Y, Ren CM. [Analysis of HIV/AIDS Knowledge and related Behavior within 400 Female Sex Workers]. Journal of Public Health and Preventive Medicine. 2012;23(3):86-7.
113. Jiang N. [Surveillance of risk behaviors facilitating among commercial sex works and analysis of HIV, Syphilis, HCV and HBV infection]. Journal of Medical Forum. 2012;33(4):81-2.

114. He B, Xuan DQ, Zeng QM, Lin YS, Wu CD, Li RY, et al. [Investigation on Characteristics of Sexual Behaviours and Acceptance towards Female Condom among Female Sex Workers]. Chinese Journal of Public Health. 2012;28(10):1383-4.
115. Zhu L, Qiu XS, Xie AQ, Gong WS, Wen MX, Yang XZ. [Analysis of AIDS Sentinel Surveillance among prostitutes in Xiangyang in 2010]. Journal of Public Health and Preventive Medicine. 2011;22(3):92-3.
116. Yang HW, Zhu XY, Sun HY, Yang XL, Feng Y, Tang G, et al. [Investigation on sexual behaviour, health-seeking behaviour and gynecological examination among female sex workers in a city]. Journal of Preventive Medicine Information. 2003;19(z1):42-3.
117. Huang ZM, Yang LG, Lan F, Zhang YP, Fang HY, Zhang XQ, et al. [An analysis of the effectiveness on HIV/STD comprehensive intervention for female sex workers in Luxi County, Yunnan Province]. Soft Science of Health. 2006;20(3):254-7.
118. Li J, He S, Xie G, Li Y, Li L, Du Z, et al. [Effectiveness evaluation of comprehensive HIV interventions among female sex workers in Ershan county, China]. Soft Science of Health. 2006;20(03):251-3.
119. Huang LH, Liu YZ, Chen ZJ, Lu MJ, Xu XR, Zhang XZ, et al. [Comprehensive HIV/AIDS intervention for female sex workers - effectively curb the spread of STD and AIDS]. Soft Science of Health. 2006;20(3):271-3.
120. Wang L. [Analysis of female sex workers behavioural surveillance data in Leshan prefecture in 2003]. Journal of Preventive Medicine Information. 2004;20(3):297-9.
121. Jin Y, Yin GG, Bao WS, Liu CB, Mao BB, Deng GY, et al. [Study on HIV/AIDS behavior intervention in the waitresses of inns around roads]. Soft Science of Health. 2006;20(1):55-8.
122. Zhang YL, Li K, Ma Y, Li JL, Zhang C, Li FR, et al. [The Evaluation of the Condom Social Marketing Project amomg female sex workers - comprehensive STD/AIDS Intervention Project on high risk populations in Hongta District, Yunnan Province]. Soft Science of Health. 2006;20(3):228-31.
123. Li S. [A Survey on 2003 Behavior Monitoring of 364 CSW in Bazhong City]. JPrev Medical Information. 2004:494-5.
124. Lau JT, Choi KC, Tsui HY, Zhang L, Zhang J, Lan Y, et al. Changes in HIV-related behaviours over time and associations with rates of HIV-related services coverage among female sex workers in Sichuan, China. Sex Transm Infect. 2011;84(3):212-6.
125. Lau JT, Wang R, Chen H, Gu J, Zhang J, Cheng F, et al. Evaluation of the overall program effectiveness of HIV-related intervention programs in a community in Sichuan, China. Sex Transm Dis. 2007;34(9):653-62.
126. Kan HY, Du CH, Long P, He R, Wen RB, Li SY. [Analysis on HIV/AIDS comprehensive intervention among female sex workers in entertainment venues in Yuanjiang County, Yunnan Province]. Soft Science of Health. 2006;20(3):262-3.
127. Lai W, Zhou D, Zhang L, Zeng Y, Huang T. [Risk behaviors of AIDS among female CSW and also IDU]. Parasitoses and Infectious Diseases. 2009;7(2):80-3.
128. Su DT, Zhang YR, Liang J, Ouyang Y, Mu SH, Luan RS. [Survey of female sexual workers in top grade entertainment place of Chengdu donwtown district]. Modern Preventive Medicine. 2005;32(1):63-5.
129. Zhao R, Wang B, Fang X, Li X, Stanton B. Condom use and self-efficacy among female sex workers with steady partners in China. AIDS Care. 2008;20(7):782-90.
130. Lau JT, Zhang J, Zhang L, Wang N, Cheng F, Zhang Y, et al. Comparing prevalence of condom use among 15,379 female sex workers injecting or not injecting drugs in China. Sex Transm Dis. 2007;34(11):908-16.

131. Zou YD, He CY, Yang JH. [Analysis on HIV/AIDS behavioural intervention project for female sex workers in Chuxiong City, Yunnan Province]. Soft Science of Health. 2006;20(3):245-8.
132. Wen Y, Zhang Q, Ren XQ, Fu YF, Yang B, Fang RP, et al. [A study of the role of maternal and child health organisations in HIV/STD prevention among female sex workers]. Soft Science of Health. 2006;20(03):287-90.
133. Tan X, Yi H, Wang Z, Yu X, Zheng Y, Tang C, et al. [An analysis of knowledge, attitude and the feature of high risk behavior on AIDS among 266 unlicensed prostitutes]. Modern Preventive Medicine. 2007;34(02):253-5+62.
134. Zeng K. [Behavioral Investigation of AIDS on Unlicensed Prostitute in Aba Canton of Sichuan Province from 2002 to 2006]. Journal of Preventive Medicine Information. 2007;23(6):720-1.
135. Ruan Y, Cao X, Qian HZ, Zhang L, Qin G, Jiang Z, et al. Syphilis among female sex workers in southwestern China: potential for HIV transmission. Sex Transm Dis. 2006;33(12):719-23.
136. Ding X, Yi H, Jiang X, Han L, Wu G, Ling H, et al. [Analysis of status of AIDS related knowledge, attitude and risk behavior among 519 female sex workers in Chongqing]. Chinese Journal of AIDS & STD. 2006;12(04):347-9.
137. Zhang Q, Jiang X, Jiang B, Wang X, Peng H, Long D, et al. [Analysis on High-risk Behaviors Related to AIDS of CSW in Nanchong City]. Journal of Preventive Medicine Information. 2006;22(1):28-32.
138. Yuzhen LC, Ya X, Ma YG, Gama ZM, Duoji WM, Suolang DJ, et al. [Investigation on HIV infection and related behaviour among female working at entertainment venues in Lhasa City of Tibet Autonomous Region]. Chinese Journal of AIDS & STD. 2006;12(6):545, 2.
139. Yuan MJ, Bai W, Dong H, Cheng CQ. [Report on HIV/AIDS surveillance among female sex workers in Deyang City of Sichuan Province in 2005]. China Journal of Clinical Medicine Hygiene. 2006;4(9):96-8.
140. Wang J, Tao R, Hu H, Shi Q, Shan X. [Effectiveness analysis of comprehensive STD/AIDS interventions among female sex workers in Huaning county, Yunnan]. Soft Science of Health. 2006;20(3):293-5.
141. Chen CL, Li XS, Zhou GH, Li QF, Zhang Q, Chen JL, et al. [Evaluation of intervention amongst roadside female sex workers in Malong County of Yunnan Province]. Soft Science of Health. 2006;20(3):238-41.
142. Luo G, Feng Z, Wang J. [Analysis of high-risk behaviors among CSWs in urban area of Meishan]. Journal of North Sichuan Medical College. 2006;21(3):223-6.
143. Jiang Z, Cao X, Ruan Y, Hao Q, Song B, Hu W, et al. [Factors associated with unprotected sex behavior among female sex workers in drug heavy using area]. Chinese Journal of Disease Control & Prevention. 2006;10(05):458-61.
144. Wang Y, Zhang GG, Yang HW, Sun HY, Feng Y, Tang G, et al. [Analysis of behaviors surveillance of 1175 female sexual workers in Mianyang City]. Modern Preventive Medicine. 2007;34(24):4611-5.
145. Chen X, Cao X, Ruan Y, Zhang W, Duan Y, Jiang Z, et al. [Factors Associated with Drug Use among Female Sex Workers in Xichang of Sichuan Province]. Chinese Journal of Drug Abuse Prevention and Treatment. 2006;12(1):1-4.
146. Wang Y, He H, Chen C. [Behavioural surveillance report of female sex workers in Shangri-La, Yunnan]. World Health Digest Medical Periodical. 2009;6(10):247-8.

147. Wang C, Yang H, Yang Y, Dong C, Zhang Y, He Z, et al. [Evaluation of promote the use of condoms 100% in entertainment places of Simao]. Chinese Medical Science & Health. 2007;(7):1-4.
148. Tian L, Ma Z, Ruan Y, Cao X, Huang J, Wang D, et al. [Incidence rates of human immunodeficiency virus and syphilis as well as the rate of retention in a 6-month follow-up study of female sex workers in areas with heavy drug use in Xichang of Sichuan province, China]. Chin J Epidemiol. 2006;27(11):939-42.
149. Cao X, Jiang Z, Ruan Y, Liang S, Qin G, Yang Y, et al. [Analysis on the factors of unprotected sex behavior among female sex workers]. Chinese Journal of Health Education. 2007;23(4):277-9.
150. Cao X, Jiang Z, Ruan Y, Liang S, Song B, Hu W, et al. [Investigation on initiation into commercial sex among female sex workers in Xichang city]. Chinese Journal of Public Health. 2006;22(5):516-7.
151. Li L, Li ZM, Zhao SP, Liu J, Pu ZL. [An evaluation and assessment report of baseline investigation on HIV/AIDS high risk population intervention in Kunming, 2005]. Journal of Dermatology and Venereology. 2007;29(1):1-3.
152. Li D, Yuan F, Hu S, Lv F. [High risk behaviors and HIV/STI prevalence among female sex workers in different settings]. Chinese Journal of AIDS & STD. 2007;13(03):210-3.
153. Wang H, Chen RY, Ding G, Ma Y, Ma J, Jiao JH, et al. Prevalence and predictors of HIV infection among female sex workers in Kaiyuan City, Yunnan Province, China. Int J Infect Dis. 2009;13(2):162-9.
154. Du J, Wang G, Wang W, Gu J, Chang D, Zhao M, et al. [Analysis on the survey of HIV/AIDS/STI among the commercial sex workers in Kaiyuan city]. Soft Science of Health. 2008;22(1):84-6.
155. Sun JY, Xiao YL, Huang GX, Lei JH. [Monitoring on AIDS behavior among prostitutes in Kaili from 2006 to 2010]. Modern Preventive Medicine. 2012;39(14):3593-8+600.
156. Wu Q, He CY, Duo L, He LM, Chen Y, Wang YY. [HIV/AIDS-related Risk Behaviour in Female Sex Workers, Southwest China]. Science & Technology Information. 2011;(13):9-10.
157. Li Q, Xu J, Wang H, Wang G, Zhang W, Wang N. [Prospective epidemiological study of factors correlated with seeking HIV post testing counseling service among female sex workers in Kaiyuan city]. Chinese Journal of AIDS & STD. 2010;16(03):295-7.
158. Huang Y, Zhou XW, Fan L, Guo ZH, Qiu XM. [A survey on HIV/AIDS knowledge and sexual behaviour among commercial female sex workers in Leshan City, Sichuan Province]. Journal of Preventive Medicine Information. 2006;22(1):73-4.
159. Lei JH, Xiao YL, Sun JY. [Analysis of the changing trend of AIDS high-risk behaviors among CSWs in Kaili city]. Chinese Journal of AIDS & STD. 2012;18(2):124-6.
160. Li Y, Wen Y, Hu Z, Chen W, He L, Yang F. [Integrated AIDS Behavioral Intervention Among Female Sex Workers in Gejiu City]. Journal of Kunming Medical University. 2009;v.30(09):113-7+21.
161. Peng H, Fu G, Feng Y, Tian X, Feng J, Zhang Q, et al. [A Survey on CSW's AIDS knowledge, behavior and intervention in an urban district in Nanchong]. Journal of North Sichuan Medical College. 2007;22(05):428-31.
162. Feng J, Hu X, Ji F, Pan H. [Psychological factor analysis of low comply degree and of female sex serving in vulgar entertainment place in AIDS behavioral intervention]. Chin J Bebav Med & Brain Sci. 2009;18(2):157-8.

163. Wu Q, Liu Q. [An analysis of sexual behaviours among female sex workers in urban Luzhou City]. Journal of Preventive Medicine Information. 2004;20(5):555-7.
164. Wen TJ, He TT, Liu Y. Survey on HIV/AIDS-related knowledge and behaviors of sex workers, drug addicts in Renhe district of Panzhihua. Modern Preventive Medicine. 2009;36(15):2907-9.
165. Wu C, Zhang J, Fan S, Wei D, Li C, Chen K, et al. [A study on HIV/SYPHILIS prevalence and related knowledge and behaviors and their influence factors among female commercial sex workers in a city]. Modern Preventive Medicine. 2010;37(03):499-501.
166. Guo L, Wang YL, Li CM. [Investigation on HIV-related knowledge and praxiology among illicit prostitutes in Guizhou Province]. Chinese Journal of Public Health. 2011;27(7):891-2.
167. Li L, Chen L, Yang Y, Che ZM, Chen YL, Chu CX. [Population Scale Estimate, HIV/AIDS Knowledge Awareness Rate and Behavior Risk Factors Survey of Female Commercial Sex Workers (FCSWS) in Kunming 2008]. Chinese Journal of AIDS & STD. 2009;31(2):1-3.
168. Zhu Q, Wang L, He CY, Zhang XB, Yao XZ. [Analysis on behavioural surveillance among 762 female sex workers]. Journal of Dematology and Venereology. 2009;31(2):45-6.
169. Gan CG, Zhang TS. [Analysis on HIV/AIDS-related knowledge and behaviors among female commercial sex workers in Jiangyou City]. Journal of Southwest University for Nationality (Natural Science Edition). 2010;36(5):866-9.
170. Fu JB, Liu LJ. [Survey on AIDS related knowledge and behaviour amongst 100 roadside female sex workers]. Zhejiang Journal of Preventive Medicine. 2010;22(5):79-80.
171. Dong L, Li Q, Chen X, Zhou M, Xie Y. [Surveillance of behavior among female sex workers in Zigong, 2008-2009]. Journal of Preventive Medicine Information. 2010;26(12):982-5.
172. Yang ZJ, Yin ZL, Li ZL, Liu B, Fang KF, Chu XQ. [Investigation on HIV/AIDS epidemic among female sex workers in entertainment establishments in Ruili City, Yunnan Province]. Soft Science of Health. 2010;24(4):373-5.
173. Li W. [HIV sentinel surveillance and analysis among female sex workers in Jianshui county, China]. Soft Science of Health. 2009;23(06):711-3.
174. Huang J, Li C, Hu S, Xuan R, Huang J, Sun X, et al. [Analysis of Intervention Effect of AIDS High Risk Behavior among Female Sexual Workers in The Entertainment Places in Qingyuan City]. Journal of Tropical Medicine. 2010;10(9):1122-6.
175. Dong CL, Hua YJ, Yang YH, Li HL, Zhang Y, He Z. [Study on the effect of condom use promotion among FSWs from Entertainment venues]. Journal of Dermatology and Venereology. 2012;34(5):301-2.
176. Xue HM, Duo L, Zhu ZB, Yang LH, Deng L, Lin B. [Analysis on HIV infection and related risk factors among China-Vietnam cross border prostitutes]. Soft Science of Health. 2011;25(2):107-8.
177. Wang H, Brown KS, Wang G, Ding G, Zang C, Wang J, et al. Knowledge of HIV seropositivity is a predictor for initiation of illicit drug use: Incidence of drug use initiation among female sex workers in a high HIV-prevalence area of China. Drug and Alcohol Dependence. 2011;117(2-3):226-32.
178. Yang YH, Wang C, Li HL, Dong CL, Zhang Y, Xie B, et al. [Study on the method and effect of 100% condom use promotion in entertainment venue in Pu'er City]. Soft Science of Health. 2012;26(8):727-9.
