# Supplementary material for: Risk Behaviours among Female Sex Workers in China: A Systematic Review and Data Synthesis
Source: PLoS One. 2015 Mar 27;10(3):e0120595. doi: 10.1371/journal.pone.0120595 (PMC4376708; doi:10.1371/journal.pone.0120595)
Supplement: S2 Table — (PDF) [file pone.0120595.s004.pdf]

Table S2. Studies reported the rate of condom use in female sex workers with male non-commercial casual partners.

First author, published year	Study period	Location	Province	Region	Recruitment venue	Sampling method	Measurement period*	Number of FSW used condom	Total number of FSW	Condom Usage (%)	QA score
Du YP, 2004 [1]	2001/11	Jingjiang	Jiangsu	East	Entertainment	--	LA	97	355	27.3%	4
Yang P, 2005 [2]	2002/12	Fuzhou	Fujian	East	Entertainment	Random sampling	LA	67	149	45.0%	3
Wei XM, 2004 [3]	2001/12-2002/03	Quanzhou	Fujian	East	Entertainment	--	P1M	78	657	11.9%	2
Wei XM, 2004 [3]	2001/12-2002/03	Quanzhou	Fujian	East	Entertainment	--	P1M	144	384	37.5%	2
Guan JH, 2009 [4]	2004/08	Fuzhou, Putian	Fujian	East	Entertainment	Random sampling	P1M	139	428	32.5%	5
Yang Y, 2011 [5]	2008/06-2008/10	Shanghai	Shanghai	East	Entertainment	--	LA	64	109	58.7%	5
Liao M, 2012 [6]	2008/02-2008/08	Jinan	Shandong	East	Entertainment	Respondent-Driven Sampling	P1M	38	113	33.6%	6
Yang Y, 2011 [5]	2009/05-2009/08	Shanghai	Shanghai	East	Entertainment	--	LA	42	71	59.2%	5
Liao M, 2012 [6]	2009/05-2009/10	Jinan	Shandong	East	Entertainment	Respondent-Driven Sampling	P1M	74	229	32.3%	6
Wang Y, 2011 [7]	2009/05-2009/08	Beijing	Beijing	North	Entertainment	--	LA	163	201	81.1%	4
Wang Y, 2011 [7]	2009/05-2009/08	Beijing	Beijing	North	Entertainment	--	P1M	126	201	62.7%	4
Liu J, 2011 [8]	2005	Heng yang	Hunan	South Central	Sentinel sites	--	P1M	46	400	11.5%	1
Xu YF, 2007 [9]	2005/07-2005/09	Nanning	Guangxi	South Central	Entertainment	Random sampling	P1M	40	109	36.7%	7
Li Y, 2009 [10]	2006/08-2007/01	Guangdong	Guangdong	South Central	Entertainment	Respondent-Driven Sampling	LA	227	320	70.9%	6

First author, published year	Study period	Location	Province	Region	Recruitment venue	Sampling method	Measurement period*	Number of FSW used condom	Total number of FSW	Condom Usage (%)	QA score
Yu DY, 2012 [11]	2008/07	Liu zhou	Guangxi	South Central	Entertainment	--	P1M	36	213	16.9%	3
Wang JY, 2010 [12]	2009/05	Zhongshan	Guangdong	South Central	Community	Convenience sampling	LA	101	130	77.7%	4
Hu SX, 2010 [13]	2009/10-2009/12	Qingyuan	Guangdong	South Central	Entertainment	Random sampling	LA	115	147	78.2%	4
Liu J, 2011 [8]	2009	Heng yang	Hunan	South Central	Sentinel sites	--	P1M	182	380	47.9%	1
Lu PN, 2008 [14]	04/2007-05/2007	Panzhihua	Sichuan	Southwest	Entertainment	Convenience sampling	LA	41	241	17.0%	3
Lu PN, 2008 [14]	04/2007-05/2007	Panzhihua	Sichuan	Southwest	Entertainment	Convenience sampling	P1M	11	241	4.6%	3
Huang JF, 2010 [15]	2009/08	Qingyuan	Yunnan	Southwest	Entertainment	Random sampling, convenience sampling	LA	81	110	73.6%	5

*LA: last sex act; P1M: in the past one month prior to the survey

References

1. Du Y, Yang H, Jian P, Qian W. [Study on the effectiveness and sustainability of implementing 100% condom use programme in entertainment establishments]. Jiangsu Journal of Preventive Medicine. 2004;15(3):31-3.
2. Yang P, Su X, Yin Y, Wei X, Xia Q, Yu Y, et al. [Investigation of STD intervention modes among female sex workers in China]. Chinese Journal of AIDS & STD. 2005;11(3):195-7.
3. Wei XM, Zhao JY, Xu YY, Hong YJ, Huang XR. [Knowledge, attitude, and practices (KAP) study on HIV/AIDS among female attendants in entertainment places]. Strait Journal of Preventive Medicine. 2004;10(2):57-8.
4. Guan J, Zheng HQ, Lin CQ, Zhang HH. [The effectiveness of high-risk behavior intervention among sex workers in Fuzhou and Putian City]. Morden Preventive Medicine. 2009;36(9):1727-9.
5. Yang Y, Yao J, Gao M, Su H, Zhang T, He N. Herpes simplex virus type 2 infection among female sex workers in Shanghai, China. AIDS Care. 2011;23 Suppl 1:37-44.
6. Liao M, Nie X, Pan R, Wang C, Ruan S, Zhang C, et al. Consistently low prevalence of syphilis among female sex workers in Jinan, China: findings from two consecutive respondent driven sampling surveys. PLoS One. 2012;7(4):e34085.
7. Wang Y, Pan JS. [The evaluation of accumulated effectiveness of interventions for AIDS/ STD among female sex workers in entertainment establishments in a district in Beijing] Chinese Journal of AIDS & STD. 2011;17(2):137-41.
8. Liu.J., Yu KP, Qiu RZ. [Analysis on Effect of Comprehensive Prevention and Control for AIDS in Hengyang from 2005 to 2009]. Practical Preventive Medicine. 2011;18(4):754-6.
9. Xu YF, Mo XJ, Liang HH, Zhou FH, Li P, Zhou J, et al. [Investigation on STD/AIDS knowledge and risk behaviors among commercial sex women in Nanning City]. Modern Preventive Medicine. 2007;34(21):4007-8, 11.
10. Li Y, Lin P, Detels R, Fu X, Deng Z, Liu Y, et al. [Prevalence of HIV infection and sexually transmitted disease and associated risk factors among female sex workers in Guangdong province]. Disease Surveillance. 2009;24(08):599-602.
11. Yu DY, Weng YQ. [Analysis on high-risk groups of AIDS comprehensive intervention effect participated by family planning department in Liuzhou city]. Chinese Journal of Pest Control. 2012;28(7):765-8.
12. Wang J, Wang T, Cen Y, Lai X, Li L, Chen C, et al. [Prevalence of sex transmitted disease or its related symptoms and associated risk factors among female sex workers in Zhongshan]. Journal of Tropical Medicine. 2010;10(4):477-80.
13. Hu S, Huang J, Li C, Huang J, Xuan R. [Survey on AIDS related knowledge and behaviors among female sex workers in entertainment places in Qingcheng district of Qingyuan City]. Occupation and Health. 2010;26(19):2218-30.
14. Lu P, Li C, Tang Z, Tan Q, Li D. [HIV /AIDS Behavioral Survey Among Female SexWorkers in CountrysideArea of Panzhihua City]. Journal of Preventive Medicine Information. 2008;24(8):608-10.
15. Huang J, Li C, Hu S, Xuan R, Huang J, Sun X, et al. [Analysis of Intervention Effect of AIDS High Risk Behavior among Female Sexual Workers in The Entertainment Places in Qingyuan City]. Journal of Tropical Medicine. 2010;10(9):1122-6.
