# Supplementary material for: Risk Behaviours among Female Sex Workers in China: A Systematic Review and Data Synthesis
Source: PLoS One. 2015 Mar 27;10(3):e0120595. doi: 10.1371/journal.pone.0120595 (PMC4376708; doi:10.1371/journal.pone.0120595)
Supplement: S3 Table — (PDF) [file pone.0120595.s005.pdf]

Table S3. Studies reported the rate of condom use in female sex workers with male clients.

First author, published year	Study period	Location	Province	Region	Recruitment venue	Sampling method	Measurement period*	Number of FSW used condom	Total number of FSW	Condom Usage (%)	QA Score
Qiu C, 2006 [1]	2000/08-2000/11	Ningde	Fujian	East	Detention Center	Cluster sampling	P1M	27	223	12.1%	4
Qiu C, 2006 [1]	2000/08-2000/11	Ningde	Fujian	East	Detention Center	Cluster sampling	LA	82	223	36.8%	4
Chen YL, 2009 [2]	2001	Longyan	Fujian	East	Detention Center	--	P1M	87	267	32.6%	5
Du YP, 2004 [3]	2001/11	Jingjiang	Jiangsu	East	Entertainment	--	LA	266	355	74.9%	4
Li L, 2003 [4]	2001/11-2001/12	--	Jiangsu	East	Entertainment	--	LA	181	355	51.0%	4
Qiu C, 2006 [1]	2001/08-2001/11	Ningde	Fujian	East	Detention Center	Cluster sampling	P1M	57	214	26.6%	4
Qiu C, 2006 [1]	2001/08-2001/11	Ningde	Fujian	East	Detention Center	Cluster sampling	LA	118	214	55.1%	4
Tang XL, 2003 [5]	2001/08-2001/12	Longyan	Fujian	East	Entertainment	Random sampling	LA	137	326	42.0%	4
Chen YL, 2009 [2]	2002	Longyan	Fujian	East	Detention Center	--	P1M	86	267	32.2%	5
Qiu C, 2003 [6]	2002/05	Ningde	Fujian	East	Entertainment	Cluster sampling	LA	301	1051	28.6%	3
Qiu C, 2003 [6]	2002/05	Ningde	Fujian	East	Entertainment	Cluster sampling	P1M	166	1051	15.8%	3
Qiu C, 2006 [1]	2002/08-2002/11	Ningde	Fujian	East	Detention Center	Cluster sampling	P1M	74	203	36.5%	4
Qiu C, 2006 [1]	2002/08-2002/11	Ningde	Fujian	East	Detention Center	Cluster sampling	LA	139	203	68.5%	4
Xiao Y, 2002 [7]	2002	Jiujiang	Jiangxi	East	Entertainment	Convenience sampling	P1M	26	201	12.9%	5
Chen YL, 2009 [2]	2003	Longyan	Fujian	East	Detention Center	--	P1M	110	252	43.7%	5
Li JX, 2007 [8]	2003	Jinan	Shandong	East	Entertainment	--	LA	289	500	57.8%	7

First author, published year	Study period	Location	Province	Region	Recruitment venue	Sampling method	Measurement period*	Number of FSW used condom	Total number of FSW	Condom Usage (%)	QA Score
Qiu C, 2006 [1]	2003/08-2003/11	Ningde	Fujian	East	Detention Center	Cluster sampling	P1M	97	209	46.4%	4
Qiu C, 2006 [1]	2003/08-2003/11	Ningde	Fujian	East	Detention Center	Cluster sampling	LA	129	209	61.7%	4
Chen YL, 2005 [9]	2004	Longyan	Fujian	East	Detention Center	--	LA	11	33	33.3%	4
Chen YL, 2005 [9]	2004	Longyan	Fujian	East	Detention Center	--	LA	64	139	46.0%	4
Chen YL, 2005 [9]	2004	Longyan	Fujian	East	Detention Center	--	LA	23	69	33.3%	4
Chen YL, 2009 [2]	2004	Longyan	Fujian	East	Detention Center	--	P1M	102	254	40.2%	5
Du YP, 2006 [10]	2004/10	Jingjiang	Jiangsu	East	Entertainment	--	LA	306	333	91.9%	4
Guo HY, 2008 [11]	2004/09	Heze	Shandong	East	Entertainment	Random sampling	LA	72	97	74.2%	4
He JG, 2005 [12]	2004/10-2004/12	Wuhu	Anhui	East	Entertainment	--	P1M	71	207	34.3%	5
He JG, 2005 [12]	2004/10-2004/12	Wuhu	Anhui	East	Entertainment	--	P1M	65	193	33.7%	5
Jin TL, 2005 [13]	2004/05	Lishui	Zhejiang	East	Entertainment	--	LA	118	137	86.1%	5
Li JX, 2007 [8]	2004	Jinan	Shandong	East	Entertainment	--	LA	281	640	43.9%	7
Li JZ, 2010 [14]	2004/06	Shandong	Shandong	East	Entertainment	Stratified cluster random sampling	LA	916	1218	75.2%	5
Li XF, 2006 [15]	2003/08-2003/10, 2004/05-2004/07	Qingdao	Shandong	East	Entertainment	Outreach	P1M	54	466	11.6%	7

First author, published year	Study period	Location	Province	Region	Recruitment venue	Sampling method	Measurement period*	Number of FSW used condom	Total number of FSW	Condom Usage (%)	QA Score
Li XF, 2006 [15]	2003/08-2003/10, 2004/05-2004/07	Qingdao	Shandong	East	Entertainment	Outreach	LA	317	466	68.0%	7
Li XJ, 2005 [16]	2004/10-2004/11	Hefei	Anhui	East	Entertainment	Two-stage cluster sampling	LA	202	225	89.8%	7
Li XJ, 2005 [16]	2004/10-2004/11	Hefei	Anhui	East	Entertainment	Two-stage cluster sampling	P1M	184	221	83.3%	7
Li XJ, 2005 [16]	2004/10-2004/11	Hefei	Anhui	East	Entertainment	Two-stage cluster sampling	LA	122	139	87.8%	7
Li XJ, 2005 [16]	2004/10-2004/11	Hefei	Anhui	East	Entertainment	Two-stage cluster sampling	P1M	101	139	72.7%	7
Liu XZ, 2006 [17]	2004	--	Shandong	East	VCT	--	P1M	541	3513	15.4%	4
Liu XZ, 2006 [17]	2004	--	Shandong	East	VCT	--	LA	854	3513	24.3%	4
Qiu C, 2006 [1]	2004/08-2004/11	Ningde	Fujian	East	Detention Center	Cluster sampling	P1M	96	193	49.7%	4
Qiu C, 2006 [1]	2004/08-2004/11	Ningde	Fujian	East	Detention Center	Cluster sampling	LA	133	193	68.9%	4
Shen YG, 2006 [18]	2004/10	Jiaxing	Zhejiang	East	Entertainment	Random sampling	LA	96	126	76.2%	3
Xiao HM, 2007 [19]	2004	Nanchang	Jiangxi	East	Entertainment	Random sampling	P1M	114	130	87.7%	4
Xiao HM, 2007 [19]	2004	Nanchang	Jiangxi	East	Entertainment	Random sampling	LA	122	130	93.8%	4
Xie Y, 2006 [20]	2002-2004	Zhangzhou	Fujian	East	Detention Center	--	P1M	137	532	25.8%	4

First author, published year	Study period	Location	Province	Region	Recruitment venue	Sampling method	Measurement period*	Number of FSW used condom	Total number of FSW	Condom Usage (%)	QA Score
Xiong ZW, 2005 [21]	2004	Nanchang	Jiangxi	East	Entertainment	Random sampling	LA	126	145	86.9%	3
Xiong ZW, 2005 [21]	2004	Nanchang	Jiangxi	East	Entertainment	Random sampling	P1M	123	145	84.8%	3
Yang JZ, 2005 [22]	2004	Hangzhou	Zhejiang	East	Entertainment	Two-stage sampling	P1M	96	314	30.6%	5
Yang JZ, 2005 [22]	2004	Hangzhou	Zhejiang	East	Entertainment	Two-stage sampling	LA	126	314	40.1%	5
Zheng H, 2012 [23]	2004/07-2004/08	Dan Yang	Jiangsu	East	Entertainment	--	LA	38	105	36.2%	3
Zheng H, 2012 [23]	2004/07-2004/08	Dan Yang	Jiangsu	East	Entertainment	--	P1M	12	105	11.4%	3
Zhu CQ, 2006 [24]	2004/08-2004/09	Shaoxing	Zhejiang	East	Entertainment	Random sampling	LA	116	307	37.8%	3
Zhu XY, 2008 [25]	2004/09-2004/10	Jiaozhou	Shandong	East	Entertainment	--	LA	230	360	63.9%	4
Zhu XY, 2008 [25]	2004/09-2004/10	Jiaozhou	Shandong	East	Entertainment	--	P1M	75	360	20.8%	4
Chen FW, 2007 [26]	2005/09	Nanchang	Jiangxi	East	Entertainment	Cluster sampling	P1M	66	310	21.3%	4
Chen YL, 2009 [2]	2005	Longyan	Fujian	East	Detention Center	--	P1M	141	345	40.9%	5
Fu SG, 2010 [27]	2005	--	Auhui	East	Sentinel sites	--	P1M	1223	2092	58.5%	5
Jiang XK, 2006 [28]	2005	Liaocheng	Shandong	East	Entertainment	--	LA	98	240	40.8%	4
Li JX, 2007 [8]	2003-2005	Jinan	Shandong	East	Entertainment	--	LA	652	1540	42.3%	7
Li JX, 2007 [8]	2005	Jinan	Shandong	East	Entertainment	--	LA	82	400	20.5%	7
Qiu C, 2006 [1]	2005/08-2005/11	Ningde	Fujian	East	Detention Center	Cluster sampling	P1M	98	184	53.3%	4
Qiu C, 2006 [1]	2005/08-2005/11	Ningde	Fujian	East	Detention Center	Cluster sampling	LA	131	184	71.2%	4

First author, published year	Study period	Location	Province	Region	Recruitment venue	Sampling method	Measurement period*	Number of FSW used condom	Total number of FSW	Condom Usage (%)	QA Score
Ruan SM, 2007 [29]	2005/09	Jinan	Shandong	East	Entertainment	Convenience sampling	P1M	177	188	94.1%	2
Wang J, 2008 [30]	2005	Jianhu	Jiangsu	East	Entertainment	--	LA	205	258	79.5%	6
Wang LW, 2006 [31]	2004/07-2005/01	Nanchang, Pingxiang	Jiangxi	East	Entertainment	Purposive and random sampling	LA	149	157	94.9%	6
Wang YH, 2006 [32]	2005/09	--	Jiangxi	East	Entertainment	--	LA	1076	1567	68.7%	2
Wang YH, 2006 [32]	2005/09	--	Jiangxi	East	Entertainment	--	P1M	591	1567	37.7%	2
Wu JJ, 2010 [33]	2005/06	Longyou	Zhejiang	East	Entertainment	Random sampling	LA	60	157	38.2%	5
Xu XH, 2007 [34]	2005/11	Jingning	Zhejiang	East	Entertainment	--	LA	80	102	78.4%	4
Yan HJ, 2007 [35]	2005	Zhangjiagang, Liyang, Tongzhou, Nanjing, Yancheng, Huaian	Jiangsu	East	Entertainment	Random sampling	LA	1209	1585	76.3%	3
Yu JF, 2007 [36]	2005	Cixi	Zhejiang	East	Entertainment	--	LA	135	237	57.0%	7
Yu JF, 2007 [36]	2005	Cixi	Zhejiang	East	Entertainment	--	P1M	49	237	20.7%	7
Zhao XP, 2006 [37]	2005/03	Suzhou	Jiangsu	East	Entertainment	--	LA	203	274	74.1%	4
Zhao XP, 2006 [37]	2005/03	Suzhou	Jiangsu	East	Entertainment	--	LA	102	126	81.0%	4
Zhu LD, 2007 [38]	2002-2005	Shanghai	Shanghai	East	Detention Center	--	LA	198	557	35.5%	6
Cai X, 2007 [39]	2006/08	Liaocheng	Shandong	East	Entertainment	Convenience sampling	LA	48	50	96.0%	1
Cai X, 2007 [39]	2006/08	Liaocheng	Shandong	East	Entertainment	Convenience sampling	LA	14	30	46.7%	1
Cai XF, 2007 [40]	2006/06-2006/07	Shanghai	Shanghai	East	Entertainment	Random sampling	P1M	39	102	38.2%	4

First author, published year	Study period	Location	Province	Region	Recruitment venue	Sampling method	Measurement period*	Number of FSW used condom	Total number of FSW	Condom Usage (%)	QA Score
Cha YF, 2008 [41]	2006/07-2006/09	Shanghai	Shanghai	East	--	--	LA	172	373	46.1%	4
Chen YL, 2009 [2]	2006	Longyan	Fujian	East	Detention Center	--	P1M	142	251	56.6%	5
Feng Z, 2007 [42]	2006/08	--	Anhui	East	Entertainment	Random sampling	LA	303	359	84.4%	4
Gu YB, 2007 [43]	2006	Funan	Anhui	East	Entertainment	Random sampling	LA	115	203	56.7%	4
Hu XB, 2009 [44]	2006/07-2006/09	Huaibei	Anhui	East	Entertainment	--	LA	93	105	88.6%	4
Hu XB, 2009 [44]	2006/07-2006/09	Huaibei	Anhui	East	Entertainment	--	LA	181	202	89.6%	4
Hu XB, 2009 [44]	2006/07-2006/09	Huaibei	Anhui	East	Entertainment	--	LA	80	98	81.6%	4
Huang Y, 2012 [45]	2006	Tong Ling	Anhui	East	Entertainment	--	LA	161	384	41.9%	4
Huang Y, 2012 [45]	2006	Tong Ling	Anhui	East	Entertainment	--	P1M	55	384	14.3%	4
Jiang GE, 2007 [46]	2006/08-2006/12	Yantai	Shandong	East	Entertainment	Convenience sampling	LA	202	265	76.2%	4
Jiang GE, 2007 [46]	2006/08-2006/12	Yantai	Shandong	East	Entertainment	Convenience sampling	P1M	192	265	72.5%	4
Li T, 2009 [47]	2006/10	Jiangxi	Zhejiang	East	Entertainment	Peer-referral	LA	558	800	69.8%	4
Liao M, 2012 [48]	2006	Dezhu, Yantai, Qingdao	Shandong	East	Entertainment	Venue-based sampling	P1M	360	1104	32.6%	5
Liu S, 2008 [49]	2006/11	Xintai	Shandong	East	Entertainment	--	LA	296	309	95.8%	3
Luo JF, 2009 [50]	2002-2006	Wuyishan	Fujian	East	Entertainment	--	P1M	375	982	38.2%	4
Luo JF, 2009 [50]	2002-2006	Wuyishan	Fujian	East	Entertainment	--	LA	667	982	67.9%	4
Luo Y, 2008 [51]	2006/04-2006/05	Hangzhou	Zhejiang	East	Detention Center	Continuous sampling	P1M	130	250	52.0%	6

First author, published year	Study period	Location	Province	Region	Recruitment venue	Sampling method	Measurement period*	Number of FSW used condom	Total number of FSW	Condom Usage (%)	QA Score
Luo Z, 2007 [52]	2006	Shanghai	Shanghai	East	Entertainment	--	P1M	97	373	26.0%	4
Luo Z, 2007 [52]	2006	Shanghai	Shanghai	East	Entertainment	--	LA	172	373	46.1%	4
Qi GP, 2007 [53]	2006/04-2006/05	Nanjing	Jiangsu	East	Entertainment	--	LA	179	209	85.6%	4
Sun ZF, 2009 [54]	2006/12	Bozhou	Anhui	East	Entertainment	--	LA	101	109	92.7%	3
Sun ZF, 2009 [54]	2006/12	Bozhou	Anhui	East	Entertainment	--	P1M	89	109	81.7%	3
Sun ZX, 2008 [55]	2006/06	Wenzhou	Zhejiang	East	Detention Center	--	LA	121	141	85.8%	5
Wang J, 2008 [30]	2006	Jianhu	Jiangsu	East	Entertainment	--	LA	225	268	84.0%	6
Wang YF, 2008 [56]	2005-2006	Qingdao	Shandong	East	Entertainment	--	LA	95	132	72.0%	4
Wu J, 2006 [57]	2006/06	Yingtan	Jiangxi	East	Entertainment	--	LA	145	284	51.1%	2
Wu J, 2006 [57]	2006/06	Yingtan	Jiangxi	East	Entertainment	--	P1M	89	284	31.3%	2
Xu SH, 2007 [58]	2006/02	Huian	Fujian	East	Entertainment	Random sampling	LA	16	75	21.3%	4
Xu SH, 2007 [58]	2006/02	Huian	Fujian	East	Entertainment	Random sampling	LA	18	65	27.7%	4
Yu X, 2007 [59]	2006/05	Haimen	Jiangsu	East	Entertainment	--	LA	90	121	74.4%	5
Zhang CQ, 2008 [60]	2006	Jinan	Shandong	East	Entertainment	Cluster sampling	LA	369	405	91.1%	4
Zhang XJ, 2012 [61]	2006	Qingdao, Yantai, Dezhou	Shandong	East	Entertainment	--	LA	635	1104	57.5%	4
Zhang XJ, 2012 [61]	2006	Qingdao, Yantai, Dezhou	Shandong	East	Entertainment	--	P1M	360	1104	32.6%	4
Zhu FG, 2009 [62]	2006/05	Yancheng	Jiangsu	East	Entertainment	--	LA	238	278	85.6%	4
Zhu HB, 2008 [63]	2006/06	Maamshan	Anhui	East	Entertainment	--	LA	187	403	46.4%	3
Zhu HB, 2008 [63]	2006/06	Maanshan	Anhui	East	Entertainment	--	P1M	97	402	24.1%	3

First author, published year	Study period	Location	Province	Region	Recruitment venue	Sampling method	Measurement period*	Number of FSW used condom	Total number of FSW	Condom Usage (%)	QA Score
Chen SP, 2010 [64]	2007/03-2007/04	Xunyang District	Jiangxi	East	Entertainment	Convenience sampling	LA	244	360	67.8%	7
Chen SP, 2010 [64]	2007/03-2007/04	Xunyang District	Jiangxi	East	Entertainment	Convenience sampling	P1M	179	353	50.7%	7
Chen YL, 2009 [2]	2007	Longyan	Fujian	East	Detention Center	--	P1M	107	223	48.0%	5
Cheng XL, 2008 [65]	2007/06-2007/11	Funan, Fuyang, Hefei	Anhui	East	Entertainment	Convenience sampling	LA	90	105	85.7%	4
Cui W, 2009 [66]	2007/12	Lixin	Anhui	East	Entertainment	Convenience sampling	LA	38	41	92.7%	3
Cui W, 2009 [66]	2007/12	Lixin	Anhui	East	Entertainment	Convenience sampling	P1M	38	41	92.7%	3
Fan HL, 2008 [67]	2007/01-2007/07	Shanghai	Shanghai	East	Entertainment	Convenience sampling	LA	155	301	51.5%	4
Fan HL, 2008 [67]	2007/01-2007/07	Shanghai	Shanghai	East	Entertainment	Convenience sampling	P1M	55	301	18.3%	4
Guo ZY, 2009 [68]	2007/01	Mengcheng	Anhui	East	Entertainment	--	LA	108	125	86.4%	5
Guo ZY, 2009 [68]	2007/01	Mengcheng	Anhui	East	Entertainment	--	P1M	104	125	83.2%	5
Huang Y, 2012 [45]	2007	Tong Ling	Anhui	East	Entertainment	--	LA	260	444	58.6%	4
Huang Y, 2012 [45]	2007	Tong Ling	Anhui	East	Entertainment	--	P1M	138	444	31.1%	4
Li N, 2009 [69]	2007/09-2007/12	Hefei	Anhui	East	Entertainment	Purposive sampling	P1M	256	335	76.4%	4
Liao M, 2012 [48]	2007	Dezhu, Yantai, Qingdao	Shandong	East	Entertainment	Venue-based sampling	P1M	642	1197	53.6%	5
Liao MZ, 2008 [70]	2007	--	Shandong	East	VCT	--	P1M	642	1197	53.6%	4
Liu YL, 2007 [71]	2007/04-2007/05	Ji'an	Jiangxi	East	Entertainment	--	LA	311	360	86.4%	4
Liu YL, 2007 [71]	2007/04-2007/05	Ji'an	Jiangxi	East	Entertainment	--	P1M	232	360	64.4%	4

First author, published year	Study period	Location	Province	Region	Recruitment venue	Sampling method	Measurement period*	Number of FSW used condom	Total number of FSW	Condom Usage (%)	QA Score
Luo Y, 2008 [72]	2007/02-2007/07	Hangzhou	Zhejiang	East	Entertainment	--	LA	179	257	69.6%	5
Luo Y, 2008 [72]	2007/02-2007/07	Hangzhou	Zhejiang	East	Entertainment	--	P1M	64	248	25.8%	5
Ni YQ, 2008 [73]	2007/07-2007/11	Shanghai	Shanghai	East	Entertainment	--	P1M	279	404	69.1%	6
Ni YQ, 2008 [73]	2007/07-2007/11	Shanghai	Shanghai	East	Entertainment	--	LA	383	404	94.8%	6
Peng B, 2008 [74]	2007/04-2007/06	Jiujiang	Jiangxi	East	Entertainment	--	P1M	170	250	68.0%	3
Peng B, 2008 [74]	2007/04-2007/06	Jiujiang	Jiangxi	East	Entertainment	--	LA	227	250	90.8%	3
Wang J, 2008 [30]	2007	Jianhu	Jiangsu	East	Entertainment	--	LA	261	273	95.6%	6
Wang L, 2008 [75]	2007/05-2007/06	Yixing	Jiangsu	East	Entertainment	--	LA	189	270	70.0%	4
Wang WM, 2008 [76]	2007/01-2007/12	Kunshan	Jiangsu	East	Detention Center	--	P1M	27	297	9.1%	4
Wang XM, 2011 [77]	2007	Cui Chang	Zhejiang	East	Entertainment	--	LA	238	255	93.3%	4
Wang XM, 2011 [77]	2007	Cui Chang	Zhejiang	East	Entertainment	--	P1M	212	255	83.1%	4
Xia JQ, 2010 [78]	2007	Nanchang	Jiangxi	East	Entertainment	Random sampling	LA	222	235	94.5%	5
Xu lq, 2008 [79]	2007/06-2007/07	Changshu	Jiangsu	East	Entertainment	Random sampling	LA	176	265	66.4%	2
Zhang XJ, 2012 [61]	2007	Qingdao, Yantai,Dezhou	Shandong	East	Entertainment	--	LA	943	1197	78.8%	4
Zhang XJ, 2012 [61]	2007	Qingdao, Yantai,Dezhou	Shandong	East	Entertainment	--	P1M	642	1197	53.6%	4
Zhou XL, 2008 [80]	2006/11-2007/03	Changzhou	Jiangsu	East	Entertainment	--	LA	94	167	56.3%	3
Chen JQ, 2009 [81]	2008/10-2008/11	Ningbo	Zhejiang	East	Entertainment	--	P1M	59	111	53.2%	3

First author, published year	Study period	Location	Province	Region	Recruitment venue	Sampling method	Measurement period*	Number of FSW used condom	Total number of FSW	Condom Usage (%)	QA Score
ChenL, 2009 [82]	2008	Shanghai	Shanghai	East	Entertainment	Continuous sampling	P1M	163	455	35.8%	5
ChenL, 2009 [82]	2008	Shanghai	Shanghai	East	Entertainment	Continuous sampling	LA	288	455	63.3%	5
Gong PT, 2009 [83]	2008/12	--	Anhui	East	Entertainment	--	LA	475	498	95.4%	3
Gong PT, 2009 [83]	2008/12	--	Anhui	East	Entertainment	--	P1M	469	498	94.2%	3
He X, 2012 [84]	2008/07	Yu Yao	Zhejiang	East	Entertainment	--	LA	151	298	50.7%	4
He X, 2012 [84]	2008/07	Yu Yao	Zhejiang	East	Entertainment	--	P1M	94	298	31.5%	4
Hu CC, 2011 [85]	2008/08	Nan Chang	Jiangxi	East	Entertainment	--	LA	6	42	14.3%	4
Hu CC, 2011 [85]	2008/08	Nan Chang	Jiangxi	East	Entertainment	--	LA	133	199	66.8%	4
Hu CC, 2011 [85]	2008/08	Nan Chang	Jiangxi	East	Entertainment	--	P1M	6	42	14.3%	4
Hu CC, 2011 [85]	2008/08	Nan Chang	Jiangxi	East	Entertainment	--	P1M	51	199	25.6%	4
Huang Y, 2012 [45]	2008	Tong Ling	Anhui	East	Entertainment	--	LA	244	402	60.7%	4
Huang Y, 2012 [45]	2008	Tong Ling	Anhui	East	Entertainment	--	P1M	124	402	30.8%	4
Jin YL, 2009 [86]	2008/05-2008/06	Wuhu	Anhui	East	Entertainment	Random sampling	LA	355	432	82.2%	4
Jin YL, 2009 [86]	2008/05-2008/06	Wuhu	Anhui	East	Entertainment	Random sampling	P1M	242	432	56.0%	4
Jin YL, 2009 [86]	2008/05-2008/06	Wuhu	Anhui	East	Entertainment	Random sampling	LA	56	68	82.4%	4
Jin YL, 2009 [86]	2008/05-2008/06	Wuhu	Anhui	East	Entertainment	Random sampling	P1M	23	68	33.8%	4
Liao M, 2011 [87]	2006-2008	Qingdao	Shandong	East	Entertainment	Convenience sampling	LA	1003	1207	83.1%	4
Liao M, 2011 [87]	2006-2008	Qingdao	Shandong	East	Entertainment	Convenience sampling	P1M	504	1202	41.9%	4
Liao M, 2012 [88]	2008/02-2008/08	Jinan	Shandong	East	Entertainment	Respondent-Driven Sampling	P1M	225	359	62.7%	6

First author, published year	Study period	Location	Province	Region	Recruitment venue	Sampling method	Measurement period*	Number of FSW used condom	Total number of FSW	Condom Usage (%)	QA Score
Liao M, 2012 [48]	2008	Dezhu, Yantai, Qingdao	Shandong	East	Entertainment	Venue-based sampling	P1M	557	1156	48.2%	5
Liu ZL, 2011 [89]	2008	Zha Bei	Shanghai	East	Entertainment	Purposive sampling	LA	152	164	92.7%	4
Liu ZL, 2011 [89]	2008	Zha Bei	Shanghai	East	Entertainment	Purposive sampling	LA	152	164	92.7%	4
Lu QL, 2009 [90]	2008	Shaoxing	Zhejiang	East	Entertainment	Two-stage cluster sampling	P1M	240	322	74.5%	3
Lu QL, 2009 [90]	2008	Shaoxing	Zhejiang	East	Entertainment	Two-stage cluster sampling	P1M	42	78	53.8%	3
Tang X, 2010 [91]	2008/10-2008/11	Shanghai	Shanghai	East	Entertainment	--	LA	126	285	44.2%	6
Tang X, 2012 [92]	2008/07	Hong Kou	Shanghai	East	Entertainment	--	LA	34	113	30.1%	4
Tang X, 2012 [92]	2008/07	Hong Kou	Shanghai	East	Entertainment	--	LA	46	131	35.1%	4
Tang X, 2012 [92]	2008/07	Hong Kou	Shanghai	East	Entertainment	--	LA	80	244	32.8%	4
Wang FH, 2009 [93]	2008	-	Anhui	East	Entertainment	--	LA	3640	4584	79.4%	2
Wang FH, 2009 [93]	2008	-	Anhui	East	Entertainment	--	P1M	2370	4584	51.7%	2
Wang XM, 2011 [77]	2008	Cui Chang	Zhejiang	East	Entertainment	--	LA	203	236	86.0%	4
Wang XM, 2011 [77]	2008	Cui Chang	Zhejiang	East	Entertainment	--	LA	227	236	96.2%	4
Xia JQ, 2010 [78]	2008	Nanchang	Jiangxi	East	Entertainment	Random sampling	LA	281	312	90.1%	5
Xue FH, 2009 [94]	2008/04-2008/06	Wenzhou	Zhejiang	East	Entertainment	Random sampling	LA	266	403	66.0%	7
Xue FH, 2009 [94]	2008/04-2008/06	Wenzhou	Zhejiang	East	Entertainment	Random sampling	P1M	239	403	59.3%	7
Yang Y, 2011 [95]	2008/06-2008/10	Shanghai	Shanghai	East	Entertainment	--	LA	70	89	78.7%	5

First author, published year	Study period	Location	Province	Region	Recruitment venue	Sampling method	Measurement period*	Number of FSW used condom	Total number of FSW	Condom Usage (%)	QA Score
Zhang XJ, 2012 [61]	2008	Qingdao, Yantai, Dezhou	Shandong	East	Entertainment	--	LA	922	1158	79.6%	4
Zhang XJ, 2012 [61]	2006-2008	Qingdao, Yantai, Dezhou	Shandong	East	Entertainment	--	LA	2500	3459	72.3%	4
Zhang XJ, 2012 [61]	2008	Qingdao, Yantai, Dezhou	Shandong	East	Entertainment	--	P1M	557	1156	48.2%	4
Zhang YH, 2011 [96]	2008/04-2008/07	Nanjing	Jiangsu	East	--	--	LA	318	400	79.5%	2
Zhang YH, 2011 [96]	2008/04-2008/07	Qingdao	Shandong	East	--	--	LA	357	400	89.3%	2
Zhang YH, 2011 [96]	2008/04-2008/07	Shanghai	Shanghai	East	--	--	LA	375	416	90.1%	2
Zhang YH, 2011 [96]	2008/04-2008/07	Hangzhou	Zhejiang	East	--	--	LA	391	460	85.0%	2
Zhang YH, 2011 [96]	2008/04-2008/07	Hangzhou	Zhejiang	East	--	--	P1M	308	460	67.0%	2
Zhang YH, 2011 [96]	2008/04-2008/07	Shanghai	Shanghai	East	--	--	P1M	262	416	63.0%	2
Zhang YH, 2011 [96]	2008/04-2008/07	Nanjing	Jiangsu	East	--	--	P1M	231	400	57.8%	2
Zhang YH, 2011 [96]	2008/04-2008/07	Qingdao	Shandong	East	--	--	P1M	237	400	59.3%	2
Zhao YQ, 2010c[97]	2008/04-2008/05	Suzhou	Jiangsu	East	Entertainment	Cluster sampling	LA	53	76	69.7%	4
Zhao YQ, 2010 [97]	2008/04-2008/05	Suzhou	Jiangsu	East	Entertainment	Cluster sampling	P1M	27	76	35.5%	4
Zhao YQ, 2010 [97]	2008/04-2008/05	Suzhou	Jiangsu	East	Entertainment	Cluster sampling	LA	265	275	96.4%	4

First author, published year	Study period	Location	Province	Region	Recruitment venue	Sampling method	Measurement period*	Number of FSW used condom	Total number of FSW	Condom Usage (%)	QA Score
Zhao YQ, 2010 [97]	2008/04-2008/05	Suzhou	Jiangsu	East	Entertainment	Cluster sampling	P1M	225	275	81.8%	4
Zhao YQ, 2010 [97]	2008/04-2008/05	Suzhou	Jiangsu	East	Entertainment	Cluster sampling	LA	34	45	75.6%	4
Zhao YQ, 2010 [97]	2008/04-2008/05	Suzhou	Jiangsu	East	Entertainment	Cluster sampling	P1M	19	45	42.2%	4
Zhou XM, 2010 [98]	2008	Nanjing	Jiangsu	East	Entertainment	--	LA	288	358	80.4%	3
Zhou XM, 2010 [98]	2008	Nanjing	Jiangsu	East	Entertainment	--	P1M	259	358	72.3%	3
Cai Y, 2010 [99]	2009/03-2009/12	Shanghai	Shanghai	East	Entertainment	Random sampling	LA	302	324	93.2%	8
Chen GS, 2010 [100]	2009/04-2009/06	--	Jiangxi	East	Entertainment	Ethnographic target sampling	P1M	281	401	70.1%	6
Chen SX, 2011 [101]	2009/07-2009/09	Gao Mi	Shandong	East	Entertainment	--	LA	198	236	83.9%	4
Chen SX, 2011 [101]	2009/07-2009/09	Gao Mi	Shandong	East	Entertainment	--	P1M	132	236	55.9%	4
Chen ZH, 2012 [102]	2009	Zhu Ji	Zhejiang	East	Entertainment	--	LA	245	409	59.9%	3
Gan WH, 2012 [103]	2009	Jin Shan	Shanghai	East	Sentinel sites	Cluster sampling	LA	232	413	56.2%	3
Hu CC, 2011 [85]	2009/06	Nan Chang	Jiangxi	East	Entertainment	--	LA	40	50	80.0%	4
Hu CC, 2011 [85]	2009/06	Nan Chang	Jiangxi	East	Entertainment	--	LA	175	200	87.5%	4
Huang Y, 2012 [45]	2009	Tong Ling	Anhui	East	Entertainment	--	LA	273	412	66.3%	4
Huang Y, 2012 [45]	2009	Tong Ling	Anhui	East	Entertainment	--	P1M	105	412	25.5%	4
Jin HJ, 2010 [104]	2009/04-2009/07	Ningbo	Zhejiang	East	Sentinel sites	Cluster sampling, convenience sampling	LA	138	163	84.7%	4

First author, published year	Study period	Location	Province	Region	Recruitment venue	Sampling method	Measurement period*	Number of FSW used condom	Total number of FSW	Condom Usage (%)	QA Score
Jin HJ, 2010 [104]	2009/04-2009/07	Ningbo	Zhejiang	East	Sentinel sites	Cluster sampling, convenience sampling	LA	221	230	96.1%	4
Kang D, 2011 [105]	2006-2009	Qingdao	Shandong	East	Entertainment	Venue-based, community outreach and peer-referral	LA	97	120	80.8%	6
Kang D, 2011 [105]	2006-2009	Qingdao	Shandong	East	Entertainment	Venue-based, community outreach and peer-referral	P1M	36	117	30.8%	6
Kang D, 2011 [105]	2006-2009	Qingdao	Shandong	East	Entertainment	Venue-based, community outreach and peer-referral	LA	681	821	82.9%	6
Kang D, 2011 [105]	2006-2009	Qingdao	Shandong	East	Entertainment	Venue-based, community outreach and peer-referral	P1M	346	819	42.2%	6
Kang D, 2011 [105]	2006-2009	Qingdao	Shandong	East	Entertainment	Venue-based, community outreach and peer-referral	LA	206	243	84.8%	6
Kang D, 2011 [105]	2006-2009	Qingdao	Shandong	East	Entertainment	Venue-based, community outreach and peer-referral	P1M	111	243	45.7%	6
Liao M, 2012 [88]	2009/05-2009/10	Jinan	Shandong	East	Entertainment	Respondent-Driven Sampling	P1M	211	430	49.1%	6

First author, published year	Study period	Location	Province	Region	Recruitment venue	Sampling method	Measurement period*	Number of FSW used condom	Total number of FSW	Condom Usage (%)	QA Score
Liao MZ, 2010 [106]	2009	--	Shandong	East	Mixed venues	--	P1M	2467	4221	58.4%	4
Liao MZ, 2010 [106]	2009	--	Shandong	East	Mixed venues	--	LA	3416	4236	80.6%	4
Luo Y, 2010 [107]	2009/03	Huainan	Anhui	East	Entertainment	--	LA	209	319	65.5%	4
Shao MC, 2010 [108]	2009/04	Suzhou	Jiangsu	East	Entertainment	Stratified cluster random sampling	LA	375	402	93.3%	1
Shao MC, 2010 [108]	2009.4	Suzhou	Jiangsu	East	Entertainment	Stratified cluster random sampling	P1M	322	402	80.1%	1
Tang X, 2012 [92]	2009	Hong Kou	Shanghai	East	Entertainment	--	LA	45	163	27.6%	4
Tang X, 2012 [92]	2009	Hong Kou	Shanghai	East	Entertainment	--	LA	83	197	42.1%	4
Tang X, 2012 [92]	2009	Hong Kou	Shanghai	East	Entertainment	--	LA	128	360	35.6%	4
Wan LJ, 2011 [109]	2008-2009	Shang Yu	Zhejiang	East	Entertainment	Two stage cluster sampling	LA	210	288	72.9%	4
Wan LJ, 2011 [109]	2008-2009	Shang Yu	Zhejiang	East	Entertainment	Two stage cluster sampling	P1M	164	288	56.9%	4
Wang F, 2010 [110]	2009	Bengbu	Anhui	East	Entertainment	--	LA	24	75	32.0%	4
Wang F, 2010 [110]	2009	Bengbu	Anhui	East	Entertainment	--	P1M	12	75	16.0%	4
Wang XM, 2011 [77]	2009	Cui Chang	Zhejiang	East	Entertainment	--	LA	224	237	94.5%	4
Wang XM, 2011 [77]	2009	Cui Chang	Zhejiang	East	Entertainment	--	P1M	202	237	85.2%	4
Wei ZY, 2010 [111]	2009/07-2009/09	Yangzhou	Jiangsu	East	Entertainment	Cluster sampling	LA	84	225	37.3%	5
Wei ZY, 2010 [111]	2009/07-2009/09	Yangzhou	Jiangsu	East	Entertainment	Cluster sampling	P1M	38	225	16.9%	5
Wei ZY, 2010 [111]	2009/07-2009/09	Yangzhou	Jiangsu	East	Entertainment	Cluster sampling	LA	445	702	63.4%	5

First author, published year	Study period	Location	Province	Region	Recruitment venue	Sampling method	Measurement period*	Number of FSW used condom	Total number of FSW	Condom Usage (%)	QA Score
Wei ZY, 2010 [111]	2009/07-2009/09	Yangzhou	Jiangsu	East	Entertainment	Cluster sampling	P1M	270	702	38.5%	5
Xi SJ, 2010 [112]	2009	Hangzhou	Zhejiang	East	Entertainment	--	LA	190	210	90.5%	5
Xi SJ, 2011 [113]	2009/04-2009/06	Hangzhou	Zhejiang	East	Entertainment	--	LA	389	446	87.2%	4
Xi SJ, 2011 [113]	2009/04-2009/06	Hangzhou	Zhejiang	East	Entertainment	--	P1M	341	446	76.5%	4
XI SJ, 2011 [114]	2009/04-2009/06	Hangzhou	Zhejiang	East	Entertainment	Cluster sampling	LA	389	446	87.2%	5
XI SJ, 2011 [114]	2009/04-2009/06	Hangzhou	Zhejiang	East	Entertainment	Cluster sampling	P1M	341	446	76.5%	5
Yang LQ, 2012 [115]	2009	Tong Xiang	Zhejiang	East	Entertainment	--	LA	289	403	71.7%	3
Yang LQ, 2012 [115]	2009	Tong Xiang	Zhejiang	East	Entertainment	--	P1M	239	403	59.3%	3
Yang Y, 2011 [95]	2009/05-2009/08	Shanghai	Shanghai	East	Entertainment	--	LA	155	189	82.0%	5
Yao Y, 2010 [116]	2009	Fuzhou	Fujian	East	Entertainment	--	LA	187	225	83.1%	3
Yao Y, 2010 [116]	2009	Fuzhou	Fujian	East	Entertainment	--	P1M	116	225	51.6%	3
Zhang QQ, 2012 [115]	2009/10-2009/12	Yang Zhou, Chang Zhou	Jiangsu	East	Entertainment	--	LA	532	648	82.1%	4
Zhang QQ, 2012 [115]	2009/07-2009/09	Yang Zhou, Chang Zhou	Jiangsu	East	Entertainment	--	LA	1248	1568	79.6%	4
Zhang QQ, 2012 [115]	2009/07-2009/09	Yang Zhou, Chang Zhou	Jiangsu	East	Entertainment	--	P1M	850	1505	56.5%	4
Zhang QQ, 2012 [115]	2009/10-2009/12	Yang Zhou, Chang Zhou	Jiangsu	East	Entertainment	--	P1M	360	619	58.2%	4
Chen CG, 2011 [117]	2009/11-2010/01	Fu Zhou	Fujian	East	Entertainment	--	LA	222	225	98.7%	2
Chen CG, 2011 [117]	2009/11-2010/01	Fu Zhou	Fujian	East	Entertainment	--	P1M	184	225	81.8%	2
Chen ZH, 2012 [102]	2010	Zhu Ji	Zhejiang	East	Entertainment	--	LA	287	400	71.8%	3

First author, published year	Study period	Location	Province	Region	Recruitment venue	Sampling method	Measurement period*	Number of FSW used condom	Total number of FSW	Condom Usage (%)	QA Score
Gan WH, 2012 [103]	2010	Jin Shan	Shanghai	East	Sentinel sites	Cluster sampling	LA	362	413	87.7%	3
He X, 2012 [84]	2010/12	Yu Yao	Zhejiang	East	Entertainment	--	LA	283	295	95.9%	4
Jiang J, 2012 [118]	2010/07	Ning Bo	Zhejiang	East	Detention Center	--	LA	262	439	59.7%	4
Jiang J, 2012 [118]	2010/07	Ning Bo	Zhejiang	East	Detention Center	--	P1M	162	439	36.9%	4
Kang DM, 2011 [119]	2009-2010	Heze, Caoxian, Chengwu, Zoucheng, Xintai, Pingdu	Shandong	East	Entertainment	Cluster, convenience sampling	LA	1073	1157	92.7%	3
Kang DM, 2011 [119]	2009-2010	Dezhou, Dongming, Gaomi, Lue Nan, Longkou, Pingyi, Rushan, Yanggu, Huaiyin	Shandong	East	Entertainment	Cluster, convenience sampling	LA	1300	1506	86.3%	3
Kang DM, 2011 [119]	2009-2010	Heze, Caoxian, Chengwu, Zoucheng, Xintai, Pingdu	Shandong	East	Entertainment	Cluster, convenience sampling	P1M	1004	1157	86.8%	3

First author, published year	Study period	Location	Province	Region	Recruitment venue	Sampling method	Measurement period*	Number of FSW used condom	Total number of FSW	Condom Usage (%)	QA Score
Kang DM, 2011 [119]	2009-2010	Dezhou, Dongming, Gaomi, Lue Nan, Longkou, Pingyi, Rushan, Yanggu, Huaiyin	Shandong	East	Entertainment	Cluster, convenience sampling	P1M	995	1506	66.1%	3
Liu LL, 2011 [120]	2010/04-2010/06	Jiang Yan	Jiangsu	East	Entertainment	Cluster sampling	LA	338	407	83.0%	6
Liu LL, 2011 [120]	2010/04-2010/06	Jiang Yan	Jiangsu	East	Entertainment	Cluster sampling	P1M	302	407	74.2%	6
Ma P, 2011 [121]	2010	Nantong	Jiangsu	East	Mixed venues	--	LA	422	696	60.6%	3
Miao XL, 2011 [122]	2010	Wu Xi	Jiangsu	East	Entertainment	--	LA	541	800	67.6%	4
Qian ZH, 2012 [123]	2009-2010	Suzhou	Jiangsu	East	Detention Center	--	LA	513	578	88.8%	4
Qian ZH, 2012 [123]	2009-2010	Suzhou	Jiangsu	East	Detention Center	--	P1M	355	578	61.4%	4
Sun XQ, 2011 [124]	2010	Tai He	Anhui	East	Entertainment	--	LA	297	354	83.9%	4
Sun XQ, 2011 [124]	2010	Tai He	Anhui	East	Entertainment	--	P1M	265	319	83.1%	4
Wang XM, 2011 [77]	2010	Cui Chang	Zhejiang	East	Entertainment	--	LA	289	295	98.0%	4
Wang XM, 2011 [77]	2010	Cui Chang	Zhejiang	East	Entertainment	--	P1M	252	295	85.4%	4
Wu SB, 2012 [125]	2010/01-2010/08	15 Counties	Fujian	East	Entertainment	--	LA	222	358	62.0%	4
Wu SB, 2012 [125]	2010/01-2010/08	15 Counties	Fujian	East	Entertainment	--	P1M	190	358	53.1%	4
Yang LQ, 2012 [115]	2010	Tong Xiang	Zhejiang	East	Entertainment	--	LA	311	426	73.0%	3
Yang LQ, 2012 [115]	2010	Tong Xiang	Zhejiang	East	Entertainment	--	P1M	218	426	51.2%	3
Zheng H, 2012 [23]	2010/08-2010/09	Dan Yang	Jiangsu	East	Entertainment	--	LA	743	916	81.1%	3

First author, published year	Study period	Location	Province	Region	Recruitment venue	Sampling method	Measurement period*	Number of FSW used condom	Total number of FSW	Condom Usage (%)	QA Score
Chen G, 2012 [126]	2011/07	Dong Yang, Yu Yao, Qu Zhou	Zhejiang	East	Entertainment	--	LA	341	514	66.3%	3
Chen G, 2012 [126]	2011/07	Dong Yang, Yu Yao, Qu Zhou	Zhejiang	East	Entertainment	--	LA	398	514	77.4%	3
Chen G, 2012 [126]	2011/11	Dong Yang, Yu Yao, Qu Zhou	Zhejiang	East	Entertainment	--	LA	456	504	90.5%	3
Chen ZH, 2012 [102]	2011	Zhu Ji	Zhejiang	East	Entertainment	--	LA	264	354	74.6%	3
Chen ZH, 2012 [102]	2009-2011	Zhu Ji	Zhejiang	East	Entertainment	--	LA	796	1163	68.4%	3
Gan WH, 2012 [103]	2011	Jin Shan	Shanghai	East	Sentinel sites	Cluster sampling	LA	361	414	87.2%	3
Ling Z, 2012 [127]	2011/12	Long You	Zhejiang	East	Entertainment	--	P1M	47	244	19.3%	4
Pan GL, 2012 [128]	2011/04-2011/07	Ning Bo	Zhejiang	East	Entertainment	--	LA	81	88	92.0%	4
Pan GL, 2012 [128]	2011/04-2011/07	Ning Bo	Zhejiang	East	Entertainment	--	LA	503	617	81.5%	4
Pan GL, 2012 [128]	2011/04-2011/07	Ning Bo	Zhejiang	East	Entertainment	--	LA	207	274	75.5%	4
Pan GL, 2012 [128]	2011/04-2011/07	Ning Bo	Zhejiang	East	Entertainment	--	LA	215	255	84.3%	4
Pan GL, 2012 [128]	2011/04-2011/07	Ning Bo	Zhejiang	East	Entertainment	--	P1M	446	617	72.3%	4
Pan GL, 2012 [128]	2011/04-2011/07	Ning Bo	Zhejiang	East	Entertainment	--	P1M	75	88	85.2%	4
Pan GL, 2012 [128]	2011/04-2011/07	Ning Bo	Zhejiang	East	Entertainment	--	P1M	188	255	73.7%	4
Pan GL, 2012 [128]	2011/04-2011/07	Ning Bo	Zhejiang	East	Entertainment	--	P1M	183	274	66.8%	4

First author, published year	Study period	Location	Province	Region	Recruitment venue	Sampling method	Measurement period*	Number of FSW used condom	Total number of FSW	Condom Usage (%)	QA Score
Qin CZ, 2012 [129]	2010/06-2011/04	Jiang Yan	Jiangsu	East	Entertainment	Cluster sampling	LA	690	817	84.5%	4
Qin CZ, 2012 [129]	2010/06-2011/04	Jiang Yan	Jiangsu	East	Entertainment	Cluster sampling	P1M	632	809	78.1%	4
Qin QR, 2012 [130]	2011/04-2011/06	Ma An Shan	Anhui	East	Entertainment	--	LA	173	319	54.2%	4
Qin QR, 2012 [130]	2011/11-2011/12	Ma An Shan	Anhui	East	Entertainment	--	LA	234	302	77.5%	4
Qin QR, 2012 [131]	2011/04-2011/06	Ma An Shan	Anhui	East	Entertainment	--	LA	173	319	54.2%	4
Qin QR, 2012 [130]	2011/04 - 2011/06	Ma An Shan	Anhui	East	Entertainment	--	P1M	138	319	43.3%	4
Qin QR, 2012 [131]	2011/04-2011/06	Ma An Shan	Anhui	East	Entertainment	--	P1M	138	319	43.3%	4
Qiu ZH, 2012 [132]	2011/04-2011/07	Hu Zhou	Zhejiang	East	Entertainment	--	LA	286	314	91.1%	4
Qiu ZH, 2012 [132]	2011/04-2011/07	Hu Zhou	Zhejiang	East	Entertainment	--	P1M	151	400	37.8%	4
Qiu ZH, 2012 [132]	2011/04-2011/07	Hu Zhou	Zhejiang	East	Entertainment	--	P1M	25	79	31.6%	4
Qiu ZH, 2012 [132]	2011/04-2011/07	Hu Zhou	Zhejiang	East	Entertainment	--	P1M	96	249	38.6%	4
Qiu ZH, 2012 [132]	2011/04-2011/07	Hu Zhou	Zhejiang	East	Entertainment	--	P1M	30	72	41.7%	4
Sun BJ, 2012 [133]	2011	Zao Zhuang	Shandong	East	Entertainment	--	LA	291	333	87.4%	4
Tao SF, 2012 [134]	2011/06-2011/12	Zong Yang	Anhui	East	Entertainment	--	LA	15	40	37.5%	2
Wang DL, 2012 [135]	2011	Chang Zhou	Jiangsu	East	Sentinel sites	Cluster sampling	LA	279	384	72.7%	4

First author, published year	Study period	Location	Province	Region	Recruitment venue	Sampling method	Measurement period*	Number of FSW used condom	Total number of FSW	Condom Usage (%)	QA Score
Wang DL, 2012 [135]	2011	Chang Zhou	Jiangsu	East	Sentinel sites	Cluster sampling	P1M	97	114	85.1%	4
Wu J, 2012 [136]	2011	Wu Xi	Jiangsu	East	Entertainment	--	LA	361	405	89.1%	4
Wu J, 2012 [136]	2011	Wu Xi	Jiangsu	East	Entertainment	--	LA	77	97	79.4%	4
Wu J, 2012 [136]	2011	Wu Xi	Jiangsu	East	Entertainment	--	LA	284	308	92.2%	4
Wu J, 2012 [136]	2011	Wu Xi	Jiangsu	East	Entertainment	--	P1M	271	405	66.9%	4
Wu J, 2012 [136]	2011	Wu Xi	Jiangsu	East	Entertainment	--	P1M	232	308	75.3%	4
Wu J, 2012 [136]	2011	Wu Xi	Jiangsu	East	Entertainment	--	P1M	40	97	41.2%	4
Xu JS, 2012 [137]	2011/04-2011/07	--	Jiangsu	East	--	--	LA	1311	1577	83.1%	4
Xu JS, 2012 [137]	2011/04-2011/07	--	Jiangsu	East	--	--	LA	3037	3676	82.6%	4
Xu JS, 2012 [137]	2011/04-2011/07	--	Jiangsu	East	--	--	LA	4425	5366	82.5%	4
Xu JS, 2012 [137]	2011/04-2011/07	--	Jiangsu	East	--	--	LA	8654	10694	80.9%	4
Xu JS, 2012 [137]	2011/04-2011/07	--	Jiangsu	East	--	--	P1M	6084	10604	57.4%	4
Xu JS, 2012 [137]	2011/04-2011/07	--	Jiangsu	East	--	--	P1M	996	1573	63.3%	4
Xu JS, 2012 [137]	2011/04-2011/07	--	Jiangsu	East	--	--	P1M	3041	5360	56.7%	4
Xu JS, 2012 [137]	2011/04-2011/07	--	Jiangsu	East	--	--	P1M	2046	3671	55.7%	4
Yang LQ, 2012 [115]	2011	Tong Xiang	Zhejiang	East	Entertainment	--	LA	369	440	83.9%	3
Yang LQ, 2012 [115]	2011	Tong Xiang	Zhejiang	East	Entertainment	--	P1M	250	440	56.8%	3
Yang YH, 2012 [138]	2011	Quan Zhou	Fujian	East	Sentinel sites	Cluster sampling	LA	560	868	64.5%	4
Yang YH, 2012 [138]	2011	Quan Zhou	Fujian	East	Sentinel sites	Cluster sampling	P1M	458	868	52.8%	4

First author, published year	Study period	Location	Province	Region	Recruitment venue	Sampling method	Measurement period*	Number of FSW used condom	Total number of FSW	Condom Usage (%)	QA Score
Ye ZM, 2012 [139]	2011/04-2011/07	Wenzhou	Zhejiang	East	Entertainment	Convenience sampling	LA	511	830	61.6%	3
Ye ZM, 2012 [139]	2011/04-2011/07	Wenzhou	Zhejiang	East	Entertainment	Convenience sampling	P1M	284	823	34.5%	3
Ye ZM, 2012 [139]	2011/04-2011/07	Wenzhou	Zhejiang	East	Entertainment	Convenience sampling	P1M	10	114	8.8%	3
Ye ZM, 2012 [139]	2011/04-2011/07	Wenzhou	Zhejiang	East	Entertainment	Convenience sampling	P1M	191	545	35.0%	3
Ye ZM, 2012 [139]	2011/04-2011/07	Wenzhou	Zhejiang	East	Entertainment	Convenience sampling	P1M	93	171	54.4%	3
Zheng H, 2012 [23]	2011/06 -2011-07	Dan Yang	Jiangsu	East	Entertainment	--	LA	640	762	84.0%	3
Zhu HW, 2012 [140]	2011/04-2011/07	Nan Chang	Jiangxi	East	Entertainment	--	LA	364	400	91.0%	4
Zhu HW, 2012 [140]	2011/04-2011/07	Nan Chang	Jiangxi	East	Entertainment	--	P1M	138	400	34.5%	4
Song ZP, 2004 [141]	2002/07-2002/08	Taiyuan	Shanxi	North	Entertainment	--	P1M	63	100	63.0%	4
Guo HY, 2008 [11]	2004/09	Langfang	Heibei	North	Entertainment	Random sampling	LA	97	99	98.0%	4
Ren XY, 2006 [142]	2004/07-2004/08	Hehehaote	Inner Mongolia	North	Entertainment	--	LA	137	179	76.5%	4
Ren XY, 2006 [142]	2004/07-2004/08	Hehehaote	Inner Mongolia	North	Entertainment	--	P1M	99	179	55.3%	4
Ren XY, 2006 [142]	2004/07-2004/08	Hehehaote	Inner Mongolia	North	Entertainment	--	LA	80	149	53.7%	4
Ren XY, 2006 [142]	2004/07-2004/08	Hehehaote	Inner Mongolia	North	Entertainment	--	P1M	57	149	38.3%	4
Ren XY, 2006 [142]	2004/07-2004/08	Hehehaote	Inner Mongolia	North	Entertainment	--	LA	53	135	39.3%	4

First author, published year	Study period	Location	Province	Region	Recruitment venue	Sampling method	Measurement period*	Number of FSW used condom	Total number of FSW	Condom Usage (%)	QA Score
Ren XY, 2006 [142]	2004/07-2004/08	Hehehaote	Inner Mongolia	North	Entertainment	--	P1M	34	135	25.2%	4
Zhao RL, 2005 [143]	2004/08-2004/10	Tongliao	Inner Mongolia	North	Entertainment	--	P1M	288	324	88.9%	4
Zhao YJ, 2007 [144]	2004/12	Beijing	Beijing	North	Entertainment	Random sampling	LA	40	57	70.2%	4
Bai JM, 2006 [145]	2005/03-2005/05	Beijing	Beijing	North	Entertainment	Cluster sampling	LA	78	112	69.6%	4
Li GY, 2008 [146]	2005	Beijing	Beijing	North	Entertainment	Cluster sampling	LA	78	115	67.8%	6
Li GY, 2008 [146]	2005	Beijing	Beijing	North	Entertainment	Cluster sampling	P1M	56	115	48.7%	6
Li GY, 2008 [146]	2005	Beijing	Beijing	North	Entertainment	Cluster sampling	LA	29	40	72.5%	6
Li GY, 2008 [146]	2005	Beijing	Beijing	North	Entertainment	Cluster sampling	P1M	22	40	55.0%	6
Lin Z, 2007 [147]	2005/08-2005/09	Tongliao	Inner Mongolia	North	Entertainment	Convenience sampling	P1M	111	364	30.5%	5
Lin Z, 2007 [147]	2005/08-2005/09	Tongliao	Inner Mongolia	North	Entertainment	Convenience sampling	LA	280	364	76.9%	5
Wang ST, 2009 [148]	2005	--	Shanxi	North	Entertainment	Snowball sampling	LA	147	168	87.5%	1
Bai JM, 2007 [149]	2006/10-2006/12	Beijing	Beijing	North	Entertainment	Random sampling	P1M	125	196	63.8%	4
Bo FB, 2007 [150]	2006/08-2006/09	Huhehaote	Inner Mongolia	North	Entertainment	Random sampling	P1M	245	617	39.7%	3
Bo FB, 2007 [150]	2006/08-2006/09	Huhehaote	Inner Mongolia	North	Entertainment	Random sampling	LA	374	617	60.6%	3
Deng PX, 2008 [151]	2006/07-2006/09	Beijing	Beijing	North	Entertainment	Random sampling	P1M	32	55	58.2%	3

First author, published year	Study period	Location	Province	Region	Recruitment venue	Sampling method	Measurement period*	Number of FSW used condom	Total number of FSW	Condom Usage (%)	QA Score
Deng PX, 2008 [151]	2006/07-2006/09	Beijing	Beijing	North	Entertainment	Random sampling	P1M	22	39	56.4%	3
Fu SG, 2010 [27]	2006	-	Tianjin	North	Sentinel sites	--	P1M	1908	2589	73.7%	5
Jia HZ, 2012 [152]	2006	Mi Yun	Beijing	North	Entertainment	--	LA	129	146	88.4%	4
Jia HZ, 2012 [152]	2006	Mi Yun	Beijing	North	Entertainment	--	P1M	107	146	73.3%	4
Liu HX, 2011 [153]	2006	Chang Ping	Beijing	North	--	--	LA	74	105	70.5%	3
Liu HX, 2011 [153]	2006	Chang Ping	Beijing	North	--	--	P1M	35	105	33.3%	3
Liu LR, 2007 [154]	2006/05-2006/10	Beijing	Beijing	North	Entertainment	Venue-based sampling	LA	247	339	72.9%	7
Liu LR, 2007 [154]	2006/05-2006/10	Beijing	Beijing	North	Entertainment	Venue-based sampling	P1M	149	338	44.1%	7
Shi WY, 2012 [155]	2006	Feng Tai	Beijing	North	Entertainment	--	LA	186	198	93.9%	4
Shi WY, 2012 [155]	2006	Feng Tai	Beijing	North	Entertainment	--	LA	211	267	79.0%	4
Shi WY, 2012 [155]	2006	Feng Tai	Beijing	North	Entertainment	--	P1M	149	198	75.3%	4
Shi WY, 2012 [155]	2006	Feng Tai	Beijing	North	Entertainment	--	P1M	188	267	70.4%	4
Shi Y, 2013 [156]	2006/07-2006/10	Hohhot	Inner Mongolia	North	Entertainment	Stratified cluster sampling	LA	379	624	60.7%	5
Shi Y, 2013 [156]	2006/07-2006/10	Hohhot	Inner Mongolia	North	Entertainment	Stratified cluster sampling	P1M	248	624	39.7%	5
Wang ST, 2009 [148]	2006	--	Shanxi	North	Entertainment	Snowball sampling	LA	233	260	89.6%	1
Yang YR, 2012 [157]	2006	Bao Tou	Inner Mongolia	North	Entertainment	--	LA	277	435	63.7%	4
Yang YR, 2012 [157]	2006	Bao Tou	Inner Mongolia	North	Entertainment	--	P1M	239	435	54.9%	4
Bai JJ, 2010 [158]	2007	Tianjin	Tianjin	North	Entertainment	Random sampling	P1M	89	336	26.5%	7
Bai JJ, 2010 [158]	2007	Tianjin	Tianjin	North	Entertainment	Random sampling	LA	136	336	40.5%	7

First author, published year	Study period	Location	Province	Region	Recruitment venue	Sampling method	Measurement period*	Number of FSW used condom	Total number of FSW	Condom Usage (%)	QA Score
Dong XY, 2009 [159]	2007/08-2007/10	Tianjin	Tianjin	North	Detention Center	--	LA	131	178	73.6%	5
Jia HZ, 2012 [152]	2007	Mi Yun	Beijing	North	Entertainment	--	LA	82	103	79.6%	4
Jia HZ, 2012 [152]	2007	Mi Yun	Beijing	North	Entertainment	--	P1M	10	103	9.7%	4
Liu HX, 2011 [153]	2007	Chang Ping	Beijing	North	--	--	LA	63	101	62.4%	3
Liu HX, 2011 [153]	2007	Chang Ping	Beijing	North	--	--	P1M	28	101	27.7%	3
Liu YQ, 2008 [160]	2006/07-2007/09	Beijing	Beijing	North	Entertainment	Random sampling	P1M	125	202	61.9%	4
Shi WY, 2012 [155]	2007	Feng Tai	Beijing	North	Entertainment	--	LA	179	202	88.6%	4
Shi WY, 2012 [155]	2007	Feng Tai	Beijing	North	Entertainment	--	LA	398	452	88.1%	4
Shi WY, 2012 [155]	2007	Feng Tai	Beijing	North	Entertainment	--	P1M	159	202	78.7%	4
Shi WY, 2012 [155]	2007	Feng Tai	Beijing	North	Entertainment	--	P1M	313	452	69.2%	4
Shi Y, 2013 [156]	2007/07-2007/10	Hohhot	Inner Mongolia	North	Entertainment	Stratified cluster sampling	LA	325	444	73.2%	5
Shi Y, 2013 [156]	2007/07-2007/10	Hohhot	Inner Mongolia	North	Entertainment	Stratified cluster sampling	P1M	246	444	55.4%	5
Wang HY, 2008 [161]	2007/08-2007/11	Beijing	Beijing	North	Entertainment	--	LA	83	151	55.0%	5
Wang HY, 2008 [161]	2007/08-2007/11	Beijing	Beijing	North	Entertainment	--	P1M	42	151	27.8%	5
Wang ST, 2009 [148]	2007	-	Shanxi	North	Entertainment	Snowball sampling	LA	339	349	97.1%	1
Yang YR, 2012 [157]	2007	Bao Tou	Inner Mongolia	North	Entertainment	--	LA	369	456	80.9%	4
Yang YR, 2012 [157]	2007	Bao Tou	Inner Mongolia	North	Entertainment	--	P1M	314	456	68.9%	4
Jia HZ, 2012 [152]	2008	Mi Yun	Beijing	North	Entertainment	--	LA	85	109	78.0%	4
Jia HZ, 2012 [152]	2008	Mi Yun	Beijing	North	Entertainment	--	P1M	40	109	36.7%	4
Li JE, 2012 [162]	2008/03	Han Dan	Hebei	North	Entertainment	--	LA	276	370	74.6%	2

First author, published year	Study period	Location	Province	Region	Recruitment venue	Sampling method	Measurement period*	Number of FSW used condom	Total number of FSW	Condom Usage (%)	QA Score
Li JE, 2012 [162]	2008/03	Han Dan	Hebei	North	Entertainment	--	P1M	165	370	44.6%	2
Liu HX, 2011 [153]	2008	Chang Ping	Beijing	North	--	--	LA	91	101	90.1%	3
Liu HX, 2011 [153]	2008	Chang Ping	Beijing	North	--	--	P1M	10	101	9.9%	3
Shi WY, 2012 [155]	2008	Feng Tai	Beijing	North	Entertainment	--	LA	192	208	92.3%	4
Shi WY, 2012 [155]	2008	Feng Tai	Beijing	North	Entertainment	--	LA	209	236	88.6%	4
Shi WY, 2012 [155]	2008	Feng Tai	Beijing	North	Entertainment	--	P1M	163	208	78.4%	4
Shi WY, 2012 [155]	2008	Feng Tai	Beijing	North	Entertainment	--	P1M	182	236	77.1%	4
Shi Y, 2013 [156]	2008/07-2008/10	Hohhot	Inner Mongolia	North	Entertainment	Stratified cluster sampling	LA	389	451	86.3%	5
Shi Y, 2013 [156]	2008/07-2008/10	Hohhot	Inner Mongolia	North	Entertainment	Stratified cluster sampling	P1M	269	451	59.6%	5
Wang ST, 2009 [148]	2008	--	Shanxi	North	Entertainment	Snowball sampling	LA	309	310	99.7%	1
Yang YR, 2012 [157]	2008	Bao Tou	Inner Mongolia	North	Entertainment	--	LA	436	485	89.9%	4
Yang YR, 2012 [157]	2008	Bao Tou	Inner Mongolia	North	Entertainment	--	P1M	371	485	76.5%	4
Zhang YH, 2011 [96]	2008/04-2008/07	Tianjin	Tianjin	North	--	--	LA	393	534	73.6%	2
Zhang YH, 2011 [96]	2008/04-2008/07	Beijing	Beijing	North	--	--	LA	1941	2688	72.2%	2
Zhang YH, 2011 [96]	2008/04-2008/07	Beijing	Beijing	North	--	--	P1M	1153	2688	42.9%	2
Zhang YH, 2011 [96]	2008/04-2008/07	Tianjin	Tianjin	North	--	--	P1M	201	534	37.6%	2
Jia HZ, 2012 [152]	2009	Mi Yun	Beijing	North	Entertainment	--	LA	69	117	59.0%	4
Jia HZ, 2012 [152]	2009	Mi Yun	Beijing	North	Entertainment	--	P1M	51	117	43.6%	4
Jia J, 2011 [163]	2009	Xian	Shanxi	North	Entertainment	--	LA	254	296	85.8%	3
Jia J, 2011 [163]	2009	Xian	Shanxi	North	Entertainment	--	P1M	251	296	84.8%	3

First author, published year	Study period	Location	Province	Region	Recruitment venue	Sampling method	Measurement period*	Number of FSW used condom	Total number of FSW	Condom Usage (%)	QA Score
Jiang DK, 2010 [164]	2009	Ankang	Shanxi	North	Mixed venues	--	LA	173	380	45.5%	6
Li M, 2012 [165]	2009	Xi Cheng	Beijing	North	Entertainment	--	LA	131	200	65.5%	4
Li M, 2012 [165]	2009	Xi Cheng	Beijing	North	Entertainment	--	P1M	78	200	39.0%	4
Liu HX, 2011 [153]	2009	Chang Ping	Beijing	North	--	--	LA	74	113	65.5%	3
Liu HX, 2011 [153]	2009	Chang Ping	Beijing	North	--	--	P1M	57	113	50.4%	3
Shi WY, 2012 [155]	2009	Feng Tai	Beijing	North	Entertainment	--	LA	191	202	94.6%	4
Shi WY, 2012 [155]	2009	Feng Tai	Beijing	North	Entertainment	--	LA	268	292	91.8%	4
Shi WY, 2012 [155]	2009	Feng Tai	Beijing	North	Entertainment	--	P1M	168	202	83.2%	4
Shi WY, 2012 [155]	2009	Feng Tai	Beijing	North	Entertainment	--	P1M	151	292	51.7%	4
Xu XY, 2011 [166]	2009	Hohhot	Inner Mongolia	North	Sentinel sites	--	LA	290	400	72.5%	3
Xu XY, 2011 [166]	2009	Hohhot	Inner Mongolia	North	Sentinel sites	--	P1M	206	400	51.5%	3
Yang YR, 2012 [157]	2009	Bao Tou	Inner Mongolia	North	Entertainment	--	LA	404	441	91.6%	4
Yang YR, 2012 [157]	2009	Bao Tou	Inner Mongolia	North	Entertainment	--	P1M	355	441	80.5%	4
Cao H, 2010 [167]	2009/07-2010/06	Tianjin	Tianjin	North	Entertainment	Random sampling	P1M	117	157	74.5%	5
Cao H, 2010 [167]	2009/07-2010/06	Tianjin	Tianjin	North	Entertainment	Random sampling	LA	146	166	88.0%	5
Feng N, 2011 [168]	2010/04-2010/06	Datong	Shanxi	North	Sentinel sites	Cluster sampling	LA	372	400	93.0%	4
Feng N, 2011 [168]	2010/04-2010/06	Datong	Shanxi	North	Sentinel sites	Cluster sampling	P1M	303	400	75.8%	4
Jia HZ, 2012 [152]	2010	Mi Yun	Beijing	North	Entertainment	--	LA	92	100	92.0%	4
Jia HZ, 2012 [152]	2010	Mi Yun	Beijing	North	Entertainment	--	P1M	75	100	75.0%	4
Li BY, 2012 [169]	2010/04-2010/07	Jin Nan	Tianjin	North	--	--	LA	285	407	70.0%	4
Li BY, 2012 [169]	2010/04-2010/07	Jin Nan	Tianjin	North	--	--	P1M	193	407	47.4%	4
Li M, 2012 [165]	2010	Xi Cheng	Beijing	North	Entertainment	--	LA	92	200	46.0%	4

First author, published year	Study period	Location	Province	Region	Recruitment venue	Sampling method	Measurement period*	Number of FSW used condom	Total number of FSW	Condom Usage (%)	QA Score
Li M, 2012 [165]	2010	Xi Cheng	Beijing	North	Entertainment	--	P1M	58	200	29.0%	4
Liu HX, 2011 [153]	2010	Chang Ping	Beijing	North	--	--	LA	61	105	58.1%	3
Liu HX, 2011 [153]	2010	Chang Ping	Beijing	North	--	--	P1M	39	105	37.1%	3
Jia HZ, 2012 [152]	2011	Mi Yun	Beijing	North	Entertainment	--	LA	41	102	40.2%	4
Jia HZ, 2012 [152]	2006-2011	Mi Yun	Beijing	North	Entertainment	--	LA	497	677	73.4%	4
Jia HZ, 2012 [152]	2011	Mi Yun	Beijing	North	Entertainment	--	P1M	20	102	19.6%	4
Li F, 2012 [48]	2011	Chang Ping	Beijing	North	Detention Center	--	LA	266	290	91.7%	4
Li F, 2012 [48]	2011	Chang Ping	Beijing	North	Detention Center	--	P1M	192	290	66.2%	4
Li JE, 2012 [162]	2011	Han Dan	Hebei	North	Entertainment	--	LA	347	365	95.1%	2
Li M, 2012 [165]	2011	Xi Cheng	Beijing	North	Entertainment	--	LA	186	200	93.0%	4
Li M, 2012 [165]	2009-2011	Xi Cheng	Beijing	North	Entertainment	--	LA	391	600	65.2%	4
Li M, 2012 [165]	2011	Xi Cheng	Beijing	North	Entertainment	--	P1M	85	200	42.5%	4
Liu ZJ, 2012 [170]	2011/04-2011/06	Luan Nan	Hebei	North	Entertainment	--	LA	227	400	56.8%	4
Liu ZJ, 2012 [170]	2011/04-2011/06	Luan Nan	Hebei	North	Entertainment	--	P1M	140	335	41.8%	4
Guo HY, 2008 [11]	2004/09	Shenyang	Liaoning	Northeast	Entertainment	Random sampling	LA	87	105	82.9%	4
Cui YZ, 2011 [171]	2006	Haerbin	Heilongjiang	Northeast	Entertainment	--	LA	179	417	42.9%	4
Cui YZ, 2011 [171]	2006	Haerbin	Heilongjiang	Northeast	Entertainment	--	P1M	41	417	9.8%	4
Lai XF, 2010 [172]	2006/07-2006/08	Tonghua	Jilin	Northeast	Entertainment	Stratified cluster sampling	LA	82	400	20.5%	5

First author, published year	Study period	Location	Province	Region	Recruitment venue	Sampling method	Measurement period*	Number of FSW used condom	Total number of FSW	Condom Usage (%)	QA Score
Lai XF, 2010 [172]	2006/07-2006/08	Tonghua	Jilin	Northeast	Entertainment	Stratified cluster sampling	P1M	53	400	13.3%	5
Wang XL, 2008 [173]	2006/08	Shenyang	Liaoning	Northeast	Entertainment	Cluster sampling	LA	361	650	55.5%	4
Wang XL, 2008 [173]	2006/08	Shenyang	Liaoning	Northeast	Entertainment	Cluster sampling	P1M	270	650	41.5%	4
Zhang QH, 2007 [174]	2006/09	Shenyang	Liaoning	Northeast	Entertainment	--	P1M	271	420	64.5%	5
Zhao L, 2012 [175]	2006	An Shan	Liaoning	Northeast	Entertainment	--	LA	265	498	53.2%	4
Zhao L, 2012 [175]	2006	An Shan	Liaoning	Northeast	Entertainment	--	P1M	122	495	24.6%	4
Cui YZ, 2011 [171]	2007	Haerbin	Heilongjiang	Northeast	Entertainment	--	LA	341	447	76.3%	4
Cui YZ, 2011 [171]	2007	Haerbin	Heilongjiang	Northeast	Entertainment	--	P1M	237	447	53.0%	4
Li Y, 2008 [176]	2007/04	Huanggu	Liaoning	Northeast	Entertainment	--	P1M	74	160	46.3%	4
Li Y, 2008 [176]	2007/04	Huanggu	Liaoning	Northeast	Entertainment	--	LA	90	160	56.3%	4
Liu WB, 2010 [177]	2007/06-2007/09	Tonghua	Jilin	Northeast	Entertainment	Cluster convenience sampling	LA	344	410	83.9%	5
Liu WB, 2010 [177]	2007/06-2007/09	Tonghua	Jilin	Northeast	Entertainment	Cluster convenience sampling	P1M	285	410	69.5%	5
Cui YZ, 2011 [171]	2008	Haerbin	Heilongjiang	Northeast	Entertainment	--	LA	375	410	91.5%	4
Cui YZ, 2011 [171]	2008	Haerbin	Heilongjiang	Northeast	Entertainment	--	P1M	281	410	68.5%	4
Shao B, 2011 [178]	2008	13 Cities	Heilongjiang	Northeast	Entertainment	--	LA	4116	5055	81.4%	4
Shao B, 2011 [178]	2008	13 Cities	Heilongjiang	Northeast	Entertainment	--	P1M	1898	5055	37.5%	4
Zhang YH, 2011 [96]	2008/04-2008/07	Haerbin	Heilongjiang	Northeast	--	--	LA	340	447	76.1%	2
Zhang YH, 2011 [96]	2008/04-2008/07	Shenyang	Liaoning	Northeast	--	--	LA	364	601	60.6%	2

First author, published year	Study period	Location	Province	Region	Recruitment venue	Sampling method	Measurement period*	Number of FSW used condom	Total number of FSW	Condom Usage (%)	QA Score
Zhang YH, 2011 [96]	2008/04-2008/07	Shenyang	Liaoning	Northeast	--	--	P1M	234	601	38.9%	2
Zhang YH, 2011 [96]	2008/04-2008/07	Haerbin	Heilongjiang	Northeast	--	--	P1M	236	447	52.8%	2
Zhao L, 2012 [175]	2008	An Shan	Liaoning	Northeast	Entertainment	--	LA	401	459	87.4%	4
Cui YZ, 2011 [171]	2009	Haerbin	Heilongjiang	Northeast	Entertainment	--	LA	411	447	91.9%	4
Cui YZ, 2011 [171]	2009	Haerbin	Heilongjiang	Northeast	Entertainment	--	P1M	339	447	75.8%	4
Zheng J, 2012 [179]	2009/04-2009/06	An Shan	Liaoning	Northeast	Entertainment	Cluster sampling	P1M	207	400	51.8%	4
Li Y, 2011 [180]	2010	--	Heilongjiang	Northeast	Sentinel sites	--	LA	6671	7547	88.4%	4
Li Y, 2011 [180]	2010	--	Heilongjiang	Northeast	Sentinel sites	--	P1M	3782	7547	50.1%	4
Xian XJ, 2011 [181]	2011	Nong An	Jilin	Northeast	Sentinel sites	Cluster sampling	LA	351	400	87.8%	3
Xian XJ, 2011 [181]	2011	Nong An	Jilin	Northeast	Sentinel sites	Cluster sampling	P1M	315	399	78.9%	3
Zhou D, 2012 [182]	2011	--	Liaoning	Northeast	Sentinel sites	--	LA	5623	6633	84.8%	4
Zhou D, 2012 [182]	2011	--	Liaoning	Northeast	Sentinel sites	--	P1M	4138	6582	62.9%	4
Li SL, 2004 [183]	2002/03	Wulumuqi	Xinjiang	Northwest	Entertainment	Venue-based sampling	LA	76	109	69.7%	5
Ni MJ, 2005 [184]	2003/12-2004/02	Kashi	Xinjiang	Northwest	Entertainment	Probability proportional to size sampling	LA	118	300	39.3%	7
Ni MJ, 2005 [184]	2003/12-2004/02	Kashi	Xinjiang	Northwest	Entertainment	Probability proportional to size sampling	P1M	86	300	28.7%	7
Song YR, 2005 [185]	2004/08	Alaer	Xinjiang	Northwest	Entertainment	--	LA	83	258	32.2%	4
Song YR, 2005 [185]	2004/08	Alaer	Xinjiang	Northwest	Entertainment	--	P1M	42	258	16.3%	4

First author, published year	Study period	Location	Province	Region	Recruitment venue	Sampling method	Measurement period*	Number of FSW used condom	Total number of FSW	Condom Usage (%)	QA Score
Wang XZ, 2005 [186]	2004/05-2004/06	Ningxia	Ningxia	Northwest	Entertainment	Cluster random sampling	LA	124	332	37.3%	5
Zhang M, 2006 [187]	1999-2004	Wulumuqi	Xinjiang	Northwest	Detention Center	--	LA	62	120	51.7%	3
Cai RLM, 2009 [188]	2005	Gannan	Gansu	Northwest	Entertainment	Two-stage clustered randomized sampling	LA	437	1107	39.5%	4
Cai RLM, 2009 [188]	2005	Gannan	Gansu	Northwest	Entertainment	Two-stage clustered randomized sampling	P1M	440	1107	39.7%	4
Chang WH, 2008 [189]	2004/11-2005/04	-	Shaanxi	Northwest	Entertainment	Convenience sampling	LA	49	63	77.8%	5
Lin L, 2009 [190]	2005	Karamay	Xinjiang	Northwest	Sentinel sites	Continuous sampling	LA	21	67	31.3%	4
Lin L, 2009 [190]	2005	Karamay	Xinjiang	Northwest	Sentinel sites	Continuous sampling	P1M	13	36	36.1%	4
Lin L, 2009 [190]	2006	Karamay	Xinjiang	Northwest	Sentinel sites	Continuous sampling	LA	500	755	66.2%	4
Lin L, 2009 [190]	2006	Karamay	Xinjiang	Northwest	Sentinel sites	Continuous sampling	P1M	238	755	31.5%	4
Liu YX, 2007 [191]	2006/08-2006/11	Yinchuan	Ningxia	Northwest	Entertainment	--	LA	219	415	52.8%	4
Liu YX, 2007 [191]	2006/08-2006/11	Yinchuan	Ningxia	Northwest	Entertainment	--	P1M	180	415	43.4%	4
Ma HMT, 2007 [192]	2005/12-2006/03	Qoqek	Xinjiang	Northwest	Entertainment	--	LA	1079	1204	89.6%	5
Ma HMT, 2007 [192]	2005/12-2006/03	Qoqek	Xinjiang	Northwest	Entertainment	--	P1M	679	1204	56.4%	5

First author, published year	Study period	Location	Province	Region	Recruitment venue	Sampling method	Measurement period*	Number of FSW used condom	Total number of FSW	Condom Usage (%)	QA Score
Ma JX, 2012 [193]	2006	Bai Yin	Gansu	Northwest	Entertainment	--	LA	282	403	70.0%	4
Ma JX, 2012 [193]	2006	Bai Yin	Gansu	Northwest	Entertainment	--	P1M	239	403	59.3%	4
Song Y, 2006 [194]	2006	Tulufan	Xinjiang	Northwest	Entertainment	cluster random sampling	LA	59	103	57.3%	4
Mahemuti Y, 2007 [195]	2005/12-2006/03	Tacheng	Xinjiang	Northwest	Entertainment	--	LA	1079	1204	89.6%	2
Mahemuti Y, 2007 [195]	2005/12-2006/03	Tacheng	Xinjiang	Northwest	Entertainment	--	P1M	679	1204	56.4%	2
Zeng KF, 2008 [196]	2006/01-2006/02	Kelamayi	Xinjiang	Northwest	Entertainment	--	LA	516	755	68.3%	3
Zeng KF, 2008 [196]	2006/01-2006/02	Kelamayi	Xinjiang	Northwest	Entertainment	--	P1M	94	298	31.5%	3
Zhang L, 2007 [197]	2005/11-2006/05	Lanzhou	Gansu	Northwest	Entertainment	--	LA	230	376	61.2%	1
Zhang L, 2007 [197]	2005/11-2006/05	Lanzhou	Gansu	Northwest	Entertainment	--	P1M	191	376	50.8%	1
Zheng CJ, 2008 [198]	2006	-	Xinjiang	Northwest	Entertainment	--	LA	539	689	78.2%	5
Amydam MAYT, 2008 [199]	2007	Wulumuqi	Xinjiang	Northwest	Entertainment	Convenience sampling	P1M	421	603	69.8%	7
Lin B, 2009 [200]	2007/01	Karamay	Xinjiang	Northwest	Entertainment	--	LA	118	182	64.8%	1
Lin B, 2009 [200]	2007/07	Kelamayi	Xinjiang	Northwest	Entertainment	--	P1M	102	182	56.0%	1
Lin L, 2009 [190]	2007	Karamay	Xinjiang	Northwest	Sentinel sites	Continuous sampling	LA	118	182	64.8%	4
Lin L, 2009 [190]	2007	Karamay	Xinjiang	Northwest	Sentinel sites	Continuous sampling	P1M	102	182	56.0%	4
Ma JX, 2012 [193]	2007	Bai Yin	Gansu	Northwest	Entertainment	--	LA	296	418	70.8%	4
Ma JX, 2012 [193]	2007	Bai Yin	Gansu	Northwest	Entertainment	--	P1M	224	418	53.6%	4
Wang MX, 2008 [201]	2007/07	Shanyang	Shaanxi	Northwest	Entertainment	--	P1M	25	30	83.3%	3

First author, published year	Study period	Location	Province	Region	Recruitment venue	Sampling method	Measurement period*	Number of FSW used condom	Total number of FSW	Condom Usage (%)	QA Score
Yang SP, 2008 [202]	2007/08-2007/09	Hami And Hetian	Xinjiang	Northwest	Entertainment	--	LA	168	200	84.0%	3
Fu SG, 2010 [27]	2008	-	Shaanxi	Northwest	Sentinel sites	--	P1M	1638	2827	57.9%	5
Liang SP, 2010 [203]	2008/05	Huinong	Ningxia	Northwest	--	--	LA	269	299	90.0%	4
Liang SP, 2010 [203]	2008/05	Huinong	Ningxia	Northwest	--	--	P1M	179	299	59.9%	4
Lin L, 2009 [190]	2008	Karamay	Xinjiang	Northwest	Sentinel sites	Continuous sampling	LA	163	169	96.4%	4
Lin L, 2009 [190]	2008	Karamay	Xinjiang	Northwest	Sentinel sites	Continuous sampling	P1M	114	169	67.5%	4
Liu SH, 2009 [204]	2008/04-2008/05	Huinong	Ningxia	Northwest	Entertainment	Random sampling	LA	194	236	82.2%	7
Liu SH, 2009 [204]	2008/04-2008/05	Huinong	Ningxia	Northwest	Entertainment	Random sampling	P1M	112	236	47.5%	7
Liu SH, 2009 [204]	2008/04-2008/05	Huinong	Ningxia	Northwest	Entertainment	Random sampling	LA	101	130	77.7%	7
Liu SH, 2009 [204]	2008/04-2008/05	Huinong	Ningxia	Northwest	Entertainment	Random sampling	P1M	35	130	26.9%	7
Ma JX, 2012 [193]	2008	Bai Yin	Gansu	Northwest	Entertainment	--	LA	355	402	88.3%	4
Ma JX, 2012 [193]	2008	Bai Yin	Gansu	Northwest	Entertainment	--	P1M	212	402	52.7%	4
Zhang XQ, 2010 [205]	2008.1	Gaolan	Gansu	Northwest	Entertainment	--	LA	133	150	88.7%	3
Zhang XQ, 2010 [205]	2008.1	Gaolan	Gansu	Northwest	Entertainment	--	P1M	128	150	85.3%	3
Zhang YH, 2011 [96]	2008/04-2008/07	Xian	Shaanxi	Northwest	--	--	LA	352	400	88.0%	2
Zhang YH, 2011 [96]	2008/04-2008/07	Xian	Shaanxi	Northwest	--	--	P1M	344	400	86.0%	2
Zhu Q, 2009 [206]	2008	Changji	Xinjiang	Northwest	Entertainment	--	LA	261	400	65.3%	4

First author, published year	Study period	Location	Province	Region	Recruitment venue	Sampling method	Measurement period*	Number of FSW used condom	Total number of FSW	Condom Usage (%)	QA Score
Gao W, 2012 [207]	2008/11-2009/01	Chenguan (Lanzhou)	Gansu	Northwest	Entertainment	Proportional stratified random sampling	LA	318	347	91.6%	7
Gao W, 2012 [207]	2008/11-2009/01	Chenguan (Lanzhou)	Gansu	Northwest	Entertainment	Proportional stratified random sampling	P1M	285	347	82.1%	7
Ma JX, 2012 [193]	2009	Bai Yin	Gansu	Northwest	Entertainment	--	LA	359	400	89.8%	4
Ma JX, 2012 [193]	2009	Bai Yin	Gansu	Northwest	Entertainment	--	P1M	278	400	69.5%	4
Ma L, 2011 [208]	2009/06	Hu Tu Bi	Xinjiang	Northwest	Entertainment	--	LA	93	144	64.6%	4
Ma L, 2011 [208]	2009/06	Hu Tu Bi	Xinjiang	Northwest	Entertainment	--	P1M	75	132	56.8%	4
Xu YJ, 2011 [209]	2009	--	Shaanxi	Northwest	--	--	LA	3558	4334	82.1%	4
Xu YJ, 2011 [209]	2009	--	Shaanxi	Northwest	--	--	P1M	2686	4226	63.6%	4
Xu YJ, 2011 [209]	2009	--	Shaanxi	Northwest	--	--	LA	505	569	88.8%	4
Xu YJ, 2011 [209]	2009	--	Shaanxi	Northwest	--	--	P1M	425	541	78.6%	4
Chang WH, 2011 [210]	2010	--	Shaanxi	Northwest	Sentinel sites	Cluster sampling	LA	3565	4439	80.3%	4
Chang WH, 2012 [210]	2010	--	Shaanxi	Northwest	Sentinel sites	Cluster sampling	P1M	2637	4439	59.4%	4
Guo H, 2012 [211]	2010	Long Nan	Gansu	Northwest	Entertainment	--	LA	252	279	90.3%	3
Guo H, 2012 [211]	2010	Long Nan	Gansu	Northwest	Entertainment	--	P1M	146	362	40.3%	3
Ma JX, 2012 [193]	2010	Bai Yin	Gansu	Northwest	Entertainment	--	LA	310	400	77.5%	4
Ma JX, 2012 [193]	2010	Bai Yin	Gansu	Northwest	Entertainment	--	P1M	209	400	52.3%	4
Ma L, 2011 [208]	2010/06	Hu Tu Bi	Xinjiang	Northwest	Entertainment	--	LA	150	150	100.0%	4
Tao LD, 2011 [212]	2010/04-2010/10	Lan Zhou	Gansu	Northwest	Entertainment	--	LA	1110	1200	92.5%	4
Tao LD, 2011 [212]	2010/04-2010/10	Lan Zhou	Gansu	Northwest	Entertainment	--	P1M	940	1200	78.3%	4

First author, published year	Study period	Location	Province	Region	Recruitment venue	Sampling method	Measurement period*	Number of FSW used condom	Total number of FSW	Condom Usage (%)	QA Score
Wu R, 2012 [213]	2010	Bole	Xinjiang	Northwest	Entertainment	--	LA	91	174	52.3%	4
Wu R, 2012 [213]	2010	Bole	Xinjiang	Northwest	Entertainment	--	P1M	69	174	39.7%	4
Zhang MN, 2011 [214]	2010/06	Lin Fen	Shaanxi	Northwest	Entertainment	--	LA	295	400	73.8%	4
Zhang MN, 2011 [214]	2010/06	Lin Fen	Shaanxi	Northwest	Entertainment	--	P1M	245	400	61.3%	4
Zhao GD, 2011 [215]	2010/04-2010/06	Shang Luo	Shaanxi	Northwest	Sentinel sites	Cluster sampling	LA	348	390	89.2%	4
Zhao GD, 2011 [215]	2010/04-2010/06	Shang Luo	Shaanxi	Northwest	Sentinel sites	Cluster sampling	P1M	233	386	60.4%	4
Guo H, 2012 [211]	2011	Long Nan	Gansu	Northwest	Entertainment	--	LA	296	399	74.2%	3
Guo H, 2012 [211]	2010-2011	Long Nan	Gansu	Northwest	Entertainment	--	LA	575	751	76.6%	3
Guo H, 2012 [211]	2011	Long Nan	Gansu	Northwest	Entertainment	--	P1M	93	400	23.3%	3
Guo H, 2012 [211]	2010-2011	Long Nan	Gansu	Northwest	Entertainment	--	P1M	238	763	31.2%	3
Huang HT, 2012 [103]	2011/04-2011/07	Gu Yuan	Ningxia	Northwest	Entertainment	--	LA	350	400	87.5%	4
Huang HT, 2012 [103]	2011/04-2011/07	Gu Yuan	Ningxia	Northwest	Entertainment	--	P1M	351	400	87.8%	4
Liu DF, 2012 [216]	2011	Yan An	Shaanxi	Northwest	Entertainment	--	P1M	253	476	53.2%	4
Fu SG, 2010 [27]	2000	--	Guangdong	South Central	Sentinel sites	--	P1M	1824	4613	39.5%	5
Qu S, 2002 [217]	2000/10-2000/12	Baise City	Guangxi	South Central	Entertainment	--	P1M	70	352	19.9%	4
Zhou YJ, 2006 [218]	2000	--	Guangxi	South Central	Entertainment	--	P1M	70	207	33.8%	4
Zhou YJ, 2008 [219]	2000	--	Guangxi	South Central	Entertainment	--	P1M	382	1198	31.9%	4

First author, published year	Study period	Location	Province	Region	Recruitment venue	Sampling method	Measurement period*	Number of FSW used condom	Total number of FSW	Condom Usage (%)	QA Score
Zhou YJ, 2008 [219]	2000	--	Guangxi	South Central	Entertainment	--	LA	548	1198	45.7%	4
Zhu QY, 2007 [220]	2000	--	Guangxi	South Central	VCT	--	P1M	224	608	36.8%	3
Zhu QY, 2007 [220]	2000	--	Guangxi	South Central	VCT	--	LA	547	879	62.2%	3
Fu SG, 2010 [27]	2001	--	Guangdong	South Central	Sentinel sites	--	P1M	1627	4357	37.3%	5
Zhnagdan C, 2008 [221]	2001/10	Wuhan	Hubei	South Central	Entertainment	--	LA	102	170	60.0%	3
Zhou YJ, 2005 [222]	2001/06	Beihai	Guangxi	South Central	Entertainment	--	LA	149	207	72.0%	3
Fu SG, 2010 [27]	2002	--	Guangdong	South Central	Sentinel sites	--	P1M	1127	4018	28.0%	5
Lu WJ, 2009 [223]	2002	Guangxi	Guangxi	South Central	Detention Center	Continuous sampling	LA	31	60	51.7%	3
Wang Y, 2004 [224]	2002/05-2002/06	--	Guangdong	South Central	Mixed venues	--	LA	87	121	71.9%	4
Fu SG, 2010 [27]	2003	--	Guangdong	South Central	Sentinel sites	--	P1M	1212	3498	34.6%	5
Li MQ, 2005 [225]	2003/06	Liuzhou	Guangxi	South Central	Entertainment	Random sampling	LA	146	227	64.3%	4
Li N, 2007 [226]	2003	Henan	Henan	South Central	Sentinel sites	Continuous sampling	LA	503	856	58.8%	3
Li N, 2007 [226]	2003	Henan	Henan	South Central	Sentinel sites	Continuous sampling	LA	511	957	53.4%	3
Li P, 2004 [227]	2003/01	Guangdong	Guangdong	South Central	--	--	LA	73	121	60.3%	4
Li P, 2006 [228]	2003/06-2003/12	Guangdong	Guangdong	South Central	--	--	P1M	171	201	85.1%	4

First author, published year	Study period	Location	Province	Region	Recruitment venue	Sampling method	Measurement period*	Number of FSW used condom	Total number of FSW	Condom Usage (%)	QA Score
Li P, 2006 [228]	2003/06-2003/12	Guangdong	Guangdong	South Central	--	--	LA	187	195	95.9%	4
Lu WJ, 2009 [223]	2003	Guangxi	Guangxi	South Central	Detention Center	Continuous sampling	LA	97	146	66.4%	3
Luo J, 2005 [229]	2003/06	Guangxi	Guangxi	South Central	Entertainment	--	LA	104	126	82.5%	4
Luo J, 2005 [229]	2003/06	Guangxi	Guangxi	South Central	Entertainment	--	LA	92	152	60.5%	4
Zheng ZJ, 2004 [230]	2003/01	Jingzhou	Hubei	South Central	Entertainment	--	LA	9	58	15.5%	3
Cao GH, 2009 [231]	2003/03-2004/09	Cai Country	Henan	South Central	--	Convenience sampling	P1M	127	150	84.7%	3
Cao GH, 2009 [231]	2003/03-2004/09	Cai Country	Henan	South Central	--	Convenience sampling	LA	130	150	86.7%	3
Fu SG, 2010 [27]	2004	--	Guangdong	South Central	Sentinel sites	--	P1M	508	4202	12.1%	5
Guo HY, 2008 [11]	2004/09	Xiangfan	Hubei	South Central	Entertainment	Random sampling	LA	77	105	73.3%	4
Guo HY, 2008 [11]	2004/09	Zhangjiajie	Hunan	South Central	Entertainment	Random sampling	LA	64	97	66.0%	4
Jiang M, 2005 [232]	2004/08-2004/10	Jingzhou	Hubei	South Central	Entertainment	Random sampling	LA	154	211	73.0%	4
Li N, 2007 [226]	2004	Henan	Henan	South Central	Sentinel sites	Continuous sampling	LA	570	984	57.9%	3
Li N, 2007 [226]	2004	Henan	Henan	South Central	Sentinel sites	Continuous sampling	LA	492	728	67.6%	3
Liu Y, 2008 [233]	2004	Shenzhen	Guangdong	South Central	Entertainment	Two-stage random sampling	P1M	20	56	35.7%	7
Lu WJ, 2009 [223]	2004	Guangxi	Guangxi	South Central	Detention Center	Continuous sampling	LA	124	253	49.0%	3

First author, published year	Study period	Location	Province	Region	Recruitment venue	Sampling method	Measurement period*	Number of FSW used condom	Total number of FSW	Condom Usage (%)	QA Score
Luo J, 2005 [234]	2004/11	Liuzhou	Guangxi	South Central	Entertainment	--	LA	247	325	76.0%	6
Sun CX, 2009 [235]	2004/07	Shiyan	Hubei	South Central	Entertainment	--	LA	44	59	74.6%	3
Sun CX, 2009 [235]	2004/07	Shiyan	Hubei	South Central	Entertainment	--	P1M	27	59	45.8%	3
Sun LM, 2005 [236]	2004/06-2004/08	--	Hubei	South Central	Entertainment	--	LA	85	113	75.2%	3
Tang GB, 2007 [237]	2004	Hecheng	Hunan	South Central	Entertainment	--	LA	130	397	32.7%	4
Wang TM, 2009 [238]	2004	Daye	Hubei	South Central	Entertainment	--	LA	330	364	90.7%	3
Wang TM, 2009 [238]	2004	Daye	Hubei	South Central	Entertainment	--	P1M	210	367	57.2%	3
Xiong CB, 2006 [239]	2004	Changde	Hunan	South Central	Entertainment	--	LA	48	136	35.3%	4
Yang BF, 2006 [240]	2004/08-2004/11	Wuhan	Hubei	South Central	Entertainment	--	LA	529	774	68.3%	3
Yang BF, 2006 [241]	2004/09	--	Hubei	South Central	Entertainment	--	LA	359	577	62.2%	2
Yang BF, 2006 [241]	2004/09	--	Hubei	South Central	Entertainment	--	P1M	180	577	31.2%	2
Yang BF, 2006 [242]	2004/08-2004/11	--	Hubei	South Central	Entertainment	--	LA	614	937	65.5%	2
Yang BF, 2006 [240]	2004/08-2004/11	Wuhan	Hubei	South Central	Entertainment	--	P1M	288	774	37.2%	3
Yang BF, 2006 [242]	2004/08-2004/11	--	Hubei	South Central	Entertainment	--	P1M	326	937	34.8%	2
Yang F, 2006 [243]	2004	Taishan, yangdong, doumen	Guangdong	South Central	--	Convenience sampling	LA	219	307	71.3%	3

First author, published year	Study period	Location	Province	Region	Recruitment venue	Sampling method	Measurement period*	Number of FSW used condom	Total number of FSW	Condom Usage (%)	QA Score
Yang F, 2006 [243]	2004	Taishan	Guangdong	South Central	--	Convenience sampling	LA	96	121	79.3%	3
Yang F, 2006 [243]	2004	Yangdong	Guangdong	South Central	--	Convenience sampling	LA	87	110	79.1%	3
Yang F, 2006 [243]	2004	Doumen	Guangdong	South Central	--	Convenience sampling	LA	35	76	46.1%	3
Yu DN, 2007 [244]	2004	Taishan	Guangdong	South Central	--	Convenience sampling	LA	110	140	78.6%	4
Zhou YJ, 2007 [245]	2004/05	-	Guangxi	South Central	Entertainment	--	LA	13	80	16.3%	6
Cao XL, 2011 [246]	2005	NanYang	Henan	South Central	Entertainment	--	LA	54	291	18.6%	3
Li N, 2007 [226]	2005	Henan	Henan	South Central	Sentinel sites	Continuous sampling	LA	1160	2535	45.8%	3
Li WJ, 2007 [247]	2005/11	Yangjiang	Guangdong	South Central	Entertainment	Cluster random sampling	LA	250	278	89.9%	6
Li WJ, 2007 [248]	2005/09	Yangjiang	Guangdong	South Central	Entertainment	Convenience sampling	P1M	168	278	60.4%	6
Liu J, 2011 [249]	2005	Heng yang	Hunan	South Central	Sentinel sites	--	LA	235	400	58.8%	1
Long CW, 2008 [250]	2005/05-2005/06	Huangshi	Hubei	South Central	Entertainment	Random sampling	LA	131	172	76.2%	4
Long CW, 2008 [250]	2005/05-2005/06	Huangshi	Hubei	South Central	Entertainment	Random sampling	P1M	111	172	64.5%	4
Lu F, 2009 [54]	2005	Liuzhou	Guangxi	South Central	--	Snowball sampling	LA	240	319	75.2%	3
Lu F, 2009 [54]	2005	Liuzhou	Guangxi	South Central	--	Snowball sampling	P1M	202	323	62.5%	3
Lu WJ, 2009 [223]	2005	Guangxi	Guangxi	South Central	Detention Center	Continuous sampling	LA	157	318	49.4%	3

First author, published year	Study period	Location	Province	Region	Recruitment venue	Sampling method	Measurement period*	Number of FSW used condom	Total number of FSW	Condom Usage (%)	QA Score
Shen CS, 2009 [251]	2005/08	Shenzhen	Guangdong	South Central	Entertainment	Random sampling	LA	197	277	71.1%	4
Xu YF, 2007 [252]	2005/07-2005/09	Nanning	Guangxi	South Central	Entertainment	Random sampling	P1M	74	192	38.5%	7
Yan LM, 2007 [253]	2005/03-2005/07	Xiaogan	Hubei	South Central	Entertainment	Random sampling	LA	62	182	34.1%	2
Zhang GS, 2009 [254]	2005/05-2005/10	Shantou	Guangdong	South Central	Entertainment	--	LA	281	461	61.0%	3
Zhang GS, 2009 [254]	2005/05-2005/10	Shantou	Guangdong	South Central	Entertainment	--	P1M	146	512	28.5%	3
Zhang WS, 2006 [255]	2004/10-2005/04	Xiangfan	Hubei	South Central	Entertainment	--	LA	274	325	84.3%	4
Zhou YJ, 2006 [218]	2005	--	Guangxi	South Central	Entertainment	--	P1M	110	203	54.2%	4
Cao XL, 2011 [246]	2006	NanYang	Henan	South Central	Entertainment	--	LA	108	294	36.7%	3
Li N, 2007 [226]	2006	Henan	Henan	South Central	Sentinel sites	Continuous sampling	LA	3086	5130	60.2%	3
Liu J, 2011 [249]	2006	Heng Yang	Hunan	South Central	Sentinel sites	--	LA	240	400	60.0%	1
Lu WJ, 2009 [223]	2006	Guangxi	Guangxi	South Central	Detention Center	Continuous sampling	LA	686	1423	48.2%	3
Pan XL, 2008 [256]	2005/11-2006/12	Baise	Guangxi	South Central	Entertainment	Random sampling	P1M	70	104	67.3%	5
Pan XL, 2008 [256]	2005/11-2006/12	Baise	Guangxi	South Central	Entertainment	Random sampling	P1M	63	114	55.3%	5
Pan XL, 2008 [256]	2005/11-2006/12	Baise	Guangxi	South Central	Entertainment	Random sampling	P1M	10	35	28.6%	5
Pan XL, 2009 [257]	2006/04-2006/06	Baise	Guangxi	South Central	Entertainment	Cluster sampling	LA	225	253	88.9%	4

First author, published year	Study period	Location	Province	Region	Recruitment venue	Sampling method	Measurement period*	Number of FSW used condom	Total number of FSW	Condom Usage (%)	QA Score
Pan XL, 2009 [257]	2006.04 - 2006.06	Baise	Guangxi	South Central	Entertainment	Cluster sampling	P1M	143	253	56.5%	4
Wang CJ, 2010 [258]	2006	--	Henan	South Central	Entertainment	--	LA	1710	2351	72.7%	4
Wang TM, 2009 [238]	2006	Daye	Hubei	South Central	Entertainment	--	LA	364	437	83.3%	3
Wang TM, 2009 [238]	2006	Daye	Hubei	South Central	Entertainment	--	P1M	240	437	54.9%	3
Wang ZQ, 2010 [259]	2006/10-2006/11	Changjiang	Hainan	South Central	Entertainment	--	LA	268	378	70.9%	3
Wang ZQ, 2010 [259]	2006/10 - 2006/11	Changjiang	Hainan	South Central	Entertainment	--	P1M	104	378	27.5%	3
Xu XY, 2007 [260]	2006/04-2006/06	Guangzhou	Guangdong	South Central	Entertainment	--	LA	242	355	68.2%	3
Cao XL, 2011 [246]	2007	Nanyang	Henan	South Central	Entertainment	--	LA	177	361	49.0%	3
Chen Y, 2010 [261]	2006/12-2007/02	--	Guangxi	South Central	Entertainment	Stratified cluster sampling, convenience sampling	P1M	84	133	63.2%	2
Cheng ZQ, 2008 [262]	2006/07-2007/12	Nanning	Guangxi	South Central	Entertainment	--	P1M	279	879	31.7%	3
He Y, 2009 [263]	2007/07	Nanning	Guangxi	South Central	Entertainment	Random sampling	P1M	59	206	28.6%	5
He Y, 2009 [263]	2007/07	Nanning	Guangxi	South Central	Entertainment	Random sampling	LA	105	206	51.0%	5
He Y, 2009 [263]	2007/07	Nanning	Guangxi	South Central	Entertainment	Random sampling	P1M	59	206	28.6%	5

First author, published year	Study period	Location	Province	Region	Recruitment venue	Sampling method	Measurement period*	Number of FSW used condom	Total number of FSW	Condom Usage (%)	QA Score
Jiang M, 2012 [264]	2007/07-2007/10	Jing zhou	Hubei	South Central	Entertainment	--	LA	154	211	73.0%	4
Jiang M, 2012 [264]	2007/07-2007/10	Jing zhou	Hubei	South Central	Entertainment	--	P1M	72	211	34.1%	4
Li XF, 2008 [11]	2007/01-2007/03	Chenzhou	Hunan	South Central	Entertainment	Random sampling	LA	221	235	94.0%	4
Li Y, 2009 [265]	2006/08-2007/01	Guangdong	Guangdong	South Central	Entertainment	Respondent-Driven Sampling	LA	299	320	93.4%	6
Liu C, 2011 [266]	2007	Wu han	Hubei	South Central	Entertainment	--	LA	412	444	92.8%	3
Liu C, 2011 [266]	2007	Wu han	Hubei	South Central	Entertainment	--	P1M	326	444	73.4%	3
Liu J, 2011 [249]	2007	Heng yang	Hunan	South Central	Sentinel sites	--	LA	220	385	57.1%	1
Lu WJ, 2008 [267]	2007	20 cities/counties of Guangxi	Guangxi	South Central	Entertainment	--	LA	2408	3344	72.0%	6
Lu WJ, 2008 [267]	2007	20 cities/counties of Guangxi	Guangxi	South Central	Entertainment	--	P1M	1338	3344	40.0%	6
Lu WJ, 2009 [223]	2007	Guangxi	Guangxi	South Central	Detention Center	Continuous sampling	LA	781	1549	50.4%	3
Nong CM, 2008 [268]	2007/07-2007/10	Congzuo	Guangxi	South Central	Entertainment	Census	LA	75	95	78.9%	5
Nong CM, 2008 [268]	2007/07-2007/10	Congzuo	Guangxi	South Central	Entertainment	Census	P1M	70	95	73.7%	5
Nong CM, 2008 [268]	2007/07-2007/10	Congzuo	Guangxi	South Central	Entertainment	Census	LA	210	311	67.5%	5
Nong CM, 2008 [268]	2007/07-2007/10	Congzuo	Guangxi	South Central	Entertainment	Census	P1M	160	311	51.4%	5

First author, published year	Study period	Location	Province	Region	Recruitment venue	Sampling method	Measurement period*	Number of FSW used condom	Total number of FSW	Condom Usage (%)	QA Score
Pan XL, 2009 [257]	2007/04-2007/06	Baise	Guangxi	South Central	Entertainment	Cluster sampling	LA	199	300	66.3%	4
Pan XL, 2009 [257]	2007/04-2007/06	Baise	Guangxi	South Central	Entertainment	Cluster sampling	P1M	144	300	48.0%	4
Qiu JJ, 2008 [269]	2007/01-2007/12	Shenzhen	Guangdong	South Central	Entertainment	--	LA	313	413	75.8%	3
Shi XH, 2008 [270]	2004-2007	Shenzhen	Guangdong	South Central	Entertainment	Random and systematic sampling	P1M	83	232	35.8%	3
Tan SN, 2010 [271]	2007/08-2007/11	Laibin	Guangxi	South Central	Entertainment	Stratified cluster sampling	LA	117	246	47.6%	5
Tan SN, 2010 [271]	2007/08-2007/11	Laibin	Guangxi	South Central	Entertainment	Stratified cluster sampling	P1M	82	246	33.3%	5
Tan WW, 2008 [272]	2007/07-2007/09	Nanning	Guangxi	South Central	Entertainment	--	LA	277	379	73.1%	4
Tan WW, 2008 [272]	2007/07-2007/09	Nanning	Guangxi	South Central	Entertainment	--	P1M	131	379	34.6%	4
Tan WW, 2011 [273]	2007/04-2007/06	Nanning	Guangxi	South Central	Entertainment	--	P1M	131	379	34.6%	4
Tan WW, 2011 [273]	2007/04-2007/06	Nanning	Guangxi	South Central	Entertainment	--	LA	323	400	80.8%	4
Tan WW, 2011 [273]	2007/04-2007/06	Nanning	Guangxi	South Central	Entertainment	--	P1M	230	400	57.5%	4
Tan WW, 2011 [273]	2007/04-2007/06	Nanning	Guangxi	South Central	Entertainment	--	LA	304	400	76.0%	4
Tan WW, 2011 [273]	2007/04-2007/06	Nanning	Guangxi	South Central	Entertainment	--	P1M	207	400	51.8%	4
Tang MJ, 2008 [274]	2007/04-2007/06	Yulin	Guangxi	South Central	Entertainment	--	LA	307	362	84.8%	4

First author, published year	Study period	Location	Province	Region	Recruitment venue	Sampling method	Measurement period*	Number of FSW used condom	Total number of FSW	Condom Usage (%)	QA Score
Wang QQ, 2009 [275]	2007/01-2007/12	--	Guangdong, Hainan	South Central	Entertainment	Stratified cluster sampling	LA	236	356	66.3%	3
Wang QQ, 2009 [275]	2007/01-2007/12	--	Guangdong, Hainan	South Central	Entertainment	Stratified cluster sampling	LA	99	138	71.7%	3
Wang QQ, 2009 [275]	2007/01-2007/12	--	Guangdong, Hainan	South Central	Entertainment	Stratified cluster sampling	P1M	1	356	0.3%	3
Wang QQ, 2009 [275]	2007/01-2007/12	--	Guangdong, Hainan	South Central	Entertainment	Stratified cluster sampling	P1M	2	138	1.4%	3
Wang QQ, 2009 [275]	2007/01-2007/12	--	Guangdong, Hainan	South Central	Entertainment	Stratified cluster sampling	LA	270	322	83.9%	3
Wang QQ, 2009 [275]	2007/01-2007/12	--	Guangdong, Hainan	South Central	Entertainment	Stratified cluster sampling	P1M	48	322	14.9%	3
Wang TM, 2009 [238]	2007	Daye	Hubei	South Central	Entertainment	--	LA	217	247	87.9%	3
Wang TM, 2009 [238]	2007	Daye	Hubei	South Central	Entertainment	--	P1M	208	247	84.2%	3
Wen XQ, 2009 [276]	2007/04-2007/05	guilin	Guangxi	South Central	Entertainment	Stratified cluster and random sampling	LA	258	360	71.7%	3
Wen XQ, 2009 [276]	2007/04-2007/05	guilin	Guangxi	South Central	Entertainment	Stratified cluster and random sampling	P1M	143	360	39.7%	3

First author, published year	Study period	Location	Province	Region	Recruitment venue	Sampling method	Measurement period*	Number of FSW used condom	Total number of FSW	Condom Usage (%)	QA Score
Wen XQ, 2012 [277]	2007	Gui Lin	Guangxi	South Central	Entertainment	--	LA	258	358	72.1%	4
Wen XQ, 2012 [277]	2007	Gui Lin	Guangxi	South Central	Entertainment	--	P1M	143	359	39.8%	4
Xu YF, 2008 [278]	2007/08-2007/09	Nanning	Guangxi	South Central	Entertainment	Random sampling	P1M	131	356	36.8%	4
Xu YF, 2009 [279]	2007	Nanning	Guangxi	South Central	Entertainment	Random sampling	LA	277	379	73.1%	7
Xu YF, 2009 [279]	2007	Nanning	Guangxi	South Central	Entertainment	Random sampling	P1M	131	357	36.7%	7
Yao ZZ, 2009 [280]	2007/02-2007/06	--	Hubei	South Central	Entertainment	Random sampling	LA	189	192	98.4%	4
Yao ZZ, 2009 [281]	2007/02-2007/06	--	Hubei	South Central	Entertainment	Random sampling	P1M	148	192	77.1%	4
Zhang SX, 2008 [282]	2007/12	Shangcai country	Henan	South Central	--	Convenience sampling	LA	130	150	86.7%	3
Zhang SX, 2008 [282]	2007/12	Shangcai country	Henan	South Central	--	Convenience sampling	P1M	127	150	84.7%	3
Zhang YX, 2011 [283]	2007/07-2007/09	Liu Zhou	Guangxi	South Central	Entertainment	--	LA	72	86	83.7%	4
Zhang YX, 2011 [283]	2007/07-2007/09	Liu Zhou	Guangxi	South Central	Entertainment	--	LA	224	315	71.1%	4
Zhang YX, 2011 [283]	2007/07-2007/09	Liu Zhou	Guangxi	South Central	Entertainment	--	LA	296	401	73.8%	4
Zhang YX, 2011 [283]	2007/07-2007/09	Liu Zhou	Guangxi	South Central	Entertainment	--	P1M	225	401	56.1%	4
Zhang YX, 2011 [283]	2007/07-2007/09	Liu Zhou	Guangxi	South Central	Entertainment	--	P1M	182	315	57.8%	4
Zhang YX, 2011 [283]	2007/07-2007/09	Liu Zhou	Guangxi	South Central	Entertainment	--	P1M	43	86	50.0%	4

First author, published year	Study period	Location	Province	Region	Recruitment venue	Sampling method	Measurement period*	Number of FSW used condom	Total number of FSW	Condom Usage (%)	QA Score
Zheng J, 2011 [284]	2007	Shigu	Hunan	South Central	--	--	LA	133	360	36.9%	3
Zheng J, 2011 [284]	2007	Hengnan	Hunan	South Central	--	--	LA	166	360	46.1%	3
Zheng J, 2011 [284]	2007	Moyang	Hunan	South Central	--	--	LA	223	322	69.3%	3
Zheng J, 2011 [284]	2007	Shigu, Hengnan, Moyang	Hunan	South Central	--	--	LA	498	1042	47.8%	3
Zheng J, 2011 [284]	2007	Shigu, Hengnan, Moyang	Hunan	South Central	--	--	P1M	269	1042	25.8%	3
Zheng J, 2011 [284]	2007	Shigu	Hunan	South Central	--	--	P1M	109	360	30.3%	3
Zheng J, 2011 [284]	2007	Hengnan	Hunan	South Central	--	--	P1M	94	360	26.1%	3
Zheng J, 2011 [284]	2007	Moyang	Hunan	South Central	--	--	P1M	66	322	20.5%	3
Zhou JH, 2010 [285]	2007/07-2007/09	Shenzhen	Guangdong	South Central	Entertainment	--	LA	311	418	74.4%	4
Zhou JH, 2010 [285]	2007/07-2007/09	Shenzhen	Guangdong	South Central	Entertainment	--	P1M	280	418	67.0%	4
Zhou YJ, 2008 [286]	2007/04-2007/05	--	Guangxi	South Central	--	--	LA	82	105	78.1%	7
Zhou YJ, 2008 [286]	2007/04-2007/05	--	Guangxi	South Central	--	--	P1M	38	105	36.2%	7
Zhou YJ, 2008 [219]	2007	--	Guangxi	South Central	Entertainment	--	P1M	1254	2232	56.2%	4
Zhou YJ, 2008 [219]	2007	--	Guangxi	South Central	Entertainment	--	LA	1703	2232	76.3%	4

First author, published year	Study period	Location	Province	Region	Recruitment venue	Sampling method	Measurement period*	Number of FSW used condom	Total number of FSW	Condom Usage (%)	QA Score
Bai Y, 2009 [287]	2008/04-2008/07	Liuzhou	Guangxi	South Central	Entertainment	Random sampling	LA	352	431	81.7%	6
Bai Y, 2009 [287]	2008/04-2008/07	Liuzhou	Guangxi	South Central	Entertainment	Random sampling	LA	385	449	85.7%	6
Bai Y, 2009 [287]	2008/04-2008/07	Liuzhou	Guangxi	South Central	Entertainment	Random sampling	LA	104	167	62.3%	6
BaiY, 2010 [288]	2008/04-2008/07	Liuzhou	Guangxi	South Central	--	Continuous sampling	LA	370	448	82.6%	5
BaiY, 2010 [288]	2008/04-2008/07	Liuzhou	Guangxi	South Central	--	Continuous sampling	P1M	344	448	76.8%	5
Cao XL, 2011 [246]	2008	Nanyang	Henan	South Central	Entertainment	--	LA	306	376	81.4%	3
He Y, 2009 [263]	2007/07	Nanning	Guangxi	South Central	Entertainment	Random sampling	LA	105	206	51.0%	5
Liao J, 2010 [289]	2008/11-2008/12	Gucheng	Hubei	South Central	--	Census	LA	93	93	100.0%	3
Liao J, 2010 [289]	2008/11-2008/12	Gucheng	Hubei	South Central	--	Census	P1M	93	93	100.0%	3
Lin MH, 2008 [290]	2007/08-2008/05	Baise	Guangxi	South Central	Mixed venues	--	LA	74	236	31.4%	5
Liu J, 2011 [249]	2008	Heng yang	Hunan	South Central	Sentinel sites	--	LA	326	391	83.4%	1
Mao AL, 2010 [291]	2008/09-2008/10	Jingzhou	Hubei	South Central	Entertainment	--	LA	152	288	52.8%	4
Mao AL, 2010 [291]	2008/09-2008/10	Jingzhou	Hubei	South Central	Entertainment	--	P1M	100	288	34.7%	4
Pan XL, 2009 [257]	2008/04-2008/06	Baise	Guangxi	South Central	Entertainment	Cluster sampling	LA	264	311	84.9%	4
Pan XL, 2009 [257]	2008/04-2008/06	Baise	Guangxi	South Central	Entertainment	Cluster sampling	P1M	186	311	59.8%	4

First author, published year	Study period	Location	Province	Region	Recruitment venue	Sampling method	Measurement period*	Number of FSW used condom	Total number of FSW	Condom Usage (%)	QA Score
Tan JG, 2009 [292]	2008/04-2008/05	Shenzhen	Guangdong	South Central	Entertainment	Stratified cluster sampling	LA	236	266	88.7%	5
Tan JG, 2009 [292]	2008/04-2008/05	Shenzhen	Guangdong	South Central	Entertainment	Stratified cluster sampling	P1M	202	266	75.9%	5
Wang TM, 2009 [238]	2008	Daye	Hubei	South Central	Entertainment	--	LA	307	347	88.5%	3
Wang TM, 2009 [238]	2008	Daye	Hubei	South Central	Entertainment	--	P1M	270	347	77.8%	3
Wang WM, 2010 [293]	2008/04	--	Henan	South Central	Entertainment	--	LA	104	110	94.5%	2
Wen XQ, 2012 [277]	2008	Gui lin	Guangxi	South Central	Entertainment	--	LA	323	380	85.0%	4
Wen XQ, 2012 [277]	2008	Gui lin	Guangxi	South Central	Entertainment	--	P1M	232	387	59.9%	4
Wen YQ, 2011 [294]	2008/04-2008/07	Liu zhou	Guangxi	South Central	Entertainment	--	LA	253	320	79.1%	5
Wen YQ, 2011 [294]	2008/04-2008/07	Liu Zhou	Guangxi	South Central	Entertainment	--	LA	577	712	81.0%	5
Wen YQ, 2011 [294]	2008/04-2008/07	Liu Zhou	Guangxi	South Central	Entertainment	--	P1M	364	712	51.1%	5
Wen YQ, 2011 [294]	2008/04-2008/07	Liu Zhou	Guangxi	South Central	Entertainment	--	P1M	229	320	71.6%	5
Wu DF, 2009 [295]	2004/09-2008/12	--	Hubei, Shangdong, Hebei, Shanxi, Henan, Shaanxi	South Central	Entertainment	--	P1M	6884	7913	87.0%	5
Xu YF, 2009 [279]	2008	Nanning	Guangxi	South Central	Entertainment	Random sampling	LA	323	400	80.8%	7

First author, published year	Study period	Location	Province	Region	Recruitment venue	Sampling method	Measurement period*	Number of FSW used condom	Total number of FSW	Condom Usage (%)	QA Score
Xu YF, 2009 [279]	2008	Nanning	Guangxi	South Central	Entertainment	Random sampling	P1M	230	398	57.8%	7
Yu DY, 2012 [296]	2008/07	Liu zhou	Guangxi	South Central	Entertainment	--	LA	199	213	93.4%	3
Zhang L, 2010 [297]	2008/12	Shangcai	Henan	South Central	Entertainment	--	LA	166	172	96.5%	3
Zhang L, 2010 [297]	2008/12	Shangcai	Henan	South Central	Entertainment	--	P1M	155	172	90.1%	3
Zhang YH, 2011 [96]	2008/04-2008/07	Haikou	Hainan	South Central	--	--	LA	202	404	50.0%	2
Zhang YH, 2011 [96]	2008/04-2008/07	Guangzhou	Guangdong	South Central	--	--	LA	259	289	89.6%	2
Zhang YH, 2011 [96]	2008/04-2008/07	Sanya	Hainan	South Central	--	--	LA	312	400	78.0%	2
Zhang YH, 2011 [96]	2008/04-2008/07	Wuhan	Hubei	South Central	--	--	LA	400	444	90.1%	2
Zhang YH, 2011 [96]	2008/04-2008/07	Guangzhou	Guangdong	South Central	--	--	P1M	252	289	87.2%	2
Zhang YH, 2011 [96]	2008/04-2008/07	Wuhan	Hubei	South Central	--	--	P1M	333	444	75.0%	2
Zhang YH, 2011 [96]	2008/04-2008/07	Sanya	Hainan	South Central	--	--	P1M	282	400	70.5%	2
Zhang YH, 2011 [96]	2008/04-2008/07	Haikou	Hainan	South Central	--	--	P1M	162	404	40.1%	2
Zhao YY, 2010 [298]	2008/10-2008/12	Guangzhou	Guangdong	South Central	Entertainment	--	LA	234	622	37.6%	4
Cao XL, 2011 [246]	2009	Nanyang	Henan	South Central	Entertainment	--	LA	323	378	85.4%	3
Chen L, 2010 [299]	2009	Shenzhen	Guangdong	South Central	Entertainment	Random sampling	LA	290	426	68.1%	2

First author, published year	Study period	Location	Province	Region	Recruitment venue	Sampling method	Measurement period*	Number of FSW used condom	Total number of FSW	Condom Usage (%)	QA Score
Dun ZJ, 2011 [300]	2007-2009	Guang zhou	Guangdong	South Central	Entertainment	--	LA	275	303	90.8%	3
Hu SX, 2010 [301]	2009/10-2009/12	Qingyuan	Guangdong	South Central	Entertainment	Random sampling	LA	351	380	92.4%	4
Huang ZX, 2011 [302]	2009/07-2009/09	Fang cheng gang	Guangxi	South Central	Entertainment	--	LA	142	203	70.0%	4
Huang ZX, 2011 [302]	2009/07-2009/09	Fang cheng gang	Guangxi	South Central	Entertainment	--	P1M	60	203	29.6%	4
Li WJ, 2008 [247]	2005/11	Yangjiang	Guangdong	South Central	Entertainment	Cluster random sampling	P1M	168	278	60.4%	6
Liao S, 2011 [303]	2008 - 2009	--	Hainan	South Central	Entertainment	Venue-based sampling	P1M	79	111	71.2%	4
Lin RL, 2012 [304]	2009	Yu Lin	Guangxi	South Central	Entertainment	Cluster sampling	LA	324	400	81.0%	4
Lin RL, 2012 [304]	2009	Yu Lin	Guangxi	South Central	Entertainment	Cluster sampling	P1M	179	400	44.8%	4
LING B, 2011 [305]	2009/04-2009/06	Zhou Kou	Henan	South Central	Entertainment	Community outreach	LA	386	421	91.7%	4
Liu J, 2011 [249]	2009	Heng Yang	Hunan	South Central	Sentinel sites	--	LA	343	380	90.3%	1
Luo MH, 2011 [306]	2009	Shao Yang	Hunan	South Central	Entertainment	--	LA	345	400	86.3%	2
Luo MH, 2011 [306]	2009	Shao Dong	Hunan	South Central	Entertainment	--	LA	384	400	96.0%	2
Luo MH, 2011 [306]	2009	Shao Dong	Hunan	South Central	Entertainment	--	P1M	321	400	80.3%	2
Luo MH, 2011 [306]	2009	Shao Yang	Hunan	South Central	Entertainment	--	P1M	295	400	73.8%	2
Nei ZQ, 2011 [307]	2009/04-2009/07	--	Guangdong	South Central	Mixed venues	Random sampling	P1M	3621	5309	68.2%	5

First author, published year	Study period	Location	Province	Region	Recruitment venue	Sampling method	Measurement period*	Number of FSW used condom	Total number of FSW	Condom Usage (%)	QA Score
Nei ZQ, 2011 [307]	2009/04-2009/07	--	Guangdong	South Central	Mixed venues	Random sampling	LA	3769	5309	71.0%	5
Wang JY, 2010 [308]	2009/05	Zhongshan	Guangdong	South Central	Community	Convenience sampling	LA	371	406	91.4%	4
Wang JY, 2010 [308]	2009/05	Zhongshan	Guangdong	South Central	Community	Convenience sampling	P1M	202	310	65.2%	4
Wen XQ, 2012 [277]	2009	Gui lin	Guangxi	South Central	Entertainment	--	LA	300	380	78.9%	4
Wen XQ, 2012 [277]	2009	Gui lin	Guangxi	South Central	Entertainment	--	P1M	255	374	68.2%	4
Wen XQ, 2010 [309]	2009/04-2009/05	Guilin	Guangxi	South Central	Entertainment	Stratified cluster sampling	P1M	225	400	56.3%	3
Xiang H, 2011 [310]	2009/04-2009/06	Lai feng	Hubei	South Central	Entertainment	--	LA	138	146	94.5%	4
Xiang H, 2011 [310]	2009/04-2009/06	Lai feng	Hubei	South Central	Entertainment	--	LA	322	400	80.5%	4
Xiang H, 2011 [310]	2009/04-2009/06	Lai feng	Hubei	South Central	Entertainment	--	LA	66	129	51.2%	4
Xiang H, 2011 [310]	2009/04-2009/06	Lai feng	Hubei	South Central	Entertainment	--	LA	118	135	87.4%	4
Xiang H, 2011 [310]	2009/04-2009/06	Lai feng	Hubei	South Central	Entertainment	--	P1M	254	400	63.5%	4
Xiang H, 2011 [310]	2009/04-2009/06	Lai feng	Hubei	South Central	Entertainment	--	P1M	131	146	89.7%	4
Xiang H, 2011 [310]	2009/04-2009/06	Lai feng	Hubei	South Central	Entertainment	--	P1M	91	135	67.4%	4
Xiang H, 2011 [310]	2009/04-2009/06	Lai feng	Hubei	South Central	Entertainment	--	P1M	32	129	24.8%	4
Xiang Z, 2012 [311]	2009/07-2009/09	Wuzhou, hezhou	Guangxi	South Central	Entertainment	Convenience sampling	P1M	483	810	59.6%	6

First author, published year	Study period	Location	Province	Region	Recruitment venue	Sampling method	Measurement period*	Number of FSW used condom	Total number of FSW	Condom Usage (%)	QA Score
Xiang Z, 2012 [311]	2009/07-2009/09	Wuzhou, Hezhou	Guangxi	South Central	Entertainment	Convenience sampling	LA	665	810	82.1%	6
Xu HF, 2011 [312]	2009/03-2009/04	Shang qiu	Henan	South Central	Entertainment	--	LA	135	200	67.5%	4
Xu HF, 2011 [312]	2009/07-2009/09	Shang qiu	Henan	South Central	Entertainment	--	LA	183	200	91.5%	4
Xu HF, 2011 [312]	2009/03-2009/04	Shang qiu	Henan	South Central	Entertainment	--	P1M	31	200	15.5%	4
Yu DY, 2012 [296]	2009/07	Liu Zhou	Guangxi	South Central	Entertainment	--	LA	295	303	97.4%	3
Zeng XL, 2011 [313]	2009/11	Nanyang	Henan	South Central	--	--	LA	354	457	77.5%	4
Zeng XL, 2011 [313]	2009/11	Nanyang	Henan	South Central	--	--	P1M	324	457	70.9%	4
Zhang L, 2011 [314]	2009	Xinyang	Henan	South Central	Entertainment	--	LA	391	400	97.8%	4
Zhang L, 2011 [314]	2009	Xinyang	Henan	South Central	Entertainment	--	P1M	307	400	76.8%	4
Zhao JZ, 2011 [315]	2009/07-2009/12	Sui Zhou	Hubei	South Central	Entertainment	--	P1M	142	198	71.7%	4
Zhong J, 2011 [316]	2009/06-2009/08	Wu zhou	Guangxi	South Central	Entertainment	--	LA	25	44	56.8%	4
Zhong J, 2011 [316]	2009/06-2009/08	Wu zhou	Guangxi	South Central	Entertainment	--	LA	824	1132	72.8%	4
Zhong J, 2011 [316]	2009/06-2009/08	Wu zhou	Guangxi	South Central	Entertainment	--	LA	382	465	82.2%	4
Zhong J, 2011 [316]	2009/06-2009/08	Wu zhou	Guangxi	South Central	Entertainment	--	LA	417	648	64.4%	4
Zhong J, 2011 [316]	2009/06-2009/08	Wu zhou	Guangxi	South Central	Entertainment	--	P1M	14	44	31.8%	4

First author, published year	Study period	Location	Province	Region	Recruitment venue	Sampling method	Measurement period*	Number of FSW used condom	Total number of FSW	Condom Usage (%)	QA Score
Zhong J, 2011 [316]	2009/06-2009/08	Wu zhou	Guangxi	South Central	Entertainment	--	P1M	282	629	44.8%	4
Zhong J, 2011 [316]	2009/06-2009/08	Wu zhou	Guangxi	South Central	Entertainment	--	P1M	153	463	33.0%	4
Zhong J, 2011 [316]	2009/06-2009/08	Wu zhou	Guangxi	South Central	Entertainment	--	P1M	449	1136	39.5%	4
Zhong J, 2011 [317]	2009/06-2009/08	Wuzhou	Guangxi	South Central	Entertainment	Random sampling	LA	25	44	56.8%	4
Zhong J, 2011 [317]	2009/06-2009/08	Wuzhou	Guangxi	South Central	Entertainment	Random sampling	P1M	14	44	31.8%	4
Zhong J, 2011 [317]	2009/06-2009/08	Wuzhou	Guangxi	South Central	Entertainment	Random sampling	LA	417	648	64.4%	4
Zhong J, 2011 [317]	2009/06-2009/08	Wuzhou	Guangxi	South Central	Entertainment	Random sampling	P1M	282	629	44.8%	4
Zhong J, 2011 [317]	2009/06-2009/08	Wuzhou	Guangxi	South Central	Entertainment	Random sampling	LA	382	465	82.2%	4
Zhong J, 2011 [317]	2009/06-2009/08	Wuzhou	Guangxi	South Central	Entertainment	Random sampling	P1M	153	463	33.0%	4
Zhou JL, 2010 [318]	2009	Linzhou	Hunan	South Central	Entertainment	Random sampling	LA	60	60	100.0%	6
Zhou JL, 2010 [318]	2009	Linzhou	Hunan	South Central	Entertainment	Random sampling	P1M	59	60	98.3%	6
Zhou JL, 2010 [318]	2009	Linzhou	Hunan	South Central	Entertainment	Random sampling	LA	112	119	94.1%	6
Zhou JL, 2010 [318]	2009	Linzhou	Hunan	South Central	Entertainment	Random sampling	P1M	101	119	84.9%	6
Zhou JL, 2010 [318]	2009	Linzhou	Hunan	South Central	Entertainment	Random sampling	LA	184	223	82.5%	6
Zhou JL, 2010 [318]	2009	Linzhou	Hunan	South Central	Entertainment	Random sampling	P1M	129	223	57.8%	6

First author, published year	Study period	Location	Province	Region	Recruitment venue	Sampling method	Measurement period*	Number of FSW used condom	Total number of FSW	Condom Usage (%)	QA Score
Zhou_XL, 2010 [319]	2008/05-2009/05	Guangzhou	Guangdong	South Central	Entertainment	Convenience sampling	LA	268	303	88.4%	5
Bai Y, 2012 [320]	2010/05-2010/07	Liu Zhou	Guangxi	South Central	--	--	LA	1552	1846	84.1%	4
Bai Y, 2012 [320]	2010/05-2010/07	Liu Zhou	Guangxi	South Central	--	--	P1M	1270	1845	68.8%	4
Chen FC, 2011 [321]	2010	Dang Yang	Hubei	South Central	Entertainment	--	LA	36	36	100.0%	3
Chen FC, 2011 [321]	2010	Dang Yang	Hubei	South Central	Entertainment	--	LA	396	400	99.0%	3
Chen FC, 2011 [321]	2010	Dang Yang	Hubei	South Central	Entertainment	--	LA	74	77	96.1%	3
Chen FC, 2011 [321]	2010	Dang Yang	Hubei	South Central	Entertainment	--	LA	286	287	99.7%	3
Chen FC, 2011 [321]	2010	Dang Yang	Hubei	South Central	Entertainment	--	P1M	360	400	90.0%	3
Chen FC, 2011 [321]	2010	Dang Yang	Hubei	South Central	Entertainment	--	P1M	36	36	100.0%	3
Chen FC, 2011 [321]	2010	Dang Yang	Hubei	South Central	Entertainment	--	P1M	271	287	94.4%	3
Chen FC, 2011 [321]	2010	Dang Yang	Hubei	South Central	Entertainment	--	P1M	53	77	68.8%	3
Chen ZB, 2011 [322]	2010/04-2010/07	Lian Zhou	Guangdong	South Central	Entertainment	--	LA	129	180	71.7%	3
Chen ZB, 2011 [322]	2010/04-2010/07	Lian Zhou	Guangdong	South Central	Entertainment	--	LA	107	157	68.2%	3
Chen ZB, 2011 [322]	2010/04-2010/07	Lian Zhou	Guangdong	South Central	Entertainment	--	P1M	79	180	43.9%	3
Chen ZB, 2011 [322]	2010/04-2010/07	Lian Zhou	Guangdong	South Central	Entertainment	--	P1M	76	157	48.4%	3

First author, published year	Study period	Location	Province	Region	Recruitment venue	Sampling method	Measurement period*	Number of FSW used condom	Total number of FSW	Condom Usage (%)	QA Score
Gui Q, 2012 [323]	2010/04-2010/07	Chang De	Hunan	South Central	Entertainment	--	LA	375	400	93.8%	4
Gui Q, 2012 [323]	2010/04-2010/07	Chang De	Hunan	South Central	Entertainment	--	P1M	310	400	77.5%	4
He B, 2011 [324]	2010/03-2010/08	Qiong Hai	Hainan	South Central	Entertainment	--	P1M	37	75	49.3%	3
Huang KZ, 2010 [325]	2010	Yangjiang	Guangdong	South Central	Entertainment	Random sampling	LA	329	391	84.1%	4
Huang KZ, 2010 [325]	2010	Yangjiang	Guangdong	South Central	Entertainment	Random sampling	P1M	219	391	56.0%	4
Jiang N, 2012 [326]	2009-2010	Zhu Ma Dian	Henan	South Central	--	--	LA	288	475	60.6%	3
Liao S, 2011 [303]	2009 - 2010	--	Guangxi	South Central	Entertainment	Venue-based sampling	P1M	84	109	77.1%	4
Lin RL, 2012 [304]	2010	Yu Lin	Guangxi	South Central	Entertainment	Cluster sampling	LA	380	400	95.0%	4
Lin RL, 2012 [304]	2010	Yu Lin	Guangxi	South Central	Entertainment	Cluster sampling	P1M	251	400	62.8%	4
Nong LP, 2011 [327]	2010/04-2010/05	Ping Xiang	Guangxi	South Central	Entertainment	Random clustered sampling	LA	28	31	90.3%	3
Nong LP, 2011 [327]	2010/04-2010/05	Ping Xiang	Guangxi	South Central	Entertainment	Random clustered sampling	LA	209	262	79.8%	3
Nong LP, 2011 [327]	2010/04-2010/05	Ping Xiang	Guangxi	South Central	Entertainment	Random clustered sampling	LA	44	57	77.2%	3
Nong LP, 2011 [327]	2010/04-2010/05	Ping Xiang	Guangxi	South Central	Entertainment	Random clustered sampling	LA	139	174	79.9%	3

First author, published year	Study period	Location	Province	Region	Recruitment venue	Sampling method	Measurement period*	Number of FSW used condom	Total number of FSW	Condom Usage (%)	QA Score
Nong LP, 2011 [327]	2010/04-2010/05	Ping Xiang	Guangxi	South Central	Entertainment	Random clustered sampling	P1M	155	262	59.2%	3
Nong LP, 2011 [327]	2010/04-2010/05	Ping Xiang	Guangxi	South Central	Entertainment	Random clustered sampling	P1M	26	31	83.9%	3
Nong LP, 2011 [327]	2010/04-2010/05	Ping Xiang	Guangxi	South Central	Entertainment	Random clustered sampling	P1M	107	174	61.5%	3
Nong LP, 2011 [327]	2010/04-2010/05	Ping Xiang	Guangxi	South Central	Entertainment	Random clustered sampling	P1M	22	57	38.6%	3
Wen XQ, 2012 [277]	2010	Gui Lin	Guangxi	South Central	Entertainment	--	LA	355	398	89.2%	4
Wen XQ, 2012 [277]	2010	Gui Lin	Guangxi	South Central	Entertainment	--	P1M	321	398	80.7%	4
Xiong CS, 2012 [138]	2010	Shi Yan	Hubei	South Central	Entertainment	Two-stage sampling	LA	356	400	89.0%	4
Zeng XL, 2011 [313]	2010/07	Nanyang	Henan	South Central	Entertainment	--	LA	323	378	85.4%	4
Zhu L, 2011 [328]	2010/04-2010/06	Xiang Yang	Hubei	South Central	Sentinel sites	--	LA	402	402	100.0%	4
Zhu L, 2011 [328]	2010/04-2010/06	Xiang Yang	Hubei	South Central	Sentinel sites	--	P1M	402	402	100.0%	4
Ke XZ, 2012 [329]	2011/04-2011/07	Huang Shi	Hubei	South Central	--	--	LA	350	400	87.5%	3
Ke XZ, 2012 [329]	2011/04-2011/07	Huang Shi	Hubei	South Central	--	--	P1M	242	400	60.5%	3
Li Y, 2012 [192]	2010-2011	Xiao Gan	Hubei	South Central	Entertainment	--	LA	380	400	95.0%	4

First author, published year	Study period	Location	Province	Region	Recruitment venue	Sampling method	Measurement period*	Number of FSW used condom	Total number of FSW	Condom Usage (%)	QA Score
Li Y, 2012 [192]	2010-2011	Xiao Gan	Hubei	South Central	Entertainment	--	P1M	265	400	66.3%	4
Liang YJ, 2012 [330]	2011/09	Fo Shan	Guangdong	South Central	Entertainment	--	LA	176	400	44.0%	0
Lin RL, 2012 [304]	2011	Yu Lin	Guangxi	South Central	Entertainment	Cluster sampling	LA	381	400	95.3%	4
Lin RL, 2012 [304]	2009-2011	Yu Lin	Guangxi	South Central	Entertainment	Cluster sampling	LA	1085	1200	90.4%	4
Lin RL, 2012 [304]	2011	Yu Lin	Guangxi	South Central	Entertainment	Cluster sampling	P1M	308	400	77.0%	4
Quan XB, 2012 [331]	2011/05-2011/07	Yu Lin	Guangxi	South Central	Entertainment	--	LA	381	400	95.3%	4
Quan XB, 2012 [331]	2011/05-2011/07	Yu Lin	Guangxi	South Central	Entertainment	--	P1M	308	400	77.0%	4
Tang J, 2012 [332]	2011	Gui Lin	Guangxi	South Central	Entertainment	--	LA	309	400	77.3%	4
Tang J, 2012 [332]	2011	Gui Lin	Guangxi	South Central	Entertainment	--	P1M	244	400	61.0%	4
Tang MJ, 2012[333]	2011	YuLin	Guangxi	South Central	Entertainment	--	LA	1067	1213	88.0%	4
Tang MJ, 2012 [333]	2011	YuLin	Guangxi	South Central	Entertainment	--	P1M	667	1213	55.0%	4
Wei XQ, 2012 [334]	2011	He Chi	Guangxi	South Central	--	--	LA	882	1110	79.5%	2
Wen XQ, 2012 [277]	2011	Gui Lin	Guangxi	South Central	Entertainment	--	LA	309	372	83.1%	4
Wen XQ, 2012 [277]	2011	Gui Lin	Guangxi	South Central	Entertainment	--	P1M	244	374	65.2%	4
Guo J, 2006 [335]	2002	Kunming	Yunnan	Southwest	Entertainment	Random sampling	LA	101	184	54.9%	2

First author, published year	Study period	Location	Province	Region	Recruitment venue	Sampling method	Measurement period*	Number of FSW used condom	Total number of FSW	Condom Usage (%)	QA Score
Guo J, 2006 [335]	2002	Kunming	Yunnan	Southwest	Entertainment	Random sampling	P1M	46	184	25.0%	2
Wang Z, 2005 [336]	2002/12	Dazhou	Sichuan	Southwest	Entertainment	--	LA	26	49	53.1%	4
Wang Z, 2005 [336]	2002/12	Dazhou	Sichuan	Southwest	Entertainment	--	P1M	32	49	65.3%	4
Wang Z, 2005 [336]	2002/12	Dazhou	Sichuan	Southwest	Entertainment	--	LA	34	80	42.5%	4
Wang Z, 2005 [336]	2002/12	Dazhou	Sichuan	Southwest	Entertainment	--	P1M	14	80	17.5%	4
Yang HW, 2003 [337]	2002/04-2002/05	Mianyang	Sichuan	Southwest	Entertainment	--	LA	66	78	84.6%	2
Fu YZ, 2006 [338]	2003	Jinping	Yunnan	Southwest	Entertainment	--	LA	124	155	80.0%	5
Fu YZ, 2006 [338]	2003	Jinping	Yunnan	Southwest	Entertainment	--	P1M	60	155	38.7%	5
Huang LH, 2006 [339]	2003	Dali	Yunnan	Southwest	Entertainment	Random sampling	LA	328	367	89.4%	4
Huang LH, 2006 [339]	2003	Dali	Yunnan	Southwest	Entertainment	Random sampling	P1M	263	369	71.3%	4
Huang ZM, 2006 [340]	2003/08	Luxi	Yunnan	Southwest	Entertainment	Two-stage probability sampling	LA	137	221	62.0%	3
Huang ZM, 2006 [340]	2003/08	Luxi	Yunnan	Southwest	Entertainment	Two-stage probability sampling	P1M	61	221	27.6%	3
Jiang HY, 2006 [341]	2003/10	Yuanjiang	Yunnan	Southwest	Entertainment	--	LA	92	216	42.6%	5
Jiang HY, 2006 [341]	2003/10	Yuanjiang	Yunnan	Southwest	Entertainment	--	P1M	59	216	27.3%	5
Jin Y, 2006 [342]	2003/03	Qujing	Yunna	Southwest	Sentinel sites	Random sampling	P1M	81	277	29.2%	6
Jin Y, 2006 [342]	2003/03	Qujing	Yunna	Southwest	Sentinel sites	Random sampling	LA	159	277	57.4%	6
Kang JX, 2008 [343]	2003/07-2003/09	--	Sichuan	Southwest	Entertainment	Random sampling	LA	284	364	78.0%	4
Kang JX, 2008 [343]	2003/07-2003/09	--	Sichuan	Southwest	Entertainment	Random sampling	P1M	132	364	36.3%	4

First author, published year	Study period	Location	Province	Region	Recruitment venue	Sampling method	Measurement period*	Number of FSW used condom	Total number of FSW	Condom Usage (%)	QA Score
Lai WH, 2009 [344]	2003	-	Sichuan	Southwest	Entertainment	Snowball sampling	LA	1921	2575	74.6%	4
Lau JT, 2007 [345]	2003	City TC, DC	Sichuan	Southwest	Entertainment	Convenience sampling	LA	338	402	84.1%	4
Lau JT, 2007 [345]	2003	City TC, DC	Sichuan	Southwest	Entertainment	Convenience sampling	P1M	175	402	43.5%	4
Lau JT, 2011 [346]	2003	Multiple locations In Sichuan	Sichuan	Southwest	Entertainment	Convenience sampling	LA	5792	7063	82.0%	5
Lau JT, 2011 [346]	2003	Multiple locations In Sichuan	Sichuan	Southwest	Entertainment	Convenience sampling	P1M	2889	7063	40.9%	5
Li JE, 2006 [347]	2003/07	Yuxi	Yunnan	Southwest	Entertainment	--	LA	104	212	49.1%	5
Li JE, 2006 [347]	2003/07	Yuxi	Yunnan	Southwest	Entertainment	--	P1M	75	212	35.4%	5
Li SJ, 2004 [348]	2003/06-2003/07	Bazhong	Sichuan	Southwest	Entertainment	--	LA	319	364	87.6%	4
Li SJ, 2004 [348]	2003/06-2003/07	Bazhong	Sichuan	Southwest	Entertainment	--	P1M	126	364	34.6%	4
Li ZF, 2006 [349]	2003	Gengma	Yunnan	Southwest	--	--	LA	140	201	69.7%	3
Li ZF, 2006 [349]	2003	Gengma	Yunnan	Southwest	--	--	P1M	54	201	26.9%	3
Luo GY, 2006 [350]	2003	Meishan	Sichuan	Southwest	Entertainment	Convenience sampling	LA	315	360	87.5%	4
Luo GY, 2006 [350]	2003	Meishan	Sichuan	Southwest	Entertainment	Convenience sampling	P1M	171	360	47.5%	4
Peng LZ, 2007 [351]	2003/08	Yuxi	Yunnan	Southwest	Entertainment	Random sampling	LA	192	366	52.5%	5
Peng LZ, 2007 [351]	2003/08	Yuxi	Yunnan	Southwest	Entertainment	Random sampling	P1M	140	366	38.3%	5
Shi XY, 2006 [352]	2003	Xujing	Yunnan	Southwest	Entertainment	Cluster sampling	LA	226	372	60.8%	3

First author, published year	Study period	Location	Province	Region	Recruitment venue	Sampling method	Measurement period*	Number of FSW used condom	Total number of FSW	Condom Usage (%)	QA Score
Shi XY, 2006 [352]	2003	Xujing	Yunnan	Southwest	Entertainment	Cluster sampling	P1M	20	372	5.4%	3
Wang GX, 2006 [353]	2003/07	--	Yunnan	Southwest	Entertainment	--	P1M	37	230	16.1%	3
Wang GX, 2006 [353]	2003/07	--	Yunnan	Southwest	Entertainment	--	LA	84	230	36.5%	3
Wang LL, 2004 [354]	2003/06-2003/07	Leshan	Sichuan	Southwest	Entertainment	Convenience sampling	LA	321	360	89.2%	4
Wang LL, 2004 [354]	2003/06-2003/07	Leshan	Sichuan	Southwest	Entertainment	Convenience sampling	P1M	167	360	46.4%	4
Zhang YL, 2006 [355]	2003	Yuxi	Yunnan	Southwest	Entertainment	--	LA	192	366	52.5%	4
Zhang YL, 2006 [355]	2003	Yuxi	Yunnan	Southwest	Entertainment	--	P1M	140	366	38.3%	4
Zhao Q, 2004 [356]	2003/06	Dazhou	Sichuan	Southwest	Entertainment	Cluster sampling	LA	8	32	25.0%	5
Zhao Q, 2004 [356]	2003/06	Dazhou	Sichuan	Southwest	Entertainment	Cluster sampling	LA	3	128	2.3%	5
Chen XY, 2006 [357]	2004/01	Wenshan	Yunnan	Southwest	Entertainment	Probability sampling	LA	117	199	58.8%	4
Duan Y, 2006 [358]	2004	Lijiang	Yunnan	Southwest	Entertainment	--	LA	282	314	89.8%	4
Duan Y, 2006 [358]	2004	Lijiang	Yunnan	Southwest	Entertainment	--	P1M	237	314	75.5%	4
He LS, 2010 [359]	2004/01	Ezhou	Hubei	Southwest	Entertainment	--	LA	137	150	91.3%	4
He LS, 2010 [359]	2004/01	Ezhou	Hubei	Southwest	Entertainment	--	P1M	83	150	55.3%	4
Jiang M, 2005 [232]	2004/08-2004/10	Jingzhou	Hubei	Southwest	Entertainment	Random sampling	P1M	72	211	34.1%	4
Jing XM, 2006 [360]	2004/06-2004/07	Chongqing	Chongqing	Southwest	Entertainment	--	LA	129	196	65.8%	4

First author, published year	Study period	Location	Province	Region	Recruitment venue	Sampling method	Measurement period*	Number of FSW used condom	Total number of FSW	Condom Usage (%)	QA Score
Lau JT, 2007 [361]	2002-2004	Multiple locations In Sichuan	Sichuan	Southwest	Entertainment	Convenience sampling	LA	12393	15323	80.9%	4
Lau JT, 2007 [361]	2002-2004	Multiple locations In Sichuan	Sichuan	Southwest	Entertainment	Convenience sampling	P1M	6119	15312	40.0%	4
Lau JT, 2007 [345]	2004	City TC, DC	Sichuan	Southwest	Entertainment	Convenience sampling	LA	283	380	74.5%	4
Lau JT, 2007 [345]	2004	City TC, DC	Sichuan	Southwest	Entertainment	Convenience sampling	P1M	152	380	40.0%	4
Lau JT, 2011 [346]	2004	Multiple locations In Sichuan	Sichuan	Southwest	Entertainment	Convenience sampling	LA	5631	6875	81.9%	5
Lau JT, 2011 [346]	2004	Multiple locations In Sichuan	Sichuan	Southwest	Entertainment	Convenience sampling	P1M	2750	6875	40.0%	5
Lei ZQ, 2005 [362]	2004/04	Dazhou	Sichuan	Southwest	Entertainment	Convenience sampling	LA	150	259	57.9%	4
Luo GY, 2006 [350]	2004	Meishan	Sichuan	Southwest	Entertainment	Convenience sampling	P1M	134	361	37.1%	4
Luo XR, 2009 [363]	2004	Yibin	Sichuan	Southwest	Entertainment	--	LA	705	927	76.1%	5
Luo XR, 2009 [363]	2004	Yibin	Sichuan	Southwest	Entertainment	--	LA	21	30	70.0%	5
Luo XR, 2009 [363]	2004	Yibin	Sichuan	Southwest	Entertainment	--	LA	169	263	64.3%	5
Mao GY, 2005 [364]	2003-2004	Liangshan	Sichuan	Southwest	Entertainment	--	LA	184	360	51.1%	5
Su DT, 2005 [365]	2004/03	Chengdu	Sichuan	Southwest	Entertainment	--	LA	85	154	55.2%	4
Su DT, 2005 [365]	2004/03	Chengdu	Sichuan	Southwest	Entertainment	--	P1M	114	154	74.0%	4
Tan Y, 2006 [366]	2004	Jinghong	Yunnan	Southwest	Entertainment	--	LA	240	360	66.7%	1
Tan Y, 2006 [366]	2004	Jinghong	Yunnan	Southwest	Entertainment	--	P1M	107	360	29.7%	1

First author, published year	Study period	Location	Province	Region	Recruitment venue	Sampling method	Measurement period*	Number of FSW used condom	Total number of FSW	Condom Usage (%)	QA Score
Wang AM, 2006 [367]	2004/10	Zhijin	Guizhou	Southwest	Entertainment	--	LA	99	150	66.0%	4
Wang QQ, 2005 [368]	2004/02	--	Sichuan	Southwest	Entertainment	--	LA	208	407	51.1%	3
Wang QQ, 2005 [368]	2004/11	--	Sichuan	Southwest	Entertainment	--	LA	176	407	43.2%	3
Wen Y, 2006 [369]	2004/03	Gejiu	Yunnan	Southwest	Mixed venues	--	LA	352	415	84.8%	4
Wen Y, 2006 [369]	2004/03	Gejiu	Yunnan	Southwest	Mixed venues	--	P1M	252	415	60.7%	4
Xu SM, 2006 [370]	2004/04-2004/11	Chongqing	Chongqing	Southwest	Detention Center	Continuous sampling	P1M	78	1113	7.0%	8
Zou YD, 2006 [371]	2004/04	Chuxiong	Yunnan	Southwest	Entertainment	--	LA	189	331	57.1%	6
Zou YD, 2006 [371]	2004/04	Chuxiong	Yunnan	Southwest	Entertainment	--	P1M	138	331	41.7%	6
Cao XY, 2006 [372]	2004/12-2005/01	Xichang	Sichuan	Southwest	Entertainment	--	P1M	185	343	53.9%	4
Cao XY, 2006 [373]	2004/12-2005/01	Xichang	Sichuan	Southwest	Entertainment	--	P1M	107	203	52.7%	5
Cao XY, 2007 [373]	2004/12-2005/01	Xichang	Sichuan	Southwest	Entertainment	--	P1M	178	330	53.9%	5
Chen CL, 2006 [374]	2005/03	Malong	Yunnan	Southwest	Entertainment	Random sampling	P1M	7	115	6.1%	7
Chen CL, 2006 [374]	2005/03	Malong	Yunnan	Southwest	Entertainment	Random sampling	LA	28	115	24.3%	7
Chen XH, 2006 [375]	2004/12-2005/01	Xichang	Sichuan	Southwest	Entertainment	--	P1M	178	330	53.9%	6
Hu HW, 2008 [376]	2005/07	Changsha	Hunan	Southwest	Entertainment	Random sampling	LA	107	133	80.5%	5
Hu HW, 2008 [376]	2005/07	Changsha	Hunan	Southwest	Entertainment	Random sampling	P1M	68	133	51.1%	5
Jiang ZQ, 2006 [377]	2004/12-2005/01	Xichang	Sichuan	Southwest	Entertainment	--	P1M	178	330	53.9%	4

First author, published year	Study period	Location	Province	Region	Recruitment venue	Sampling method	Measurement period*	Number of FSW used condom	Total number of FSW	Condom Usage (%)	QA Score
kang JX, 2008 [343]	2003-2005	--	Sichuan	Southwest	Entertainment	Random sampling	LA	908	1089	83.4%	4
kang JX, 2008 [343]	2003-2005	--	Sichuan	Southwest	Entertainment	Random sampling	P1M	507	1089	46.6%	4
Lau JT, 2007 [345]	2005	City TC, DC	Sichuan	Southwest	Entertainment	Convenience sampling	LA	331	371	89.2%	4
Lau JT, 2007 [345]	2005	City TC, DC	Sichuan	Southwest	Entertainment	Convenience sampling	P1M	213	371	57.4%	4
Lau JT, 2011 [346]	2005	Multiple locations In Sichuan	Sichuan	Southwest	Entertainment	Convenience sampling	LA	6017	6853	87.8%	5
Lau JT, 2011 [346]	2005	Multiple locations In Sichuan	Sichuan	Southwest	Entertainment	Convenience sampling	P1M	3612	6853	52.7%	5
Li L, 2007 [378]	2005/12	Kunming	Yunnan	Southwest	Entertainment	Random sampling	LA	1604	2355	68.1%	5
Li Z, 2009 [379]	2005/07-2005/08	Qinyang, Chengdu	Sichuan	Southwest	Entertainment	--	LA	220	394	55.8%	5
Li Z, 2009 [379]	2005/07-2005/08	Qinyang, Chengdu	Sichuan	Southwest	Entertainment	--	P1M	48	394	12.2%	5
Li ZL, 2006 [380]	2005/01	Yongning	Yunnan	Southwest	Entertainment	--	LA	125	164	76.2%	2
Li ZL, 2006 [380]	2005/01	Yongning	Yunnan	Southwest	Entertainment	--	P1M	53	164	32.3%	2
Luo GY, 2006 [381]	2005/08-2005/09	Meishan	Sichuan	Southwest	Entertainment	Convenience sampling	LA	59	62	95.2%	3
Luo GY, 2006 [381]	2005/08-2005/09	Meishan	Sichuan	Southwest	Entertainment	Convenience sampling	P1M	36	62	58.1%	3
Luo GY, 2006 [381]	2005/08-2005/09	Meishan	Sichuan	Southwest	Entertainment	Convenience sampling	LA	205	214	95.8%	3
Luo GY, 2006 [381]	2005/08-2005/09	Meishan	Sichuan	Southwest	Entertainment	Convenience sampling	P1M	90	214	42.1%	3

First author, published year	Study period	Location	Province	Region	Recruitment venue	Sampling method	Measurement period*	Number of FSW used condom	Total number of FSW	Condom Usage (%)	QA Score
Luo GY, 2006 [381]	2005/08-2005/09	Meishan	Sichuan	Southwest	Entertainment	Convenience sampling	LA	59	84	70.2%	3
Luo GY, 2006 [381]	2005/08-2005/09	Meishan	Sichuan	Southwest	Entertainment	Convenience sampling	P1M	16	84	19.0%	3
Ruan Y, 2006 [382]	2004/12-2005/01	Xichang	Sichuan	Southwest	Entertainment	--	P1M	185	343	53.9%	5
Tian LG, 2006 [383]	2004/12-2005/1	Xichang	Sichuan	Southwest	Community	--	P1M	178	330	53.9%	4
Wang C, 2007 [384]	2005/12	Simao	Yunnan	Southwest	Entertainment	Random sampling	P1M	210	250	84.0%	4
Wang JH, 2006 [385]	2005.02	Huaning	Yunnan	Southwest	Entertainment	--	LA	35	100	35.0%	6
Wang JH, 2006 [385]	2005/02	Huaning	Yunnan	Southwest	Entertainment	--	P1M	5	100	5.0%	6
Wang Y, 2007 [386]	2005	Mianyang	Sichuan	Southwest	Entertainment	Convenience sampling	LA	905	1175	77.0%	4
Wang Y, 2007 [386]	2005	Mianyang	Sichuan	Southwest	Entertainment	Convenience sampling	P1M	550	1175	46.8%	4
Wang YH, 2009 [387]	2005.8	Shangri-la	Yunnan	Southwest	Entertainment	--	LA	342	407	84.0%	3
Yuan MJ, 2006 [388]	2005/03	Deyang	Sichuan	Southwest	Entertainment	--	LA	39	41	95.1%	4
Yuan MJ, 2006 [388]	2005/03	Deyang	Sichuan	Southwest	Entertainment	--	LA	27	41	65.9%	4
Yuzhen LC, 2006 [389]	2005/06-2005/08	Lasa	Tibet	Southwest	Entertainment	--	LA	274	881	31.1%	2
Zeng K, 2007 [390]	2002-2005	Aba	Sichuan	Southwest	Entertainment	--	LA	984	1203	81.8%	2
Zhang Q, 2006 [79]	2005	Nanchong	Sichuan	Southwest	Entertainment	Random sampling	LA	84	103	81.6%	2
Zhang Q, 2006 [79]	2005	Nanchong	Sichuan	Southwest	Entertainment	Random sampling	P1M	71	103	68.9%	2
Zhang Q, 2006 [79]	2005	Nanchong	Sichuan	Southwest	Entertainment	Random sampling	LA	118	259	45.6%	2

First author, published year	Study period	Location	Province	Region	Recruitment venue	Sampling method	Measurement period*	Number of FSW used condom	Total number of FSW	Condom Usage (%)	QA Score
Zhang Q, 2006 [79]	2005	Nanchong	Sichuan	Southwest	Entertainment	Random sampling	P1M	52	259	20.1%	2
Zou YD, 2006 [371]	2005/05	Chuxiong	Yunnan	Southwest	Entertainment	--	LA	388	400	97.0%	6
Zou YD, 2006 [371]	2005/05	Chuxiong	Yunnan	Southwest	Entertainment	--	P1M	350	400	87.5%	6
Du JQ, 2008 [391]	2006	Kaiyuan	Yunnan	Southwest	Entertainment	--	LA	303	331	91.5%	4
Du JQ, 2008 [391]	2006	Kaiyuan	Yunnan	Southwest	Entertainment	--	P1M	293	363	80.7%	4
Gu J, 2011 [392]	2005/08-2006/04	Dazhou	Sichuan	Southwest	Mixed venues	Snowball sampling	P1M	36	216	16.7%	6
Gu J, 2011 [392]	2005/08-2006/04	Dazhou	Sichuan	Southwest	Mixed venues	Snowball sampling	LA	144	216	66.7%	6
Huang Y, 2006 [393]	2006	Leshan	Sichuan	Southwest	Entertainment	--	LA	233	356	65.4%	4
Huang Y, 2006 [393]	2006	Leshan	Sichuan	Southwest	Entertainment	--	P1M	88	356	24.7%	4
Ji JH, 2007 [394]	2006/10-2006/11	Changjiang	Hainan	Southwest	Entertainment	--	LA	268	378	70.9%	4
Ji JH, 2007 [394]	2006/10-2006/11	Changjiang	Hainan	Southwest	Entertainment	--	P1M	104	378	27.5%	4
Lau JTF, 2012 [395]	2005-2006	Dazhou	Sichuan	Southwest	Entertainment	Snowball sampling	P1M	117	376	31.1%	7
Lei JH, 2012 [396]	2006	Kai Li	Guizhou	Southwest	--	--	LA	351	400	87.8%	2
Lei JH, 2012 [396]	2006	Kai Li	Guizhou	Southwest	--	--	P1M	260	400	65.0%	2
Li DM, 2007 [397]	2006	Guiyang	Guizhou	Southwest	Entertainment	Random sampling	LA	177	220	80.5%	7
Li DM, 2007 [397]	2006	Guiyang	Guizhou	Southwest	Entertainment	Random sampling	P1M	127	220	57.7%	7
Li DM, 2007 [397]	2006	Guiyang	Guizhou	Southwest	Entertainment	Random sampling	LA	174	212	82.1%	7
Li DM, 2007 [397]	2006	Guiyang	Guizhou	Southwest	Entertainment	Random sampling	P1M	67	212	31.6%	7
Li QH, 2009 [398]	2006/09-2006/10	Kaiyuan	Yunnan	Southwest	Entertainment	Venue-based sampling	LA	326	335	97.3%	7

First author, published year	Study period	Location	Province	Region	Recruitment venue	Sampling method	Measurement period*	Number of FSW used condom	Total number of FSW	Condom Usage (%)	QA Score
Li QH, 2009 [398]	2006/09-2006/10	Kaiyuan	Yunnan	Southwest	Entertainment	Venue-based sampling	LA	304	312	97.4%	7
Li QH, 2009 [398]	2006/09-2006/10	Kaiyuan	Yunnan	Southwest	Entertainment	Venue-based sampling	LA	56	87	64.4%	7
Li QH, 2010 [399]	2006/03-2006/04	Kaiyuan	Yunnan	Southwest	Entertainment	--	LA	660	718	91.9%	6
Li YY, 2009 [400]	2004/10-2006/12	Gejiu	Yunnan	Southwest	Entertainment	Random sampling	LA	352	415	84.8%	4
Li YY, 2009 [400]	2004/10-2006/12	Gejiu	Yunnan	Southwest	Entertainment	Random sampling	P1M	252	415	60.7%	4
Luo XR, 2008 [41]	05/2006-06/2006	Yibin	Sichuan	Southwest	Entertainment	--	LA	127	216	58.8%	5
Luo XR, 2008 [41]	2006/05-2006/06	Yibin	Sichuan	Southwest	Entertainment	--	LA	115	160	71.9%	5
Luo XR, 2008 [41]	2006/05-2006/06	Yibin	Sichuan	Southwest	Entertainment	--	LA	12	56	21.4%	5
Shi XL, 2008 [401]	2006	Suining	Sichuan	Southwest	Entertainment	--	LA	301	462	65.2%	6
Sun JY, 2012 [402]	2006	Jie Li	Guizhou	Southwest	Entertainment	--	LA	182	209	87.1%	4
Sun JY, 2012 [402]	2006	Jie Li	Guizhou	Southwest	Entertainment	--	LA	351	400	87.8%	4
Sun JY, 2012 [402]	2006	Jie Li	Guizhou	Southwest	Entertainment	--	LA	30	32	93.8%	4
Sun JY, 2012 [402]	2006	Jie Li	Guizhou	Southwest	Entertainment	--	LA	139	159	87.4%	4
Sun JY, 2012 [402]	2006	Jie Li	Guizhou	Southwest	Entertainment	--	P1M	260	400	65.0%	4
Sun JY, 2012 [402]	2006	Jie Li	Guizhou	Southwest	Entertainment	--	P1M	145	209	69.4%	4
Sun JY, 2012 [402]	2006	Jie Li	Guizhou	Southwest	Entertainment	--	P1M	93	159	58.5%	4
Sun JY, 2012 [402]	2006	Jie Li	Guizhou	Southwest	Entertainment	--	P1M	22	32	68.8%	4
Tan Y, 2011 [403]	2006	Gan Zi	Sichuan	Southwest	Entertainment	--	LA	319	381	83.7%	3
Tan Y, 2011 [403]	2006	Gan Zi	Sichuan	Southwest	Entertainment	--	P1M	139	378	36.8%	3
Wang GX, 2009 [404]	2006	-	Yunnan	Southwest	Entertainment	--	LA	338	363	93.1%	1

First author, published year	Study period	Location	Province	Region	Recruitment venue	Sampling method	Measurement period*	Number of FSW used condom	Total number of FSW	Condom Usage (%)	QA Score
Wang GX, 2009 [404]	2006	--	Yunnan	Southwest	Entertainment	--	P1M	293	363	80.7%	1
Wang HB, 2007 [405]	2006/03-2006/05	--	Yunnan	Southwest	Entertainment	--	P1M	212	279	76.0%	4
Wu Q, 2011 [406]	2006	Si mao, Qin lin, Da li, Lu feng, Meng zi,	Yunnan	Southwest	Entertainment	Cluster sampling	LA	903	1065	84.8%	4
Wu Q, 2011 [406]	2006	Si mao, Qin lin, Da li, Lu feng, Meng zi,	Yunnan	Southwest	Entertainment	Cluster sampling	P1M	797	1065	74.8%	4
Zheng WB, 2012 [407]	2006/01	Bao Shan	Yunnan	Southwest	Entertainment	--	LA	105	144	72.9%	3
Zheng WB, 2012 [407]	2006/07	Bao Shan	Yunnan	Southwest	Entertainment	--	LA	159	162	98.1%	3
Zheng WB, 2012 [407]	2006/01	Bao Shan	Yunnan	Southwest	Entertainment	--	P1M	47	144	32.6%	3
Zheng Y, 2010 [408]	2006	Chongqing	Chongqing	Southwest	Entertainment	--	LA	159	231	68.8%	7
Zheng Y, 2010 [408]	2006	Chongqing	Chongqing	Southwest	Entertainment	--	P1M	75	231	32.5%	7
Zheng Y, 2010 [408]	2006	Chongqing	Chongqing	Southwest	Entertainment	--	LA	81	162	50.0%	7
Zheng Y, 2010 [408]	2006	Chongqing	Chongqing	Southwest	Entertainment	--	P1M	29	162	17.9%	7
Zhi Q, 2011 [409]	2006	Chongqing	Chongqing	Southwest	Entertainment	--	LA	96	122	78.7%	3
Zhi Q, 2011 [409]	2006	Chongqing	Chongqing	Southwest	Entertainment	--	LA	310	400	77.5%	3
Zhi Q, 2011 [409]	2006	Chongqing	Chongqing	Southwest	Entertainment	--	LA	22	37	59.5%	3
Zhi Q, 2011 [409]	2006	Chongqing	Chongqing	Southwest	Entertainment	--	LA	193	241	80.1%	3
Zhi Q, 2011 [409]	2006	Chongqing	Chongqing	Southwest	Entertainment	--	P1M	91	400	22.8%	3
Zhi Q, 2011 [409]	2006	Chongqing	Chongqing	Southwest	Entertainment	--	P1M	49	122	40.2%	3
Zhi Q, 2011 [409]	2006	Chongqing	Chongqing	Southwest	Entertainment	--	P1M	50	241	20.7%	3
Zhi Q, 2011 [409]	2006	Chongqing	Chongqing	Southwest	Entertainment	--	P1M	8	37	21.6%	3
Liu ZY, 2010 [410]	2007	Qilin	Yunnan	Southwest	--	--	LA	295	365	80.8%	6

First author, published year	Study period	Location	Province	Region	Recruitment venue	Sampling method	Measurement period*	Number of FSW used condom	Total number of FSW	Condom Usage (%)	QA Score
Liu ZY, 2010 [410]	2007	Qilin	Yunnan	Southwest	--	--	P1M	156	365	42.7%	6
Dong XY, 2009 [159]	2007/08-2007/10	Tianjin	Tianjin	Southwest	Detention Center	--	P1M	158	178	88.8%	5
Fu SG, 2010 [27]	2007	--	Sichuan	Southwest	Sentinel sites	--	P1M	1679	3059	54.9%	5
Gao LL, 2008 [411]	2007	--	Yunnan	Southwest	Detention Center	--	LA	41	52	78.8%	4
Gao LL, 2008 [411]	2007	--	Yunnan	Southwest	Detention Center	--	P1M	29	52	55.8%	4
Gao LL, 2008 [411]	2007	--	Yunnan	Southwest	Detention Center	--	LA	37	43	86.0%	4
Gao LL, 2008 [411]	2007	--	Yunnan	Southwest	Detention Center	--	P1M	33	43	76.7%	4
Gao LL, 2008 [411]	2007	--	Yunnan	Southwest	Detention Center	--	LA	141	175	80.6%	4
Gao LL, 2008 [411]	2007	--	Yunnan	Southwest	Detention Center	--	P1M	108	175	61.7%	4
Guo L, 2011[412]	2007	--	Guizhou	Southwest	Entertainment	--	P1M	191	272	70.2%	4
Han DL, 2010 [413]	2007/04	Xindu district, Chengdu	Sichuan	Southwest	Entertainment	Two-stage stratified sampling	LA	127	267	47.6%	5
Han DL, 2010 [413]	2007/04	Chenghua district, Chengdu	Sichuan	Southwest	Entertainment	Two-stage stratified sampling	LA	174	315	55.2%	5
Han DL, 2010 [413]	2007/04	Xindu district, Chengdu	Sichuan	Southwest	Entertainment	Two-stage stratified sampling	P1M	75	267	28.1%	5
Han DL, 2010 [413]	2007/04	Chenghua district, Chengdu	Sichuan	Southwest	Entertainment	Two-stage stratified sampling	P1M	18	315	5.7%	5
Han WX, 2012 [414]	2007	Long Chuan	Yunnan	Southwest	--	--	LA	113	137	82.5%	2

First author, published year	Study period	Location	Province	Region	Recruitment venue	Sampling method	Measurement period*	Number of FSW used condom	Total number of FSW	Condom Usage (%)	QA Score
Han WX, 2012 [414]	2007	Long Chuan	Yunnan	Southwest	--	--	P1M	79	137	57.7%	2
He JC, 2010 [415]	2007/07	Chongqing	Chongqing	Southwest	Entertainment	Random sampling	LA	159	231	68.8%	4
He JC, 2010 [415]	2007/07	Chongqing	Chongqing	Southwest	Entertainment	Random sampling	P1M	75	231	32.5%	4
He JC, 2010 [415]	2007/07	Chongqing	Chongqing	Southwest	Entertainment	Random sampling	LA	100	186	53.8%	4
He JC, 2010 [415]	2007/07	Chongqing	Chongqing	Southwest	Entertainment	Random sampling	P1M	36	186	19.4%	4
He QX, 2011 [416]	2007	Luliang	Yunnan	Southwest	Entertainment	--	LA	210	365	57.5%	3
He QX, 2011 [416]	2007	Luliang	Yunnan	Southwest	Entertainment	--	P1M	80	365	21.9%	3
He QX, 2011 [417]	2007	Luliang	Yunnan	Southwest	Entertainment	Two-stage probability sampling method	LA	210	365	57.5%	4
He QX, 2011 [417]	2007	Luliang	Yunnan	Southwest	Entertainment	Two-stage probability sampling method	P1M	80	365	21.9%	4
He QX, 2011 [417]	2007	Luliang	Yunnan	Southwest	Entertainment	Two-stage probability sampling method	LA	210	365	57.5%	4
He QX, 2011 [417]	2007	Luliang	Yunnan	Southwest	Entertainment	Two-stage probability sampling method	P1M	80	365	21.9%	4
Liu CQ, 2010 [418]	2007	Kunming	Yunnan	Southwest	--	--	P1M	283	365	77.5%	4
Liu CQ, 2010 [418]	2007	Kunming	Yunnan	Southwest	--	--	LA	343	365	94.0%	4

First author, published year	Study period	Location	Province	Region	Recruitment venue	Sampling method	Measurement period*	Number of FSW used condom	Total number of FSW	Condom Usage (%)	QA Score
Lu PN, 2008 [419]	2007/04-2007/05	Panzhihua	Sichuan	Southwest	Entertainment	Convenience sampling	LA	163	358	45.5%	3
Lu PN, 2008 [419]	2007/04-2007/05	Panzhihua	Sichuan	Southwest	Entertainment	Convenience sampling	P1M	60	358	16.8%	3
Luo L, 2009 [50]	2007/07-2007/09	Mianyang	Sichuan	Southwest	Entertainment	Random sampling	P1M	186	411	45.3%	7
Luo L, 2009 [50]	2007/07-2007/09	Mianyang	Sichuan	Southwest	Entertainment	Random sampling	LA	310	411	75.4%	7
Luo XR, 2009 [363]	2006/04-2007/11	Yibin	Sichuan	Southwest	Entertainment	--	LA	736	927	79.4%	5
Luo XR, 2009 [363]	2006/04-2007/11	Yibin	Sichuan	Southwest	Entertainment	--	LA	22	30	73.3%	5
Luo XR, 2009 [363]	2006/04-2007/11	Yibin	Sichuan	Southwest	Entertainment	--	LA	176	263	66.9%	5
Luo YJ, 2010 [420]	2007	Sichuan	Sichuan	Southwest	Entertainment	Stratified sampling	LA	229	302	75.8%	5
Luo YJ, 2010 [420]	2007	Sichuan	Sichuan	Southwest	Entertainment	Stratified sampling	P1M	157	302	52.0%	5
Peng HB, 2007 [421]	2007/04-2007/05	Nanchong	Sichuan	Southwest	Entertainment	Stratified random sampling	LA	377	419	90.0%	5
Peng HB, 2007 [421]	2007/04-2007/05	Nanchong	Sichuan	Southwest	Entertainment	Stratified random sampling	P1M	255	419	60.9%	5
Shi XL, 2008 [401]	2007	Suining	Sichuan	Southwest	Entertainment	--	LA	335	400	83.8%	6
Wen TJ, 2009 [422]	2007/04-2007/05	Panzhihua	Sichuan	Southwest	Entertainment	Convenience and snowball sampling	LA	78	358	21.8%	3
Wen TJ, 2009 [422]	2007/04-2007/05	Panzhihua	Sichuan	Southwest	Entertainment	Convenience and snowball sampling	P1M	60	358	16.8%	3

First author, published year	Study period	Location	Province	Region	Recruitment venue	Sampling method	Measurement period*	Number of FSW used condom	Total number of FSW	Condom Usage (%)	QA Score
Wu CL, 2010 [423]	2007/10-2007/11	Xichang	Sichuan	Southwest	Entertainment	Cluster sampling	LA	425	444	95.7%	5
Wu CL, 2010 [423]	2007/10-2007/11	Xichang	Sichuan	Southwest	Entertainment	Cluster sampling	P1M	313	444	70.5%	5
Wu MR, 2004 [424]	2003-2007	Luzhou	Sichuan	Southwest	Entertainment	--	LA	282	360	78.3%	2
Wu MR, 2004 [424]	2003-2007	Luzhou	Sichuan	Southwest	Entertainment	--	P1M	85	360	23.6%	2
Yang JF, 2012 [425]	2007/01-2007/07	Bao shan	Yunnan	Southwest	Entertainment	--	LA	1020	1080	94.4%	4
Yang JF, 2012 [425]	2007/01-2007/07	Bao shan	Yunnan	Southwest	Entertainment	--	P1M	871	1080	80.6%	4
Zhang CP, 2008 [426]	2007/04	Lijiang	Yunnan	Southwest	Entertainment	--	P1M	274	483	56.7%	3
Zheng WB, 2012 [407]	2007/01	Bao shan	Yunnan	Southwest	Entertainment	--	LA	194	196	99.0%	3
Zhou SX, 2007 [427]	2006-2007	Changning	Sichuan	Southwest	Entertainment	--	LA	99	132	75.0%	4
Zhou SX, 2007 [427]	2006-2007	Changning	Sichuan	Southwest	Entertainment	--	P1M	57	132	43.2%	4
Zhou SX, 2007 [427]	2006-2007	Changning	Sichuan	Southwest	Entertainment	--	LA	31	50	62.0%	4
Zhou SX, 2007 [427]	2006-2007	Changning	Sichuan	Southwest	Entertainment	--	P1M	15	50	30.0%	4
Liu ZY, 2010 [410]	2008	Qilin	Yunnan	Southwest	--	--	LA	390	409	95.4%	6
Liu ZY, 2010 [410]	2008	Qilin	Yunnan	Southwest	--	--	P1M	275	409	67.2%	6
Chen Y, 2009 [428]	2008/05-2008/07	Huangshi	Hubei	Southwest	Entertainment	Random sampling	P1M	116	172	67.4%	4
Chen Y, 2009 [428]	2008/05-2008/07	Huangshi	Hubei	Southwest	Entertainment	Random sampling	LA	143	172	83.1%	4

First author, published year	Study period	Location	Province	Region	Recruitment venue	Sampling method	Measurement period*	Number of FSW used condom	Total number of FSW	Condom Usage (%)	QA Score
Dong LM, 2010 [429]	2008/04-2008/06	A District Zigong	Sichuan	Southwest	Entertainment	--	LA	471	536	87.9%	6
Dong LM, 2010 [429]	2008/04-2008/06	A District Zigong	Sichuan	Southwest	Entertainment	--	LA	249	536	46.5%	6
Fu JB, 2010 [430]	2008/09-2008/12	Zhoushan	Zhejiang	Southwest	Entertainment	Convenience sampling	LA	79	100	79.0%	3
Fu JB, 2010 [430]	2008/09-2008/12	Zhoushan	Zhejiang	Southwest	Entertainment	Convenience sampling	P1M	33	100	33.0%	3
Gan GC, 2010 [431]	2008	Jiangyou	Sichuan	Southwest	Entertainment	Convenience sampling	LA	247	264	93.6%	5
Gan GC, 2010 [431]	2008	Jiangyou	Sichuan	Southwest	Entertainment	Convenience sampling	P1M	174	264	65.9%	5
Han DL, 2010 [413]	2008/09	Xindu district, Chengdu	Sichuan	Southwest	Entertainment	Two-stage stratified sampling	LA	115	199	57.8%	5
Han DL, 2010 [413]	2008/09	Chenghua district, Chengdu	Sichuan	Southwest	Entertainment	Two-stage stratified sampling	LA	199	264	75.4%	5
Han DL, 2010 [413]	2008/09	Xindu district, Chengdu	Sichuan	Southwest	Entertainment	Two-stage stratified sampling	P1M	67	199	33.7%	5
Han DL, 2010 [413]	2008/09	Chenghua district, Chengdu	Sichuan	Southwest	Entertainment	Two-stage stratified sampling	P1M	4	264	1.5%	5
Han WX, 2012 [414]	2008	Long Chuan	Yunnan	Southwest	--	--	LA	117	121	96.7%	2
Han WX, 2012 [414]	2008	Long Chuan	Yunnan	Southwest	--	--	P1M	98	121	81.0%	2
He QX, 2011 [416]	2008	Lu liang county	Yunnan	Southwest	Entertainment	--	LA	310	364	85.2%	3
He QX, 2011 [416]	2008	Lu liang county	Yunnan	Southwest	Entertainment	--	P1M	181	364	49.7%	3
Lei JH, 2012 [396]	2008	Kai Li	Guizhou	Southwest	--	--	LA	365	400	91.3%	2

First author, published year	Study period	Location	Province	Region	Recruitment venue	Sampling method	Measurement period*	Number of FSW used condom	Total number of FSW	Condom Usage (%)	QA Score
Lei JH, 2012 [396]	2008	Kai Li	Guizhou	Southwest	--	--	P1M	306	400	76.5%	2
Li L, 2009 [432]	2008/12	Kunming	Yunnan	Southwest	Entertainment	--	LA	2126	2472	86.0%	4
Li L, 2009 [432]	2008/12	Kunming	Yunnan	Southwest	Entertainment	--	P1M	1772	2472	71.7%	4
Li SH, 2010 [433]	2008/10	Kunming	Yunnan	Southwest	Entertainment	--	LA	129	200	64.5%	5
Li SH, 2010 [433]	2008/10	Kunming	Yunnan	Southwest	Entertainment	--	P1M	117	200	58.5%	5
Li WZ, 2009 [434]	2008	Jianshui	Yunnan	Southwest	Entertainment	Probability proportional to size sampling	LA	374	393	95.2%	6
Liu CQ, 2010 [418]	2008	Kunming	Yunnan	Southwest	--	--	P1M	333	370	90.0%	4
Liu CQ, 2010 [418]	2008	Kunming	Yunnan	Southwest	--	--	LA	346	370	93.5%	4
Sun JY, 2012 [402]	2008	Jie Li	Guizhou	Southwest	Entertainment	--	LA	159	173	91.9%	4
Sun JY, 2012 [402]	2008	Jie Li	Guizhou	Southwest	Entertainment	--	LA	365	400	91.3%	4
Sun JY, 2012 [402]	2008	Jie Li	Guizhou	Southwest	Entertainment	--	LA	201	221	91.0%	4
Sun JY, 2012 [402]	2008	Jie Li	Guizhou	Southwest	Entertainment	--	P1M	306	400	76.5%	4
Sun JY, 2012 [402]	2008	Jie Li	Guizhou	Southwest	Entertainment	--	P1M	145	173	83.8%	4
Sun JY, 2012 [402]	2008	Jie Li	Guizhou	Southwest	Entertainment	--	P1M	158	221	71.5%	4
Wu Y, 2012 [435]	2008	Hong Ya	Sichuan	Southwest	--	Two-stage sampling	LA	45	60	75.0%	4
Wu Y, 2012 [435]	2008	Hong Ya	Sichuan	Southwest	--	Two-stage sampling	LA	93	200	46.5%	4
Wu Y, 2012 [435]	2008	Hong Ya	Sichuan	Southwest	--	Two-stage sampling	LA	6	60	10.0%	4
Wu Y, 2012 [435]	2008	Hong Ya	Sichuan	Southwest	--	Two-stage sampling	LA	42	80	52.5%	4
Wu Y, 2012 [435]	2008	Hong Ya	Sichuan	Southwest	--	Two-stage sampling	P1M	45	200	22.5%	4
Wu Y, 2012 [435]	2008	Hong Ya	Sichuan	Southwest	--	Two-stage sampling	P1M	18	60	30.0%	4

First author, published year	Study period	Location	Province	Region	Recruitment venue	Sampling method	Measurement period*	Number of FSW used condom	Total number of FSW	Condom Usage (%)	QA Score
Wu Y, 2012 [435]	2008	Hong Ya	Sichuan	Southwest	--	Two-stage sampling	P1M	22	80	27.5%	4
Wu Y, 2012 [435]	2008	Hong Ya	Sichuan	Southwest	--	Two-stage sampling	P1M	5	60	8.3%	4
Yang GX, 2009 [436]	2008.10-2008.11	Cangxi	Sichuan	Southwest	Entertainment	--	LA	129	200	64.5%	2
Yang GX, 2009 [436]	2008.10-2008.11	Cangxi	Sichuan	Southwest	Entertainment	--	P1M	34	200	17.0%	2
Yang JF, 2012 [425]	2008/01-2008/07	Bao Shan	Yunnan	Southwest	Entertainment	--	LA	1040	1082	96.1%	4
Yang JF, 2012 [425]	2008/01-2008/07	Bao Shan	Yunnan	Southwest	Entertainment	--	P1M	916	1082	84.7%	4
Yang ZJ, 2010 [437]	2007-2008	Ruili	Yunnan	Southwest	Entertainment	--	LA	735	751	97.9%	4
Yang ZJ, 2010 [437]	2007-2008	Ruili	Yunnan	Southwest	Entertainment	--	P1M	682	751	90.8%	4
Zhang YH, 2011 [96]	2008/04-2008/07	Chongqing	Chongqing	Southwest	--	--	LA	315	429	73.4%	2
Zhang YH, 2011 [96]	2008/04-2008/07	Kunming	Yunnan	Southwest	--	--	LA	344	405	84.9%	2
Zhang YH, 2011 [96]	2008/04-2008/07	Chongqing	Chongqing	Southwest	--	--	P1M	128	429	29.8%	2
Zhang YH, 2011 [96]	2008/04-2008/07	Kunming	Yunnan	Southwest	--	--	P1M	281	405	69.4%	2
Zhou Y, 2011 [438]	2008/08	Fu Shun	Sichuan	Southwest	Entertainment	--	LA	117	138	84.8%	4
Zhu Q, 2009 [439]	2008	Chuxiong	Yunnan	Southwest	Entertainment	--	LA	710	762	93.2%	4
Zi GS, 2009 [440]	2008/01	Weishan	Yunnan	Southwest	Entertainment	--	LA	83	85	97.6%	4
Zi GS, 2009 [440]	2008/01	Weishan	Yunnan	Southwest	Entertainment	--	P1M	68	85	80.0%	4
Liu ZY, 2010 [410]	2009	Qilin	Yunnan	Southwest	--	--	LA	394	400	98.5%	6
Liu ZY, 2010 [410]	2009	Qilin	Yunnan	Southwest	--	--	P1M	372	400	93.0%	6

First author, published year	Study period	Location	Province	Region	Recruitment venue	Sampling method	Measurement period*	Number of FSW used condom	Total number of FSW	Condom Usage (%)	QA Score
Dong CL, 2012 [441]	2009/12	Pu Er	Yunnan	Southwest	Entertainment	--	LA	196	201	97.5%	4
Dong CL, 2012 [441]	2009/12	Pu Er	Yunnan	Southwest	Entertainment	--	P1M	179	201	89.1%	4
Dong LM, 2010 [429]	2009/04-2009/06	B District Zigong	Sichuan	Southwest	Entertainment	--	LA	280	355	78.9%	6
Dong LM, 2010 [429]	2009/04-2009/06	B District Zigong	Sichuan	Southwest	Entertainment	--	P1M	172	355	48.5%	6
Fan SF, 2010 [442]	2009/05	-	Sichuan	Southwest	Entertainment	Stratified cluster sampling	LA	138	150	92.0%	5
Fan SF, 2010 [442]	2009/05	-	Sichuan	Southwest	Entertainment	Stratified cluster sampling	P1M	85	150	56.7%	5
Fu SG, 2010 [27]	2009	-	Sichuan	Southwest	Sentinel sites	--	P1M	1698	2734	62.1%	5
Han WX, 2012 [414]	2009	Long Chuan	Yunnan	Southwest	--	--	LA	109	128	85.2%	2
Han WX, 2012 [414]	2009	Long Chuan	Yunnan	Southwest	--	--	P1M	90	128	70.3%	2
Hao QZ, 2010 [443]	2009	Yingkou	Liaoning	Southwest	Entertainment	Random sampling	LA	78	123	63.4%	4
Hao QZ, 2010 [443]	2009	Yingkou	Liaoning	Southwest	Entertainment	Random sampling	P1M	73	123	59.3%	4
Hao QZ, 2010 [443]	2009	Yingkou	Liaoning	Southwest	Entertainment	Random sampling	LA	70	81	86.4%	4
Hao QZ, 2010 [443]	2009	Yingkou	Liaoning	Southwest	Entertainment	Random sampling	P1M	48	81	59.3%	4
Hao QZ, 2010 [443]	2009	Yingkou	Liaoning	Southwest	Entertainment	Random sampling	LA	39	56	69.6%	4
Hao QZ, 2010 [443]	2009	Yingkou	Liaoning	Southwest	Entertainment	Random sampling	P1M	25	56	44.6%	4
He B, 2011 [444]	2009/04-2009/05	An Ning	Yunnan	Southwest	Entertainment	--	LA	69	79	87.3%	4

First author, published year	Study period	Location	Province	Region	Recruitment venue	Sampling method	Measurement period*	Number of FSW used condom	Total number of FSW	Condom Usage (%)	QA Score
He B, 2011 [444]	2009/04-2009/05	An Ning	Yunnan	Southwest	Entertainment	--	LA	158	203	77.8%	4
He B, 2011 [444]	2009/04-2009/05	An Ning	Yunnan	Southwest	Entertainment	--	LA	35	57	61.4%	4
He B, 2011 [444]	2009/04-2009/05	An Ning	Yunnan	Southwest	Entertainment	--	LA	54	70	77.1%	4
He B, 2011 [444]	2009/04-2009/05	An Ning	Yunnan	Southwest	Entertainment	--	P1M	111	203	54.7%	4
He QX, 2011 [416]	2009	Luliang	Yunnan	Southwest	Entertainment	--	LA	374	400	93.5%	3
He QX, 2011 [416]	2009	Luliang	Yunnan	Southwest	Entertainment	--	P1M	295	400	73.8%	3
Huang JF, 2010 [445]	2009/08	Qingyuan	Yunnan	Southwest	Entertainment	Random sampling and Convenience sampling	LA	299	333	89.8%	5
Huang JF, 2010 [445]	2009/08	Qingyuan	Yunnan	Southwest	Entertainment	Random sampling and Convenience sampling	P1M	254	333	76.3%	5
Lei JH, 2012 [396]	2009	Kai Li	Guizhou	Southwest	--	--	LA	345	400	86.3%	2
Lei JH, 2012 [396]	2009	Kai Li	Guizhou	Southwest	--	--	P1M	275	400	68.8%	2
Li YK, 2012 [446]	2009	--	Sichuan	Southwest	--	--	LA	701	1041	67.3%	2
Li YK, 2012 [446]	2009	--	Sichuan	Southwest	--	--	P1M	402	1031	39.0%	2
Liang F, 2011 [447]	2009/03-2009/12	Jian Yang	Sichuan	Southwest	Entertainment	--	LA	54	58	93.1%	4
Liang F, 2011 [447]	2009/03-2009/12	Jian Yang	Sichuan	Southwest	Entertainment	--	LA	282	398	70.9%	4
Liang F, 2011 [447]	2009/03-2009/12	Jian Yang	Sichuan	Southwest	Entertainment	--	LA	20	36	55.6%	4
Liang F, 2011 [447]	2009/03-2009/12	Jian yang	Sichuan	Southwest	Entertainment	--	LA	208	304	68.4%	4

First author, published year	Study period	Location	Province	Region	Recruitment venue	Sampling method	Measurement period*	Number of FSW used condom	Total number of FSW	Condom Usage (%)	QA Score
Liang F, 2011 [447]	2009/03-2009/12	Jian yang	Sichuan	Southwest	Entertainment	--	P1M	247	398	62.1%	4
Liang F, 2011 [447]	2009/03-2009/12	Jian yang	Sichuan	Southwest	Entertainment	--	P1M	41	58	70.7%	4
Liang F, 2011 [447]	2009/03-2009/12	Jian yang	Sichuan	Southwest	Entertainment	--	P1M	190	304	62.5%	4
Liang F, 2011 [447]	2009/03-2009/12	Jian yang	Sichuan	Southwest	Entertainment	--	P1M	16	36	44.4%	4
Liu CQ, 2010 [418]	2009	Kunming	Yunnan	Southwest	--	--	P1M	318	376	84.6%	4
Liu CQ, 2010 [418]	2009	Kunming	Yunnan	Southwest	--	--	LA	370	376	98.4%	4
Liu L, 2010 [448]	2009/03-2009/12	Shuangliu	Sichuan	Southwest	Entertainment	--	LA	95	133	71.4%	4
Liu L, 2010 [448]	2009/03-2009/12	Shuangliu	Sichuan	Southwest	Entertainment	--	P1M	29	133	21.8%	4
Ou YN, 2010 [449]	2009/06	Kunming	Yunnan	Southwest	Entertainment	--	LA	314	345	91.0%	4
Sun JY, 2012 [402]	2009	Jie li	Guizhou	Southwest	Entertainment	--	LA	198	241	82.2%	4
Sun JY, 2012 [402]	2009	Jie Li	Guizhou	Southwest	Entertainment	--	LA	345	400	86.3%	4
Sun JY, 2012 [402]	2009	Jie Li	Guizhou	Southwest	Entertainment	--	LA	133	142	93.7%	4
Sun JY, 2012 [402]	2009	Jie Li	Guizhou	Southwest	Entertainment	--	P1M	275	400	68.8%	4
Sun JY, 2012 [402]	2009	Jie Li	Guizhou	Southwest	Entertainment	--	P1M	169	241	70.1%	4
Sun JY, 2012 [402]	2009	Jie Li	Guizhou	Southwest	Entertainment	--	P1M	94	142	66.2%	4
Wang QF, 2010 [450]	2009/05	Songming	Yunnan	Southwest	Entertainment	--	P1M	153	193	79.3%	3
Wang WW, 2010 [451]	2009/04	Neijiang	Sichuan	Southwest	VCT	Random stage cluster	P1M	122	406	30.0%	5
Wang WW, 2010 [451]	2009/04	Neijiang	Sichuan	Southwest	VCT	Random stage cluster	P1M	242	754	32.1%	5
Wu Y, 2012 [435]	2009	Hong Ya	Sichuan	Southwest	--	Two-stage sampling	LA	50	60	83.3%	4

First author, published year	Study period	Location	Province	Region	Recruitment venue	Sampling method	Measurement period*	Number of FSW used condom	Total number of FSW	Condom Usage (%)	QA Score
Wu Y, 2012 [435]	2009	Hong Ya	Sichuan	Southwest	--	Two-stage sampling	LA	133	200	66.5%	4
Wu Y, 2012 [435]	2009	Hong Ya	Sichuan	Southwest	--	Two-stage sampling	LA	22	80	27.5%	4
Wu Y, 2012 [435]	2009	Hong Ya	Sichuan	Southwest	--	Two-stage sampling	LA	61	80	76.3%	4
Wu Y, 2012 [435]	2009	Hong Ya	Sichuan	Southwest	--	Two-stage sampling	P1M	76	200	38.0%	4
Wu Y, 2012 [435]	2009	Hong Ya	Sichuan	Southwest	--	Two-stage sampling	P1M	30	60	50.0%	4
Wu Y, 2012 [435]	2009	Hong Ya	Sichuan	Southwest	--	Two-stage sampling	P1M	35	80	43.8%	4
Wu Y, 2012 [435]	2009	Hong Ya	Sichuan	Southwest	--	Two-stage sampling	P1M	11	80	13.8%	4
Xue HM, 2011 [452]	2009/12	Hekou	Yunnan	Southwest	Entertainment	--	LA	179	200	89.5%	5
Yan WZ, 2011 [453]	2009	Jiong Hong	Yunnan	Southwest	Entertainment	--	LA	167	179	93.3%	3
Yan WZ, 2011 [453]	2009	Jiong Hong	Yunnan	Southwest	Entertainment	--	P1M	148	180	82.2%	3
Yang JF, 2012 [425]	2009/01-2009/07	Bao Shan	Yunnan	Southwest	Entertainment	--	LA	1162	1200	96.8%	4
Yang JF, 2012 [425]	2009/01-2009/07	Bao Shan	Yunnan	Southwest	Entertainment	--	P1M	1079	1200	89.9%	4
Yang YH, 2012 [454]	2009/06	Pu Er	Yunnan	Southwest	Entertainment	Probability proportional to size sampling	LA	91	98	92.9%	2
Yang YH, 2012 [454]	2009/12	Pu Er	Yunnan	Southwest	Entertainment	Probability proportional to size sampling	LA	200	200	100.0%	2
Yang YH, 2012 [454]	2009/01	Pu Er	Yunnan	Southwest	Entertainment	Probability proportional to size sampling	LA	210	250	84.0%	2

First author, published year	Study period	Location	Province	Region	Recruitment venue	Sampling method	Measurement period*	Number of FSW used condom	Total number of FSW	Condom Usage (%)	QA Score
Yu XW, 2010 [455]	2009/04-2009/06	Lancang	Yunnan	Southwest	Entertainment	--	LA	307	320	95.9%	7
Yu XW, 2010 [455]	2009/04-2009/06	Lancang	Yunnan	Southwest	Entertainment	--	P1M	291	320	90.9%	7
Zheng Y, 2010 [408]	2009	Chongqing	Chongqing	Southwest	Entertainment	--	LA	131	146	89.7%	7
Zheng Y, 2010 [408]	2009	Chongqing	Chongqing	Southwest	Entertainment	--	P1M	82	146	56.2%	7
Zheng Y, 2010 [408]	2009	Chongqing	Chongqing	Southwest	Entertainment	--	LA	138	161	85.7%	7
Zheng Y, 2010 [408]	2009	Chongqing	Chongqing	Southwest	Entertainment	--	P1M	76	161	47.2%	7
Zheng Y, 2010 [408]	2009	Chongqing	Chongqing	Southwest	Entertainment	--	LA	88	105	83.8%	7
Zheng Y, 2010 [408]	2009	Chongqing	Chongqing	Southwest	Entertainment	--	P1M	42	105	40.0%	7
Zhi Q, 2011 [409]	2009	Chongqing	Chongqing	Southwest	Entertainment	--	LA	103	115	89.6%	3
Zhi Q, 2011 [409]	2009	Chongqing	Chongqing	Southwest	Entertainment	--	LA	367	402	91.3%	3
Zhi Q, 2011 [409]	2009	Chongqing	Chongqing	Southwest	Entertainment	--	LA	41	51	80.4%	3
Zhi Q, 2011 [409]	2009	Chongqing	Chongqing	Southwest	Entertainment	--	LA	222	236	94.1%	3
Ci Ren WM, 2012 [456]	2010/06-2010/07	La Sa	Tibet	Southwest	Entertainment	--	LA	257	442	58.1%	3
Guo HJ, 2011 [457]	2010/04-2010/07	Zun Yi	Guizhou	Southwest	--	--	LA	150	235	63.8%	4
Lei JH, 2012 [396]	2010	Kai Li	Guizhou	Southwest	--	--	LA	360	400	90.0%	2
Lei JH, 2012 [396]	2010	Kai Li	Guizhou	Southwest	--	--	P1M	257	400	64.3%	2
Li Y, 2012 [458]	2010	Lan Cang	Yunnan	Southwest	Entertainment	--	LA	287	301	95.3%	3
Sun JY, 2012 [402]	2010	Jie Li	Guizhou	Southwest	Entertainment	--	LA	194	226	85.8%	4
Sun JY, 2012 [402]	2010	Jie Li	Guizhou	Southwest	Entertainment	--	LA	361	400	90.3%	4
Sun JY, 2012 [402]	2010	Jie Li	Guizhou	Southwest	Entertainment	--	LA	141	148	95.3%	4
Sun JY, 2012 [402]	2010	Jie Li	Guizhou	Southwest	Entertainment	--	P1M	257	400	64.3%	4
Sun JY, 2012 [402]	2010	Jie Li	Guizhou	Southwest	Entertainment	--	P1M	130	226	57.5%	4
Sun JY, 2012 [402]	2010	Jie Li	Guizhou	Southwest	Entertainment	--	P1M	104	148	70.3%	4

First author, published year	Study period	Location	Province	Region	Recruitment venue	Sampling method	Measurement period*	Number of FSW used condom	Total number of FSW	Condom Usage (%)	QA Score
Tan Y, 2011 [403]	2010	Gan Zi	Sichuan	Southwest	Entertainment	--	LA	377	399	94.5%	3
Tan Y, 2011 [403]	2010	Gan Zi	Sichuan	Southwest	Entertainment	--	P1M	354	398	88.9%	3
Wu Y, 2012 [435]	2010	Hong Ya	Sichuan	Southwest	--	Two-stage sampling	LA	54	60	90.0%	4
Wu Y, 2012 [435]	2010	Hong Ya	Sichuan	Southwest	--	Two-stage sampling	LA	153	200	76.5%	4
Wu Y, 2012 [435]	2010	Hong Ya	Sichuan	Southwest	--	Two-stage sampling	LA	32	60	53.3%	4
Wu Y, 2012 [435]	2010	Hong Ya	Sichuan	Southwest	--	Two-stage sampling	LA	67	80	83.8%	4
Wu Y, 2012 [435]	2010	Hong Ya	Sichuan	Southwest	--	Two-stage sampling	P1M	92	200	46.0%	4
Wu Y, 2012 [435]	2010	Hong Ya	Sichuan	Southwest	--	Two-stage sampling	P1M	34	60	56.7%	4
Wu Y, 2012 [435]	2010	Hong Ya	Sichuan	Southwest	--	Two-stage sampling	P1M	43	80	53.8%	4
Wu Y, 2012 [435]	2010	Hong Ya	Sichuan	Southwest	--	Two-stage sampling	P1M	15	60	25.0%	4
Yang JF, 2012 [425]	2010/01-2010/07	Bao Shan	Yunnan	Southwest	Entertainment	--	LA	1294	1405	92.1%	4
Yang JF, 2012 [425]	2010/01-2010/07	Bao Shan	Yunnan	Southwest	Entertainment	--	P1M	1082	1405	77.0%	4
Yang ZJ, 2012 [459]	2010/03-2010/06	Rui Li	Yunnan	Southwest	Entertainment	--	LA	485	501	96.8%	4
Yang ZJ, 2012 [459]	2010/03-2010/06	Rui Li	Yunnan	Southwest	Entertainment	--	P1M	448	501	89.4%	4
He B, 2011 [444]	2011/04-2011/05	An Ning	Yunnan	Southwest	Entertainment	--	LA	45	47	95.7%	4
He B, 2011 [444]	2011/04-2011/05	An Ning	Yunnan	Southwest	Entertainment	--	LA	233	273	85.3%	4

First author, published year	Study period	Location	Province	Region	Recruitment venue	Sampling method	Measurement period*	Number of FSW used condom	Total number of FSW	Condom Usage (%)	QA Score
He B, 2011 [444]	2011/04-2011/05	An Ning	Yunnan	Southwest	Entertainment	--	LA	29	33	87.9%	4
He B, 2011 [444]	2011/04-2011/05	An Ning	Yunnan	Southwest	Entertainment	--	LA	159	193	82.4%	4
Li Y, 2012 [458]	2011	Lan Cang	Yunnan	Southwest	Entertainment	--	LA	389	400	97.3%	3
Li Y, 2012 [458]	2010-2011	Lan Cang	Yunnan	Southwest	Entertainment	--	LA	667	701	95.1%	3
Li Y, 2012 [458]	2010-2011	Lan Cang	Yunnan	Southwest	Entertainment	--	P1M	638	701	91.0%	3
Li YK, 2012 [446]	2011	--	Sichuan	Southwest	--	--	LA	1200	1339	89.6%	2
Li YK, 2012 [446]	2011	--	Sichuan	Southwest	--	--	P1M	678	1339	50.6%	2
Ou YH, 2012 [460]	2011/04-2011/06	He Jiang	Sichuan	Southwest	Entertainment	--	LA	324	400	81.0%	4
Ou YH, 2012 [460]	2011/04-2011/06	He Jiang	Sichuan	Southwest	Entertainment	--	P1M	215	400	53.8%	4
Yang JF, 2012 [425]	2011/01-2011/07	Bao Shan	Yunnan	Southwest	Entertainment	--	LA	1328	1409	94.3%	4
Yang JF, 2012 [425]	2011/01-2011/07	Bao Shan	Yunnan	Southwest	Entertainment	--	P1M	1232	1409	87.4%	4
Zhang H, 2012 [461]	2011	Zhen Yuan	Yunnan	Southwest	--	--	LA	249	269	92.6%	2
Zhang H, 2012 [461]	2011	zhen yuan	Yunnan	Southwest	--	--	LA	1184	1301	91.0%	2
Zhang H, 2012 [461]	2011	Zhen Yuan	Yunnan	Southwest	--	--	LA	207	228	90.8%	2
Zhang H, 2012 [461]	2011	Zhen Yuan	Yunnan	Southwest	--	--	LA	728	804	90.5%	2
Zhang H, 2012 [461]	2011	Zhen yuan	Yunnan	Southwest	--	--	P1M	1101	1301	84.6%	2
Zhang H, 2012 [461]	2011	Zhen Yuan	Yunnan	Southwest	--	--	P1M	223	269	82.9%	2
Zhang H, 2012 [461]	2011	Zhen Yuan	Yunnan	Southwest	--	--	P1M	680	804	84.6%	2
Zhang H, 2012 [461]	2011	Zhen Yuan	Yunnan	Southwest	--	--	P1M	198	228	86.8%	2

*LA: last sex act; P1M: in the past one month prior to the survey

References

1. Qiu C, Chen XG, Chen GW, Guo J. [Analysis on epidemiological survey and sentinel surveillance of HIV/AIDS in Ningde City]. Disease Surveillance. 2006;21(12):635-7.
2. Chen Y, Cai X, You T, Chen Q. [Analysis on risk behaviors and HIV/syphilis infection among female sex workers(FSWS) in detention home of Longyan city from 2001 to 2007]. Preventive Medicine Tribune. 2009;15(09):827-8.
3. Du Y, Yang H, Jian P, Qian W. [Study on the effectiveness and sustainability of implementing 100% condom use programme in entertainment establishments]. Jiangsu Journal of Preventive Medicine. 2004;15(3):31-3.
4. Li L, Ding JP, Du YP, Chen GH, Yang HT. [Analysis on baseline study on Jiangsu / WHO 100% condom use program to prevent STD & AIDS]. Jiangsu Journal of Preventive Medicine. 2003;14(3):19-21.
5. Tang X, Chen X, Li T, Ni Y, Yan J, Huang X. [Analysis of STD/AIDS behaviour intervention among entertainment service persons]. Modern Preventive Medicine. 2003;30(2):275-7.
6. Qiu C, Cao S. [A study of effectiveness of HIV high-risk behavioural interventions among entertainment-based female sex workers in Ningde prefecture, China]. Strait Journal of Preventive Medicine. 2003;9(6):56-7.
7. Xiao Y, Zhang H, Wei X, Hu Y, Yang Z. [A Survey on HIV awareness and Characteristics of Sexual Behavior 201 Women Engaging in Clandestine Prostitution]. Chinese Journal of STD & AIDS Prevention and Control. 2002;8(5):296-8.
8. Liao M, Liu X, Qian Y, Kang D, Fu J. [A Survey on AIDS Knowledge among Seven Population Groups in Shandong Province]. Preventive Medicine Tribune. 2007;13(5):404-6.
9. Chen YL, Chen QJ, Zhang YH, Li SR, Lin MY. [Seroepidemiological and behavioural study among prostitutes Longyan City of Fujian Province, 2004]. Occupation and Health. 2005;21(12):132-3.
10. Du Y, Qian W, Shao T. [Analysis on results of behavior surveillance and serum examination among female sex workers in entertainment establishments]. Jiangsu Journal of Preventive Medicine. 2006;17(03):12-5.
11. Li X, Guo D, Hu Y, Li Y, Wang Y. [Cross Sectional Study on Knowledge of AIDS and Sexual Behavior Among Female Sexual Workers in Public Places]. China and Foreign Medical Journal. 2008:12-4.
12. He J, Wu Z, Dou Z. [A survey on HIV awareness and characteristics of sexual behavior among CSWs in public places of entertainments in Wuhu city]. Anhui Journal of Preventive Medicine. 2005;11(5):274-7.
13. Jin T, Yan J, Ma Q, Pan X, Chen W. [Survey on status Quo of Knowledge about and behaviors of AIDS prevention and control of unlicensed prostitutes in the entertainment establishments in the city of Lishui]. Disease Surveillance. 2005;20(12):621-4.
14. Li J, Kang D, Tao X, Zhen W, Zhang N, Zhu X, et al. [Effectiveness evaluation of HIV interventions among female sex workers in Shandong province during 2004-2008]. Preventive Medicine Tribune. 2010;v.16(03):193-5.
15. Li X, Zhang B, Liu M, Zhang N, Sun B, Zhang P. [Survey on the behavioral characteristics related to HIV/AIDS and the sero prevalence of sexually transmitted disease (STD) among 466 female sex workers (FSW) in Qingdao]. Chinese Journal of AIDS & STD. 2006;12(1):16-8.

16. Li X, Han H, Zhang X, Li Z. [Analysis on the results from CSWs integrating surveillance in Hefei city in 2004]. Chinese Journal of Disease Control & Prevention. 2005;9(6):580-3.
17. Liu XZ, Liao MZ, Fu JH, Su SL, Huang T. [Analysis on HIV/AIDS Surveillance of Shandong Province in 2004]. Preventive Medicine Tribune. 2006;12(1):80-2.
18. Shen YG, Gu XJ. [Investigation on HIV infection, knowledge, attitude, behavior and practice among female sex workers in rural areas]. Chinese Journal of AIDS & STD. 2006;12(6):560, 6.
19. Xiao H, Fu Y, Xiong Z, Xu D, Li J, Tang X. [Evaluation on Effects of Venerism Prevention and AIDS Interference on Female Sex Workers]. Mod Diagn Treat. 2007;18(5):277-81.
20. Xie Y, Chen JH, Wang ZH. [A survey on the characteristics and HIV, syphilis infection among prostitutes in re-education center in Zhangzhou City, Fujian Province]. Strait Journal of Preventive Medicine. 2006;12(4):38-9.
21. Xiong ZW, Li J, Xu D, Lai XM, Mei J. [Intervention study of HIV high-risk behavior among commercial sex workers]. Modern Preventive Medicine. 2005;32(12):1717-8, 21.
22. Yang JZ, Pan XH, Zou Y, Li XT, Yang Q, Xu Y. [Analysis on Behavior Surveillance of AIDS in Zhejiang Province in 2004]. Zhejiang Journal of Preventive Medicine. 2005;17(11):13-5.
23. Zheng H, Ding Y, Fu QF, Wang YJ. [Effect of Intervention to Commercial Sex Workers in Comprehensive Prevention Demonstrative Area]. Jiangsu Health Care. 2012;14(1):8-10.
24. Zhu CQ, Wu JH, Fu LJ, Guo TY, Pan NY, Lu QL, et al. [A survey on AIDS knowledge and related risk behavior among female sex workers in public entertainment venues in Shaoxing City, Zhejiang Province]. Chinese Journal of AIDS & STD. 2006;12(2):166,8.
25. Zhu X, Kang D, Liu X, Liao M, Fu J. [Analysis of behavioral changes among clandestine prostitutes in Jiaozhou city]. Chinese Journal of AIDS & STD. 2008;14(1):28-30.
26. Chen FW, Wang YN, Liu J, Yang XL, Wang QR. [Evaluation on the effectiveness among female sex workers in entertainment venues]. Practical Clinical Medicine. 2007;8(2):128-30.
27. Fu S, Sun L, Meng X, Ding Z, Wang L. [Surveillance of AIDS among female sex workers in China, 1995-2009]. Disease Surveillance. 2010;v.25(11):850-3.
28. Jiang X, Zheng X, Zhang L, Yu H, Yang J, Jiang B. [Surveillance of HIV infection among HIV high risk population in Liaocheng city]. Journal of Shandong University (Health Sciences). 2006;44(7):710-3.
29. Ruan S, Zhang C, Shi Z, Wang C, Pan R, Yang H, et al. [Evaluation on the Effect of Health Education on AIDS Prevention among Commercial Sexual Workers in Public Places of Entertainment in Ji'nan City]. Preventive Medicine Tribune. 2007;13(11):974-5, 80.
30. Wang J, Liu ZL, Wang B, Yu WX, Wang WB. [Investigation on HIV/STD knowledge, attitude, practice among female sex workers in entertainment venues in Jianhu County, Jiangsu Province]. Jiangsu Journal of Preventive Medicine. 2008;19(3):24-7.
31. Wang LW, Li R, Xu XW. [Analysis on HIV infection trend among female sex workers in entertainment establishments]. Practical Clinical Medicine. 2006;7(7):144-6.
32. Wang Y, Liao Q, Chen H, Gong J. Baseline analysis of HIV high-risk behaviours among drug users and female sex workers in Jiangxi province. Journal of Public Health and Preventive Medicine. 2006;17(05):60-1.

33. Wu J. [Effectiveness of HIV interventions for entertainment-based female sex workers]. Zhejiang Journal of Preventive Medicine. 2010;22(12):84-5.
34. Xu X, Lan J, Chen X, Lan L, Ban X. [A survey on AIDS- related knowledge and sexual behavior s among female sex workers in Jingning County of Zhejiang Province]. Disease Surveillance. 2007;22(4):240-1,50.
35. Yan H, Chen G, Cao G, Ding P, Shi P. [Analysis of baseline survey on expanding behavior interventions programme at entertainment establishments in Jiangsu province]. Jiangsu Journal of Preventive Medicine. 2007;18(1):12-5.
36. Yu JF, Pan QF, Wang KZ. [A survey on HIV/AIDS behavior surveillance among prostitutes in Cixi City of Zhejiang Province, 2005]. China Preventive Medicine. 2007;8(3):265-6.
37. Zhao XP, Que JL, Liu YC, Mi HH, Zhao YQ, Cao XP, et al. [An investigation of HIV/AIDS prevention and knowledge among female working in entertainment venues in Suzhou City, Jiangsu Province]. Shanghai Journal of Preventive Medicine. 2006;18(11):561-2.
38. Zhu LD, Xu L, Zhu Y. [Survey of knowledge, attitude and behavior toward AIDS in jailed sexual workers]. China Tropical Medicine. 2007;7(1):3.
39. Cai X, Jiang B, Jiang X, Zhang S, Yu H, Hu H. [Evaluation on AIDS risk behavioral intervention among female commercial sex workers in public entertainment places]. Chinese Journal of Public Health. 2007;23(11):1355-6.
40. Cai XF, Yang MX. [Survey on status of knowledge and behaviors of AIDS/STD prevention and control of hostesses in 85 recreational places]. Shanghai Journal of Preventive Medicine. 2007;19(5):218-9.
41. Zha YF, Zhen L, Qian JL, Huang ZM, Lu LJ. [Evaluation of HIV/AIDS behavioral interventions among female sex workers in Songjiang District in Shanghai]. Shanghai Journal of Preventive Medicine. 2008;20(9):434-5.
42. Feng C, Ji GP, Wang HD, Su J, Xu LF, Leng J. [Investigation on AIDS knowledge and behaviors among commercial sex workers in Anhui province]. Chinese Journal of Public Health. 2007;23(12):1430-1.
43. Gu Y, Ding X, Chen X. [Survey on attitude to voluntary HIV test results disclosure among female sex workers in Funan County of Anhui Province, China]. Chinese Journal of Health Education. 2007;23(7):502-4.
44. Hu X. [Comprehensive AIDS surveillance among female sex workers in Huabei city, Anhui province]. Disease Surveillance. 2009;24(2):97-8.
45. Huang Y, He YX, Wang Y, Han CM, Wang DF. [Evaluation of AIDS intervention program based on sexually transmitted disease clinics among female sex workers]. Chinese Journal of AIDS & STD. 2012;18(8):543-6.
46. Jiang G, Li H, Zhu Y. [An analysis of result of AIDS/STD behavioral surveillance among commercial sex workers]. Journal of Chinese Medicine Research. 2007;7(3):270-1.
47. Li T, Li M, Kong X, Mao X, Wang S. [Model Study on Two-Way Intervention Between Commercial Sex Workers and Their Clients]. Journal of Preventive Medicine Information. 2009;25(3):177-80.
48. Liao M, Bi Z, Liu X, Kang D, Fu J, Song Q, et al. Condom use, intervention service utilization and HIV knowledge among female sex workers in China: results of three consecutive cross-sectional surveys in Shandong Province with historically low HIV prevalence. International journal of STD & AIDS. 2012;23(3):e23-9.
49. Liu S, An B. [Survey on AIDS related knowledge and behavior in sex workers at recreation places]. Chinese Journal of Public Health. 2008;24(01):18-9.

50. Luo JF. [Analysis of HIV/AIDS surveillance in Wuyishan City in Fujian Province, 2002-2006]. Chinese Journal of Ethnomedicine and Ethnopharmacy. 2009;18(13):71-2.
51. Luo Y, Chen SC, Ding JM, Cheng J, Xu K, Yuan H, et al. [Analysis of HIV/AIDS sentinel surveillance in Hangzhou]. Disease Surveillance,. 2008;23(11):717-9.
52. Luo Z, Zha YF, Huang ZM. [Analysis on surveillance of secret prostitutes in Songjiang district in year 2006 Shanghai]. Shanghai Journal of Preventive Medicine. 2007;19(4):184-5.
53. Qi G, Zhou X, Yan H, Yu R, Hu X. [Baseline survey on STD/ AIDS knowledge, attitude and practice among female sex workers]. Chinese Journal of Public Health. 2007;23(7):861-2.
54. Lu F, Jia Y, Sun X, Wang L, Liu W, Xiao Y, et al. Prevalence of HIV infection and predictors for syphilis infection among female sex workers in southern China. Southeast Asian J Trop Med Public Health. 2009;40(2):263-72.
55. Sun Z, Xue F, Lin S, Wen M. [Survey of knowledge and behavior associated with AIDS in prostitutes in educational house]. China Tropical Medicine. 2008;8(03):511-2.
56. Wang Y, Li X, Zhang B, Wang L, Liu M, Wu D. [Factors associated with HIV/AIDS STD among young female sex workers in Qingdao]. China J Lepr Skin Dis. 2008;24(06):424-6.
57. Wu J, Wang W, Wang F. [The effect of behavior intervention among the commercial sec workers in entertainment places of Yingtan City]. Journal of Preventive Medicine Information. 2006;22(6):647—9.
58. Xu SH, Chen AX, Liu MH. [Analysis on HIV risk factor among female sex workers in Southeast region]. Chinese Journal of AIDS & STD. 2007;13(3):277.
59. Yu X. [Survey on the knowledge, attitude, and behavior related to HIV/AIDS among prostitutes in the entertainment establishments in Haimen city]. Journal of Public Health and Preventive Medicine. 2007;18(2):104-5.
60. Zhang C, Ruan S, Shi Z, Yang H, Zhu Y, Jia Z, et al. [Survey of HIV/AIDS related knowledge and behavior among high risk population Jinan city]. Chinese Journal of AIDS & STD. 2008;76(01):55-7.
61. Zhang XJ, Liao MZ, Kang DM, Tao XR, Qian YS, Wang GR, et al. [Condom Use and Correlates Among Female Sex Workers in Shandong Province,2006-2008]. Preventive Medicine Tribune. 2012;18(6):405-7+10.
62. Zhu F, Ji K, Li Z, Xu x, Wang R. [Evaluation on the Effect fo AIDS/STD Related Knowledge Training and Behavior Intervention among Female Sex Workers in Entertainment Places]. Preventive Medicine Tribune. 2009;v.15(05):420-2.
63. Zhu H, Cao D, Ma Z, Xu X, Yan H, Zhu M, et al. [Effective analysis of STD/ AIDS high 2risk behaviour intervention among commercial sex workers in Maanshan]. Chinese Journal of Disease Control & Prevention. 2008;12(06):534-7.
64. Chen SP, Tu BY, Wang X. [Survey and Analysis on HIV/AIDS-Related Behavior and Recognition among HIV/AIDS High-risk Population of Xunyang District Jiujiang City]. Chinese Journal of Evidence-Based Medicine. 2010;10(7):817-21.
65. Cheng X, Xiao Y, Wang H. [Analysis on HIV/AIDS knowledge and condom use of mid-low-end female sex workers]. Anhui Journal of Preventive Medicine. 2008;20(14):410-2.

66. Cui W, Liu J, Yang L. [Changes of AIDS related knowledge and behaviors among FSWs in Lixin County from 2007 to 2008]. Anhui Journal of Preventive Medicine. 2009;30(9):1048-50.
67. Fan H, Wu J, Song Q, Ruan Y, Li Y. [Investigation on Sexually Transmitted Disease and AIDS knowledge and risk behavior of female sex workers in certain district of Shanghai]. Community Healthcare. 2008;7(4):291-2.
68. Guo Z, Wan D, Feng C, Cai L, Fan Y. [Trend analysis on knowledge and behavior of AIDS prevention among the CSWs in Mengcheng County]. Chinese Journal of Disease Control & Prevention. 2009;13(04):411-3.
69. Li N, Li Z, Fan J, Huang Z, Zeng Z, Xu S. [Analysis on condom use and its determinants among female sex workers in cheap entertainment places in Hefei]. Chinese Journal of Disease Control & Prevention. 2009;13(4):417-9.
70. Liao MZ, Liu XZ, Fu JH, Qian YS, Wang TZ. [Analysis of HIV/ AIDS Surveillance Data in Shandong Province in 2007]. Preventive Medicine Tribune 2008;14(12):1143-5.
71. Liu Y, He J, Deng W. [HIV transmission and behavioural characteritics among female sex workers in Jizhou district, Jilin City, 2007]. Journal of Jinggangshan Medical College. 2007;14(06):41-2.
72. Luo Y, Chen S, Xu K, Yuan H, Chen J, Hu J, et al. [Survey of STD/AIDS-related knowledge, behaviors and infection rates of sex workers in entertainment places in Hangzhou]. Disease Surveillance. 2008;23(10):607-9.
73. Ni YQ, Wang ZY, Shi CL. [Investigation on HIV/AIDS knowledge, attitude and practice (KAP) among female sex workers in three small-sized entertainment venues in Changning District, Shanghai]. Shanghai Journal of Preventive Medicine. 2008;20(11):537-8.
74. Peng B, Jiang M, Wu GZ, Sun YM, Ke CB. [Analysis on HIV epidemic among three high risk populations]. Chinese Journal of Disease Control & Prevention. 2008;12(6):631-2.
75. Wang L, Gu B, Yang H. [Investigation on HIV/AIDS knowledge and behaviors among female sex workers in entertainment venues in Yixing City, Jiangsu Province]. Shanghai Journal of Preventive Medicine. 2008;20(10):493-4.
76. Wang W, Xue L, Xia L, Tang Q, Shen L. [Survey of 297 sex workers and the infectious status of STD and HIV/AIDS]. China Tropical Medicine. 2008;8(05):827-8.
77. Wang XM, Liu XH. [Study on the HIV detection and related behavior among FSWs]. Zhejiang Journal of Preventive Medicine. 2011;23(7):37-8+42.
78. Xia J, Yu G, Zhou X, Wang X, Zhou H. [Investigation on AIDS related Knowledge, Attitude and Behavior among Female Sex Workers in Entertainment Place of Xihu District from 2007 to 2008]. Preventive Medicine Tribune. 2010:120-1.
79. Zhang Q, Jiang X, Jiang B, Wang X, Peng H, Long D, et al. [Analysis on High-risk Behaviors Related to AIDS of CSW in Nanchong City]. Journal of Preventive Medicine Information. 2006;22(1):28-32.
80. Zhou ZL, Que JL, Liu YC, Mi HW, Zhao YQ, Cao XP, et al. [A baseline investigation on female sex workers in entertainment venues]. Jiangsu Journal of Preventive Medicine. 2008;19(2):25-6.
81. Chen JH, Fang JA, Zhang YF, Xie ZJ. [Investigation on HIV/STD infection and related risk behaviors among roadside female sex workers]. Modern Preventive Medicine. 2009;21(3):280-1.

82. Chen L. [Analysis on the surveillance in observation sites of AIDS and venereal diseases in year 2008 Cong Ming county Shanghai municipality]. Shanghai Journal of Preventive Medicine. 2009;21(2):83.
83. Gong P, Wang H, Zhou H, Wu M, Chen C, Sang Q, et al. [Investigation of Awareness Situation on AIDS Knowledge Among CSWs in Anhui Province!]. Anhui Journal of Preventive Medicine. 2009;15(06):409-10+16.
84. He X. [Evaluation on AIDS comprehensive intervention program among CSWs in Yuyao City]. Zhejiang Journal of Preventive Medicine. 2012;24(11):21-3.
85. Hu CC, Yuan ZK, Liu Y. [HIV/AIDS Intervention Among Female Sex Workers in Different Places in Nanchang City]. Journal of Nanchang University(Medical Science). 2011;51(2):74-7.
86. Jin Y, Yao Y, Ye D, He J, Dou Z, Qi S, et al. [Investigation and analysis on condom use status female commercial sex workers]. Chinese Journal of Disease Control & Prevention. 2009;13(01):20-2.
87. Liao M, Jiang Z, Zhang X, Kang D, Bi Z, Liu X, et al. Syphilis and methamphetamine use among female sex workers in Shandong Province, China. Sex Transm Dis. 2011;38(1):57-62.
88. Liao M, Nie X, Pan R, Wang C, Ruan S, Zhang C, et al. Consistently low prevalence of syphilis among female sex workers in Jinan, China: findings from two consecutive respondent driven sampling surveys. PLoS One. 2012;7(4):e34085.
89. Liu ZL, Ge MH. [Condom Use and Its Impact Factors in Female Sex Workers]. Chinese Primary Health Care. 2011;25(6):79-81.
90. Lu Q, Chen K, Fu L, Shan X. [Comprehensive analysis of sentinel surveillance results among female sex workers in urban Shaoxing City, China] Zhejiang Journal of Preventive Medicine. 2009;21(6):28-9.
91. Tang X, Wu P, Li Y, Zhong Y, Yu X, Pan R, et al. [A study on AIDS related knowledge and behavioral characteristics among mini-type entertainment venues based female sex workers in Hongkou district of Shanghai]. China Preventive Medicine. 2010;11(02):162-5.
92. Tang X, Li Y, Li LY, Pan R, Zhong XY, Yu XN, et al. [The assessment of HIV/AIDS structural intervention program among female sex workers in small entertainment settings in Hongkou District of Shanghai]. Chinese Journal of AIDS & STD. 2012;18(10):669-72.
93. Wang F, Chen X, Su B, Ji G. [Analysis of the results of the comprehensive HIV/AIDS surveillance among sex workers in Anhui]. Anhui Journal of Preventive Medicine. 2009;15(06):407-8.
94. Xue F. [Survey of AIDS-related knowledge and infection rates of HIV, HBsAg and Syphilis among female sex workers in entertainment places in Lucheng district of Wenzhou city]. Chinese Journal of Health Laboratory Technology. 2009;19(7):1649-51.
95. Yang Y, Yao J, Gao M, Su H, Zhang T, He N. Herpes simplex virus type 2 infection among female sex workers in Shanghai, China. AIDS Care. 2011;23 Suppl 1:37-44.
96. Zhang YH, Bao YG, Li CM, Han L, Sun JP, Tan HZ. [Study of HIV/ syphilis infection status of commercial sex workers in 15 cities of China]. China Preventive Medicine. 2011;12(5):387-90.
97. Zhao YQ, Zhao XP, Cao XP, Zhang FX, Shao MR. [AIDS high-risk behaviour and STD infection rates: Investigation on 396 demimondaines]. Shanghai Journal of Preventive Medicine. 2010;v.22(03):137+41.
98. Zhou XM, Yu M, Chi H, Zhang B. [Effect of HIV/AIDS high risk behavioral intervention among prostitutes in China]. Chinese General Practice. 2010;13(240):25-6.

99. Cai Y, Shang M, Shen T, Pei B, Jiang X, Huang H, et al. [Awareness and behavioral research of AIDS among female sex workers in small entertainment venues in Shanghai]. Journal of Shanghai Jiaotong University (Medical Science). 2010;30(8):890-3.
100. Chen GS, Wu HS, Yao ZM. [Investigation on HIV/AIDS related knowledge, behavior and infection among commercial female sex workers in one county]. Anhui Journal of Preventive Medicine. 2010;16(5):418-9.
101. Chen SX, Zhang ML, Han XM. [Survey on AIDS Related Knowledge, Behavior and the HIV Infection Status Among Commercial Sex Workers in Gaomi City]. Preventive Medicine Tribune 2011;17(12):1119-20, 23.
102. Chen ZH, Xie JR, He J, Ren XP. [Study on HIV/AIDS detection and related knowledge among female sex workers in Zunji]. Zhejiang Journal of Preventive Medicine. 2012;24(3):81-2.
103. Gan WH, Zhu JM, Jiang CH, Huang RR, Chen L. [Analysis of AIDS sentinel surveillance among the female sex workers in Jinshan district of Shanghai from 2009-2011]. Chinese Journal of AIDS & STD. 2012;18(7):457-8+74.
104. Jin HJ, Fang JA, Liu JM, Zhang YF, Pan PY. [Sentinel surveillance analysis on female sex workers in Zhenhai County in Ningbo City]. Shanghai Journal of Preventive Medicine. 2010;22(8):405-6.
105. Kang D, Liao M, Jiang Z, Zhang X, Mao W, Zhang N, et al. Commercial sex venues, syphilis and methamphetamine use among female sex workers. AIDS Care. 2011;23 Suppl 1:26-36.
106. Liao MZ, Liu XZ, Kang DM, Fu JH, Wang TZ, Qian YS, et al. [Analysis on the HIV/AIDS Surveillance Data in Shandong Province in 2009]. Preventive Medicine Tribune. 2010;16(5):398-400, 3.
107. Luo Y, Bao Q, Zhang X. [Exploration About AIDS Interventional Model Among Female Sex Workers In Entertainment Places]. Anhui Journal of Preventive Medicine. 2010;16(4):256-9.
108. Shao M, Shen J, Zhao X, Zhao Y, Zhang F, Cao X. [Investigation on AIDS and Venereal disease infection among CSW crowd in Suzhou Area]. Occupation and Health. 2010;26(8):886-7.
109. Wan LJ, Zhang XX, Gu XM. [A study on HIV/AIDS-related knowledge, attitudes and practices among female sex workers working at different venues]. Zhejiang Journal of Preventive Medicine. 2011;23(5):81-2, 5.
110. Wang F, Chu Y, Wang L, Wang F. [Serological survey and investigation on AIDS knowledge and behavior among CSW in Huaiyuan county]. Anhui Journal of Preventive Medicine. 2010;16(03):190-1+202.
111. Wei Z, Sun L. [Investigation on behavior and infection of STDs and AIDS among female sex workers at entertainment sites in Yangzhou city]. Preventive Medicine Tribune. 2010;16(12):1123-5.
112. Xi SJ, He YF, Zhou XH. [Analysis on the Result of AIDS Test Intervention in CSW, MSM, IDU Population in Xiacheng District]. Zhejiang Journal of Preventive Medicine. 2010;22(9):29-30.
113. Xi SJ, He YF, Zhou XH, Zhou DD, Wang CC. [A Survey on the Status and Wishes of HIV Voluntary Counseling and Testing and Its Influencing Factors among Community Female Commercia l Sex Workers]. Zhejiang Journal of Preventive Medicine. 2011;23(1):8-10+6.

114. Xi S, He Y, Zhou X, Zhou D, Wang C. [A Survey on the status and wishes of HIV voluntary counseling and testing and its influencing factors among community female commerical sex workers]. Zhejiang Journal of Preventive Medicine. 2011;23(1):8-10, 6.
115. Zhang QQ, Huan XP, Yin YP, Wang XL, Hu HY, Jiang N, et al. [Incidence rates of sexually transmitted infection and the characteristic analysis of female sex workers failing in follow-up in cohort study]. Acta Universitatis Medicinalis Anhui. 2012;47(9):1050-4.
116. Yao X, Ying YL, Xu S, Chen ZW, Huang CW, Lu Y, et al. [Evaluation on AIDS high-risk behaviors intervention among female sex workers in entertainment places of Fuzhou]. Modern Preventive Medicine. 2010;37(21):4094-6.
117. Chen CC, Lin H, Zhang H. [AIDS knowledge Levels and Behavior Characteristics of 225 Female Sex Workers in Entertainment Places of Fuzhou City]. Occupation and Health. 2011;27(2):170-2.
118. Jiang J, Wang HB, Fang WM, Sun JL, Chen BB, Bo DY, et al. [Survey of AIDS prevalence in female sex workers detained in a correctional facility in Ningbo, Zhejiang]. Disease Surveillance. 2012;27(08):634-6.
119. Kang DM, Tao XR, Li JZ, Liao WZ, Zhu XH, Zhang H, et al. [Evaluation of AIDS intervention among female sex workers in Global Fund Project counties in Shandong Province]. Journal of Shandong University (Health Sciences). 2011;49(10):155-9.
120. Liu LL, Yin J. [Analysis of sentinel monitoring on FSWs in Jiangyen City in 2010]. Chinese Primary Health Care. 2011;25(8):96-7.
121. Ma P, Chen DL, Yuan JM. [Survey on syphilis prevalence and impact factors among female sex workers in Nantong, 2010]. Chinese Journal of AIDS & STD. 2011;17(5):561-2+76.
122. Miao XL, Cheng H, Zhang X, Gu J, Ji YY, He EQ. [Analysis on HIV /AIDS Sentinel Surveillance in Wuxi City in 2010]. Occupation and Health. 2011;27(22):2599-601.
123. Qian ZH, Wang J, Fan XQ. [HIV/AIDS sentinel surveillance results among prostitutes in Suzhou city]. Jiangsu Journal of Preventive Medicine. 2012;23(1):30-1.
124. Sun XQ, Tang GX, Mao TS, Zhang YJ, Mao N. [2010 baseline survey among commercial sex workers of Taihe County]. China Modern Medicine. 2011;18(28):151-3.
125. Wu SB, Chen G, Pan WJ, Lin YT, Zheng WX, Lin L, et al. [Survey on AIDS-related Knowledge and Behavior among Commercial Sex Workers in Fujian Province]. Chinese Journal of Social Medicine. 2012;29(4):264-5.
126. Chen G, Wu ZH, He X, Wang W. [Effect evaluation of the AIDS health education for CSW in 3 cities]. Chinese Journal of Health Education. 2012;v.28(08):682-4+7.
127. Ling Z, Wu JJ. [Survey on AIDS-related Knowledge and Behaviour among CSWs in Entertainment Venues]. Zhejiang Journal of Preventive Medicine. 2012;24(12):87-8.
128. Pan GL, Jin Y, Dong XJ. [Status of STDs Epidemic Among 617 Commercial Sexual Workers]. Zhejiang Journal of Preventive Medicine. 2012;24(2):72-3.
129. Qin CZ, Yin J, Liu.Y., Zhang W. [Analysis on AIDS Infection and Influencing Factors Among Female Sexual Workers in Jiangyen City]. Acta Universitatis Medicinalis Nanjing (Natural Science). 2012;32(12):1784-6+9.
130. Qin QR, Su B, Xu FN, Zhu HB, Zhang Z, Gao Y, et al. [Analysis on effects of the AIDS/reproductive tract infections intervention to female sex workers in low-class establishments of Maanshan] Chinese Journal of Disease Control & Prevention. 2012;16(12):1056-9.
131. Qin QR, Su B, Xu FN, Zhu HB, Zhang Z, Gao Y, et al. [Survey on KABP of AIDS / reproductive tract infections among female sex workers in low-class establishments of Maanshan]. Chinese Journal of Disease Control & Prevention. 2012;v.16(09):771-3.
132. Qiu ZH, Dong ZQ, Jin MH, Yang ZR. [Sentinel surveillance of AIDS among female sex workers in Huzhou,Zhejiang, 2011]. Disease Surveillance. 2012;27(4):291-3.

133. Sun BJ. [Survey on AIDS-related Knowledge, Behavior and HIV-infection Among Commercial Sex Workers in Shizhong District, Zaozhuang City, 2011]. Preventive Medicine Tribune. 2012;18(9):654-6.
134. Tao SF, Zheng YJ. [Analysis of the characteristics of sexual behavior and syphilis infection among high risk population in Zongyang county, Anhui province]. Anhui Journal of Preventive Medicine. 2012;18(3):195-7.
135. Wang DL, Zhu YS, Gu CY, Jiang W, Feng LJ. [Analysis of sentinel monitoring of CSWs in Zhonglou district of Changzhou city]. Jiangsu Journal of Preventive Medicine. 2012;23(3):47-8.
136. Yang YR, Du JH, Duan XY, Kang Y, Wu LX, Meng R. [A study on HIV and syphilis infection and related risk behaviours among female sex workers working at entertainment venues in Baotou city of Inner Mongolia Automous Region, 2006-2009]. Chinese Journal of AIDS & STD. 2012;18(02):128-9.
137. Xu JS, Liu XY, Fu GF, Huan XP, Li L, Xu XQ, et al. [Syphilis and HIV Infection Status Among Female Sex Workers in Jiangsu Province]. The Chinese Journal of Dermatovenereology. 2012;26(6):513-5.
138. Yang YH, Gong CT, Wang ZQ, Chen CY. [Analysis of national sentinel monitoring of AIDS infection in Quanzhou City in 2011]. Strait Journal of Preventive Medicine. 2012;18(6):32-3.
139. Ye ZM, Wang DY, Zhang HM, Zhao LN, XUE FH, Jin Q, et al. [Survey on HIV/AIDS -related knowledge and behaviors among FSWs]. Zhejiang Journal of Preventive Medicine. 2012;24(11):74-6.
140. Zhu HW, Huang SP, Zhu RH, Zhan MW, Yan W. [Analysis of AIDS Sentinel Surveillance in Nanchang County in 2011]. Chinese Community Doctors. 2012;14(32):338.
141. Song ZP. [Evaluation on the effect of HIV/AIDS prevention intervention programs among bargirls in Yingze District of Taiyuan City, Shanxi] Chinese Journal of Public Health Management. 2004;20(5):457-9.
142. Ren X, Bo F, Bao Z, Xu R, Zhou B, Liu X, et al. [A study of HIV and syphilis infection and related behaviours among female sex workers in Hohhot, China]. Chinese Journal of AIDS & STD. 2006;12(06):551-2.
143. Zhao RL, Zhang XM, Lin Z, Li ZM, Wang J, Wang Y, et al. [Report on HIV surveillance among female sex workers in the entertainment establishments in Tongliao City, Inner Mongolia]. Chinese Journal of AIDS & STD. 2005;11(3):214.
144. Zhao Y, Li G, Xia D, Chu T, Xu M, Liu G, et al. [A behavioral intervention study on STD/AIDS prevention for young female sex workers in Beijing]. Modern Preventive Medicine. 2007;34(10):1936-8.
145. Bai JM, Shi WY, Zhu LY, Qu YM. [Study on behaviours and related diseases among 114 female sex workers in Fengtai District in Beijing]. Chinese Journal of AIDS & STD. 2006;12(3):264.
146. Li G, Xia D, Lu H, Yang Y, Gao J, Zhang M, et al. [Study on AIDS related risk factors and behaviors among female commercial sex workers]. China Preventive Medicine. 2008;9(1):1-4.
147. Lin Z, Liu GY, Li ZM, Zhao RL, Zhang XM, Chen Y, et al. [Analysis of HIV surveillance among 364 female sex workers in Tongliao City, Inner Mongolia]. Chinese Journal of Public Health. 2007;23(3):355.

148. Wang S, Shi C, Mu S. [Shanxi GF3 region 2005 - 2008 survey of CSWs knowledge and behavior]. Chinese Journal of Disease Control & Prevention. 2009;13(4):405-7.

149. Bai JM, Shi WY, Xie HY, Qu YM, Zhai CX, Tian ML, et al. [Prevalence of high-risk behavior and HIV infection among bargirls in Fengtai District of Beijing]. The Chinese Journal of Human Sexuality 2007;16(6):45-6, 8.

150. Bao F, Gao P, Yun Z, Ren X, Liu X, Guo S. [An AIDS epidemiological survey of female sex workers crowd in Hohhot]. Inner Mongolia Medical Journal. 2007;39(08):971-3.

151. Deng PX, Zhang SQ, Cheng CW, Wang CF, Chu TX, Hu Y. [Study on a feasible model of integrated interventions to promote condom use in high-risk sites]. Chinese Journal of AIDS & STD. 2008;14(4):382-4.

152. Jia HZ, Sun Y. [Monitoring and analysis of AIDS among female sex workers in Minyun County of Beijing from 2006-2011]. Occupation and Health. 2012;28(13):1614-5+8.

153. Liu HX, Ma SB, Li F, Wang HS, Hou Z. [Analysis of AIDS sentinel surveillance among commercial sex workers in Changping District from 2005-2010]. Chinese Journal of AIDS & STD. 2011;17(5):579-80.

154. Liu L, Liu M, Lu H, Xia D. [Analysis of HIV/AIDS related risk behaviors among female sex workers at entertainment establishments in two districts of Beijing]. Chinese Journal of AIDS & STD. 2007;13(6):532-5.

155. Shi WY, Xie YY, Liu C. [Behavioral and serological surveillance among female sex workers in Fengtai district, Beijing from 2006-2009]. Chinese Journal of Public Health. 2012;28(01):109-10.

156. Shi Y, Guo S, Bo F, Zhang X, Cao W, Wang P. Impact evaluation of a sexually transmitted disease preventive intervention among female sex workers in Hohhot, China. Int J Infect Dis. 2013;17(1):e59-64.

157. Yang YR, Du JH, Duan XY, Kang Y, Wu LX, Meng R. [A study on HIV and syphilis infection and related risk behaviours among female sex workers working at entertainment venues in Baotou city of Inner Mongolia Autonomous Region, 2006-2009]. Chinese Journal of AIDS & STD. 2012;18(02):128-9.

158. Bai J, Zhou N, Dong X, Cheng S. [Condom use among commercial sex workers in low class establishments and related factors]. Chinese Journal of AIDS & STD. 2010;16(01):18-21.

159. Dong XY, Zhou N, Guo Y, Yu MH. [Analysis of HIV/AIDS Sentinel Surveillance among female sex workers and male clients in Tianjin City, 2007]. South China Journal of Preventive Medicine. 2009;35(2):40-1.

160. Liu Y, Zhang M, Zhang W. [STD/AIDS-related risk behaviours and condom usage among 202 female sex workers]. Chinese Journal of AIDS & STD. 2008;14(5):516-8.

161. Wang H, Gao J, Ao X. [Investigation on AIDS related knowledge, attitude and behavior among female sex workers in entertainment places in Daxing district in 2007]. Preventive Medicine Tribune. 2008;14(8):717-8.

162. Xu W, Hang J, Gao W, Zhao Y, Li W, Wang X, et al. Association between effort-reward imbalance and glycosylated hemoglobin (HbA1c) among Chinese workers: results from SHISO study. Int Arch Occup Environ Health. 2012;85(2):215-20.

163. Jia J, Gao LQ, Xing LY, Han YD, Liu M, Lei XY, et al. [Investigation on HIV-related knowledge and praxeology among commercial sex workers in Beilin District, Xi'an City]. Chinese Journal of Misdiagnostics. 2011;11(32):7940-1.
164. Jiang DK, Zhang YY, Zhou L, Zhou YJ, Zhong ML, Jin HY, et al. [The Analyses and Investigation Reports of The Surveillance Program among The Population at High Risk for AIDS]. Journal of Medical Pest Control. 2010;26(09):795-6, 9.
165. Li M, Li M, Yu JP, Min J, Zhang XJ, Han Q. [Investigation and analysis on AIDS-related knowledge and behaviors among female sex workers in Xicheng district of Beijing from 2009-2011]. Occupation and Health. 2012;28(22):2792-3, 5.
166. Xu XF, Liu SY, Zhang JF. [Analysis of AIDS Sentinel Surveillance in Huhhot in 2009]. Journal of Diseases Monitor & Control 2011;5(7):393-4+0.
167. Cao H. [Analysis of high risk AIDS-related behavior characteristics among 186 female sex workers at Xiqing district of Tianjin]. Port Health Control. 2010;15(5):27-9.
168. Feng N. [Analysis of surveillance monitoring of FSWs in Datong City in 2010]. Medical Information. 2011;24(8):5028-9.
169. Li BY, Zhao XH, Wang GR. [Recognition of AIDS among unlicensed prostitutes in Jinnan district of Tianjin]. Occupation and Health. 2012;28(4):465-6.
170. Liu ZJ, Jiang L, Wang CB, Liu TT. [Sentinel surveillance of female sex workers in Luannan County in 2011]. Journal of Capital Medical University. 2012;33(5):621-4.
171. Cui YZ, Zhu L, Yuan LL, Zhao YS, Wang J. [AIDS knowledge, behavior and HIV/syphilis infection survey among FSW in Harin from 2006 to 2009]. Chinese Journal of AIDS & STD. 2011;17(3):351-2.
172. Lai X, Zhang X, Wang X, Guo W. [Survey and Analysis of Interference effect of AIDS knowledge and related behavior to demimondaine groups in Tonghua city]. Medical and society. 2010;23(08):20-2.
173. Wang X, Gu Y, Wang L, Guan W, Gu J, Zhao L, et al. [Effectiveness evaluation of HIV prevention and intervention among female sex workers in Shenyang City]. Chinese Journal of Public Health. 2008;24(06):732-3.
174. Zhang QH. [Investigation on condom usage among female sex workers at entertainment venues in Heping District of Shenyang City, 2006] Preventive Medicine Tribune. 2007;13(6):569.
175. Zhao L, Jiang YF, Zheng J, Xu J. [Effect Evaluation on behavioral intervention among female sex workers in Anshan City]. Chinese Journal of Public Health. 2012;28(2):237-8.
176. Li Y, Du J. [HIV knowledge and sexual behaviours characteristics among female sex workers in Huanggu district, Shenyang City]. Disease Monitor and Control. 2008;2(4):195-6.
177. Liu W. [AIDS/STD serology and behavioural studies among entertainment-based female sex workers in Tonghua prefecture, China]. Contemporary Medicine. 2010;16(12):155-6.
178. Shao B, Yao SP, Wang KL, Yang JQ, Cao B, Wang J, et al. [The survey of AIDS knowledge behaviors and condom use among female sex workers in Heilongjiang Province]. Chinese Journal of Disease Control & Prevention. 2011;v.15(04):318-22.
179. Zheng J, Yin Y, Zhao WS. [Effect Evaluation on the intervention on HIV-related knowledge and behaviors among female sex workers in Anshan City] Chinese Medical Journal of Metallurgical Industry. 2012;29(2):201-2.
180. Yuan JM, Zhang GS, Xia LH, Yin GC. [Investigation on Syphilis awareness rate among populations in Nantong City of Jiangsu in 2010]. Chinese Journal of Pest Control. 2012;28(2):121-2.

181. Xian XJ. [Analysis of sentinel monitoring on HIV/AIDS in Nongan County of Jilin Province]. Blooming Season. 2011;462(22):261.
182. Zhou D, Zhou JL, Jiang FX, Wang L, Yao WQ. [An analysis of HIV/AIDS sentinel surveillance to high risk populations in Liaoning province in 2011]. Chinese Journal of AIDS & STD. 2012;18(10):648-50+56.
183. Li S, Rui B, Wang Q. [A study on HIV prevention and interventions among entertainment-venue female sex workers in Urumqi, China]. Journal of Chinese Modern Dermatology. 2004;1(1):94-6.
184. Ni M, Liu Y, Chen J, Wang D, Dong Y, Gong X, et al. [A comprehensive survey on HIV/AIDS in Kashgar prefecture of Xinjiang]. Chinese Journal of AIDS & STD. 2005;11(5):353-6.
185. Song Y, Wang Q. [A study of HIV infection status among entertainment-based female sex workers in Alar City, Xinjiang, 2004]. Endemic Diseases Bulletin. 2005;20(02):45-6.
186. Wang X, Jiang A, Xu X, Zhang Y, Li G. [AIDS risk behaviours, knowledge and healthcare seeking behaviours among female sex workers]. Journal of Medical Pest Control. 2005;21(5):345-7.
187. Zhang M, Rui BL, Xue Q, Zhai SH, Wang L. [Study on HIV and Syphilis infection among female sex workers in Urumqi of Xinjiang]. China Preventive Medicine. 2006;7(03):216-7.
188. Cai RLM, Lu WM, Chen QG, Su YM, Li YJ. [Evaluation on HIV/AIDS intervention and surveillance among high-risk populations in Gannan]. Journal of Diseases Monitor and Control. 2009;3(9):516-8.
189. Cao JB, Jiang XF, Cui ZL, Lv HY, Zhao LL, Wang RM, et al. [Analysis on HIV/AIDS sentinel surveillance sites among female sex workers in Zhengzhou City in Henan Province, 1995-2001]. Chinese Journal of AIDS & STD. 2004;10(1):61.
190. Lin B, Ceng KF, Meng W, Guo J, A Si MGL, Hu J. [Analysis of AIDS sentinel surveillance among female sex workers in Karamay from 2004 to 2008]. Chinese Journal of Public Health. 2009;25(1):75-6.
191. Liu Y, Yang X. [HIV knowledge, behavioural characteristics and condom usage among 415 female sex workers]. Modern Preventive Medicine. 2007;34(21):4144-5.
192. Li Y, Wu D, Wang SY. [Analysis of sentinel surveillance on FSWs in Xiaonan district of Hubei province in 2011]. Today Nurse. 2012;(4):132-3.
193. Ma JX, Fan XM, Su YZ, Yang Q, Zhang YM, Liu ZQ, et al. [Analysis of the changing trend of AIDS high-risk behaviors among CSWs in Baiyin City]. Health Vocational Education. 2012;30(6):98-9.
194. Song Y, Muheta, Aziguli, Yimiti, Aibibula, Zhang H, et al. [A survey of sexually transmitted diseases among entertainment-based female sex workers in Turpan City, Xinjiang, in 2006]. Endemic Diseases Bulletin. 2006;21(06):36-7.
195. Mahemuti Y, Yu FH. [A comprehensive analysis on HIV/AIDS among high risk populations in Qoqek District of Xinjiang Prefecture, 2005]. Endemic Diseases Bulletin. 2007;22(4):39-40.
196. Zeng K, Lin B, Wang F, Meng Y, Guo J. [Investigation on Risk Behavior of Sexual Transmitted Diseases/ADIS among Commercial Workers in Entertainment Places in Karamay City]. Preventive Medicine Tribune. 2008;14(02):124-6.
197. Zhang L, Li Y, Chen JJ. [Analysis on HIV knowledge and awareness among female sex workers in entertainment venues in Lanzhou City of Gansu Province]. Health Vovational Education. 2007;25(20):95-6.

198. Zheng CJ, Li F, Li XQ, Zhang ZZ, Li RL, Shi L, et al. [Investigation on knowledge, attitude and practice among female sex workers in entertainment places in Xinjiang]. Modern Preventive Medicine. 2008;35(1):96-7.
199. MAYT A. [HIV Cross-section Study on Female Sex Workers in Xinshi District, Urumqi in 2007]. Endemic Diseases Bulletin. 2008;23(4):27-8.
200. Lin B, Luo M, Wang S, Asimu G, Zeng K, Guo J, et al. [Effectiveness evaluation of AIDS related integrated interventions among female sex workers in entertainment settings in Karamay city]. Chinese Journal of AIDS & STD. 2009;v.15;No.82(01):41-3.
201. Wang M, Chen Y, Mao Z, Xue W, Liu J, Zhang K. [A study of HIV knowledge, behaviours and needs among female sex workers in Shanyang county, Sha'anxi province]. Chinese Journal of AIDS & STD. 2008;14(6):626.
202. Yang SP, Zhang ZJ, Dong YH, Zhi Q, Li RL, Wang RF, et al. [Evaluation on HIV/STD intervention for female sex workers in Hami City, Xinjiang Uyghur Autonomous Region]. Chinese Journal of AIDS & STD. 2008;14(05):513-5.
203. Liang S, Zhang S. [Effectiveness evaluation of HIV interventions among female sex workers in Huinong district, Guangxi province]. J Med Pest Control 2010;26(12):1155-6.
204. Liu S, Jiang W, Ma S, Li F. [A study of HIV-related knowledge and high-risk behaviours among community female sex workers in Huinong district, Ningxia, China]. Ningxia Med J. 2009;31(9):833-4.
205. Zhang X, Luo X. [Effect Analysis on AIDS Know ledge and Behavior Intervened among Sex Workers in Gaolan County of Gansu Province]. J Diagn Ther Derma Venereo. 2010;v.17(03):238-41.
206. Zhu MF, Liu WB. [Evaluation of the effectiveness on HIV/STD intervention among roadside female sex workers in Changji City, Xinjiang Province]. Endemic Diseases Bulletin. 2009;v.24(03):79.
207. Gao W, Li Z, Yan H, Wang D, Li Y, Dang S, et al. Preventive measures against sexually transmitted infections among female sex workers in Lanzhou, China. Scand J Infect Dis. 2012;44(5):374-80.
208. Ma L, Yang YH. [Report on Final Evaluation of Condom use Intervention to FSWs in Hutubi county in Changji Hui autonomous prefecture]. Bulletin of Disease Control and Prevention. 2011;26(6):28-30.
209. Xu YJ, Wang SP, Xue ZD, Shen JP. [HIV knowledge and sexual behaviours among female sex workers in Shanxi province, 2009]. Chinese Remedies & Clinics. 2011;11(3):304-6.
210. Chang WH, Xing AH, Wang BS, Li X, Jia H, Zhang L, et al. [Analysis of HIV /AIDS sentinel surveillance among high risk population in Shanxi in 2010]. Occupation and Health. 2012;28(4):399-402.
211. Guo H, SheN MX, Ma Q, Zhang WX, Pang RP, Li GB. [The HIV Risk Behavior Surveillance Analysis for 820 Slip Women in Longnan Municiple]. Chinese Primary Health Care. 2012;26(4):65-6.
212. Tao LD, Qi YJ, Wei HW, Chen JJ. [Epidemiology of HIV and Syphilis Among Female Sex Workers in Chenguan County, Gansu Province]. Chinese Primary Health Care. 2011;25(9):65-6.
213. Wu R, Xue SF, Yao XW. [An investigation of HIV/AIDS-related risk information among 412 female sex workers]. Chinese Journal of AIDS & STD. 2012;18(11):793, 6.

214. Zhang MN, Zhang ZH, Huang L, Wang XM. [An investigation of sexually transmitted infection and related behavioral feature on unlicensed prostitutes in Shanxi province]. Chinese Remedies & Clinics. 2011;11(9):1051-2.
215. Zhao GD, Zhong L, Li YY. [Analysis of AIDS sentinel surveillance among illicit prostitutes in Shangluo City in 2010]. Journal of Hebei United University(Health Sciences). 2011;13(4):469-70.
216. Liu DF, Chen HX. [2011 Epidemiology of HIV among Female Sex Workers in Yanchuan County, Shaanxi Province] Henan Journal of preventive Medicine. 2012;23(5):398+400.
217. Qu S, Liu W, Choi K-H, Li R, Jiang D, Zhou Y, et al. The Potential for Rapid Sexual Transmission of HIV in China: Sexually Transmitted Diseases and Condom Failure Highly Prevalent Among Female Sex Workers. AIDS Behav. 2002;6(3):267-75.
218. Zhou YJ, Liu W, Guo WG, Zhu QY, Li RJ, Zhou L, et al. [Result of behavioral surveillance of female sex workers in a coastal city in Guangxi, China.]. Journal of Applied Preventive Medicine. 2006;12(4):239-40.
219. Zhou YJ, Liu W, Liang FX, Zhu QY, Wei X, Lan GH. [Analysis of Guangxi Health IX project CSW behavioral surveillance results]. Journal of Applied Preventive Medicine. 2008;14(z1):47-9.
220. Zhu QY, Liu W, Lu WJ, Zhou YJ, Li RJ. [Analysis on the results of AIDS- related behavior surveillance in health IX project in Guangxi in 2005]. Disease Surveillance. 2007;22(11):736-9.
221. Zhongdan C, Schilling RF, Shanbo W, Caiyan C, Wang Z, Jianguo S. The 100% Condom Use Program: a demonstration in Wuhan, China. Eval Program Plann. 2008;31(1):10-21.
222. Zhou YJ, Liu W, Guo WG, Li RJ, Lu WJ, Chen L, et al. [Survey on HIV/STIs - related behaviors in female workers in recreation service]. Guangxi Journal of Preventive Medicine 2005;11(3):153-4.
223. Lu W, Liu W, Zhu Q, Li R, Liang F, Li F. [Sentinel surveillance of AIDS in Guangxi Autonomous Region, 2002-2007]. Disease Surveillance. 2009;v.24(02):103-7.
224. Wang Y, Fu X, Lin P, Wu B, Liu Y, Xu R, et al. [Survey of STD/ AIDS related knowledge and behaviors among community female sex workers]. South China Journal of Preventive Medicine. 2004;30(6):18-20.
225. Li MQ, Gan ZG, Wang YS, Wei L, Yang Y, Wang X. [Evaluation of behavioral intervention of AIDS among the commercial sex workers in the public entertainment places]. Chinese Journal of Disease Control & Prevention. 2005;9(4):306-8.
226. Li N, Sun G. [Analysis of AIDS sentinel surveillance among commercial sex workers in Henan Province]. Disease Surveillance. 2007;22(09):610-1.
227. Lin P, Sun B, Liang L, Liu H, Fu X, Yang F, et al. [Evaluation of AIDS/STDs intervention program on community female sex workers]. South China Journal of Preventive Medicine. 2004;30(6):12-4.
228. Lin P, Sun B, Liang L, Liu H, Fu X, Yang F, et al. [Influencing factors of condom usage during commercial sex acts of community female sex workers]. Chinese Journal of Disease Control & Prevention. 2006;10(5):462-5.
229. Lu L, Jia MH, Lu JY, Luo HB, Zhang XB, Ma YL, et al. [Analysis of HIV/AIDS prevalence in Yunnan province]. Chinese Journal of AIDS & STD. 2005;11(3):172-4, 64.
230. Zheng Z, Gu H, Zhang J, Wei B, Wang S. [A Survey of Awareness of AIDS and Sexual Behavior in Roadside Sex Workers]. Chinese Journal of Current Practical Medicine. 2004;3(8):13-5.

231. Cao GH, Zhi YH, He HT, Liu LH. [Survey on AIDS knowledge, attitude and behavior among four groups in Shangcai County of Henan]. Modern Preventive Medicine. 2009;36(6):1093-5.
232. Jiang M, Wu JH, Huang JG, Huang GY, Zhu BY, Sun C, et al. [STD/AIDS knowledge, attitude, behaviour and infection status among prostitutes in entertainment sites]. Theory and practice of Chinese Medicine. 2005;15(1):152-3.
233. Liu Y. [Effectiveness of intervention measure of AIDS prevention on prostitutes in Futian district in Shenzhen]. Journal of Environmental and Occupational Medicine. 2008;25(3):288-90.
234. Luo J. [Analysis of the results from HIV Surveillance of unlicensed prostitutes]. Disease Surveillance. 2005;20(8):409-12.
235. Sun C, Chen X, Zhu M, Zeng W, Shao X, Dong L. [Effect assessment on the intervention to the high-risk behavior of female sex workers in entertainment places]. Chinese primary health care 2009;23(01):30-1.
236. Sun I, Pan X, Qin L, Wang D. [A study of HIV knowledge and behavioural characteristics among county entertainment-based female commercial sex workers]. Chinese Primary Health Care. 2005;19(10):72-3.
237. Tang G, Lan S, Wu Z, Yu Z, Liu L. [Evaluation of Effect of 100% Condom Usage Promotional Project in Prevention of STD in Hecheng District]. Practical Preventive Medicine. 2007;3:920-1.
238. Wang T. Effectiveness analysis of behavioural interventions among entertainment-based female sex workers in Dazhi prefecture in 2004-2008]. Journal of Public Health and Preventive Medicine. 2009;20(109):115-6.
239. Xiong C, Li S, Chen X, Xu W. [Effectiveness evaluation of condom distribution in prevention of HIV/STD in entertainment establishment in Wuling district, Hunan province]. China Preventive Medicine. 2006;7(04):316-7.
240. Yang BF, Ye LX, Yao ZZ, Chen ZD, Xu J, Wang X, et al. [Study on HIV/AIDS knowledge among commercial female sex workers in Wuhan City, Hubei Province]. Central China Medical Journal. 2006;30(5):419-7.
241. Yang B, Xu J, Wang X, Yao Z, Liu M, Tang L, et al. [Investigation on high risk behavior and chlamydia trachomatis infection in prostitutes]. J of Pub Health and Prev Med. 2006;(03):31-3.
242. Yang BF, Xu J, Yao ZZ, Chen ZD, Wang X, Liu MQ, et al. [An analysis of the behavior characteristics and the infection of HIV and syphilis among the commercial female sex workers]. Chinese Journal of Disease Control & Prevention. 2006;2006(4):406-8.
243. Yang F, Lin P, He Q, Xu RH, Yu DN, Mo KS, et al. [A Survey on AIDS-related KABP among 5 target groups in 3 China comprehensive AIDS responses in Guangdong]. South China Journal of Preventive Medicine. 2006;32(1):10-3.
244. Yu DN, Liang QR, Luo WQ, Liu WJ. [Evaluate the impact of intervention in HIV/AIDS high-risk populations in Taishan city of Guangdong province]. Chinese Journal of AIDS & STD. 2007;13(6):34-6.
245. Zhou Y, Liu W, Dong B, Liang S, Li X, Fang X, et al. [Analysis on HIV 2related knowledge , attitude , condom using and STD prevalence among commercial sex workers in a township]. Chinese Journal of Disease Control & Prevention. 2007;11(5):488-90.
246. Ceng XL. [Effect Evaluation of Propaganda Intervention of AIDS in Female Sex Workers in Wolong District of Nangyang City,2005-2009]. Preventive Medicine Tribune. 2011;17(2):97-8+101.

247. Li W, Li Y, Mai R, Lin P, Yang L, Liu Y, et al. [Svey of STD and AIDS Knowledge and High-risk Behavior of Female Sexual Workers in Urban Area of Yangjiang City]. China Tropical Medicine. 2007;7(10):1921-2+40.
248. Li W, Lin P, Mai R, Liu Y, Yang L, Li Y, et al. [Effectiveness evaluation of HIV interventions among female sex workers in Yangjiang prefecture, China]. South China Journal of Preventive Medicine. 2008;34(02):31-2.
249. Liu.J., Yu KP, Qiu RZ. [Analysis on Effect of Comprehensive Prevention and Control for AIDS in Hengyang from 2005 to 2009]. Practical Preventive Medicine. 2011;18(4):754-6.
250. Long CW, Ng XH, Xiao SQ, Ke XZ, Chen WS, Deng J. [Analysis of AIDS knowledge and behavoral features of female sex workers in entertainment places]. China Tropical Medicine. 2008;8(4):697-700.
251. Shen C, Wang T. [STD/HIV knowledge, attitude and behavioural study among entertainment-based female sex workers in Shenzhen City, China]. Chinese Primary Health Care. 2009;23(6):87-8.
252. Xu YF, Mo XJ, Liang HH, Zhou FH, Li P, Zhou J, et al. [Investigation on STD/AIDS knowledge and risk behaviors among commercial sex women in Nanning City]. Modern Preventive Medicine. 2007;34(21):4007-8, 11.
253. Yan LM, Wang SY, Li ZH, Tang KL. [STD/AIDS knowledge, attitude, behavior and infection status among 182 prostitutes in Xiaogan urban area]. Journal of Public Health and Preventive Medicine. 2007;18(1):67-8.
254. Zhang G, Ou H, Lan L. [Analysis on AIDS knowledge, behavior and HIV infection among 512 female sex workers]. South China Journal of Preventive Medicine. 2009;35(2):22-4+7.
255. Zhang WS, Qiu XQ, Wen YM. [Survery of sexually transmitted diseases and HIV infected condition in Xiangfan city]. Journal of Public Health and Preventive Medicine. 2006;17(4):67-8.
256. Pan X, Liu Z, Chen F, Liang X. [A study of HIV and syphilis related risk behaviours among female sex workers in Baise City, 2005-2006]. Practical Preventive Medicine. 2008;No.96(05):287-9.
257. Pan X. [The investigation of Venereal disease for 864 female sex workers]. Youjiang Medical Journal. 2009;37(01):10-3.
258. Wang C. [Impacts of 100%-condom-usage promotion on HIV knowledge and risk behaviours among female sex workers]. Henan Journal of Preventive Medicine. 2010;21(1):28-9.
259. Wang Z, Zeng X, He Q, Ji J, Lin X, Han X. [Assessment of HIV/AIDS control and reproductive health service among female sex workers in the community]. China Tropical Medicine. 2010;v.10(10):1202-3.
260. Xu X, Yang Y, Xu H. [Correlation of AIDS and Prostitutes in High-Class Entertainment Centers]. Journal of Tropical Medicine. 2007;7(6):610-1.
261. Chen Y, Liu W, Ted H, Meng D, Xiang S. [Survey of sex workers along Guangxi border region about their HIV related knowledge, behavior and infections]. Chinese Journal of Disease Control & Prevention. 2010;14(7):619-22.
262. Chen ZJ, Xia WZ. [Investigation on HIV infection among 879 commercial female sex workers in Nanning City in Guangxi Province]. Journal of Guangxi Traditional Chinese Medical University. 2008;11(1):30-1.

263. He Y, Xu Y, Liang F, Zhou F, Mo X. [Results of intervention of AIDS health education and behavior in sex workers in entertainment places in Nanning City]. China Tropical Medicine. 2009;9(02):333-4.
264. Jiang M, Wu JH, Huang JQ, Huang GY, Zhu BY, Sun C, et al. [Survey of STI/AIDS-related knowledge, attitude and behaviors and infection rates of sex workers in entertainment places in Jingzhou City]. Chinese Journal of Disease Control & Prevention 2012;16(2):175-7.
265. Li Y, Lin P, Detels R, Fu X, Deng Z, Liu Y, et al. [Prevalence of HIV infection and sexually transmitted disease and associated risk factors among female sex workers in Guangdong province]. Disease Surveillance. 2009;24(08):599-602.
266. Liu C, Xu J, Zhou W, Yang DL, Yao ZZ, Wang X, et al. [Baseline investigation on three kinds of high risk population in Wuhan, China Bill & Melinda Gates Foundation AIDS program]. Journal of Public Health and Preventive Medicine. 2011;22(2):15-8.
267. Lu WJ, Liu W, Zhu QY, Lan GH, Li F. [Analysis of the results of comprehensive surveillance in AIDS-related high risk groups in 20 cities/counties of Guangxi in 2007]. Chinese Journal of AIDS & STD. 2008;14(6):583-6.
268. Nong C, Huang H, Meng D, Xiang C, Zhao X. [Situational study of 406 female sex workers in Ningming county, Guangxi province, in 2007]. Applied Prev Med. 2008;14(S1):76-7.
269. Qiu J, Wang Z, Li L, Liu Y. [Survey on AIDS Related Know ledge and Behavior Among Prostitutes in Shenzhen]. Journal of Preventive Medicine Information. 2008;24(11):876-8.
270. Liu Y, Huang C, Li L, Fang L, Shi X. [Effective of Intervention Measure of AIDS Prevention on Prostitutes in Futian District in Shenzhen During 2004 Through 2007]. J Environ Occup Med. 2008:288-90.
271. Tan S, Lan R, Wei J, Xie S, Qin Q, Lin F. [A study of STD/HIV infection status among female sex workers in Laibin City, 2007]. Applied Prev Med. 2010;16(02):98-9.
272. Tan W, Zhou H, Liu H, Mo X, Luan W. [Comprehensive HIV surveillance results analysis for commercial sex workers in Nanning City, 2007]. Guangxi Medical Journal. 2008;30(11):1727-8.
273. Tan WW, Liu HY, Liu FX, Huang CH, Zhou HF. [Sentinel surveillance of syphilis in female sex workers in Nanning city, 2007-2009]. Disease Surveillance. 2011;26(2):106-9.
274. Tan S. [Current status of health education and behavioral interventions on AIDS unlicensed prostitute]. Chinese Journal of Dermatovenereology. 2009;14(12):1115-6.
275. Wang Q, Yang P, Gong X, Jiang J, Yang B, Yang L, et al. [Syphilis prevalence and high risk behaviors among female sex workers in different settings]. Chinese Journal of AIDS & STD. 2009;15(04):398-401.
276. Wen X. [Survey on KAB and serology of AIDS among 360 female commercial sex workers in Guilin]. Modern Preventive Medicine. 2009;36(14):2687-9.
277. Wen XQ. [Effects of HIV-related health education and intervention to FSWs in Guilin City from 2007 - 2011]. Journal of Public Health and Preventive Medicine. 2012;23(6):125-7.
278. Xu Y, Mo X, Li S, He Y, Huang C. [Survey of high risk behaviors related with HIV/AIDS and infectious status in female sexual workers in Nanning City]. China Tropical Medicine. 2008;8(10):1809-10.
279. Xu YF, Zhou FH, Mo SJ, Li SS, He Y, Huang CH. [Surveillance of Commercial Sex Workers in Nanning, 200 -2008]. Journal of Preventive Medicine Information. 2009;25(8):615-7.

280. Wu ZZ, Zhu JP, Fang PQ, Ding J. [An investigation of HIV/AIDS knowledge, attitude and behaviour among commercial female sex workers in Hubei Province after the implementation of 100% condom use intervention program]. Chinese Journal of AIDS & STD. 2009;15(3):308-9.
281. Wu ZZ, Zhu JP, Fang PQ, Ding J. [An investigation of HIV/AIDS knowledge, attitude and behaviour among commercial female sex workers in Hubei Province after the implementation of 100% condom use intervention program]. Chinese Journal of AIDS and STD. 2009;15(3):308-9.
282. Zhang SX, Zhi YH. [Analysis on AIDS related knowledge, attitude and behavior among the different crowds in Shangcai Country of Henan Province]. China Medical Herald. 2008;5(36):108-10.
283. Zhang YX, Lin HT, Feng WD, Shan GS, Zhang TJ. [Syphilis and HIV infection status among commercial sexual workers in Liuzhou, Guangxi]. Journal of Tropical Medicine. 2011;11(3):337-9+55.
284. Zheng J, Tan BY, Liu J, Wu YP, Yu KP, Zhao JS, et al. [Study on the Estimated Number, AIDS Knowledge Level and Risk Behavior of Female Sex Workers and Drug Users in 3 Counties of Hengyang] Practical Preventive Medicine. 2011;18(2):211-3.
285. Zhou J, Huang Z, Deng B, Chen Y, Luo R. [Survey on the behavioral characteristics and sexually transmitted infection among 418 female commercial sex workers]. Modern Preventive Medicine. 2010;37(6):1158-9, 61.
286. Zhou YJ. [Analysis of risk behaviors of 105 female sexual workers positive for Treponema pallidum]. China Tropical Medicine. 2008;8(10):1832, 47.
287. Ba iY. [Investigation on AIDS related knowledge, behaviors and infection among commercial female sex workers in Liuzhou city in 2008]. Preventive Medicine Tribune. 2009;15(12):1224-6.
288. Bai Y, Weng YQ, Feng WD. [Sentinel Surveillance of AIDS in Liuzhou, 2008]. Journal of Preventive Medicine Information. 2010;26(7):527-30.
289. Liao J, Zhou HY, Rong DF, Li ZX, Wang J, Wang L. [HIV/AIDS related knowledge, attitude and behavior among dif ferent crowds in Gucheng county of Hubei province]. Chinese Rural Health Service Administration,. 2010;30(9):766-8.
290. Lin M, Jian H, Liang F, Meng A, Ban L, Huang Y. [STD/AIDS Screening Results and Related Knowledge and Behavior of Female Sex Workers in Western Guangxi]. Journal of Nursing Science. 2008;23(17):27-9.
291. Mao A, Shi X, Xiao L, Zhang J, Peng J. [Effectiveness evaluation of HIV knowledge and behavioural interventions among lower-end female sex workers in Jingzhou City, China]. J of Pub Health and Prev Med. 2010;v.21(02):128-9.
292. Tan J, Chen L, Cai W, Yang Z, Shi X, Wang X. [Study on AIDS/STD related risk behaviors among female sex workers in Shenzhen]. Chinese journal of Social Medicine. 2009;v.26(04):242-4.
293. Wang WM, Chen ZZ, Li P. [Survey on AIDA related knowledge and behavior among female sex workers in entertainment setting in a global fund AIDS program county in Henan province]. Henan Journal of Preventive Medicine. 2010;21(1):36-7.
294. Weng YQ, Bai Y, Feng WD. [High risk behavior toward HIV/AIDS of female sexual service workers with different marital status in Liuzhou City]. China Tropical Medicine. 2011;11(2):170-1.
295. Wu D, Hu J, Zhu X, Zhang Z, Shan J, Tao X, et al. [Descriptive research of knowledge , attitude and practice related to AIDS among female sex workers]. Chinese Journal of Disease Control & Prevention. 2009;13(4):398-401.

296. Yu DY, Weng YQ. [Analysis on high-risk groups of AIDS comprehensive intervention effect participated by family planning department in Liuzhou city]. Chinese Journal of Pest Control. 2012;28(7):765-8.
297. Zhang L, Xue FH, Zhang XQ, Dong SB. [Study on HIV/AIDS related knowledge and behavior among female sex workers in Xincai County of Henan Province]. Henan Journal of Preventive Medicine. 2010;21(3):235-7.
298. Zhao Y, Li M, Li Y, Liang Z, Zhang X, Guo Z. [The status of sexually transmitted infections in the female sex workers at the entertainment venues in Panyu district. International Medicine & Health Guidance News. 2010;16(18):2212-4.
299. Chen L, Tan J, Shi X, Gan Y, Zhang Y, Zhao J, et al. [The comprehensive surveillance of AIDS among unlicensed female sex workers in Shenzhen city]. Journal of Tropical Medicine. 2010;10(6):748-9.
300. Dun ZJ, Ling L, Xia HY, Wang C, Lin AH, Lu CY. [Survey on HIV/AIDS related KABP of four high-risk population in Guangzhou City of Guangdong Province]. Chinese Journal of Health Education. 2011;v.27(11):843-6.
301. Hu S, Huang J, Li C, Huang J, Xuan R. [Survey on AIDS related knowledge and behaviors among female sex workers in entertainment places in Qingcheng district of Qingyuan City]. Occupation and Health. 2010;26(19):2218-30.
302. Huang ZX, Long HY, Huang YC, Wei YM. [Survey of HIV knowledge and infection in sexual workers in Fangchenggang City]. China Tropical Medicine 2011;11(6):778.
303. Liao S, Weeks MR, Wang Y, Nie L, Li F, Zhou Y, et al. Inclusion of the female condom in a male condom-only intervention in the sex industry in China: a cross-sectional analysis of pre- and post-intervention surveys in three study sites. Public health. 2011;125(5):283-92.
304. Lin RL, Quan XB, Ye RG, Huang ZN, Ning JR. [Investigation on core indicator of behavioral surveillance among female sex workers in Yulin, 2009-2011]. China Tropical Medicine. 2012;12(6):700-2, 24.
305. Leng B, Zhang L, Zi XM, Xu K. [Study on the HIV-related knowledge and behaviours of illicit prostitutes in Zhoukou City]. Henan Journal of preventive Medicine. 2011;22(5):367-8.
306. Luo JH, Jin WD, Li GB, Wu P. [Analysis of surveillance monitoring of illicit prostitutes in Shaodong County, Shaoyang County of Hunan Province in 2009] Practical Preventive Medicine. 2011;18(1):166-7.
307. Nie ZQ, Lin P, Li Y, Wang Y. [Surveillance of AIDS high-risk people in Guangdong province,2009]. Journal of Tropical Medicine. 2011;11(01):29-31+45.
308. Wang J, Wang T, Cen Y, Lai X, Li L, Chen C, et al. [Prevalence of sex transmitted disease or its related symptoms and associated risk factors among female sex workers in Zhongshan]. Journal of Tropical Medicine. 2010;10(4):477-80.
309. Wen X. [An analysis on condom using behavior and influencing factors among female sex workers(FSW) in Guilin city]. Health World. 2010;4(9):2.
310. Xiang H. [Analysis of the HIV-related praxiology among FSWs from entertainment venues in Laifeng County of Hubei Province at 2009]. Journal of Mathematical Medicine. 2011;24(2):199-201.
311. Xiang Z, Yin YP, Shi MQ, Jiang N, Han Y, Wang HC, et al. Risk factors for Mycoplasma genitalium infection among female sex workers: a cross-sectional study in two cities in southwest China. BMC public health. 2012;12:414.

312. Xu HF, Song T, Xie YK, Gao DL, Lv JY, Liu HY. Effectiveness and analysis of interventions for HIV/AIDS prevention among female sex workers in entertainment establishments in Shangqiu]. Henan Journal of preventive Medicine. 2011;22(2):95-6.
313. Ceng XL, Li P, Ma CH. [The effects of sexually transmitted infections AIDS behavioral intervention among female sex workers in project region]. Henan Journal of preventive Medicine. 2011;22(2):92-4.
314. Zhang L. [Investigation on HIV-related Knowledge and Behaviors among Illicit Prostitutes in Xinyang City in 2009]. Henan Journal of preventive Medicine. 2011;22(2):109-10.
315. Zhao JZ, Ren SH, Wan Y, Xu H, Zhou T, Sun Y. [Research on AIDS/Infectious Diseases of Genital Tract and Related Behavior of Female Sex Workers in Low-grade Areas]. Chinese Journal of Social Medicine. 2011;28(5):326-8.
316. Zhong J, Lin J, Hu YM, Tan LL, Wang G. [HIV/STD infection and risk behaviors among commercial sex workers at various places in Wuzhou City, Guangxi Zhuang Autonomous Region]. Chinese Journal of Health Education. 2011;27(3):177-80.
317. Zhong J, Lin J, Hu Y, Tan L, Wang G. [HIV/STD infection and risk behaviors among commercial sex workers at various places in Wuzhou City, Guangxi Zhuang Autonomous Rgion]. Chinese Journal of Health Education. 2011;v.27(03):177-80.
318. Zhou JL, Duan KM. [Comprehensive analysis on HIV/AIDS epidemic in Beihu District in Hunan Province, 2009]. Practical Preventive Medicine. 2010;17(2):392-3, 0.
319. Zhou X, Ling L, Xu H, Lu C, Wu J, Deng X, et al. [Knowledge and behavior about AIDS/STD among female sex workers]. Chinese Journal of Public Health. 2010;26(8):954-6.
320. Bai Y, Zhang JP, Ouyang Y. [Analysis on monitoring results of AIDS among female sexual workers in Liuzhou City in 2010]. Chinese Journal of Pest Control. 2012;28(9):964-6.
321. Chen CF, Zhao Y, Xiao HT, Tong XY. [Analysis of HIV/AIDS sentinel surveillance among commercial sex workers in Dangyang City in 2010]. Journal of Public Health and Preventive Medicine. 2011;22(3):73-4.
322. Chen ZB, Pan XM, Chen ZT, Deng YL. [A study of HIV/AIDS-related knowledge and behaviours on female sex workers at different venues in Lianzhou city of Guangdong province]. South China Journal of Preventive Medicine. 2011;37(2):34-6.
323. Gui Q, Zeng LG, Wang LY, Liu SJ, Kang XZ, Tu LL. [Analysis of sentinel surveillance of FSWs in Wuling District of Changde City in 2010]. Chinese Journal of AIDS & STD. 2012;18(10):701-2.
324. He B, Xuan DQ, Zeng QM, Lin YS, Wu CD, Li RY, et al. [Investigation on Characteristics of Sexual Behaviours and Acceptance towards Female Condom among Female Sex Workers]. Chinese Journal of Public Health. 2012;28(10):1383-4.
325. Huang KZ, Chen XH, Li WJ, Chen ZF. [Survey of STD and AIDS knowledge and high risk behavior of female sexual workers in Yangjiang City]. South China Journal of Preventive Medicine. 2010;36(04):41-2.
326. Jiang N. [Surveillance of risk behaviors facilitating among commercial sex works and analysis of HIV, Syphilis, HCV and HBV infection]. Journal of Medical Forum. 2012;33(4):81-2.
327. Nong LP, Nie L, He B, Bi SZ, Li B. [Condom use among female sex workers in Pingxiang city of Guangxi Zhuang Autonomous Region]. Chinese Journal of AIDS & STD. 2011;17(4):444-7.

328. Zhu L, Qiu XS, Xie AQ, Gong WS, Wen MX, Yang XZ. [Analysis of AIDS Sentinel Surveillance among prostitutes in Xiangyang in 2010]. Journal of Public Health and Preventive Medicine. 2011;22(3):92-3.
329. Ke XZ, Xiong F, Xie RQ, Peng YH, Song W. [Analysis of sentinel monitoring of HIV, syphilis and HCV in Huangshi City in 2011]. Journal of Public Health and Preventive Medicine. 2012;23(5):80-1.
330. Liang YJ, Yu ZW, Ma JQ. [Comparative study on the HIV/AIDS awareness among local residents and high-risk population in Shunde District of Foshan City]. China Health Industry. 2012;9(2):129.
331. Quan XB, Lin RL, Huang ZN, Ning JR. [Study on HIV /TP /HCV infection and condom use of prostitute in Yulin city]. Chinese Journal of Health Laboratory Technology. 2012;22(7):1687-9.
332. Tang J, Zhang ZK, Zhou Y, Wen XQ, Zhou HJ. [Analysis of sentinel monitoring of AIDS among high risk population in Guilin,2011]. Chinese Journal of AIDS & STD. 2012;18(8):533-6.
333. Tang MJ, Zhong FH, Liu JJ, Zhang DL, Tan XZ. [Analysis of AIDS Sentinel Surveillance in Yulin city in 2011]. Journal of Applied Preventive Medicine. 2012;18(1):40-2.
334. Wei XQ, Liang ZQ, Wei KB, Liang XW. [Analysis on the condom use of commercial sex workers in cheap entertainment places in Hechi city in 2011]. Journal of Applied Preventive Medicine. 2012;18(4):233-5.
335. Guo J, Lu YC, Sun LK, Xie JH, Wen Y, Feng L. [Evaluation of behavioral intervention among female sex workers in Guandu District of Kunming City]. Soft Science of Health. 2006;20(3):224-7.
336. Wang Z, Liu W, Chen H, Zhou X, Miao D, Tao X, et al. [HIV sentinel surveillance among community female sex workers in Dazhou prefecture in 2002]. Journal of Preventive Medicine Information. 2005;2:192-3.
337. Yang HW, Zhu XY, Sun HY, Yang XL, Feng Y, Tang G, et al. [Investigation on sexual behaviour, health-seeking behaviour and gynecological examination among female sex workers in a city]. Journal of Preventive Medicine Information. 2003;19(z1):42-3.
338. Fu YC, Guo QA, Xiong YC, Xu FC, Dong XQ. [Evaluation of comprehensive HIV/AIDS intervention program at entertainment venues in Jinping County]. Soft Science of Health. 2006;20(3):249-50.
339. Huang LH, Liu YZ, Chen ZJ, Lu MJ, Xu XR, Zhang XZ, et al. [Comprehensive HIV/AIDS intervention for female sex workers - effectively curb the spread of STD and AIDS]. Soft Science of Health. 2006;20(3):271-3.
340. Huang ZM, Yang LG, Lan F, Zhang YP, Fang HY, Zhang XQ, et al. [An analysis of the effectiveness on HIV/STD comprehensive intervention for female sex workers in Luxi County, Yunnan Province]. Soft Science of Health. 2006;20(3):254-7.
341. Kan HY, Du CH, Long P, He R, Wen RB, Li SY. [Analysis on HIV/AIDS comprehensive intervention among female sex workers in entertainment venues in Yuanjiang County, Yunnan Province]. Soft Science of Health. 2006;20(3):262-3.
342. Jin Y, Yin GG, Bao WS, Liu CB, Mao BB, Deng GY, et al. [Study on HIV/AIDS behavior intervention in the waitresses of inns around roads]. Soft Science of Health. 2006;20(1):55-8.

343. Kang JX, Zhou JS, Zhang LL, Zhou DL, Lai WH, Liu L, et al. [Evaluation of AIDS intervention program on unlicensed prostitute by using continual behavior surveillance survey data]. Modern Preventive Medicine. 2008;35(6):1001-3.
344. Lai W, Zhou D, Zhang L, Zeng Y, Huang T. [Risk behaviors of AIDS among female CSW and also IDU]. Parasitoses and Infectious Diseases. 2009;7(2):80-3.
345. Lau JT, Wang R, Chen H, Gu J, Zhang J, Cheng F, et al. Evaluation of the overall program effectiveness of HIV-related intervention programs in a community in Sichuan, China. Sex Transm Dis. 2007;34(9):653-62.
346. Lau JT, Choi KC, Tsui HY, Zhang L, Zhang J, Lan Y, et al. Changes in HIV-related behaviours over time and associations with rates of HIV-related services coverage among female sex workers in Sichuan, China. Sex Transm Infect. 2011;84(3):212-6.
347. Li J, He S, Xie G, Li Y, Li L, Du Z, et al. [Effectiveness evaluation of comprehensive HIV interventions among female sex workers in Ershan county, China]. Soft Science of Health. 2006;20(03):251-3.
348. Li S. [A Survey on 2003 Behavior Monitoring of 364 CSW in Bazhong City]. JPrev Medical Information. 2004:494-5.
349. Li ZF, Hong WX, Long GQ, Li XQ, Tan LF, Bu LQ. [Effectiveness analysis of comprehensive HIV interventions among sex workers and drug users in Gengma county, Yunnan province]. Soft Science of Health. 2006;20(3):212-4.
350. Luo G, Feng Z, Wang J. [HIV behavioural surveillance among female sex workers in Meishan prefecture, China, in 2003-2005]. Journal of Preventive Medicine Information. 2006;22(4):438-41.
351. Peng L, Luo J, Chen L, Yu Z, Mao Y, Chang W, et al. [Evaluation on AIDS Intervention to Unlicensed Prostitute in Hongta District of Yuxi from 2003 to 2004]. Journal of Preventive Medicine Information. 2007;13(4):291-3, 5.
352. Shi X, Jin Y, Bao W, Yang Z, Liu L, Mao B, et al. [Effectiveness analysis of comprehensive HIV interventions among female sex workers in Qujing prefecture, China]. Soft Science of Health. 2006;20(3):235-7.
353. Wang GX, Ma JG, Zhang MR, Zeng JH, Li N, Chang D, et al. [Effect assessment on the implementation of China-UK HIV/AIDS Prevention and Care Project in Kaiyuan]. Soft Science of Health. 2006;20(03):219-21+34.
354. Wang L. [Analysis of female sex workers behavioural surveillance data in Leshan prefecture in 2003]. Journal of Preventive Medicine Information. 2004;20(3):297-9.
355. Zhang YL, Li K, Ma Y, Li JL, Zhang C, Li FR, et al. [The Evaluation of the Condom Social Marketing Project amomg female sex workers - comprehensive STD/AIDS Intervention Project on high risk populations in Hongta District, Yunnan Province]. Soft Science of Health. 2006;20(3):228-31.
356. Zhao Q, Tang JB, Zhou HH, Wang P. [A survey on HIV/AIDS behaviour and surveillance among prostitutes in Dazhou City, Sichuan Province]. Journal of Preventive Medicine Information. 2004;20(5):561-3.
357. Chen XY, Peng ZZ, Li XC, Ding YP, Shu XL, Gong LH, et al. [Evaluation of HIV/AIDS prevention intervention for female sex workers in Wenshan District of Yunnan Province]. Soft Science of Health. 2006;20(3):265-7.
358. Duan Y, He WZ, Zhang CP, He CH. [Evaluation on comprehensive HIV/STD prevention intervention for urban female sex workers. Soft Science of Health. 2006;20(3):268-70.
359. He LS, Li QY, Xu CJ. [Intervention of high risk behavior among prostitutes in the recreational places]. Journal of Public Health and Preventive Medicine. 2007;18(1):68-9.

360. Jing X, Ou K, Zhu Z. [Effect evaluation of AIDS health education for female sex workers in the public entertainment places]. Chinese Internal of Health Education. 2006;22(11):858-60.
361. Lau JT, Zhang J, Zhang L, Wang N, Cheng F, Zhang Y, et al. Comparing prevalence of condom use among 15,379 female sex workers injecting or not injecting drugs in China. Sex Transm Dis. 2007;34(11):908-16.
362. Lei Z, Du M, Wang Z, Zhong X. [Analysis of serological and behavioral survey results for HIV and TP in 259 female sex workers (FSW) from communities in 2004] Disease Surveillance. 2005;20(09):30-2.
363. Luo XR. [Current HIV/AIDS related knowledge and behaviors condition among female commercial sexual workers in Yibin]. Morden Preventive Medicine. 2009;36(14):2690-2.
364. Mao YG, Gong YH, Zhang JH, Han YH, Mao MX, Luan RS. [An Analysis Study on Integrate Surveillance of HIV/AIDS Infection From 2003 To 2004 in Liangshan District]. Modern Preventive Medicine. 2005;32(06):637-8+43.
365. Su DT, Zhang YR, Liang J, Ouyang Y, Mu SH, Luan RS. [Survey of female sexual workers in top grade entertainment place of Chengdu donwtown district]. Modern Preventive Medicine. 2005;32(1):63-5.
366. Tan Y, Xie W, Huang J. [Effectiveness analysis of behavioural interventions among entertainment-based female sex workers in Menghai county, Yunnan]. Soft Science of Health. 2006;20(3):276-8.
367. Wang A, Chen F, Peng C, Chen D, Xiao K, Luo L. [A study of HIV infection status and related risk behaviours among female sex workers in Zhijin county, Guizhou province]. Chinese Journal of AIDS & STD. 2006;12(5):414.
368. Wang Q, Yang P, Wan S, Zhong M, Wang G, Zhang L, et al. [Mass treatment service integrated with comprehensive intervention for female sex workers in Sichuan Province]. Chinese Journal of AIDS & STD. 2005;11(6):438-41.
369. Wen Y, Zhang Q, Ren XQ, Fu YF, Yang B, Fang RP, et al. [A study of the role of maternal and child health organisations in HIV/STD prevention among female sex workers]. Soft Science of Health. 2006;20(03):287-90.
370. Xu SM, Qian TX, Peng CL, Yang MF, Lu RR. [Surveillance of HIV and syphilis in Chongqing in 2004]. Modern Preventive Medicine. 2006;33(05):833-4.
371. Zou YD, He CY, Yang JH. [Analysis on HIV/AIDS behavioural intervention project for female sex workers in Chuxiong City, Yunnan Province]. Soft Science of Health. 2006;20(3):245-8.
372. Cao X, Jiang Z, Ruan Y, Liang S, Song B, Hu W, et al. [Investigation on initiation into commercial sex among female sex workers in Xichang city]. Chinese Journal of Public Health. 2006;22(5):516-7.
373. Cao XT, Ruan YH, Jiang ZQ, Liang S, Qin GM, Chen KL, et al. [Study on sexually transmitted diseases and related risk factors among female sex workers who have regular partners]. Chinese Journal of Preventive Medicine. 2006;40(2):144.
374. Chen CL, Li XS, Zhou GH, Li QF, Zhang Q, Chen JL, et al. [Evaluation of intervention amongst roadside female sex workers in Malong County of Yunnan Province]. Soft Science of Health. 2006;20(3):238-41.
375. Chen X, Cao X, Ruan Y, Zhang W, Duan Y, Jiang Z, et al. [Factors Associated with Drug Use among Female Sex Workers in Xichang of Sichuan Province]. Chinese Journal of Drug Abuse Prevention and Treatment. 2006;12(1):1-4.

376. Hu WH, Li J, Liu HM. [Condom usage among female sex workers at star-rated hotels in Furong District of Changsha City]. Practical Preventive Medicine. 2008;15(5):1667-70.
377. Jiang Z, Cao X, Ruan Y, Hao Q, Song B, Hu W, et al. [Factors associated with unprotected sex behavior among female sex workers in drug heavy using area]. Chinese Journal of Disease Control & Prevention. 2006;10(05):458-61.
378. Li L, Li ZM, Zhao SP, Liu J, Pu ZL. [An evaluation and assessment report of baseline investigation on HIV/AIDS high risk population intervention in Kunming, 2005]. Journal of Dermatology and Venereology. 2007;29(1):1-3.
379. Li Z, Feng X. [Knowledge and Behavior About AIDS of Female Sex Workers in Entertainment Places in Qinyang District, Chengdu]. Journal of Preventive Medicine Information. 2009;25(5):423-5.
380. Li Z. [A study of comprehensive HIV interventions among female sex workers in Moso region, Yunnan province, China]. Soft Science of Health. 2006;20(3):279-80.
381. Luo G, Feng Z, Wang J. [Analysis of high-risk behaviors among CSWs in urban area of Meishan]. Journal of North Sichuan Medical College. 2006;21(3):223-6.
382. Ruan Y, Cao X, Qian HZ, Zhang L, Qin G, Jiang Z, et al. Syphilis among female sex workers in southwestern China: potential for HIV transmission. Sex Transm Dis. 2006;33(12):719-23.
383. Tian L, Ma Z, Ruan Y, Cao X, Huang J, Wang D, et al. [Incidence rates of human immunodeficiency virus and syphilis as well as the rate of retention in a 6-month follow-up study of female sex workers in areas with heavy drug use in Xichang of Sichuan province, China]. Chin J Epidemiol. 2006;27(11):939-42.
384. Wang C, Yang H, Yang Y, Dong C, Zhang Y, He Z, et al. [Evaluation of promote the use of condoms 100% in entertainment places of Simao]. Chinese Medical Science & Health. 2007;(7):1-4.
385. Wang J, Tao R, Hu H, Shi Q, Shan X. [Effectiveness analysis of comprehensive STD/AIDS interventions among female sex workers in Huaning county, Yunnan]. Soft Science of Health. 2006;20(3):293-5.
386. Wang Y, Zhang GG, Yang HW, Sun HY, Feng Y, Tang G, et al. [Analysis of behaviors surveillance of 1175 female sexual workers in Mianyang City]. Modern Preventive Medicine. 2007;34(24):4611-5.
387. Wang Y, He H, Chen C. [Behavioural surveillance report of female sex workers in Shangri-La, Yunnan]. World Health Digest Medical Periodical. 2009;6(10):247-8.
388. Yuan MJ, Bai W, Dong H, Cheng CQ. [Report on HIV/AIDS surveillance among female sex workers in Deyang City of Sichuan Province in 2005]. China Journal of Clinical Medicine Hygiene. 2006;4(9):96-8.
389. Yuzhen LC, Ya X, Ma YG, Gama ZM, Duoji WM, Suolang DJ, et al. [Investigation on HIV infection and related behaviour among female working at entertainment venues in Lhasa City of Tibet Autonomous Region]. Chinese Journal of AIDS & STD. 2006;12(6):545, 2.
390. Zeng K. [Behavioral Investigation of AIDS on Unlicensed Prostitute in Aba Canton of Sichuan Province from 2002 to 2006]. Journal of Preventive Medicine Information. 2007;23(6):720-1.
391. Du J, Wang G, Wang W, Gu J, Chang D, Zhao M, et al. [Analysis on the survey of HIV/AIDS/STI among the commercial sex workers in Kaiyuan city]. Soft Science of Health. 2008;22(1):84-6.
392. Gu J, Lau JT, Chen H, Tsui H, Ling W. Prevalence and factors related to syringe sharing behaviours among female injecting drug users who are also sex workers in China. Int J Drug Policy. 2011;22(1):26-33.

393. Huang Y, Zhou XW, Fan L, Guo ZH, Qiu XM. [A survey on HIV/AIDS knowledge and sexual behaviour among commercial female sex workers in Leshan City, Sichuan Province]. Journal of Preventive Medicine Information. 2006;22(1):73-4.
394. Ji J. [Results of monitoring of AIDS behavior of female sexual workers in Changjiang County]. China Tropical Medicine. 2007;7(10):1853-4.
395. Lau JT, Gu J, Tsui HY, Chen H, Holroyd E, Wang R, et al. Prevalence and associated factors of condom use during commercial sex by female sex workers who were or were not injecting drug users in China. Sexual health. 2012;9(4):368-76.
396. Lei JH, Xiao YL, Sun JY. [Analysis of the changing trend of AIDS high-risk behaviors among CSWs in Kaili city]. Chinese Journal of AIDS & STD. 2012;18(2):124-6.
397. Li D, Yuan F, Hu S, Lv F. [High risk behaviors and HIV/STI prevalence among female sex workers in different settings]. Chinese Journal of AIDS & STD. 2007;13(03):210-3.
398. Li Q, Xu J, Wang W, Wang G, Chang D, Wang N. [Survey of high risk behaviors and HIV/STD infection among FSWs from different venues of Kaiyuan city]. Chinese Journal of AIDS & STD. 2009;15(02):164-6.
399. Li Q, Xu J, Wang H, Wang G, Zhang W, Wang N. [Prospective epidemiological study of factors correlated with seeking HIV post testing counseling service among female sex workers in Kaiyuan city]. Chinese Journal of AIDS & STD. 2010;16(03):295-7.
400. Li Y, Wen Y, Hu Z, Chen W, He L, Yang F. [Integrated AIDS Behavioral Intervention Among Female Sex Workers in Gejiu City]. Journal of Kunming Medical University. 2009;v.30(09):113-7+21.
401. Shi X. [Analyzing behavior surveillance of female sex workers in Shehong county from 2006 to 2007]. The Chinese Health Service Management. 2008;241(07):492-4.
402. Sun JY, Xiao YL, Huang GX, Lei JH. [Monitoring on AIDS behavior among prostitutes in Kaili from 2006 to 2010]. Modern Preventive Medicine. 2012;39(14):3593-8+600.
403. Tan Y, Feng L. [STDs /AIDS Sentinel Surveillance of FSW in Ganzi Prefecture, 2006 and 2010]. Journal of Preventive Medicine Information. 2011;27(9):670-3.
404. Wang G, Chang D, Bi A, Du X, Li N. [Exploring intervention model for female sex workers with low payment in one city]. Soft Science of Health. 2009;23(1):107-9.
405. Sun X, Wang N, Li D, Zheng X, Qu S, Wang L, et al. The development of HIV/AIDS surveillance in China. AIDS. 2007;21 Suppl 8:S33-8.
406. Wu Q, He CY, Duo L, He LM, Chen Y, Wang YY. [HIV/AIDS-related Risk Behaviour in Female Sex Workers, Southwest China]. Science & Technology Information. 2011;(13):9-10.
407. Zheng WB, Huang DS, Li YP, Guo JH, Yang JF, Liu L, et al. [Evaluation on intervention effect of HIV /AIDS relevant knowledge and behaviors among female commercial sex workers]. Chinese Journal of Pest Control. 2012;28(6):600-2+7.
408. Zheng Y, She X, Long Q, Hua I. [Evaluation of AIDS Intervention Among Female Sex Workers in Jiulongpo District of Chongqing 2006-2009]. Journal of Preventive Medicine Information. 2010;26(10):788-91.
409. Zhi Q, He J. [Effect evaluation of consecutive behavior intervention among female sex workers for preventing HIV/AIDS]. Journal of Third Military Medical University. 2011;33(6):615-7.
410. Liu ZY, Gong YH. [Evaluating intervention effects on HIV/AIDS high-risk behavior in Qilin]. Soft Science of Health. 2010;24(02):133-6.
411. Gao L, Che Z, Lu Y. [A cross-sectional study on STDs/AIDS and hiv of 270 female sex workers]. J Dematology and Venereology. 2008;30(03):39-42.

412. Guo L, Wang YL, Li CM. [Investigation on HIV-related knowledge and praxiology among illicit prostitutes in Guizhou Province]. Chinese Journal of Public Health. 2011;27(7):891-2.
413. Han D, Fang Q, Jiang W, Li Z, Zhang J. [Evaluation of AIDS intervention among female sex workers in entertainment places in districts with different fund support in Chengdu]. Journal of Preventive Medicine Information. 2010;26(6):425-9.
414. Chen Y, Li X, Zhou Y, Zhang C, Wen X, Guo W. Alcohol consumption in relation to work environment and key sociodemographic characteristics among female sex workers in China. Subst Use Misuse. 2012;47(10):1086-99.
415. He J, Yi R, Long Q, Li N, Yu X, Zheng Y. [Evaluation of the effect of AIDS outreach educational intervention based on sexually transmitted disease clinics for female sex workers]. Modern Preventive Medicine. 2010;v.37(15):2888-90.
416. He QX, Xu QZ, Wang YB, Ma GS. [Analysis of the effect of Comprehensive Analysis on illicit prostitutes in Luliang County, Yuinan]. Soft Science of Health. 2011;25(2):115-8.
417. He QX, Xu ZQ, Wang YB, Ma GS. [Evaluation on comprehensive HIV/AIDS intervention for female sex workers in Luliang City in Yunnan Province]. Soft Science of Health. 2011;25(2):115-8.
418. Liu CQ, Hou WJ, Kong XS, Su J, Xi JM, Zhang JX, et al. [Evaluation on HIV prevention project for high risk populations in Chengjiang County in Yunnan Province]. Soft Science of Health. 2010;24(4):335-8.
419. Lu P, Li C, Tang Z, Tan Q, Li D. [HIV/AIDS behavioral survey among female sex workers in countryside area of Panzhihua city]. Journal of Preventive Medicine Information. 2008;24(8):608-10.
420. Luo Y, Lai W, Deng B, Xi J, Zhang L, Pan X. [Survey of AIDS-related behavior of sex workers in a certain place in Sichuan province]. Morden Preventive Medicine. 2010;37(2):210-2.
421. Peng H, Fu G, Feng Y, Tian X, Feng J, Zhang Q, et al. [A Survey on CSW's AIDS knowledge, behavior and intervention in an urban district in Nanchong]. Journal of North Sichuan Medical College. 2007;22(05):428-31.
422. Wen TJ, He TT, Liu Y. Survey on HIV/AIDS-related knowledge and behaviors of sex workers, drug addicts in Renhe district of Panzhihua. Modern Preventive Medicine. 2009;36(15):2907-9.
423. Wu C, Zhang J, Fan S, Wei D, Li C, Chen K, et al. [A study on HIV/SYPHILIS prevalence and related knowledge and behaviors and their influence factors among female commercial sex workers in a city]. Modern Preventive Medicine. 2010;37(03):499-501.
424. Wu Q, Liu Q. [An analysis of sexual behaviours among female sex workers in urban Luzhou City]. Journal of Preventive Medicine Information. 2004;20(5):555-7.
425. Yang JF, Hu AY, Zhao CZ, Guo JH, Peng JY, Liu L, et al. [Data analysis of HIV prevalence and related behaviors among female sex workers in Baoshan of Yunnan Province from 2007 to 2011]. Chinese Journal of AIDS & STD. 2012;18(7):454-6.
426. Zhang C. [Surveillance report of HIV and syphilis among the commercial sex workers in the entertainment establishments in Gucheng District]. Soft Science of Health. 2008;22(3):269-70.
427. Zhou SX, Mei YY, Wang CZ, Chen Y, Liu M, Guo K. [Analysis on AIDS relative knowledge and higher risk behaviors in unlicensed prostitutes]. Parasitoses and Infectious Diseases. 2007;5(4):206-8.

428. Chen Y, Xiong H, Xiong F. [AIDS knowledge and behavior of female sexual workers in rural areas]. China Tropical Medicine. 2009;9(8):1645-6.
429. Dong L, Li Q, Chen X, Zhou M, Xie Y. [Surveillance of behavior among female sex workers in Zigong, 2008-2009]. Journal of Preventive Medicine Information. 2010;26(12):982-5.
430. Fu JB, Liu LJ. [Survey on AIDS related knowledge and behaviour amongst 100 roadside female sex workers]. Zhejiang Journal of Preventive Medicine. 2010;22(5):79-80.
431. Gan CG, Zhang TS. [Analysis on HIV/AIDS-related knowledge and behaviors among female commercial sex workers in Jiangyou City]. Journal of Southwest University for Nationality (Natural Science Edition). 2010;36(5):866-9.
432. Li L, Chen L, Yang Y, Che Z, Chen Y, Chu C. [Population scale estimate. HIV/AIDS knowledge awareness rate and behavior risk factors survey of female commercial sex workers (FCSWs) in Kunming 2008]. Chinese Journal of AIDS & STD. 2009;31(2):1-3.
433. Li S, Wang Q, Xiang G, Li Y. [Effectiveness analysis of comprehensive HIV interventions for high-risk behaviours among female sex workers in Gaoming county, China]. Soft Science of Health. 2010;24(3):259-61.
434. Li W. [HIV sentinel surveillance and analysis among female sex workers in Jianshui county, China]. Soft Science of Health. 2009;23(06):711-3.
435. Wu Y, Luo GY, Wan SP, Zhou H. [Analysis the trends of the prevalence of AIDS and syphilis among female sex workers in Hongya county from 2008 to 2010]. Journal of North Sichuan Medical College. 2012;27(5):441-4.
436. Yang G, Deng S, Liu H, Wu W, Zhao S, Liu Z. [Survey on AIDS Related K nowledge and Behavior Among Unl icensed Prostitutes in Cangxi County]. Practical Preventive Medicine. 2009;16(6):1966-8.
437. Yang ZJ, Yin ZL, Li ZL, Liu B, Fang KF, Chu XQ. [Investigation on HIV/AIDS epidemic among female sex workers in entertainment establishments in Ruili City, Yunnan Province]. Soft Science of Health. 2010;24(4):373-5.
438. Zhou Y, Fan SF, Liu P, Zhong ML, Luan RS. [Study on condoms access among female commercial sex workers in Fushun county]. Modern Preventive Medicine. 2011;38(5):889-93.
439. Zhu Q, Wang L, He CY, Zhang XB, Yao XZ. [Analysis on behavioural surveillance among 762 female sex workers]. Journal of Dematology and Venereology. 2009;31(2):45-6.
440. Zi G, Yao H, Cha X, Guo J, Zi Z. [Investigation on the Status Related to AIDS among Female Sex Workers in Weishan County in 2008]. Preventive Medicine Tribune. 2009;15(11):1092-3.
441. Dong CL, Hua YJ, Yang YH, Li HL, Zhang Y, He Z. [Study on the effect of condom use promotion among FSWs from Entertainment venues]. Journal of Dermatology and Venereology. 2012;34(5):301-2.
442. Fan S, Liu P, Chen W, Song G, Hu Y, Luan R. [A theory of planned behavior based study on commercial sexual behavior among female commercial sex workers in Fushun county]. Journal of Preventive Medicine Information. 2010;26(02):88-91.
443. He QZ, Lu CM. [A survey on HIV/AIDS knowledge and behavior among female sex workers in Yingkou City in Liaoning Province]. Chinese Journal of Public Health. 2010;26(10):1321.

444. Zhang C, Li X, Hong Y, Stanton B, Chen Y, Zhou Y, et al. Pro-alcohol-use social environment and alcohol use among female sex workers in China: beyond the effects of serving alcohol. World Health Popul. 2012;13(4):15-27.
445. Huang J, Li C, Hu S, Xuan R, Huang J, Sun X, et al. [Analysis of Intervention Effect of AIDS High Risk Behavior among Female Sexual Workers in The Entertainment Places in Qingyuan City]. Journal of Tropical Medicine. 2010;10(9):1122-6.
446. Wong WC, Leung PW, Li CW. HIV behavioural risks and the role of work environment among Chinese male sex workers in Hong Kong. AIDS Care. 2012;24(3):340-7.
447. Fu L, Xia J, Li XL, Qing MH, Zhang JX, Duan B, et al. [Study on KAP for HIV/AIDS among female sex workers and influential factors in Jianyang City] Modern Preventive Medicine. 2012;39(9):2222-4.
448. Liu L, Liang Y, Zhou Z, Liu D, Zhang Y, Zhou Y, et al. [Explore on the Model of HIV/AIDS Comprehensive Intervention among the Female Sex Workers Carried out by NGO]. Preventive Medicine Tribune. 2010;16(7):577-9.
449. Ou Y, Xu F. Surveillance analysis of HIV/AIDS among female sex workers in Dongchuan district of Qunming City in 2009. Soft Science of Health. 2010;24(5):471-3.
450. Wang QF. [Analysis on HIV/AIDS knowledge and behaviour among prostitutes in Songming County, Yunnan Province]. Medical Information. 2010;23(4):928-9.
451. Wang WW, Yu SL, Lin GL. [Analysis on HIV/AIDS sentinel surveillance among high risk populations in Neijiang City, Sichuan Province]. Journal of Preventive Medicine Information. 2010;26(4):309-11.
452. Xue HM, Duo L, Zhu ZB, Yang LH, Deng L, Lin B. [Analysis on HIV infection and related risk factors among China-Vietnam cross border prostitutes]. Soft Science of Health. 2011;25(2):107-8.
453. Yan WZ, Zheng KQ, Feng DL, Cai XY, Liu JR, Long Y, et al. [Investigation on status of STDs and AIDS among female sex workers in JingHong County of Yuinan in 2009]. Journal of Dermatology and Venereology. 2011;33(1):51+4.
454. Yang YH, Wang C, Li HL, Dong CL, Zhang Y, Xie B, et al. [Study on the method and effect of 100% condom use promotion in entertainment venue in Pu'er City]. Soft Science of Health. 2012;26(8):727-9.
455. Yu K, Wang C, Qiu G, Li X, Wang L, Gao L, et al. [Results from Sentinel Surveillance of HIV/AIDS among Commercial Sex Workers in Lancang County in 2009]. Practical Preventive Medicine. 2010;17(11):2303-5.
456. Wangmo T, Zhou YL, Zhang XH, Liang SF, Zhang S. [Analysis of results of sentinel monitoring on FSWs in Lhasa City]. Jiangsu Journal of Preventive Medicine. 2012;23(6):41-2.
457. Guo HJ, Feng D, Chen ZY, Zhou CX, Sun X, Chen ZM. [AIDS Knowledge Levels and Behavior Characteristics of FSWs in Places of Entertainment in Zunyi City]. Occupation and Health. 2011;27(22):2603-4.
458. Yang Y, Li HL, Yang YH. [Surveillance of HIV/AIDS among FSWs in Lahuzu county, Yuinan]. Soft Science of Health. 2012;26(10):911-2.
459. Yang ZJ, Yin ZL, Li ZL, Liu B, Fang KF, Li RC, et al. [Analysis of AIDS Sentinel Surveillance in Ruili City in 2010]. Soft Science of Health. 2012;26(9):785-7.
460. Ouyang H. [Analysis of STDs infection and HIV-related knowledge among commercial sex workers in Hejiang in 2011]. Journal of Occupational Health and Damage. 2012;27(2):126-7.
461. Zhang H, Li HL, Wang C, Yang YH. [Investigation on HIV/AIDS know ledge and related behavior of 1301 female commercial sex workers]. Soft Science of Health. 2012;26(1):47-9.
