# Supplementary material for: Risk Behaviours among Female Sex Workers in China: A Systematic Review and Data Synthesis
Source: PLoS One. 2015 Mar 27;10(3):e0120595. doi: 10.1371/journal.pone.0120595 (PMC4376708; doi:10.1371/journal.pone.0120595)
Supplement: S4 Table — (PDF) [file pone.0120595.s006.pdf]

Table S4. Studies reported rate of condom use in female sex workers with any male partners.

First author, published year	Study period	Location	Province	Region	Recruitment venue	Sampling method	Measurement period*	Number of FSW used condom	Total number of FSW	Condom Usage (%)	QA Score
Chen YL, 2005 [1]	2000/08-2000/10	Longyan	Fujian	East	Entertainment	--	P1M	18	111	16.2%	5
Li YH, 2006 [2]	2000	Fu'an, Ningde	Fujian	East	Entertainment	--	LA	88	200	44.0%	5
Li YH, 2006 [2]	2000	Fu'an, Ningde	Fujian	East	Entertainment	--	P1M	47	200	23.5%	5
Yan JP, 2004 [3]	2000	Longyan	Fujian	East	Entertainment	--	P1M	18	117	15.4%	2
Yan JP, 2004 [3]	2000	Longyan	Fujian	East	Entertainment	--	LA	44	117	37.6%	2
Yan JP, 2006 [4]	2000	Longyan	Fujian	East	Entertainment	--	LA	47	111	42.3%	4
Zhu TW, 2006 [5]	2000	Linyi	Shandong	East	Entertainment	--	P1M	53	323	16.4%	3
Chen LM, 2004 [6]	2001/10-2001/12	Changle	Fujian	East	Entertainment	--	P1M	166	358	46.4%	3
Lin ZM, 2005 [7]	2001	Zhangping	Fujian	East	Entertainment	--	P1M	41	203	20.2%	4
Yan JP, 2004 [3]	2001	Longyan	Fujian	East	Entertainment	--	P1M	115	305	37.7%	2
Yan JP, 2004 [3]	2001	Longyan	Fujian	East	Entertainment	--	LA	130	305	42.6%	2
Yan JP, 2006 [4]	2001	Longyan	Fujian	East	Entertainment	--	P1M	90	291	30.9%	4
Yan JP, 2006 [4]	2001	Longyan	Fujian	East	Entertainment	--	LA	149	291	51.2%	4
Wei XM, 2004 [8]	2001/12-2002/03	Quanzhou	Fujian	East	Entertainment	--	LA	150	657	22.8%	2
Wei XM, 2004 [8]	2001/12-2002/03	Quanzhou	Fujian	East	Entertainment	--	LA	297	384	77.3%	2
Yan JP, 2004 [3]	2002	Longyan	Fujian	East	Entertainment	--	P1M	96	240	40.0%	2
Yan JP, 2004 [3]	2002	Longyan	Fujian	East	Entertainment	--	LA	155	240	64.6%	2
Yan JP, 2006 [4]	2002	Longyan	Fujian	East	Entertainment	--	LA	140	229	61.1%	4
Lin ZM, 2005 [7]	2003	Zhangping	Fujian	East	Entertainment	--	P1M	63	216	29.2%	4
Xie HY, 2004 [9]	2000-2003	Quanzhou	Fujian	East	Detention Center	--	P1M	507	1996	25.4%	7
Yan JP, 2004 [3]	2003	Longyan	Fujian	East	Entertainment	--	P1M	174	276	63.0%	2
Yan JP, 2004 [3]	2003	Longyan	Fujian	East	Entertainment	--	LA	189	276	68.5%	2

First author, published year	Study period	Location	Province	Region	Recruitment venue	Sampling method	Measurement period*	Number of FSW used condom	Total number of FSW	Condom Usage (%)	QA Score
Yan JP, 2006 [4]	2003	Longyan	Fujian	East	Entertainment	--	P1M	80	217	36.9%	4
Yan JP, 2006 [10]	2003	Longyan	Fujian	East	Entertainment	--	LA	150	217	69.1%	4
Liang WC, 2005 [10]	2003-2004	Anyuan	Jiangxi	East	Entertainment	Random sampling	P1M	95	404	23.5%	3
Liang WC, 2005 [10]	2003-2004	Anyuan	Jiangxi	East	Entertainment	Random sampling	LA	177	404	43.8%	3
Wu WH, 2010 [11]	2005	Feixi	Anhui	East	Entertainment	--	LA	78	100	78.0%	3
Yan JP, 2006 [4]	2005	Longyan	Fujian	East	Entertainment	--	P1M	49	72	68.1%	4
Yan JP, 2006 [4]	2005	Longyan	Fujian	East	Entertainment	--	LA	56	72	77.8%	4
Dou ZD, 2012 [12]	2006/07-2006/09	Wu Hu	Anhui	East	Sentinel sites	Cluster sampling	LA	349	394	88.6%	4
Dou ZD, 2012 [12]	2006/07-2006/09	Wu Hu	Anhui	East	Sentinel sites	Cluster sampling	P1M	237	394	60.2%	4
Hu JL, 2007[13]	2006/04-2006/05	Huaian	Anhui	East	Entertainment	Random sampling	LA	133	256	52.0%	5
Wu WH, 2010 [11]	2006	Feixi	Anhui	East	Entertainment	--	LA	108	130	83.1%	3
Xu XF, 2010 [14]	2006	Shanghai	Shanghai	East	Entertainment	Random sampling	LA	32	125	25.6%	3
Dou ZD, 2012 [12]	2007/07-2007/09	Wu Hu	Anhui	East	Sentinel sites	Cluster sampling	LA	304	381	79.8%	4
Dou ZD, 2012 [12]	2007/07-2007/09	Wu Hu	Anhui	East	Sentinel sites	Cluster sampling	P1M	214	381	56.2%	4
Wu WH, 2010 [11]	2007	Feixi	Anhui	East	Entertainment	--	LA	108	125	86.4%	3
Dou ZD, 2012 [12]	2008/04-2008/06	Wu Hu	Anhui	East	Sentinel sites	Cluster sampling	LA	342	402	85.1%	4
Dou ZD, 2012 [12]	2008/04-2008/06	Wu Hu	Anhui	East	Sentinel sites	Cluster sampling	P1M	218	402	54.2%	4
Wu WH, 2010 [11]	2008	Feixi	Anhui	East	Entertainment	--	LA	141	153	92.2%	3
Cao DZ, 2011 [15]	2009/08-2009/11	Maanshan	Anhui	East	Entertainment	--	LA	83	154	53.9%	3

First author, published year	Study period	Location	Province	Region	Recruitment venue	Sampling method	Measurement period*	Number of FSW used condom	Total number of FSW	Condom Usage (%)	QA Score
Dou ZD, 2012 [12]	2009/04-2009/06	Wu Hu	Anhui	East	Sentinel sites	Cluster sampling	LA	332	412	80.6%	4
Dou ZD, 2012 [12]	2009/04-2009/06	Wu Hu	Anhui	East	Sentinel sites	Cluster sampling	P1M	214	412	51.9%	4
Hao XG, 2010 [16]	2009/04-2009/07	Quzhou	Zhejiang	East	Entertainment	Random sampling	LA	104	130	80.0%	4
Hao XG, 2010 [16]	2009/04-2009/07	Quzhou	Zhejiang	East	Entertainment	Random sampling	LA	88	103	85.4%	4
Hao XG, 2010 [16]	2009/04-2009/07	Quzhou	Zhejiang	East	Entertainment	Random sampling	P1M	86	130	66.2%	4
Hao XG, 2010 [16]	2009/04-2009/07	Quzhou	Zhejiang	East	Entertainment	Random sampling	P1M	68	103	66.0%	4
Wu WH, 2010 [11]	2009	Feixi	Anhui	East	Entertainment	--	LA	128	137	93.4%	3
Dou ZD, 2012 [12]	2010/04-2010/06	Wu Hu	Anhui	East	Sentinel sites	Cluster sampling	LA	363	412	88.1%	4
Dou ZD, 2012 [12]	2010/04-2010/06	Wu Hu	Anhui	East	Sentinel sites	Cluster sampling	P1M	245	412	59.5%	4
Ma P, 2012 [17]	2010	Nan Tong	Jiangsu	East	Mixed venues	--	P1M	245	798	30.7%	3
Tang ZL, 2011 [18]	2010/03-2010/07	Qing Dao	Shandong	East	Entertainment	--	P1M	102	216	47.2%	4
Tang ZL, 2011 [18]	2010/03-2010/07	Qing Dao	Shandong	East	Entertainment	--	P1M	132	322	41.0%	4
Tang ZL, 2011 [18]	2010/03-2010/07	Qing Dao	Shandong	East	Entertainment	--	P1M	30	106	28.3%	4
Tang ZL, 2011 [18]	2010/03-2010/07	Qing Dao	Shandong	East	Entertainment	--	LA	69	106	65.1%	4
Tang ZL, 2011 [18]	2010/03-2010/07	Qing Dao	Shandong	East	Entertainment	--	LA	177	216	81.9%	4
Tang ZL, 2011 [18]	2010/03-2010/07	Qing Dao	Shandong	East	Entertainment	--	LA	246	322	76.4%	4

First author, published year	Study period	Location	Province	Region	Recruitment venue	Sampling method	Measurement period*	Number of FSW used condom	Total number of FSW	Condom Usage (%)	QA Score
Dou ZD, 2012 [12]	2006-2011	Wu Hu	Anhui	East	Sentinel sites	Cluster sampling	LA	2049	2394	85.6%	4
Dou ZD, 2012 [12]	2011/04-2011/06	Wu Hu	Anhui	East	Sentinel sites	Cluster sampling	LA	359	405	88.6%	4
Dou ZD, 2012 [12]	2011/04-2011/06	Wu Hu	Anhui	East	Sentinel sites	Cluster sampling	P1M	271	405	66.9%	4
Hao XG, 2012 [19]	2011	Qu Zhou	Zhejiang	East	Sentinel sites	Cluster sampling	LA	324	398	81.4%	4
Hao XG, 2012[19]	2011	Qu Zhou	Zhejiang	East	Sentinel sites	Cluster sampling	P1M	317	377	84.1%	4
Ling Z, 2012 [20]	2011/12	Long You	Zhejiang	East	Entertainment	--	LA	124	275	45.1%	4
Liu HT, 2012 [21]	2011/05-2011/07	Pu Dong	Shanghai	East	Entertainment	--	P1M	14	71	19.7%	3
Liu HT, 2012 [21]	2011/05-2011/07	Pu Dong	Shanghai	East	Entertainment	--	P1M	26	73	35.6%	3
Liu HT, 2012 [21]	2011/05-2011/07	Pu Dong	Shanghai	East	Entertainment	--	LA	51	93	54.8%	3
Liu HT, 2012 [21]	2011/05-2011/07	Pu Dong	Shanghai	East	Entertainment	--	LA	63	98	64.3%	3
Liu YJ, 2007 [22]	2005/09	--	Beijing	North	Entertainment	--	P1M	50	226	22.1%	4
Liu YJ, 2007 [22]	2005/09	--	Beijing	North	Entertainment	--	LA	92	226	40.7%	4
Yan L, 2010 [23]	2006	--	Beijing	North	Entertainment	Random sampling	P1M	13	72	18.1%	4
Yan L, 2010 [23]	2006	--	Beijing	North	Entertainment	Random sampling	LA	44	72	61.1%	4
Di HY, 2012 [24]	2007/12	Shen Ze	Hebei	North	Entertainment	--	P1M	37	120	30.8%	4
Yan L, 2010 [23]	2007	--	Beijing	North	Entertainment	Random sampling	P1M	24	106	22.6%	4
Yan L, 2010 [23]	2007	--	Beijing	North	Entertainment	Random sampling	LA	71	106	67.0%	4

First author, published year	Study period	Location	Province	Region	Recruitment venue	Sampling method	Measurement period*	Number of FSW used condom	Total number of FSW	Condom Usage (%)	QA Score
Miao XF, 2009 [25]	2008	Tangxian, Pingshanxian	Hebei	North	Entertainment	--	LA	185	263	70.3%	5
Miao XF, 2009 [25]	2008	Tangxian, Pingshanxian	Hebei	North	Entertainment	--	P1M	128	263	48.7%	5
Yan L, 2010 [23]	2008	--	Beijing	North	Entertainment	Random sampling	P1M	21	102	20.6%	4
Yan L, 2010 [23]	2008	--	Beijing	North	Entertainment	Random sampling	LA	78	102	76.5%	4
Li YX, 2011[26]	2009/01-2009/06	Shi Jia Zhuang	Hebei	North	Entertainment	--	P1M	5	105	4.8%	3
Xu YJ, 2011 [27]	2009	--	Shanxi	North	Entertainment	--	P1M	3111	4767	65.3%	3
Xu YJ, 2011 [27]	2009	--	Shanxi	North	Entertainment	--	P1M	425	541	78.6%	3
Xu YJ, 2011 [27]	2009	--	Shanxi	North	Entertainment	--	P1M	2686	4226	63.6%	3
Xu YJ, 2011 [27]	2009	--	Shanxi	North	Entertainment	--	LA	4063	4903	82.9%	3
Xu YJ, 2011 [27]	2009	--	Shanxi	North	Entertainment	--	LA	505	569	88.8%	3
Xu YJ, 2011 [27]	2009	--	Shanxi	North	Entertainment	--	LA	3558	4334	82.1%	3
Ji CH, 2009 [28]	2004	Gaoping	Shaanxi	Northwest	Entertainment	--	LA	207	296	69.9%	4
Ji CH, 2009 [28]	2004	Gaoping	Shaanxi	Northwest	Entertainment	--	P1M	158	296	53.4%	4
Ji CH, 2009 [28]	2005	Gaoping	Shaanxi	Northwest	Entertainment	--	LA	239	305	78.4%	4
Ji CH, 2009 [28]	2005	Gaoping	Shaanxi	Northwest	Entertainment	--	P1M	205	305	67.2%	4
Ji CH, 2009 [28]	2006	Gaoping	Shaanxi	Northwest	Entertainment	--	LA	263	303	86.8%	4
Ji CH, 2009 [28]	2006	Gaoping	Shaanxi	Northwest	Entertainment	--	P1M	219	303	72.3%	4
A SI YA, 2011 [29]	2008/07	Tu Lu Fan	Xinjiang	Northwest	--	--	P1M	73	200	36.5%	2
He B, 2006 [30]	2002/03	Pingxiang	Guangxi	South Central	Entertainment	--	LA	112	167	67.1%	4
He QY, 2003 [31]	2002/09	Zhanzhou	Hainan	South Central	Entertainment	--	LA	57	337	16.9%	5
Hu B, 2004 [32]	2002	Zhanzhou	Hainan	South Central	Entertainment	Random sampling	LA	56	393	14.2%	5

First author, published year	Study period	Location	Province	Region	Recruitment venue	Sampling method	Measurement period*	Number of FSW used condom	Total number of FSW	Condom Usage (%)	QA Score
Fan XJ, 2007 [33]	2003	Shimen	Hunan	South Central	Entertainment	--	LA	21	109	19.3%	5
Li N, 2005 [34]	2003/04	Henan	Henan	South Central	--	Cluster Random sampling	LA	1557	2761	56.4%	5
Li N, 2005 [34]	2003/04	Henan	Henan	South Central	--	Cluster Random sampling	P1M	815	2761	29.5%	5
Wei MG, 2004 [35]	2003	Haikou, Sanya	Hainan	South Central	Entertainment	--	LA	27	338	8.0%	3
Wei MG, 2004 [35]	2003	Haikou, Sanya	Hainan	South Central	Entertainment	--	P1M	9	338	2.7%	3
Chen M, 2007 [36]	2004/05	Wuhan	Hubei	South Central	Entertainment	--	P1M	64	152	42.1%	2
Chen M, 2007 [36]	2004/05	Wuhan	Hubei	South Central	Entertainment	--	LA	89	152	58.6%	2
Chen M, 2007 [36]	2005/07	Wuhan	Hubei	South Central	Entertainment	--	P1M	118	145	81.4%	2
Chen M, 2007 [36]	2005/07	Wuhan	Hubei	South Central	Entertainment	--	LA	122	145	84.1%	2
Duane KM, 2007 [37]	2006/03	Chenzhou	Hunan	South Central	Entertainment	--	LA	76	340	22.4%	3
Duane KM, 2007 [37]	2006/03	Chenzhou	Hunan	South Central	Entertainment	--	P1M	11	340	3.2%	3
Li MQ, 2007 [38]	2006	Liuzhou	Guangxi	South Central	Entertainment	Random sampling	LA	136	201	67.7%	4
Li XX, 2007[39]	2006/01	Binyang, Nanning	Guangxi	South Central	Entertainment	--	LA	155	364	42.6%	3
Xu Cj, 2007 [40]	2006	Zhou	Hubei	South Central	Entertainment	Random sampling	P1M	51	54	94.4%	3

First author, published year	Study period	Location	Province	Region	Recruitment venue	Sampling method	Measurement period*	Number of FSW used condom	Total number of FSW	Condom Usage (%)	QA Score
Xu Cj, 2007 [40]	2006	Ezhou	Hubei	South Central	Entertainment	Random sampling	P1M	31	45	68.9%	3
Xu Cj, 2007 [40]	2006	Ezhou	Hubei	South Central	Entertainment	Random sampling	P1M	24	39	61.5%	3
Zhang JL, 2008 [41]	2006/01-2006/10	Hezhou	Guangxi	South Central	Entertainment	--	P1M	132	203	65.0%	3
Zhang JL, 2008 [41]	2006/01-2006/10	Hezhou	Guangxi	South Central	Entertainment	--	LA	153	200	76.5%	3
Zhang YY, 2007 [42]	2006/03	Huaihua	Hunan	South Central	Entertainment	--	LA	110	145	75.9%	1
Qin WW, 2011 [43]	2007	Nan Ning	Guangxi	South Central	Entertainment	--	LA	277	379	73.1%	4
Wang XX, 2008 [44]	2006-2007	Dongwan	Guangdong	South Central	Detention Center	--	LA	143	228	62.7%	6
Zhang SJ, 2008 [45]	2007	Congzuo	Guangxi	South Central	VCT	--	P1M	37	385	9.6%	4
Zhang SJ, 2008 [45]	2007	Congzuo	Guangxi	South Central	VCT	--	LA	260	385	67.5%	4
Zhao SH, 2007 [46]	2006/01-2007/10	Xiangtan	Hunan	South Central	Detention Center	--	LA	52	140	37.1%	4
Zhu BY, 2012 [47]	2007/05-2007/10	Liuzhou, Beihai, Chongzuo, Guigang	Guangxi	South Central	Entertainment	Venue-based sampling	LA	298	488	61.1%	6
Qin WW, 2011 [43]	2008	Nan Ning	Guangxi	South Central	Entertainment	--	LA	323	400	80.8%	4
Wu SX, 2010 [48]	2008-10	Sanmenxia	Henan	South Central	Entertainment	Random sampling	LA	32	125	25.6%	6
Liao S, 2011 [49]	2008-2009	--	Hainan	South Central	Entertainment	Venue-based sampling	P1M	120	157	76.4%	4

First author, published year	Study period	Location	Province	Region	Recruitment venue	Sampling method	Measurement period*	Number of FSW used condom	Total number of FSW	Condom Usage (%)	QA Score
Qin WW, 2011 [43]	2009	Nan Ning	Guangxi	South Central	Entertainment	--	LA	304	400	76.0%	4
Qin WW, 2011 [43]	2007-2009	Nan Ning	Guangxi	South Central	Entertainment	--	LA	904	1179	76.7%	4
Zhou Y, 2012 [50]	2009/12	Gui Lin	Guangxi	South Central	Entertainment	--	P1M	194	292	66.4%	4
Liao S, 2011 [49]	2009 - 2010	--	Guangxi	South Central	Entertainment	Venue-based sampling	P1M	127	155	81.9%	4
Tang MJ, 2011 [51]	2004-2010	Yu Lin	Guangxi	South Central	Entertainment	--	LA	605	633	95.6%	3
Tang MJ, 2011 [51]	2004-2010	Yu Lin	Guangxi	South Central	Entertainment	--	LA	1257	1479	85.0%	3
Tang MJ, 2011 [51]	2004-2010	Yu Lin	Guangxi	South Central	Entertainment	--	LA	1905	2111	90.2%	3
Tang MJ, 2011 [51]	2004-2010	Yu Lin	Guangxi	South Central	Entertainment	--	LA	3767	4223	89.2%	3
Wu ZZ, 2011 [52]	2009/07-2010/01	Jiangmen	Guangdong	South Central	Entertainment	Convenience sampling	P1M	497	702	70.8%	6
Wu ZZ, 2011 [53]	2009/07-2010/01	Jiang Men	Guangdong	South Central	Entertainment	Convenience sampling	P1M	401	542	74.0%	6
Wu ZZ, 2011 [53]	2009/07-2010/01	Jiang Men	Guangdong	South Central	Entertainment	Convenience sampling	P1M	497	702	70.8%	6
Wu ZZ, 2011 [53]	2009/07-2010/01	Jiang Men	Guangdong	South Central	Entertainment	Convenience sampling	P1M	48	93	51.6%	6
Wu ZZ, 2011 [53]	2009/07-2010/01	Jiang Men	Guangdong	South Central	Entertainment	Convenience sampling	P1M	48	67	71.6%	6
Zeng JJ, 2012 [54]	2010	Hui Zhou	Guangdong	South Central	Entertainment	Cluster sampling	LA	126	325	38.8%	4
Zeng JJ, 2012 [54]	2010	Hui Zhou	Guangdong	South Central	Entertainment	Cluster sampling	P1M	135	325	41.5%	4

First author, published year	Study period	Location	Province	Region	Recruitment venue	Sampling method	Measurement period*	Number of FSW used condom	Total number of FSW	Condom Usage (%)	QA Score
Huang GL, 2012 [55]	2011/07-2011/09	--	Guangxi	South Central	--	--	LA	281	301	93.4%	0
Liang X, 2005 [56]	2003/12	Chengdu	Sichuan	Southwest	Entertainment	Venue-based sampling	LA	115	301	38.2%	4
Jiang H, 2009 [57]	2005	Xingwen	Sichuan	Southwest	Entertainment	--	LA	18	162	11.1%	5
Li SS, 2006 [58]	2005/01-2006/04	Yuxi	Yunnan	Southwest	Entertainment	--	LA	40	101	39.6%	4
Yang HW, 2005 [59]	2003-2007	Mianyang	Sichuan	Southwest	Entertainment	--	P1M	113	265	42.6%	2
Yang HW, 2005 [59]	2003-2007	Mianyang	Sichuan	Southwest	Entertainment	--	P1M	29	99	29.3%	2
Yang HW, 2005 [59]	2003-2007	Mianyang	Sichuan	Southwest	Entertainment	--	LA	199	265	75.1%	2
Yang HW, 2005 [59]	2003-2007	Mianyang	Sichuan	Southwest	Entertainment	--	LA	62	99	62.6%	2
Zhu Q, 2009 [60]	2008	Chuxiong	Yunnan	Southwest	Entertainment	--	P1M	529	762	69.4%	4
Zhou Z, 2012 [61]	2010/04-2010/06	Da Li	Yunnan	Southwest	Entertainment	--	LA	1873	2019	92.8%	4
Zhou Z, 2012 [61]	2010/04-2010/06	Da Li	Yunnan	Southwest	Entertainment	--	P1M	1668	2019	82.6%	4
Li YK, 2011 [62]	2011/05-2011/09	8 counties	Sichuan	Southwest	--	--	LA	318	368	86.4%	3
Li YK, 2011 [62]	2011/05-2011/09	8 counties	Sichuan	Southwest	--	--	P1M	190	368	51.6%	3

*LA: last sex act; P1M: in the past one month prior to the survey

References

1. Chen YL, Yan JP, Li TR, Lian JJ, Lin MY, Chen QJ. [Surveillance of risk behaviors facilitating HIV/AIDS transmission among female sex workers in entertainment venues in Longyan City of Fujian Province]. Practical Preventive Medicine. 2005;12(6):1366-7.
2. Li Y, Lin S, Zheng X. [Effectiveness evaluation of health education and behavioural interventioins among female sex workers in Fu'an prefecture, China]. Strait Journal of Preventive Medicine. 2006;12(2):77-8.
3. Yan JP, Lan JJ, Li TR, Chen YL. [Surveillance on HIV/AIDS surveillance and risk behaviour among female working in entertainment venues in urban area of Longyan City, Fujian Province]. Preventive Medicine Tribune. 2004;10(5):560-1.
4. Chen YL, Yan JP, Li TR, Lin MY, Chen CJ. [HIV/AIDS knowledge and risk behavior in female workers in recreation service in Longyan, 2000-2005]. South China Journal of Preventive Medicine. 2006;32(1):20-2.
5. Zhu T. [The Analysis of AIDS Surveil lance among Female Sex Workers in Some City]. Journal of Community Medicine. 2006;4(13):3-4.
6. Chen LM, Lin LY, Lin JC, Chen JY, Gao W. [Investigation on HIV/AIDS related risk behaviors and factors among female sex workers in entertainment venues]. Strait Journal of Preventive Medicine. 2004;10(2):58-9.
7. Lin Z, Li Y. [A behavioural study on female sex workers in entertainment centres in Zhangping City in 2001-2003]. Strait Journal of Preventive Medicine. 2005;11(4):32-3.
8. Wei XM, Zhao JY, Xu YY, Hong YJ, Huang XR. [Knowledge, attitude, and practices (KAP) study on HIV/AIDS among female attendants in entertainment places]. Strait Journal of Preventive Medicine. 2004;10(2):57-8.
9. Xie HY. [Investigation on HIV and syphilis infection among 1996 female sex workers in Quanzhou City, Fujian Province]. Strait Journal of Preventive Medicine. 2004;10(6):44-5.
10. Liang W. [Effectiveness evaluation of HIV interventions for sex workers in Anyuan district, Jiangxi province]. Medicine Healthcare Apparatus. 2005;(12):76-7.
11. Wu W, Luo Y, Wu S. [Evaluation on interventional effects to high-risk behaviors among sex workers in Feixi County]. Anhui Journal of Preventive Medicine. 2010;16(5):378-9.
12. Dou ZD, HE JG, Jin YL, Fang YJ, An Z. [Study of effects of HIV intervention in female sex workers in Wuhui]. Anhui Journal of Preventive Medicine. 2012;18(5):326-8+31.

13. Hu J, Liu L, Ge H, Zhang Z, Zhang Q, Zhang X. [Investigation on knowledge, behavior about STD/AIDS HIV infection situation of 256 female commercial sex workers]. Modern Preventive Medicine. 2007;34(13):2521-3.
14. Xu XF, Tian FQ. [Evaluation of behavioral intervention related to AIDS among female sex workers in entertainment places]. Henan Journal of Preventive Medicine. 2010;21(3):232-4.
15. Cao DZ, Zhan SW, Qin QR, Xu XQ, Cui XS. [Knowledge, awareness and risk behavior related to HIV/ AIDS among female sex workers in low class establishments in Maanshan of China]. Chinese Rural Health Service Administration. 2011;31(4):389-90.
16. Hao XG. [A survey on HIV/AIDS knowledge among female sex workers in Kecheng District, Zhejiang Province]. Zhejiang Journal of Preventive Medicine. 2010;22(11):73-4+7.
17. Li F, Ma SB, Liu HX. [Analysis of AIDS sentinel surveillance of Changping District in Beijing,2011]. Chinese Journal of Health Education. 2012;28(6):469-71+75.
18. Tang ZL, Li XF, Dong XP, Wang YF, Chen GZ. [Infection of HIV/STDs through sexual behavior among female sex workers during menstruation]. Chinese Journal of Public Health. 2011;27(12):1510-2.
19. Hao XG, Wang W. [Sentinel surveillance of HIV/AIDS in female sex workers in Kecheng district of Quzhou city, Zhejiang province, 2011]. Disease Surveillance. 2012;27(4):300-3.
20. Ling Z, Wu JJ. [Survey on AIDS-related Knowledge and Behaviour among CSWs in Entertainment Venues]. Zhejiang Journal of Preventive Medicine. 2012;24(12):87-8.
21. Liu HT, Wang SM, Chen XT, Lu S, Zhang X, Li P. [Investigation of AIDS KAP Intervention among Two Communities Female Sex Workers in Pudong New District, Shanghai]. Health Education and Health Promotion. 2012;7(3):182-5.
22. Liu Y, Ding H, Yu S. [The effects of sexually transmitted infections(STI)/AIDS behavioral intervention among female sex workers]. Chin J Prev Med. 2007;41(06):492-5.
23. Yan L, Liu H, Li Y, Li C. [Dynamic Analysis of AIDS Knowledge and Risk Behaviors among Female Sex Workers]. Occupation and Health. 2010;26(10):1132-4.
24. Di HY, Liu XS, Zhao R. [Analysis of Intervention Effect of AIDS-related behaviors among CSWs in Shenzhe County, Hebei Province]. Chinese Journal of Pest Control. 2012;28(10):1157-8.
25. Miao X, Zhao H, Zong X, Wang A. [Analysis on the character of commercial sex workers in cheap entertainment places in rural areas]. Chinese Journal of Disease Control & Prevention. 2009;13(4):420-2.

26. Liao M, Jiang Z, Zhang X, Kang D, Bi Z, Liu X, et al. Syphilis and methamphetamine use among female sex workers in Shandong Province, China. Sex Transm Dis. 2011;38(1):57-62.
27. Xu YJ, Wang SP, Xue ZD, Shen JP. [An investigation of awareness rate on HIV/AIDS-related knowledge and risky sexual behaviours of unlicensed prostitutes in Shanxi province in 2009]. Chinese Remedies & Clinics. 2011;11(3):304-6.
28. Ji CH, Ren XP, Shen JP, Zhao LH, Feng Y, Hao ZW. [Analysis of STD and AIDS of commercial sex workers in entertainment places in Gaoping city from 2004 to 2006]. Preventive Medicine Tribune. 2009;15(10):918-9.
29. Abudureyimu AYS, Amuti DLK. [Evaluation of AIDS intervention among road-sided prostitutes in Turpan City]. Xinjiang Medical Journal. 2011;41(3):58-9.
30. He B, Nong LP, Zhou YJ, Liang SL, Bi SZ, Liang FQ, et al. [Evaluation on the effectiveness of HIV/AIDS health education and condom use intervention among commerical female sex workers] Guangxi Journal of Preventive Medicine. 2006;12(1):56-8.
31. He Q, Lin J, Zeng X, Yang J, Zhang F, Chen S, et al. [Analysis on STD and HIV infection situation in 417 female sex workers]. Chinese Journal of Public Health. 2003;19(9):1096-7.
32. HU B, Lin J, Feng Y, Yang J, He Q. [Analysis of results in popularization the condoms in entertainment places for prevention of STD]. Chin Tropical medicine. 2004;4(4):658-9.
33. Fan XJ, Li XP, Du DC, Du JG, Wang ZH. [Evaluation of behavioural intervention for female sex workers in entertainment sites in Shimen County in Hunan Province]. Chinese Journal of AIDS & STD. 2007;13(S1):26-7.
34. Li N, Wang Z. [The survey of HIV prevalence among commercial sex workers in Henan Province]. Henan J Prev Med. 2005;16(04):213-4.
35. Wei M, Li J, Xu D, Zhang F, Zeng X. [Epidemic survey among female sex workers and drug users in Hainan province in 2003]. Hai Nan Medical Journal. 2004;15(11):107-8.
36. Che M, Li L, Long JQ. [Evaluation of AIDS/STD behavior intervention among entertainment service Miss] Modern Preventive Medicine. 2007;34(13):2544-5.
37. Duan KM, Tang XY, Hou SQ, Tang JH. [Evaluation of the impact of health education on prostitutes in entertainment venues]. Practical Preventive Medicine. 2007;14(4):1305-7.
38. Li M, Huang Y, Wang Y, Luo L. [Evaluation of health interventions and outreach services among entertainment sex workers in China]. Guangxi Medical Journal. 2007;29(8):1228-9.
39. Li X, Li Q, Wei X. [A study of STD/AIDS knowledge, belief and behaviours among 364 female sex workers]. Applied Prev Med. 2007;13(6):1.

40. Xu CJ, He LS, Li QY. [Investigation on AIDS knowledge and behavior among prostitutes in entertainment sites]. Journal of Public Health and Preventive Medicine. 2007:58-9.
41. Zhang JL, Liao YZ, Huang JW, Mai XY. [Investigation on HIV/STD-related knowledge and behaviours among 203 high-risk female in Hezhou city of Guangxi Zhuang Autonomous Region]. Journal of Applied Preventive Medicine. 2008;14(Z1):2.
42. Zhang YY, Lv HY. [Evaluation of the effectiveness on health education among female sex workers in entertainment venues]. Practical Preventive Medicine. 2007;14(4):1307-8.
43. Tan WW, Liu HY, Liu FX, Huang CH, Zhou HF. [Sentinel surveillance of syphilis in female sex workers in Nanning city, 2007-2009]. Disease Surveillance. 2011;26(2):106-9.
44. Wang XX, Zhang QL, Chen BF, Fang XJ. [Sex criminal HIV / syphilis surveillance report in Dongguan, Guangdong province]. Disease Surveillance. 2008;23(08):490-2.
45. Zhang SJ. [Characteristics and trend of HIV/AIDS epidemic in Chongzuo city of Guangxi, 1996-2007]. Internal Medicine of China. 2008;3(06):932-5.
46. Zhao SH, Wang JJ, Fang ZH, Yan TQ, He W, Peng SY. [Survey on the behavioral characteristics related to HIV/AIDS and the sero-prevalence of sexually transmitted diseases (STD) among 140 female sex workers]. Practical Preventive Medicine. 2007;14(6):1926-7.
47. Zhu BY, Bu J, Huang PY, Zhou ZG, Yin YP, Chen XS, et al. Epidemiology of sexually transmitted infections, HIV, and related high-risk behaviors among female sex workers in Guangxi Autonomous Region, China. Jpn J Infect Dis. 2012;65(1):75-8.
48. Wu SX. [Evaluation of Behavioral Intervention Related to AIDS Among female Sex Workers in Entertainment Places in Sanmenxia downtown area]. Henan Journal of Preventive Medicine. 2010;21(5):369-71.
49. Liao S, Weeks MR, Wang Y, Nie L, Li F, Zhou Y, et al. Inclusion of the female condom in a male condom-only intervention in the sex industry in China: a cross-sectional analysis of pre- and post-intervention surveys in three study sites. Public health. 2011;125(5):283-92.
50. Zhou Y, Chen W, Zhou MR. [Evaluation on AIDS intervention programme among female sex workers in Guilin,2009-1010]. Disease Surveillance. 2012;27(1):35-9.
51. Tang MJ, Zhang DL, Zhong FH, Li L, Li EL, Liu J. [Syphilis and HIV Infection Status Among Commercial Sex Workers in Yulin City from 2004 -2010]. Journal of Applied Preventive Medicine. 2011;17(5):300-1+276.
52. Wu ZZ, Deng WJ, Zhu X, Huang SJ, Chen XS, Jiang N. [Study of commercial sex workers positive of syphilis serum and its related factors]. Qingdao Medical Journal. 2011;42(5):321-4.

53. Wu ZZ, Deng WJ, Zhu X, Huang SJ, Chen XS, Jiang N. [Study of commercial sex workers positive of syphilis serum and its related factors]. Qingdao Medical Journal. 2011;43(5):321-4.
54. Miao XL, Cheng H, Zhang X, Gu J, Ji YY, He EQ. [Analysis on HIV /AIDS Sentinel Surveillance in Wuxi City in 2010]. Occupation and Health. 2011;27(22):2599-601.
55. Huang GL, Zhai QX. [AIDS related knowledge and risk behaviors investigation among the female commercial sex works in a county of Guangxi]. Chinese Journal of Pest Control. 2012;28(5):540-2.
56. Liang X, Tuo XL, Wu DB, Du CH. [Evaluation the integrated intervention on STD/AIDS prevention among female CSWS of Chengdu City]. Modern Preventive Medicine. 2005;32(4):335-6.
57. Jiang H, Luo Y, Liao Y. [Effect analysis of AIDS Intervention among female sex workers in Wenxing County]. Occupation and Health. 2009;25(23):2545-6.
58. Li S, Zhang M, Zhu Y, Li Y. [Evaluation of comprehensive interventions among entertainment venue female sex workers in Xiping county, China]. Soft Science of Health. 2006;20(3):299-300.
59. Yang HW, Zhu XY, Zhang GG. [A survey on HIV/AIDS related knowledge and risk behavior among prostitutes in middle-high class entertainment venues Mianyang City, Sichuan Province]. Journal of Preventive Medicine Information. 2005;21(2):194-6.
60. Zhu Q, Wang L, He CY, Zhang XB, Yao XZ. [Analysis on behavioural surveillance among 762 female sex workers]. Journal of Dematology and Venereology. 2009;31(2):45-6.
61. Zhou Z, Huang LH, Chen ZJ, Lu MJ. [Analysis of surveillance monitoring of illicit prostitutes in Dalian City in 2010]. Soft Science of Health. 2012;26(5):449-51.
62. Nie ZQ, Lin P, Li Y, Wang Y. [Surveillance of AIDS high-risk people in Guangdong province,2009]. Journal of Tropical Medicine. 2011;11(1):29-31, 45.
