# Supplementary material for: Risk Behaviours among Female Sex Workers in China: A Systematic Review and Data Synthesis
Source: PLoS One. 2015 Mar 27;10(3):e0120595. doi: 10.1371/journal.pone.0120595 (PMC4376708; doi:10.1371/journal.pone.0120595)
Supplement: S5 Table — (PDF) [file pone.0120595.s007.pdf]

Table S5. Studies reported the uptake rate of an HIV test in the past 12 months in female sex workers.

First author, published year	Study period	Location	Province	Region	Recruitment venue	Sampling method	Number of FSW tested for HIV	Total number of FSW	Testing rate (%)	QA Score
Chu QS, 2005 [1]	2000	--	Shandong	East	Detention Center	--	17	100	17.0%	3
Zhu XY, 2008 [2]	2004/09-2004/10	Jiaozhou	Shandong	East	Entertainment	--	33	360	9.2%	4
Liao M, 2012 [3]	2006	Dezhu, Yantai, Qingdao	Shandong	East	Entertainment	Venue-based sampling	179	1104	16.2%	5
Wu J, 2006 [4]	2006/06	Yingtan	Jiangxi	East	Entertainment	--	9	284	3.2%	2
Dou ZD, 2012 [5]	2006/07-2006/09	Wu Hu	Anhui	East	Sentinel sites	Cluster sampling	75	394	19.0%	4
Liao M, 2012 [3]	2007	Dezhu, Yantai, Qingdao	Shandong	East	Entertainment	Venue-based sampling	357	1197	29.8%	5
Wang XM, 2011 [6]	2007	Cui Chang	Zhejiang	East	Entertainment	--	62	272	22.8%	4
Guo ZY, 2009 [7]	2007/01	Mengcheng	Anhui	East	Entertainment	--	9	125	7.2%	5
Luo Y, 2008 [8]	2007/02-2007/07	Hangzhou	Zhejiang	East	Entertainment	--	40	257	15.6%	5
Liu YL, 2007 [9]	2007/04-2007/05	Ji'an	Jiangxi	East	Entertainment	--	163	360	45.3%	4
Liu YL, 2007 [9]	2007/04-2007/05	Ji'an	Jiangxi	East	Entertainment	--	163	360	45.3%	4
Dou ZD, 2012 [5]	2007/07-2007/09	Wu Hu	Anhui	East	Sentinel sites	Cluster sampling	135	381	35.4%	4
Tang QR, 2009 [10]	2007/08	Ningbo	Zhejiang	East	--	Random sampling	23	90	25.6%	4
ChenL, 2009 [11]	2008	Shanghai	Shanghai	East	Entertainment	Continuous sampling	140	455	30.8%	5
Liao M, 2012 [3]	2008	Dezhu, Yantai, Qingdao	Shandong	East	Entertainment	Venue-based sampling	552	1159	47.6%	5
Wang XM, 2011 [6]	2008	Cui Chang	Zhejiang	East	Entertainment	--	115	251	45.8%	4
Zhang XJ, 2012 [12]	2006-2008	Qingdao, Yantai,Dezhou	Shandong	East	Entertainment	--	1087	3460	31.4%	4

First author, published year	Study period	Location	Province	Region	Recruitment venue	Sampling method	Number of FSW tested for HIV	Total number of FSW	Testing rate (%)	QA Score
Dou ZD, 2012 [5]	2008/04-2008/06	Wu Hu	Anhui	East	Sentinel sites	Cluster sampling	127	402	31.6%	4
Zhang YH, 2011 [13]	2008/04-2008/07	Nanjing	Jiangsu	East	--	--	24	400	6.0%	2
Zhang YH, 2011 [13]	2008/04-2008/07	Shanghai	Shanghai	East	--	--	79	416	19.0%	2
Zhang YH, 2011 [13]	2008/04-2008/07	Hangzhou	Zhejiang	East	--	--	111	460	24.1%	2
Zhang YH, 2011 [13]	2008/04-2008/07	Qingdao	Shandong	East	--	--	156	400	39.0%	2
Hu CC, 2011 [14]	2008/08	Nan Chang	Jiangxi	East	Entertainment	--	0	42	0.0%	4
Hu CC, 2011 [14]	2008/08	Nan Chang	Jiangxi	East	Entertainment	--	83	199	41.7%	4
Wang XM, 2011 [6]	2009	Cui Chang	Zhejiang	East	Entertainment	--	131	252	52.0%	4
Kang D, 2011 [15]	2006-2009	Qingdao	Shandong	East	Entertainment	Venue-based, community outreach and peer-referral	108	244	44.3%	6
Kang D, 2011 [15]	2006-2009	Qingdao	Shandong	East	Entertainment	Venue-based, community outreach and peer-referral	402	823	48.8%	6
Kang D, 2011 [15]	2006-2009	Qingdao	Shandong	East	Entertainment	Venue-based, community outreach and peer-referral	59	120	49.2%	6
XI SJ, 2011 [16]	2009/04-2009/06	Hangzhou	Zhejiang	East	Entertainment	Cluster sampling	63	446	14.1%	5
XI SJ, 2011 [16]	2009/04-2009/06	Hangzhou	Zhejiang	East	Entertainment	Cluster sampling	63	446	14.1%	5
Xi SJ, 2011 [17]	2009/04-2009/06	Hangzhou	Zhejiang	East	Entertainment	--	63	446	14.1%	4

First author, published year	Study period	Location	Province	Region	Recruitment venue	Sampling method	Number of FSW tested for HIV	Total number of FSW	Testing rate (%)	QA Score
Chen GS, 2010 [18]	2009/04-2009/06	--	Jiangxi	East	Entertainment	Ethnographic target sampling	131	401	32.7%	6
Dou ZD, 2012 [5]	2009/04-2009/06	Wu Hu	Anhui	East	Sentinel sites	Cluster sampling	103	412	25.0%	4
Chen SX, 2011 [19]	2009/07-2009/09	Gao Mi	Shandong	East	Entertainment	--	38	236	16.1%	4
Zhang H, 2011 [20]	2010	Fuzhou	Fujian	East	Entertainment	--	159	479	33.2%	4
Sun XQ, 2011 [21]	2010	Tai He	Anhui	East	Entertainment	--	125	354	35.3%	4
Wang XM, 2011 [6]	2010	Cui Chang	Zhejiang	East	Entertainment	--	196	303	64.7%	4
Qian ZH, 2012 [22]	2009-2010	Suzhou	Jiangsu	East	Detention Center	--	521	578	90.1%	4
Kang DM, 2011 [23]	2009-2010	Dezhou, Dongming, Gaomi, Lue Nan, Longkou, Pingyi, Rushan, Yanggu, Huaiyin	Shandong	East	Entertainment	Cluster, convenience sampling	435	1506	28.9%	3
Kang DM, 2011 [23]	2009-2010	Heze, Caoxian, Chengwu, Zoucheng, Xintai, Pingdu	Shandong	East	Entertainment	Cluster, convenience sampling	755	1157	65.3%	3
Wu SB, 2012 [24]	2010/01-2010/08	15 Counties	Fujian	East	Entertainment	--	203	358	56.7%	4
Dou ZD, 2012 [5]	2010/04-2010/06	Wu Hu	Anhui	East	Sentinel sites	Cluster sampling	200	400	50.0%	4
Jiang J, 2012 [25]	2010/07	Ning Bo	Zhejiang	East	Detention Center	--	166	439	37.8%	4

First author, published year	Study period	Location	Province	Region	Recruitment venue	Sampling method	Number of FSW tested for HIV	Total number of FSW	Testing rate (%)	QA Score
Sun BJ, 2012 [26]	2011	Zao Zhuang	Shandong	East	Entertainment	--	220	372	59.1%	4
Dou ZD, 2012 [5]	2006-2011	Wu Hu	Anhui	East	Sentinel sites	Cluster sampling	624	2394	26.1%	4
Gan WH, 2012 [27]	2009-2011	Jin Shan	Shanghai	East	Sentinel sites	Cluster sampling	353	1240	28.5%	3
Dou ZD, 2012 [5]	2011/04-2011/06	Wu Hu	Anhui	East	Sentinel sites	Cluster sampling	234	405	57.8%	4
Xu JS, 2012 [28]	2011/04-2011/07	--	Jiangsu	East	--	--	729	1591	45.8%	4
Qiu ZH, 2012 [29]	2011/04-2011/07	Hu Zhou	Zhejiang	East	Entertainment	--	251	400	62.8%	4
Xu JS, 2012 [28]	2011/04-2011/07	--	Jiangsu	East	--	--	1233	3754	32.8%	4
Zhu HW, 2012 [30]	2011/04-2011/07	Nan Chang	Jiangxi	East	Entertainment	--	242	400	60.5%	4
Xu JS, 2012 [28]	2011/04-2011/07	--	Jiangsu	East	--	--	1939	5502	35.2%	4
Xu JS, 2012 [28]	2011/04-2011/07	--	Jiangsu	East	--	--	3903	10926	35.7%	4
Chen G, 2012 [31]	2011/07	Dong Yang, Yu Yao, Qu Zhou	Zhejiang	East	--	--	269	514	52.3%	3
Bo FB, 2007 [32]	2006/08-2006/09	Huhehaote	Inner Mongolia	North	Entertainment	Random sampling	25	630	4.0%	3
Ao X, 2008 [33]	2006/08-2006/10	Beijing	Beijing	North	Entertainment	Random sampling	3	105	2.9%	7
Zhang YH, 2011 [13]	2008/04-2008/07	Beijing	Beijing	North	--	--	46	2688	1.7%	2
Zhang YH, 2011 [13]	2008/04-2008/07	Tianjin	Tianjin	North	--	--	13	534	2.4%	2
Li M, 2012 [34]	2009	Xi Cheng	Beijing	North	Entertainment	--	58	200	29.0%	4
Jia J, 2011 [35]	2009	Xian	Shanxi	North	Entertainment	--	164	296	55.4%	3

First author, published year	Study period	Location	Province	Region	Recruitment venue	Sampling method	Number of FSW tested for HIV	Total number of FSW	Testing rate (%)	QA Score
Li M, 2012 [34]	2010	Xi Cheng	Beijing	North	Entertainment	--	70	200	35.0%	4
Li BY, 2012 [36]	2010/04-2010/07	Jin Nan	Tianjin	North	--	--	221	407	54.3%	4
Li M, 2012 [34]	2011	Xi Cheng	Beijing	North	Entertainment	--	144	200	72.0%	4
Qi GH, 2008 [37]	2006/07	Changchun	Jilin	Northeast	Entertainment	Cluster sampling	8	124	6.5%	4
Qi GH, 2008 [37]	2006/07	Changchun	Jilin	Northeast	Entertainment	Cluster sampling	8	122	6.6%	4
Qi GH, 2008 [37]	2006/07	Changchun	Jilin	Northeast	Entertainment	Cluster sampling	116	239	48.5%	4
Zhang YH, 2011 [13]	2008/04-2008/07	Shenyang	Liaoning	Northeast	--	--	48	601	8.0%	2
Zhang YH, 2011 [13]	2008/04-2008/07	Haerbin	Heilongjiang	Northeast	--	--	45	447	10.1%	2
Li Y, 2011[38]	2010	-	Heilongjiang	Northeast	Sentinel sites	--	1916	7662	25.0%	4
Xian XJ, 2011 [39]	2011	Nong An	Jilin	Northeast	Sentinel sites	Cluster sampling	254	400	63.5%	3
Wang XZ, 2005 [40]	2004/05-2004/06	Ningxia	Ningxia	Northwest	Entertainment	Cluster random sampling	31	332	9.3%	5
Amydam MAYT, 2008 [41]	2007	Wulumuqi	Xinjiang	Northwest	Entertainment	Convenience sampling	547	882	62.0%	7
Wang MX, 2008 [42]	2007/07	Shanyang	Shaanxi	Northwest	Entertainment	--	17	30	56.7%	3
Zhang YH, 2011 [13]	2008/04-2008/07	Xian	Shaanxi	Northwest	--	--	4	400	1.0%	2
Chang WH, 2011[43]	2010	--	Shaanxi	Northwest	Sentinel sites	Cluster sampling	1296	4439	29.2%	4
Wu R, 2012 [44]	2010	Bole	Xinjiang	Northwest	Entertainment	--	373	412	90.5%	4
Zhao GD, 2011 [45]	2010/04-2010/06	Shang Luo	Shaanxi	Northwest	Sentinel sites	Cluster sampling	135	412	32.8%	4

First author, published year	Study period	Location	Province	Region	Recruitment venue	Sampling method	Number of FSW tested for HIV	Total number of FSW	Testing rate (%)	QA Score
Zhang MN, 2011 [46]	2010/06	Lin Fen	Shaanxi	Northwest	Entertainment	--	191	400	47.8%	4
Wang TM, 2009 [47]	2004	Daye	Hubei	South Central	Entertainment	--	81	367	22.1%	3
Cao XL, 2011 [48]	2005	Nanyang	Henan	South Central	Entertainment	--	2	300	0.7%	3
Cao XL, 2011 [48]	2006	Nanyang	Henan	South Central	Entertainment	--	51	300	17.0%	3
Wang TM, 2009 [47]	2006	Daye	Hubei	South Central	Entertainment	--	216	437	49.4%	3
Pan XL, 2009 [49]	2006/04-2006/06	Baise	Guangxi	South Central	Entertainment	Cluster sampling	40	253	15.8%	4
Wen XQ, 2012 [50]	2007	Gui Lin	Guangxi	South Central	Entertainment	--	25	360	6.9%	4
Cao XL, 2011 [48]	2007	Nanyang	Henan	South Central	Entertainment	--	214	361	59.3%	3
Li LN, 2009 [51]	2007	Daye, Suizhou, Xiangcheng	Hubei	South Central	Entertainment	Two-stage sampling	530	817	64.9%	5
Zhang SJ, 2008 [52]	2007	Congzuo	Guangxi	South Central	VCT	--	157	385	40.8%	4
Wang TM, 2009 [47]	2007	Daye	Hubei	South Central	Entertainment	--	172	247	69.6%	3
Liu C, 2011[53]	2007	Wu Han	Hubei	South Central	Entertainment	--	245	444	55.2%	3
Pan XL, 2009 [49]	2006/04-2006/06	Baise	Guangxi	South Central	Entertainment	Cluster sampling	109	300	36.3%	4
Wen XQ, 2009 [54]	2007/04-2007/05	Guilin	Guangxi	South Central	Entertainment	Stratified cluster and random sampling	86	360	23.9%	3

First author, published year	Study period	Location	Province	Region	Recruitment venue	Sampling method	Number of FSW tested for HIV	Total number of FSW	Testing rate (%)	QA Score
Zhou JH, 2010 [55]	2007/07-2007/09	Shenzhen	Guangdong	South Central	Entertainment	--	171	418	40.9%	4
Tan SN, 2010 [56]	2007/08-2007/11	Laibin	Guangxi	South Central	Entertainment	Stratified cluster sampling	40	246	16.3%	5
Wen XQ, 2012 [50]	2008	Gui Lin	Guangxi	South Central	Entertainment	--	85	400	21.3%	4
Cao XL, 2011 [48]	2008	Nanyang	Henan	South Central	Entertainment	--	298	376	79.3%	3
Wang TM, 2009 [47]	2008	Daye	Hubei	South Central	Entertainment	--	269	347	77.5%	3
Pan XL, 2009 [49]	2006/04-2006/06	Baise	Guangxi	South Central	Entertainment	Cluster sampling	145	311	46.6%	4
Tan JG, 2009 [57]	2008/04-2008/05	Shenzhen	Guangdong	South Central	Entertainment	Stratified cluster sampling	72	335	21.5%	5
Bai Y, 2009 [58]	2008/04-2008/07	Liuzhou	Guangxi	South Central	Entertainment	Random sampling	210	431	48.7%	6
Zhang YH, 2011 [13]	2008/04-2008/07	Haikou	Hainan	South Central	--	--	16	404	4.0%	2
Zhang YH, 2011 [13]	2008/04-2008/07	Sanya	Hainan	South Central	--	--	47	400	11.8%	2
Zhang YH, 2011 [13]	2008/04-2008/07	Guangzhou	Guangdong	South Central	--	--	52	289	18.0%	2
Zhang YH, 2011 [13]	2008/04-2008/07	Wuhan	Hubei	South Central	--	--	209	444	47.1%	2
Wen YQ, 2011 [59]	2008/04-2008/07	Liu Zhou	Guangxi	South Central	Entertainment	--	154	320	48.1%	5
Wen YQ, 2011 [59]	2008/04-2008/07	Liu Zhou	Guangxi	South Central	Entertainment	--	154	320	48.1%	5
Wen YQ, 2011 [59]	2008/04-2008/07	Liu Zhou	Guangxi	South Central	Entertainment	--	528	1047	50.4%	5

First author, published year	Study period	Location	Province	Region	Recruitment venue	Sampling method	Number of FSW tested for HIV	Total number of FSW	Testing rate (%)	QA Score
Wen YQ, 2011 [59]	2008/04-2008/07	Liu Zhou	Guangxi	South Central	Entertainment	--	370	712	52.0%	5
Wen YQ, 2011 [59]	2008/04-2008/07	Liu Zhou	Guangxi	South Central	Entertainment	--	370	712	52.0%	5
Bai Y, 2009 [58]	2008/04-2008/07	Liuzhou	Guangxi	South Central	Entertainment	Random sampling	107	167	64.1%	6
Bai Y, 2009 [58]	2008/04-2008/07	Liuzhou	Guangxi	South Central	Entertainment	Random sampling	220	449	49.0%	6
BaiY, 2010 [60]	2008/04-2008/07	Liuzhou	Guangxi	South Central	--	Continuous sampling	194	448	43.3%	5
Zhang L, 2010 [61]	2008/12	Shangcai	Henan	South Central	Entertainment	--	43	172	25.0%	3
Chen L, 2010 [62]	2009	Shenzhen	Guangdong	South Central	Entertainment	Random sampling	78	426	18.3%	2
Wen XQ, 2012 [50]	2009	Gui Lin	Guangxi	South Central	Entertainment	--	120	400	30.0%	4
Cao XL, 2011 [48]	2009	Nanyang	Henan	South Central	Entertainment	--	319	378	84.4%	3
Xiang SB, 2010 [63]	2009	Hongjiang	Hunan	South Central	Entertainment	Random sampling	304	401	75.8%	4
LING B, 2011 [64]	2009/04-2009/06	Zhou Kou	Henan	South Central	Entertainment	Community outreach	324	421	77.0%	4
Zeng XL, 2011 [65]	2009/11	Nanyang	Henan	South Central	Entertainment	--	187	457	40.9%	4
Zhou Y, 2012 [66]	2009/12	Gui Lin	Guangxi	South Central	Entertainment	--	98	300	32.7%	4
Wen XQ, 2012 [50]	2010	Gui Lin	Guangxi	South Central	Entertainment	--	177	400	44.3%	4
Weir SS, 2012 [67]	2009/10-2010/01	Liuzhou	Guangxi	South Central	--	Respondent-Driven Sampling	75	161	46.6%	6

First author, published year	Study period	Location	Province	Region	Recruitment venue	Sampling method	Number of FSW tested for HIV	Total number of FSW	Testing rate (%)	QA Score
Bai Y, 2012 [68]	2010/05-2010/07	Liu Zhou	Guangxi	South Central	--	--	1039	1846	56.3%	4
Wen XQ, 2012 [50]	2011	Gui Lin	Guangxi	South Central	Entertainment	--	155	400	38.8%	4
Tang MJ, 2012 [69]	2011	Yulin	Guangxi	South Central	Entertainment	--	450	1213	37.1%	4
Tang J, 2012 [70]	2011	Gui Lin	Guangxi	South Central	Entertainment	--	155	400	38.8%	4
Xu DL, 2012 [71]	2009/04-2011/12	Zhu Hai	Guangdong	South Central	Entertainment	--	51	154	33.1%	3
Li Y, 2012 [72]	2010-2011	Xiao Gan	Hubei	South Central	Entertainment	--	318	400	79.5%	4
Huang LH, 2006 [73]	2003	Dali	Yunnan	Southwest	Entertainment	Random sampling	52	370	14.1%	4
Cao XY, 2007 [74]	2004/12-2005/01	Xichang	Sichuan	Southwest	Entertainment	--	64	330	19.4%	5
Sun JY, 2012 [75]	2006	Jie Li	Guizhou	Southwest	Entertainment	--	218	400	54.5%	4
Tan Y, 2011 [76]	2006	Gan Zi	Sichuan	Southwest	Entertainment	--	117	406	28.8%	3
Lei JH, 2012 [77]	2006	Kai Li	Guizhou	Southwest	--	--	218	400	54.5%	2
Yang JF, 2012 [78]	2007/01-2007/07	Bao Shan	Yunnan	Southwest	Entertainment	--	629	1080	58.2%	4
Lu PN, 2008 [79]	2007/04-2007/05	Panzhihua	Sichuan	Southwest	Entertainment	Convenience sampling	23	358	6.4%	3
Peng HB, 2007 [80]	2007/04-2007/05	Nanchong	Sichuan	Southwest	Entertainment	Stratified random sampling	136	420	32.4%	5
Luo L, 2009 [81]	2007/07-2007/09	Mianyang	Sichuan	Southwest	Entertainment	Random sampling	147	411	35.8%	7
Sun JY, 2012 [75]	2008	Jie Li	Guizhou	Southwest	Entertainment	--	252	400	63.0%	4
Lei JH, 2012 [77]	2008	Kai Li	Guizhou	Southwest	--	--	252	400	63.0%	2
Zi GS, 2009 [82]	2008/01	Weishan	Yunnan	Southwest	Entertainment	--	65	85	76.5%	4

First author, published year	Study period	Location	Province	Region	Recruitment venue	Sampling method	Number of FSW tested for HIV	Total number of FSW	Testing rate (%)	QA Score
Yang JF, 2012 [78]	2008/01-2008/07	Bao Shan	Yunnan	Southwest	Entertainment	--	665	1082	61.5%	4
Dong LM, 2010 [83]	2008/04-2008/06	A District Zigong	Sichuan	Southwest	Entertainment	--	201	536	37.5%	6
Zhang YH, 2011 [13]	2008/04-2008/07	Chongqing	Chongqing	Southwest	--	--	110	429	25.6%	2
Zhang YH, 2011 [13]	2008/04-2008/07	Kunming	Yunnan	Southwest	--	--	111	405	27.4%	2
Sun JY, 2012 [75]	2009	Jie Li	Guizhou	Southwest	Entertainment	--	114	400	28.5%	4
Yan WZ, 2011 [84]	2009	Jiong Hong	Yunnan	Southwest	Entertainment	--	33	180	18.3%	3
Lei JH, 2012 [77]	2009	Kai Li	Guizhou	Southwest	--	--	114	400	28.5%	2
Yang JF, 2012 [78]	2009/01-2009/07	Bao Shan	Yunnan	Southwest	Entertainment	--	868	1200	72.3%	4
Yu XW, 2010 [85]	2009/04-2009/06	Lancang	Yunnan	Southwest	Entertainment	--	72	320	22.5%	7
Dong LM, 2010 [83]	2009/04-2009/06	B District Zigong	Sichuan	Southwest	Entertainment	--	106	335	31.6%	6
Wang QF, 2010 [86]	2009/05	Songming	Yunnan	Southwest	Entertainment	--	108	193	56.0%	3
Sun JY, 2012 [75]	2010	Jie Li	Guizhou	Southwest	Entertainment	--	186	400	46.5%	4
Tan Y, 2011 [76]	2010	Gan Zi	Sichuan	Southwest	Entertainment	--	13	400	3.3%	3
Lei JH, 2012 [77]	2010	Kai Li	Guizhou	Southwest	--	--	186	400	46.5%	2
Yang JF, 2012 [78]	2010/01-2010/07	Bao Shan	Yunnan	Southwest	Entertainment	--	878	1405	62.5%	4
Yang ZJ, 2012 [87]	2010/03-2010/06	Rui Li	Yunnan	Southwest	Entertainment	--	257	501	51.3%	4
Zhou Z, 2012 [88]	2010/04-2010/06	Da Li	Yunnan	Southwest	Entertainment	--	1084	2019	53.7%	4
Guo HJ, 2011 [89]	2010/04-2010/07	Zun Yi	Guizhou	Southwest	--	--	16	235	6.8%	4
Ci Ren WM, 2012 [90]	2010/06-2010/07	La Sa	Tibet	Southwest	Entertainment	--	57	442	12.9%	3

First author, published year	Study period	Location	Province	Region	Recruitment venue	Sampling method	Number of FSW tested for HIV	Total number of FSW	Testing rate (%)	QA Score
Li Y, 2012 [91]	2010-2011	Lan Cang	Yunnan	Southwest	Entertainment	--	195	701	27.8%	3
Yang JF, 2012 [78]	2011/01-2011/07	Bao Shan	Yunnan	Southwest	Entertainment	--	1071	1405	76.2%	4
Li YK, 2011 [92]	2011/05-2011/09	8 Counties	Sichuan	Southwest	--	--	89	368	24.2%	3

References

1. Chu Q, Zhang X, Fa P, Jiang Z, Wu W, Hao B, et al. [Investigation of demographic and behavioral changes related to AIDS among urban female sex workers (FSW)]. Chinese Journal of AIDS & STD. 2005;11(1):23-6.
2. Zhu X, Kang D, Liu X, Liao M, Fu J. [Analysis of behavioral changes among clandestine prostitutes in Jiaozhou city]. Chinese Journal of AIDS & STD. 2008;14(1):28-30.
3. Liao M, Bi Z, Liu X, Kang D, Fu J, Song Q, et al. Condom use, intervention service utilization and HIV knowledge among female sex workers in China: results of three consecutive cross-sectional surveys in Shandong Province with historically low HIV prevalence. International journal of STD & AIDS. 2012;23(3):e23-9.
4. Wu J, Wang W, Wang F. [The effect of behavior intervention among the commercial sec workers in entertainment places of Yingtan City]. Journal of Preventive Medicine Information. 2006;22(6):647—9.
5. Dou ZD, He JG, Jin YL, Fang YJ, An Z. [Study of effects of HIV intervention in female sex workers in Wuhui]. Anhui Journal of Preventive Medicine. 2012;18(5):326-8+31.
6. Wang XM, Liu XH. [Study on the HIV detection and related behavior among FSWs]. Zhejiang Journal of Preventive Medicine. 2011;23(7):37-8+42.
7. Guo Z, Wan D, Feng C, Cai L, Fan Y. [Trend analysis on knowledge and behavior of AIDS prevention among the CSWs in Mengcheng County]. Chinese Journal of Disease Control & Prevention. 2009;13(04):411-3.
8. Luo Y, Chen S, Xu K, Yuan H, Chen J, Hu J, et al. [Survey of STD/AIDS-related knowledge, behaviors and infection rates of sex workers in entertainment places in Hangzhou]. Disease Surveillance. 2008;23(10):607-9.
9. Liu Y, He J, Deng W. [HIV transmission and behavioural characteristics among female sex workers in Jizhou district, Jilin City, 2007]. Journal of Jinggangshan Medical College. 2007;14(06):41-2.
10. Tang Q, Fang W, Wang H, Bo D, Jiang J. [Effect of AIDS comprehensive intervention among the commercial sex workers (CSW) in Haishu District, Ningbo City]. Chinese Rural Health Service Administration. 2009;29(11):855-7.
11. Chen L. [Analysis on the surveillance in observation sites of AIDS and venereal diseases in year 2008 Cong Ming county Shanghai municipality]. Shanghai Journal of Preventive Medicine. 2009;21(2):83.
12. Zhang XJ, Liao MZ, Kang DM, Tao XR, Qian YS, Wang GR, et al. [Condom Use and Correlates Among Female Sex Workers in Shandong Province,2006-2008]. Preventive Medicine Tribune. 2012;18(6):405-7+10.
13. Zhang YH, Bao YG, Li CM, Han L, Sun JP, Tan HZ. [Study of HIV/ syphilis infection status of commercial sex workers in 15 cities of China]. China Preventive Medicine. 2011;12(5):387-90.
14. Hu CC, Yuan ZK, Liu Y. [HIV/AIDS Intervention Among Female Sex Workers in Different Places in Nanchang City]. Journal of Nanchang University(Medical Science). 2011;51(2):74-7.
15. Kang D, Liao M, Jiang Z, Zhang X, Mao W, Zhang N, et al. Commercial sex venues, syphilis and methamphetamine use among female sex workers. AIDS Care. 2011;23 Suppl 1:26-36.

16. Xi S, He Y, Zhou X, Zhou D, Wang C. [A Survey on the status and wishes of HIV voluntary counseling and testing and its influencing factors among community female commercial sex workers]. Zhejiang Journal of Preventive Medicine. 2011;23(1):8-10, 6.
17. Xi SJ, He YF, Zhou XH, Zhou DD, Wang CC. [A Survey on the Status and Wishes of HIV Voluntary Counseling and Testing and Its Influencing Factors among Community Female Commercial Sex Workers]. Zhejiang Journal of Preventive Medicine. 2011;23(1):8-10+6.
18. Chen GS, Wu HS, Yao ZM. [Investigation on HIV/AIDS related knowledge, behavior and infection among commercial female sex workers in one county]. Anhui Journal of Preventive Medicine. 2010;16(5):418-9.
19. Chen SX, Zhang ML, Han XM. [Survey on AIDS Related Knowledge, Behavior and the HIV Infection Status Among Commercial Sex Workers in Gaomi City]. Preventive Medicine Tribune 2011;17(12):1119-20, 23.
20. Zhang H, Chen CG, Lin FH, Xu SY, Yao X, Lin H, et al. [Analysis of Comprehensive Surveillance Results of HIV /AIDS-related High- risk Groups in Fuzhou City in 2010]. Occupation and Health. 2011;27(21):2406-9.
21. Sun XQ, Tang GX, Mao TS, Zhang YJ, Mao N. [2010 baseline survey among commercial sex workers of Taihe County]. China Modern Medicine. 2011;18(28):151-3.
22. Qian ZH, Wang J, Fan XQ. [HIV/AIDS sentinel surveillance results among prostitutes in Suzhou city]. Jiangsu Journal of Preventive Medicine. 2012;23(1):30-1.
23. Kang DM, Tao XR, Li JZ, Liao WZ, Zhu XH, Zhang H, et al. [Evaluation of AIDS intervention among female sex workers in Global Fund Project counties in Shandong Province]. Journal of Shandong University (Health Sciences). 2011;49(10):155-9.
24. Wu SB, Chen G, Pan WJ, Lin YT, Zheng WX, Lin L, et al. [Survey on AIDS-related Knowledge and Behavior among Commercial Sex Workers in Fujian Province]. Chinese Journal of Social Medicine. 2012;29(4):264-5.
25. Jiang J, Wang HB, Fang WM, Sun JL, Chen BB, Bo DY, et al. [Survey of AIDS prevalence in female sex workers detained in a correctional facility in Ningbo, Zhejiang]. Disease Surveillance. 2012;27(08):634-6.
26. Sun BJ. [Survey on AIDS-related Knowledge, Behavior and HIV-infection Among Commercial Sex Workers in Shizhong District, Zaozhuang City, 2011]. Preventive Medicine Tribune. 2012;18(9):654-6.
27. Gan WH, Zhu JM, Jiang CH, Huang RR, Chen L. [Analysis of AIDS sentinel surveillance among the female sex workers in Jinshan district of Shanghai from 2009-2011]. Chinese Journal of AIDS & STD. 2012;18(7):457-8+74.
28. Xu JS, Liu XY, Fu GF, Huan XP, Li L, Xu XQ, et al. [Syphilis and HIV Infection Status Among Female Sex Workers in Jiangsu Province]. The Chinese Journal of Dermatovenereology. 2012;26(6):513-5.
29. Qiu ZH, Dong ZQ, Jin MH, Yang ZR. [Sentinel surveillance of AIDS among female sex workers in Huzhou, Zhejiang, 2011]. Disease Surveillance. 2012;27(4):291-3.
30. Zhu HW, Huang SP, Zhu RH, Zhan MW, Yan W. [Analysis of AIDS Sentinel Surveillance in Nanchang County in 2011]. Chinese Community Doctors. 2012;14(32):338.
31. Chen G, Wu ZH, He X, Wang W. [Effect evaluation of the AIDS health education for CSW in 3 cities]. Chinese Journal of Health Education. 2012;v.28(08):682-4+7.
32. Bao F, Gao P, Yun Z, Ren X, Liu X, Guo S. [An AIDS epidemiological survey of female sex workers crowd in Hohhot]. Inner Mongolia Medical Journal. 2007;39(08):971-3.
33. AO X, Han Q. [Survey of AIDS-related knowledge, behaviors and infection HIV and syphilis among 105 sex workers]. Disease Surveillance. 2008;23(11):714-6.

34. Li M, Li M, Yu JP, Min J, Zhang XJ, Han Q. [Investigation and analysis on AIDS-related knowledge and behaviors among female sex workers in Xicheng district of Beijing from 2009-2011]. Occupation and Health. 2012;28(22):2792-3, 5.
35. Jia J, Gao LQ, Xing LY, Han YD, Liu M, Lei XY, et al. [Investigation on HIV-related knowledge and praxeology among commercial sex workers in Beilin District, Xi'an City]. Chinese Journal of Misdiagnostics. 2011;11(32):7940-1.
36. Li BY, Zhao XH, Wang GR. [Recognition of AIDS among unlicensed prostitutes in Jinnan district of Tianjin]. Occupation and Health. 2012;28(4):465-6.
37. Qi G, Zhang X, Li X, Sun J, Wang Q, Li S. [Investigation on HIV/AIDS-related knowledge and behaviors of commercial sex workers in Changchun entertainment establishments]. Chinese primary health care. 2008;22(12):66-8.
38. Li Y, Wang KL, Tong X, Yan HM. [Analysis of sentinel monitoring of AIDS among high risk population in Heilongjiang Province in 2010]. Chinese Primary Health Care. 2011;25(5):59-61.
39. Xian XJ. [Analysis of sentinel monitoring on HIV/AIDS in Nongan County of Jilin Province]. Blooming Season. 2011;462(22):261.
40. Wang X, Jiang A, Xu X, Zhang Y, Li G. [AIDS risk behaviours, knowledge and healthcare seeking behaviours among female sex workers]. Journal of Medical Pest Control. 2005;21(5):345-7.
41. MAYT A. [HIV Cross-section Study on Female Sex Workers in Xinshi District, Urumqi in 2007]. Endemic Diseases Bulletin. 2008;23(4):27-8.
42. Wang M, Chen Y, Mao Z, Xue W, Liu J, Zhang K. [A study of HIV knowledge, behaviours and needs among female sex workers in Shanyang county, Sha'anxi province]. Chinese Journal of AIDS & STD. 2008;14(6):626.
43. Chang WH, Xing AH, Wang BS, Li X, Jia H, Zhang L, et al. [Analysis of HIV /AIDS sentinel surveillance among high risk population in Shanxi in 2010]. Occupation and Health. 2012;28(4):399-402.
44. Wu R, Xue SF, Yao XW. [An investigation of HIV/AIDS-related risk information among 412 female sex workers]. Chinese Journal of AIDS & STD. 2012;18(11):793, 6.
45. Zhao GD, Zhong L, Li YY. [Analysis of AIDS sentinel surveillance among illicit prostitutes in Shangluo City in 2010]. Journal of Hebei United University(Health Sciences). 2011;13(4):469-70.
46. Zhang MN, Zhang ZH, Huang L, Wang XM. [An investigation of sexually transmitted infection and related behavioral feature on unlicensed prostitutes in Shanxi province]. Chinese Remedies & Clinics. 2011;11(9):1051-2.
47. Wang T. Effectiveness analysis of behavioural interventions among entertainment-based female sex workers in Dazhi prefecture in 2004-2008]. Journal of Public Health and Preventive Medicine. 2009;20(109):115-6.
48. Ceng XL. [Effect Evaluation of Propaganda Intervention of AIDS in Female Sex Workers in Wolong District of Nangyang City,2005-2009]. Preventive Medicine Tribune. 2011;17(2):97-8+101.
49. Pan X. [The investigation of Venereal disease for 864 female sex workers]. Youjiang Medical Journal. 2009;37(01):10-3.
50. Wen XQ. [Effects of HIV-related health education and intervention to FSWs in Guilin City from 2007 - 2011]. Journal of Public Health and Preventive Medicine. 2012;23(6):125-7.
51. Li L, Yang H, Liu S, Zhou J, Ma Y, Yin P. [Evaluation of the effect of spreading HIV/ AIDS knowledge among commercial sex workers in three cities in Hubei]. Chinese Journal of Disease Control & Prevention. 2009;13(4):408-10.

52. Zhang SJ. [Characteristics and trend of HIV/AIDS epidemic in Chongzuo city of Guangxi, 1996-2007]. Internal Medicine of China. 2008;3(06):932-5.
53. Liu C, Xu J, Zhou W, Yang DL, Yao ZZ, Wang X, et al. [Baseline investigation on three kinds of high risk population in Wuhan, China Bill & Melinda Gates Foundation AIDS program]. Journal of Public Health and Preventive Medicine. 2011;22(2):15-8.
54. Wen X. [Survey on KAB and serology of AIDS among 360 female commercial sex workers in Guilin]. Modern Preventive Medicine. 2009;36(14):2687-9.
55. Zhou J, Huang Z, Deng B, Chen Y, Luo R. [Survey on the behavioral characteristics and sexually transmitted infection among 418 female commercial sex workers]. Modern Preventive Medicine. 2010;37(6):1158-9, 61.
56. Tan S, Lan R, Wei J, Xie S, Qin Q, Lin F. [A study of STD/HIV infection status among female sex workers in Laibin City, 2007]. Applied Prev Med. 2010;16(02):98-9.
57. Tan J, Chen L, Cai W, Yang Z, Shi X, Wang X. [Study on AIDS/STD related risk behaviors among female sex workers in Shenzhen]. Chinese journal of Social Medicine. 2009;v.26(04):242-4.
58. Ba iY. [Investigation on AIDS related knowledge, behaviors and infection among commercial female sex workers in Liuzhou city in 2008]. Preventive Medicine Tribune. 2009;15(12):1224-6.
59. Weng YQ, Bai Y, Feng WD. [High risk behavior toward HIV/AIDS of female sexual service workers with different marital status in Liuzhou City]. China Tropical Medicine. 2011;11(2):170-1.
60. Bai Y, Weng YQ, Feng WD. [Sentinel Surveillance of AIDS in Liuzhou, 2008]. Journal of Preventive Medicine Information. 2010;26(7):527-30.
61. Zhang L, Xue FH, Zhang XQ, Dong SB. [Study on HIV/AIDS related knowledge and behavior among female sex workers in Xincai County of Henan Province]. Henan Journal of Preventive Medicine. 2010;21(3):235-7.
62. Chen L, Tan J, Shi X, Gan Y, Zhang Y, Zhao J, et al. [The comprehensive surveillance of AIDS among unlicensed female sex workers in Shenzhen city]. Journal of Tropical Medicine. 2010;10(6):748-9.
63. Xiang S, Peng J, Tang J, Hu C. [HIV knowledge, attitude and behavioural survey among female sex workers in Hongjiang city]. Practical Preventive Medicine. 2010;17(3):596-7.
64. Leng B, Zhang L, Zi XM, Xu K. [Study on the HIV-related knowledge and behaviours of illicit prostitutes in Zhoukou City]. Henan Journal of preventive Medicine. 2011;22(5):367-8.
65. Ceng XL, Li P, Ma CH. [The effects of sexually transmitted infections AIDS behavioral intervention among female sex workers in project region]. Henan Journal of preventive Medicine. 2011;22(2):92-4.
66. Zhang C, Li X, Hong Y, Chen Y, Liu W, Zhou Y. Partner violence and HIV risk among female sex workers in China. AIDS Behav. 2012;16(4):1020-30.
67. Weir SS, Merli MG, Li J, Gandhi AD, Neely WW, Edwards JK, et al. A comparison of respondent-driven and venue-based sampling of female sex workers in Liuzhou, China. Sex Transm Infect. 2012;88 Suppl 2:i95-101.
68. Bai Y, Zhang JP, Ouyang Y. [Analysis on monitoring results of AIDS among female sexual workers in Liuzhou City in 2010]. Chinese Journal of Pest Control. 2012;28(9):964-6.
69. Tang MJ, Zhong FH, Liu JJ, Zhang DL, Tan XZ. [Analysis of AIDS Sentinel Surveillance in Yulin city in 2011]. Journal of Applied Preventive Medicine. 2012;18(1):40-2.

70. Tang J, Zhang ZK, Zhou Y, Wen XQ, Zhou HJ. [Analysis of sentinel monitoring of AIDS among high risk population in Guilin,2011]. Chinese Journal of AIDS & STD. 2012;18(8):533-6.
71. Xu DL, Zhou A, Huang HW, Li X, Wang Q, He HF. [The Assessment of Medical Needs and Characteristics among 154 Female Sex Workers]. Journal of Community Medicine. 2012;10(11):53-4.
72. Li Y, Wu D, Wang SY. [Analysis of sentinel surveillance on FSWs in Xiaonan district of Hubei province in 2011]. Today Nurse. 2012;(4):132-3.
73. Huang LH, Liu YZ, Chen ZJ, Lu MJ, Xu XR, Zhang XZ, et al. [Comprehensive HIV/AIDS intervention for female sex workers - effectively curb the spread of STD and AIDS]. Soft Science of Health. 2006;20(3):271-3.
74. Cao X, Jiang Z, Ruan Y, Liang S, Qin G, Yang Y, et al. [Analysis on the factors of unprotected sex behavior among female sex workers]. Chinese Journal of Health Education. 2007;23(4):277-9.
75. Sun JY, Xiao YL, Huang GX, Lei JH. [Monitoring on AIDS behavior among prostitutes in Kaili from 2006 to 2010]. Modern Preventive Medicine. 2012;39(14):3593-8+600.
76. Tan Y, Feng L. [STDs /AIDS Sentinel Surveillance of FSW in Ganzi Prefecture, 2006 and 2010]. Journal of Preventive Medicine Information. 2011;27(9):670-3.
77. Lei JH, Xiao YL, Sun JY. [Analysis of the changing trend of AIDS high-risk behaviors among CSWs in Kaili city]. Chinese Journal of AIDS & STD. 2012;18(2):124-6.
78. Yang JF, Hu AY, Zhao CZ, Guo JH, Peng JY, Liu L, et al. [Data analysis of HIV prevalence and related behaviors among female sex workers in Baoshan of Yunnan Province from 2007 to 2011]. Chinese Journal of AIDS & STD. 2012;18(7):454-6.
79. Lu P, Li C, Tang Z, Tan Q, Li D. [HIV/AIDS behavioral survey among female sex workers in countryside area of Panzhihua city]. Journal of Preventive Medicine Information. 2008;24(8):608-10.
80. Peng H, Fu G, Feng Y, Tian X, Feng J, Zhang Q, et al. [A Survey on CSW's AIDS knowledge, behavior and intervention in an urban district in Nanchong]. Journal of North Sichuan Medical College. 2007;22(05):428-31.
81. Luo JF. [Analysis of HIV/AIDS surveillance in Wuyishan City in Fujian Province, 2002-2006]. Chinese Journal of Ethnomedicine and Ethnopharmacy. 2009;18(13):71-2.
82. Zi G, Yao H, Cha X, Guo J, Zi Z. [Investigation on the Status Related to AIDS among Female Sex Workers in Weishan County in 2008]. Preventive Medicine Tribune. 2009;15(11):1092-3.
83. Dong L, Li Q, Chen X, Zhou M, Xie Y. [Surveillance of behavior among female sex workers in Zigong, 2008-2009]. Journal of Preventive Medicine Information. 2010;26(12):982-5.
84. Yan WZ, Zheng KQ, Feng DL, Cai XY, Liu JR, Long Y, et al. [Investigation on status of STDs and AIDS among female sex workers in JingHong County of Yuinan in 2009]. Journal of Dermatology and Venereology. 2011;33(1):51+4.
85. Yu K, Wang C, Qiu G, Li X, Wang L, Gao L, et al. [Results from Sentinel Surveillance of HIV/AIDS among Commercial Sex Workers in Lancang County in 2009]. Practical Preventive Medicine. 2010;17(11):2303-5.
86. Wang QF. [Analysis on HIV/AIDS knowledge and behaviour among prostitutes in Songming County, Yunnan Province]. Medical Information. 2010;23(4):928-9.
87. Yang ZJ, Yin ZL, Li ZL, Liu B, Fang KF, Li RC, et al. [Analysis of AIDS Sentinel Surveillance in Ruili City in 2010]. Soft Science of Health. 2012;26(9):785-7.

88. Zhou Z, Huang LH, Chen ZJ, Lu MJ. [Analysis of surveillance monitoring of illicit prostitutes in Dalian City in 2010]. Soft Science of Health. 2012;26(5):449-51.
89. Guo HJ, Feng D, Chen ZY, Zhou CX, Sun X, Chen ZM. [AIDS Knowledge Levels and Behavior Characteristics of FSWs in Places of Entertainment in Zunyi City]. Occupation and Health. 2011;27(22):2603-4.
90. Wangmo T, Zhou YL, Zhang XH, Liang SF, Zhang S. [Analysis of results of sentinel monitoring on FSWs in Lhasa City]. Jiangsu Journal of Preventive Medicine. 2012;23(6):41-2.
91. Yang Y, Li HL, Yang YH. [Surveillance of HIV/AIDS among FSWs in Lahuzu county, Yuinan]. Soft Science of Health. 2012;26(10):911-2.
92. Nie ZQ, Lin P, Li Y, Wang Y. [Surveillance of AIDS high-risk people in Guangdong province,2009]. Journal of Tropical Medicine. 2011;11(1):29-31, 45.
