# Supplementary material for: Risk Behaviours among Female Sex Workers in China: A Systematic Review and Data Synthesis
Source: PLoS One. 2015 Mar 27;10(3):e0120595. doi: 10.1371/journal.pone.0120595 (PMC4376708; doi:10.1371/journal.pone.0120595)
Supplement: S6 Table — (PDF) [file pone.0120595.s008.pdf]

Table S6. Studies reported the drug use behaviours in female sex workers.

First author, published year	Study period	Location	Province	Region	Recuitment venue	Sampling method	Number of FSW used drug	Total number of FSW	Drug usage (%)	QA Score
Wang BF, 2004 [1]	2002	Longyan	Fujian	East	Detention Center	--	1	267	0.4%	5
Xiao Y, 2002 [2]	2002	Jiujiang	Jiangxi	East	Entertainment	Convenience sampling	38	201	18.9%	5
Xie HY, 2004 [3]	2000-2003	Quanzhou	Fujian	East	Detention Center	--	18	1996	0.9%	7
Yang JZ, 2004 [4]	2003	-	Zhejiang	East	VCT	Time-location based sampling	3	279	1.1%	6
Liu XZ, 2006 [5]	2004	-	Shandong	East	VCT	--	35	3513	1.0%	4
Shen YG, 2006 [6]	2004/10	Jiaxing	Zhejiang	East	Entertainment	Random sampling	0	126	0.0%	3
Yang JZ, 2005 [7]	2004	Hangzhou	Zhejiang	East	Entertainment	Two stage cluster sampling	14	314	4.5%	5
Zhu XY, 2008 [8]	2004/09-2004/10	Jiaozhou	Shandong	East	Entertainment	--	15	360	4.2%	4
Zou Y, 2005 [9]	2004/04-2004/05	Ningbo, Wenzhou, Shaoxing	Zhejiang	East	Detention Center	Continuous sampling	5	452	1.1%	7
Zou Y, 2005 [9]	2004/10-2004/11	Ningbo, Wenzhou, Shaoxing	Zhejiang	East	Detention Center	Continuous sampling	7	842	0.8%	7
Cheng XI, 2009 [10]	2005	-	Anhui	East	VCT	--	129	393	32.8%	4
Li JX, 2007 [11]	2003-2005	Jinan	Shandong	East	Entertainment	--	18	1540	1.2%	7
Cheng XL, 2009 [10]	2006	-	Anhui	East	VCT	--	115	401	28.7%	4
Liao M, 2012 [12]	2006	Dezhu, Yantai, Qingdao	Shandong	East	Entertainment	Venue-based sampling	139	1104	12.6%	5

First author, published year	Study period	Location	Province	Region	Recuitment venue	Sampling method	Number of FSW used drug	Total number of FSW	Drug usage (%)	QA Score
Luo Y, 2008 [13]	2006/04-2006/05	Hangzhou	Zhejiang	East	Detention Center	Continuous sampling	3	250	1.2%	6
Luo Z, 2007 [14]	2006	Shanghai	Shanghai	East	Entertainment	--	8	373	2.1%	4
Sun ZX, 2008 [15]	2006/06	Wenzhou	Zhejiang	East	Detention Center	--	0	141	0.0%	5
Chen SP, 2010 [16]	2007/03-2007/04	Xunyang District	Jiangxi	East	Entertainment	Convenience sampling	27	360	7.5%	7
Chen YL, 2009 [17]	2001-2007	Longyan	Fujian	East	Detention Center	--	5	1859	0.3%	5
Cheng XI, 2009 [10]	2007		Anhui	East	VCT	--	3	312	1.0%	4
Liao M, 2012 [12]	2007	Dezhu, Yantai, Qingdao	Shandong	East	Entertainment	Venue-based sampling	98	1197	8.2%	5
Liao MZ, 2008 [18]	2007		Shandong	East	VCT	--	98	1197	8.2%	4
Luo Y, 2008 [19]	2007/02-2007/07	Hangzhou	Zhejiang	East	Entertainment	--	1	257	0.4%	5
Ni YQ, 2008 [20]	2007/07-2007/11	Shanghai	Shanghai	East	Entertainment	--	0	404	0.0%	6
Peng B, 2008 [21]	2007/04-2007/06	Jiujiang	Jiangxi	East	Entertainment	--	14	250	5.6%	3
Qi CM, 2009 [22]	2007/07-2007/10	Zhaoyuan	Shandong	East	Entertainment	--	15	197	7.6%	3
Wang WM, 2008 [23]	2007/01-2007/12	Kunshan	Jiangsu	East	Detention Center	--	2	297	0.7%	4
ChenL, 2009 [24]	2008	Shanghai	Shanghai	East	Entertainment	Continuous sampling	7	455	1.5%	5
Liao M, 2011 [25]	2006-2008	Qingdao	Shandong	East	Entertainment	Convenience sampling	366	1211	30.2%	4
Liao M, 2012 [26]	2008/02-2008/08	Jinan	Shandong	East	Entertainment	Respondent-driven sampling	46	363	12.7%	6

First author, published year	Study period	Location	Province	Region	Recuitment venue	Sampling method	Number of FSW used drug	Total number of FSW	Drug usage (%)	QA Score
Liao M, 2012 [12]	2008	Dezhu, Yantai, Qingdao	Shandong	East	Entertainment	Venue-based sampling	152	1159	13.1%	5
Tang X, 2010 [27]	2008/10-2008/11	Shanghai	Shanghai	East	Entertainment	--	1	285	0.4%	6
Wang FH, 2009 [28]	2008		Anhui	East	Entertainment	--	0	4584	0.0%	2
Xue FH, 2009 [29]	2008/04-2008/06	Wenzhou	Zhejiang	East	Entertainment	Random sampling	3	403	0.7%	7
Yang Y, 2011 [30]	2008/06-2008/10	Shanghai	Shanghai	East	Entertainment	--	5	411	1.2%	5
Zhang XJ, 2012 [31]	2006-2008	Qingdao, Yantai,Dezhou	Shandong	East	Entertainment	--	389	3460	11.2%	4
Kang D, 2011 [32]	2006-2009	Qingdao	Shandong	East	Entertainment	Venue-based, community outreach and peer-referral	19	120	15.8%	6
Kang D, 2011 [32]	2006-2009	Qingdao	Shandong	East	Entertainment	Venue-based, community outreach and peer-referral	272	823	33.0%	6
Kang D, 2011 [32]	2006-2009	Qingdao	Shandong	East	Entertainment	Venue-based, community outreach and peer-referral	69	244	28.3%	6
Liao M, 2012 [26]	2009/05-2009/10	Jinan	Shandong	East	Entertainment	Respondent-driven sampling	60	432	13.9%	6
Liao MZ, 2010 [33]	2009		Shandong	East	Mixed venues	--	169	4732	3.6%	4
Wan LJ, 2011 [34]	2008-2009	Shang Yu	Zhejiang	East	Entertainment	Two stage cluster sampling	6	288	2.1%	4

First author, published year	Study period	Location	Province	Region	Recuitment venue	Sampling method	Number of FSW used drug	Total number of FSW	Drug usage (%)	QA Score
Wang F, 2010 [35]	2009	Bengbu	Anhui	East	Entertainment	--	0	75	0.0%	4
Xi SJ, 2010 [36]	2009	Hangzhou	Zhejiang	East	Entertainment	--	9	210	4.3%	5
Xi SJ, 2011 [37]	2009/04-2009/06	Hangzhou	Zhejiang	East	Entertainment	--	18	446	4.0%	4
XI SJ, 2011 [38]	2009/04-2009/06	Hangzhou	Zhejiang	East	Entertainment	Cluster sampling	18	446	4.0%	5
Yang Y, 2011 [30]	2009/05-2009/08	Shanghai	Shanghai	East	Entertainment	--	13	411	3.2%	5
Zhang QQ, 2012 [39]	2009/07-2009/09	Yang Zhou, Chang Zhou	Jiangsu	East	Entertainment	--	4	1069	0.4%	4
Zhang QQ, 2012 [39]	2009/10-2009/12	Yang Zhou,Chang Zhou	Jiangsu	East	Entertainment	--	2	737	0.3%	4
Chen CG, 2011 [40]	2009/11-2010/01	Fu Zhou	Fujian	East	Entertainment	--	9	225	4.0%	2
Jiang J, 2012 [41]	2010/07	Ning Bo	Zhejiang	East	Detention Center	--	23	416	5.5%	4
Miao XL, 2011 [42]	2010	Wu Xi	Jiangsu	East	Entertainment	--	25	800	3.1%	4
Qian ZH, 2012 [43]	2009-2010	Suzhou	Jiangsu	East	Detention Center	--	8	578	1.4%	4
Sun XQ, 2011 [44]	2010	Tai He	Anhui	East	Entertainment	--	0	354	0.0%	4
Tang ZL, 2011 [45]	2010/03-2010/07	Qing Dao	Shandong	East	Entertainment	--	10	106	9.4%	4
Tang ZL, 2011 [45]	2010/03-2010/07	Qing Dao	Shandong	East	Entertainment	--	5	216	2.3%	4
Sun BJ, 2012 [46]	2011	Zao Zhuang	Shandong	East	Entertainment	--	2	372	0.5%	4
Yang YH, 2012 [47]	2011	Quan Zhou	Fujian	East	Sentinel sites	Cluster sampling	16	868	1.8%	4
Ye ZM, 2012 [48]	2011/04-2011/07	Wenzhou	Zhejiang	East	Entertainment	Convenience sampling	8	830	1.0%	3

First author, published year	Study period	Location	Province	Region	Recuitment venue	Sampling method	Number of FSW used drug	Total number of FSW	Drug usage (%)	QA Score
Lin Z, 2007 [49]	2005/08-2005/09	Tongliao	Inner Mongolia	North	Entertainment	Convenience sampling	0	364	0.0%	5
Liu YJ, 2006 [50]	2005/01-2005/12	Bejing	Beijing	North	Detention Center	--	2	403	0.5%	5
Shi WY, 2007 [51]	2005/04-2005/05	Beijing	Beijing	North	Entertainment	--	6	114	5.3%	3
Bai JM, 2007 [52]	2006/10-2006/12	Beijing	Beijing	North	Entertainment	Random sampling	3	270	1.1%	4
Liu LR, 2007 [53]	2006/05-2006/10	Beijing	Beijing	North	Entertainment	Venue-based sampling	10	341	2.9%	7
Shi WY, 2012 [54]	2006	Feng Tai	Beijing	North	Entertainment	--	3	198	1.5%	4
Shi WY, 2012 [54]	2006	Feng Tai	Beijing	North	Entertainment	--	2	267	0.7%	4
Shi WY, 2012 [54]	2006	Feng Tai	Beijing	North	Entertainment	--	3	198	1.5%	4
Shi WY, 2012 [54]	2006	Feng Tai	Beijing	North	Entertainment	--	2	267	0.7%	4
Dong XY, 2009 [55]	2007/08-2007/10	Tianjin	Tianjin	North	Detention Center	--	2	178	1.1%	5
Shi WY, 2012 [54]	2007	Feng Tai	Beijing	North	Entertainment	--	5	202	2.5%	4
Shi WY, 2012 [54]	2007	Feng Tai	Beijing	North	Entertainment	--	4	452	0.9%	4
Shi WY, 2012 [54]	2007	Feng Tai	Beijing	North	Entertainment	--	5	202	2.5%	4
Shi WY, 2012 [54]	2007	Feng Tai	Beijing	North	Entertainment	--	4	452	0.9%	4
Shi WY, 2012 [54]	2008	Feng Tai	Beijing	North	Entertainment	--	11	208	5.3%	4
Shi WY, 2012 [54]	2008	Feng Tai	Beijing	North	Entertainment	--	0	236	0.0%	4
Shi WY, 2012 [54]	2008	Feng Tai	Beijing	North	Entertainment	--	11	208	5.3%	4
Shi WY, 2012 [54]	2008	Feng Tai	Beijing	North	Entertainment	--	0	236	0.0%	4
Jia J, 2011 [56]	2009	Xian	Shanxi	North	Entertainment	--	7	296	2.4%	3
Shi WY, 2012 [54]	2009	Feng Tai	Beijing	North	Entertainment	--	3	202	1.5%	4
Shi WY, 2012 [54]	2009	Feng Tai	Beijing	North	Entertainment	--	3	292	1.0%	4
Shi WY, 2012 [54]	2009	Feng Tai	Beijing	North	Entertainment	--	3	202	1.5%	4
Shi WY, 2012 [54]	2009	Feng Tai	Beijing	North	Entertainment	--	3	292	1.0%	4

First author, published year	Study period	Location	Province	Region	Recuitment venue	Sampling method	Number of FSW used drug	Total number of FSW	Drug usage (%)	QA Score
Cao H, 2010 [57]	2009/07-2010/06	Tianjin	Tianjin	North	Entertainment	Random sampling	1	186	0.5%	5
Li BY, 2012 [58]	2010/04-2010/07	Jin Nan	Tianjin	North	--	--	0	407	0.0%	4
Liu HX, 2011 [59]	2005-2010	Chang Ping	Beijing	North	--	--	23	547	4.2%	3
Li F, 2012 [12]	2011	Chang Ping	Beijing	North	Detention Center	--	4	290	1.4%	4
Liu ZJ, 2012 [60]	2011/04-2011/06	Luan Nan	Hebei	North	Entertainment	--	4	400	1.0%	4
Cui YZ, 2011 [61]	2006	Haerbin	Heilongjiang	Northeast	Entertainment	--	4	417	1.0%	4
Cui YZ, 2011 [61]	2007	Haerbin	Heilongjiang	Northeast	Entertainment	--	3	447	0.7%	4
Li Y, 2008 [62]	2007/04	Huanggu	Liaoning	Northeast	Entertainment	--	0	160	0.0%	4
Cui YZ, 2011 [61]	2008	Haerbin	Heilongjiang	Northeast	Entertainment	--	14	410	3.4%	4
Shao B, 2011 [63]	2008	13 Cities	Heilongjiang	Northeast	Entertainment	--	8	5055	0.2%	4
Cui YZ, 2011 [61]	2009	Haerbin	Heilongjiang	Northeast	Entertainment	--	4	447	0.9%	4
Li Y, 2011 [64]	2010		Heilongjiang	Northeast	Sentinel sites	--	93	7662	1.2%	4
Xian XJ, 2011 [65]	2011	Nong An	Jilin	Northeast	Sentinel sites	Cluster sampling	1	400	0.3%	3
Ni MJ, 2005 [66]	2003/12-2004/02	Kashi	Xinjiang	Northwest	Entertainment	Probability proportional to size sampling	12	300	4.0%	7
Liu YX, 2007 [67]	2006/08-2006/11	Yinchuan	Ningxia	Northwest	Entertainment	--	2	415	0.5%	4
Song Y, 2006 [68]	2006	Tulufan	Xinjiang	Northwest	Entertainment	Cluster random sampling	0	103	0.0%	4
Zeng KF, 2008 [69]	2006/01-2006/02	Kelamayi	Xinjiang	Northwest	Entertainment	--	3	755	0.4%	3
Chang WH, 2011 [70]	2010		Shaanxi	Northwest	Sentinel sites	Cluster sampling	34	4439	0.8%	4

First author, published year	Study period	Location	Province	Region	Recuitment venue	Sampling method	Number of FSW used drug	Total number of FSW	Drug usage (%)	QA Score
Hu XQ, 2011 [71]	2010	Baoji	Gansu	Northwest	--	--	1	400	0.3%	4
Zhang MN, 2011 [72]	2010/06	Lin Fen	Shaanxi	Northwest	Entertainment	--	1	400	0.3%	4
Zhao GD, 2011 [73]	2010/04-2010/06	Shang Luo	Shaanxi	Northwest	Sentinel sites	Cluster sampling	10	412	2.4%	4
Guo H, 2012 [74]	2010-2011	Long Nan	Gansu	Northwest	Entertainment	--	4	820	0.5%	3
Qu S, 2002 [75]	2000/10-2000/12	Baise City	Guangxi	South Central	Entertainment	--	36	482	7.5%	4
Ding Y, 2005 [76]	2000/10-2001/01	Zhengzhou	Henan	South Central	Entertainment	Snowball sampling	14	621	2.3%	4
He QY, 2002 [77]	2001/07		Hainan	South Central	Mixed venues	--	69	373	18.5%	3
Pei DN, 2002 [78]	2000/09-2001/08	Hainan	Hainan	South Central	Detention Center	--	47	317	14.8%	5
Zhou YJ, 2005 [79]	2001/06	Beihai	Guangxi	South Central	Entertainment	--	4	207	1.9%	3
Wang Y, 2004 [80]	2002/05-2002/06		Guangdong	South Central	Mixed venues	--	7	121	5.8%	4
Zhao XX, 2004 [81]	2002		Guangdong	South Central	VCT	--	395	1562	25.3%	4
Li N, 2007 [82]	2003	Henan	Henan	South Central	Sentinel sites	Continuous sampling	9	957	0.9%	3
Peng SQ, 2004 [83]	2003/09-2003/12	Shenzhen	Guangdong	South Central	Detention Center	--	3	110	2.7%	4
Wang XX, 2005 [84]	2003/08	Dongguan	Guangdong	South Central	Detention Center	--	7	103	6.8%	4
ChenL, 2006 [85]	2004	Shenzhen	Guangdong	South Central	Detention Center	--	2	245	0.8%	4
Hong Y, 2009 [86]	2004	H County	Guangxi	South Central	Entertainment	Ethnographic target sampling	13	450	2.9%	4

First author, published year	Study period	Location	Province	Region	Recuitment venue	Sampling method	Number of FSW used drug	Total number of FSW	Drug usage (%)	QA Score
Li N, 2007 [82]	2004	Henan	Henan	South Central	Sentinel sites	Continuous sampling	22	728	3.0%	3
Luo J, 2005 [87]	2004/11	Liuzhou	Guangxi	South Central	Entertainment	--	5	362	1.4%	6
Wu XT, 2005 [88]	2004/04-2004/10	Xinyang	Henan	South Central	Entertainment	--	47	465	10.1%	3
Li WJ, 2007 [89]	2005/11	Yangjiang	Guangdong	South Central	Entertainment	Cluster random sampling	1	285	0.4%	6
Long CW, 2008 [90]	2005/05-2005/06	Huangshi	Hubei	South Central	Entertainment	Random sampling	4	172	2.3%	4
Lu F, 2009 [91]	2005	Liuzhou	Guangxi	South Central	--	Snowball sampling	5	354	1.4%	3
Xu YF, 2007 [92]	2005/07-2005/09	Nanning	Guangxi	South Central	Entertainment	Random sampling	9	265	3.4%	7
Li N, 2007 [93]	2006		Henan	South Central	Mixed venues	Continuous sampling	44	5130	0.9%	5
Li XX, 2007 [94]	2006/01	Binyang, Nanning	Guangxi	South Central	Entertainment	--	8	364	2.2%	3
Zhang JL, 2008 [95]	2006/01-2006/10	Hezhou	Guangxi	South Central	Entertainment	--	9	203	4.4%	3
Zhou XL, 2007 [96]	2006/03-2006/05	Shenzhen	Guangdong	South Central	Entertainment	Random sampling	5	271	1.8%	4
Chen YH, 2009 [97]	2002-2007	Hezhou	Guangxi	South Central	Entertainment	--	4	1104	0.4%	5
Cheng ZQ, 2008 [98]	2006/07-2007/12	Nanning	Guangxi	South Central	Entertainment	--	0	879	0.0%	3
Li Y, 2012 [99]	2006/08-2007/01		Guangdong	South Central	Entertainment	Respondent-driven sampling	31	318	9.7%	6
Wang XX, 2008 [100]	2006 - 2007	Dongwan	Guangdong	South Central	Detention Center	--	7	228	3.1%	6

First author, published year	Study period	Location	Province	Region	Recuitment venue	Sampling method	Number of FSW used drug	Total number of FSW	Drug usage (%)	QA Score
Wen XQ, 2009 [101]	2007/04-2007/05	Guilin	Guangxi	South Central	Entertainment	Stratified cluster and random sampling	9	360	2.5%	3
Xu YF, 2009 [102]	2007	Nanning	Guangxi	South Central	Entertainment	Random sampling	5	379	1.3%	7
Zhang SJ, 2008 [103]	2007	Congzuo	Guangxi	South Central	VCT	--	13	385	3.4%	4
Zhang YX, 2011 [104]	2007/07-2007/09	Liu Zhou	Guangxi	South Central	Entertainment	--	7	403	1.7%	4
Zhang YX, 2011 [104]	2007/07-2007/09	Liu Zhou	Guangxi	South Central	Entertainment	--	7	317	2.2%	4
Zhang YX, 2011 [104]	2007/07-2007/09	Liu Zhou	Guangxi	South Central	Entertainment	--	0	86	0.0%	4
Bai Y, 2009 [105]	2008/04-2008/07	Liuzhou	Guangxi	South Central	Entertainment	Random sampling	44	431	10.2%	6
Bai Y, 2009 [105]	2008/04-2008/07	Liuzhou	Guangxi	South Central	Entertainment	Random sampling	9	449	2.0%	6
Bai Y, 2009 [105]	2008/04-2008/07	Liuzhou	Guangxi	South Central	Entertainment	Random sampling	0	167	0.0%	6
Huang XT, 2009 [106]	2008	Shantou	Guangdong	South Central	Entertainment	--	9	315	2.9%	4
Mao AL, 2010 [107]	2008/09-2008/10	Jingzhou	Hubei	South Central	Entertainment	--	22	288	7.6%	4
Wen YQ, 2011 [108]	2008/04-2008/07	Liu Zhou	Guangxi	South Central	Entertainment	--	6	712	0.8%	5
Wen YQ, 2011 [108]	2008/04-2008/07	Liu Zhou	Guangxi	South Central	Entertainment	--	47	320	14.7%	5
Xu YF, 2009 [102]	2008	Nanning	Guangxi	South Central	Entertainment	Random sampling	4	400	1.0%	7
Zhang L, 2010 [109]	2008/12	Shangcai	Henan	South Central	Entertainment	--	1	172	0.6%	3

First author, published year	Study period	Location	Province	Region	Recuitment venue	Sampling method	Number of FSW used drug	Total number of FSW	Drug usage (%)	QA Score
Zheng K, 2003 [110]	2002/06-2008/12		Hainan	South Central	Detention Center	--	229	1144	20.0%	3
Chen L, 2010 [111]	2009	Shenzhen	Guangdong	South Central	Entertainment	Random sampling	11	426	2.6%	2
Dun ZJ, 2011 [112]	2007-2009	Guang Zhou	Guangdong	South Central	Entertainment	--	48	303	15.8%	3
Nei ZQ, 2011 [113]	2009/04-2009/07		Guangdong	South Central	Mixed venues	Random sampling	175	5309	3.3%	5
Nie ZQ, 2011 [114]	2009		Guangdong	South Central	Entertainment	--	450	13620	3.3%	4
Wang JY, 2010 [115]	2009/05	Zhongshan	Guangdong	South Central	Community	Convenience sampling	21	520	4.0%	4
Xiang SB, 2010 [116]	2009	Hongjiang	Hunan	South Central	Entertainment	Random sampling	4	401	1.0%	4
Xiang Z, 2012 [117]	2009/07-2009/09	Wuzhou, Hezhou	Guangxi	South Central	Entertainment	Convenience sampling	99	810	12.2%	6
Zhong J, 2011 [118]	2009/06-2009/08	Wu Zhou	Guangxi	South Central	Entertainment	--	5	45	11.1%	4
Zhong J, 2011 [118]	2009/06-2009/08	Wu Zhou	Guangxi	South Central	Entertainment	--	59	648	9.1%	4
Zhong J, 2011 [118]	2009/06-2009/08	Wu Zhou	Guangxi	South Central	Entertainment	--	30	473	6.3%	4
Zhong J, 2011 [118]	2009/06-2009/08	Wu Zhou	Guangxi	South Central	Entertainment	--	113	1163	9.7%	4
Bai Y, 2012 [119]	2010/05-2010/07	Liu Zhou	Guangxi	South Central	--	--	136	1846	7.4%	4
Chen ZB, 2011 [112]	2010/04-2010/07	Lian Zhou	Guangdong	South Central	Entertainment	--	5	200	2.5%	3
Huang KZ, 2010 [120]	2010	Yangjiang	Guangdong	South Central	Entertainment	Random sampling	1	391	0.3%	4
Liang HX, 2011 [121]	2009-2010	Shi Yan	Hubei	South Central	--	--	5	800	0.6%	4

First author, published year	Study period	Location	Province	Region	Recuitment venue	Sampling method	Number of FSW used drug	Total number of FSW	Drug usage (%)	QA Score
Wu ZZ, 2011 [122]	2009/07-2010/01	Jiangmen	Guangdong	South Central	Entertainment	Convenience sampling	53	702	7.5%	6
Zhu L, 2011 [123]	2010/04-2010/06	Xiang Yang	Hubei	South Central	Sentinel sites	--	2	402	0.5%	4
Zhu L, 2011 [123]	2010/04-2010/06	Xiang Yang	Hubei	South Central	Sentinel sites	--	1	402	0.2%	4
Ke XZ, 2012 [124]	2011/04-2011/07	Huang Shi	Hubei	South Central	--	--	21	400	5.3%	3
Tang J, 2012 [125]	2011	Gui Lin	Guangxi	South Central	Entertainment	--	9	400	2.3%	4
Wei XQ, 201 2[124]	2011	He Chi	Guangxi	South Central	--	--	16	400	4.0%	2
Chen XS, 2005 [126]	1999/11-2000/05	Yunan	Yunnan	Southwest	Detention Center	Convenience sampling	293	505	58.0%	5
Wang SQ, 2004 [127]	2002/12-2003/01	Panzhihua	Sichuan	Southwest	Entertainment	--	5	178	2.8%	3
Jiang HY, 2006 [128]	2003/10	Yuanjiang	Yunnan	Southwest	Entertainment	--	2	216	0.9%	5
Jin Y, 2006 [129]	2003/03	Qujing	Yunna	Southwest	Sentinel sites	Random sampling	20	277	7.2%	6
Lai WH, 2009 [130]	2003		Sichuan	Southwest	Entertainment	Snowball sampling	213	2577	8.3%	4
Lei ZQ, 2005 [131]	2004/04	Dazhou	Sichuan	Southwest	Entertainment	Convenience sampling	6	259	2.3%	4
Wen Y, 2006 [132]	2004/03	Gejiu	Yunnan	Southwest	Mixed venues	--	62	415	14.9%	4
Xu SM, 2006 [133]	2004/04-2004/11	Chongqing	Chongqing	Southwest	Detention Center	Continuous sampling	599	1113	53.8%	8
Cao XY, 2006 [134]	2004/12-2005/01	Xichang	Sichuan	Southwest	Entertainment	--	24	203	11.8%	5
Cao XY, 2007 [134]	2004/12-2005/01	Xichang	Sichuan	Southwest	Entertainment	--	30	330	9.1%	5
Choi SY, 2011 [135]	2005	Na	NA	Southwest	Entertainment	Convenience sampling	22	200	11.0%	3

First author, published year	Study period	Location	Province	Region	Recuitment venue	Sampling method	Number of FSW used drug	Total number of FSW	Drug usage (%)	QA Score
Ding XB, 2006 [136]	2005/08-2005/09	Chongqing	Chongqing	Southwest	Entertainment	Convenience sampling	22	519	4.2%	4
Lv F, 2007 [137]	2004/07-2005/07	Sichuan	Sichuan	Southwest	Entertainment	Random sampling	16	43	37.2%	2
Ruan Y, 2006 [138]	2004/12-2005/01	Xichang	Sichuan	Southwest	Entertainment	--	32	343	9.3%	5
Tan XJ, 2007 [139]	2005/08-2005/09	Chongqing	Chongqing	Southwest	Entertainment	--	3	266	1.1%	5
Yuzhenlacuo, 2006 [140]	2005/06-2005/08	Lasa	Tibet	Southwest	Entertainment	--	32	1310	2.4%	2
Du JQ, 2008 [141]	2006	Kaiyuan	Yunnan	Southwest	Entertainment	--	79	366	21.6%	4
Huang Y, 2006 [142]	2006	Leshan	Sichuan	Southwest	Entertainment	--	56	362	15.5%	4
Ji JH, 2007 [143]	2006/10-2006/11	Changjiang	Hainan	Southwest	Entertainment	--	2	378	0.5%	4
Lei JH, 2012 [144]	2006	Kai Li	Guizhou	Southwest	--	--	0	400	0.0%	2
Li YY, 2009 [145]	2004/10-2006/12	Gejiu	Yunnan	Southwest	Entertainment	Random sampling	47	415	11.3%	4
Sun JY, 2012 [146]	2006	Jie Li	Guizhou	Southwest	Entertainment	--	0	400	0.0%	4
Wang H, 2009 [147]	2006/03-2006/05	Kaiyuan	Yunnan	Southwest	Entertainment	Convenience sampling	24	458	5.2%	4
Wang H, 2009 [147]	2006/03-2006/05	Kaiyuan	Yunnan	Southwest	Entertainment	Convenience sampling	27	279	9.7%	4
Wang HB, 2007 [148]	2006/03-2006/05	-	Yunnan	Southwest	Entertainment	--	120	737	16.3%	4
Wang HB, 2010 [149]	2006/03	Kaiyuan	Yunnan	Southwest	Entertainment	Venue-based sampling	120	737	16.3%	7
Wang HB, 2010 [150]	2006/03-2006/12	Kaiyuan	Yunnan	Southwest	Entertainment	Convenience sampling	261	1484	17.6%	6
Wang HB, 2010 [149]	2006/10	Kaiyuan	Yunnan	Southwest	Entertainment	Venue-based sampling	141	747	18.9%	7

First author, published year	Study period	Location	Province	Region	Recuitment venue	Sampling method	Number of FSW used drug	Total number of FSW	Drug usage (%)	QA Score
Wu Q, 2011 [151]	2006	Si Mao, Qin Lin, Da Li, Lu Feng, Meng Zi,	Yunnan	Southwest	Entertainment	Cluster sampling	13	1065	1.2%	4
Han DL, 2012 [152]	2007/04	Cheng Du	Sichuan	Southwest	Entertainment	--	68	400	17.0%	2
Han WX, 2012 [151]	2007	Long Chuan	Yunnan	Southwest	--	--	4	137	2.9%	2
He QX, 2011 [153]	2007	Luliang	Yunnan	Southwest	Entertainment	Two-stage probability sampling method	5	365	1.4%	4
He QX, 2011 [154]	2007	Lu Liang County	Yunnan	Southwest	Entertainment	--	5	365	1.4%	3
Jin X, 2009 [155]	2007/09-2007/11		Yunnan	Southwest	Entertainment	--	89	391	22.8%	4
Liu CQ, 2010 [156]	2007	Kunming	Yunnan	Southwest	--	--	3	365	0.8%	4
Luo L, 2009 [157]	2007/07-2007/09	Mianyang	Sichuan	Southwest	Entertainment	Random sampling	4	411	1.0%	7
Luo YJ, 2010 [158]	2007	Sichuan	Sichuan	Southwest	Entertainment	Stratified sampling	3	302	1.0%	5
Peng HB, 2007 [159]	2007/04-2007/05	Nanchong	Sichuan	Southwest	Entertainment	Stratified random sampling	18	420	4.3%	5
Wang HB, 2010 [149]	2007/05	Kaiyuan	Yunnan	Southwest	Entertainment	Convenience sampling	171	705	24.3%	6
Xu JJ, 2012 [160]	2007/03-2007-07	Kaiyuan	Yunnan	Southwest	Entertainment	Census	150	705	21.3%	6
Yao Y, 2012 [161]	2007/09-2007/10	Kaiyuan	Yunnan	Southwest	Entertainment	Convenience sampling	94	399	23.6%	7
Chen Y, 2009 [162]	2008/05-2008/07	Huangshi	Hubei	Southwest	Entertainment	Random sampling	4	172	2.3%	4
Dong LM, 2010 [163]	2008/04-2008/06	A District Zigong	Sichuan	Southwest	Entertainment	--	3	536	0.6%	6

First author, published year	Study period	Location	Province	Region	Recuitment venue	Sampling method	Number of FSW used drug	Total number of FSW	Drug usage (%)	QA Score
Han WX, 2012 [164]	2008	Long Chuan	Yunnan	Southwest	--	--	1	121	0.8%	2
Lei JH, 2012 [144]	2008	Kai Li	Guizhou	Southwest	--	--	16	400	4.0%	2
Li WZ, 2009 [165]	2008	Jianshui	Yunnan	Southwest	Entertainment	Probability proportional to size sampling	13	405	3.2%	6
Liu CQ, 2010 [156]	2008	Kunming	Yunnan	Southwest	--	--	1	370	0.3%	4
Sun JY, 2012 [146]	2008	Jie Li	Guizhou	Southwest	Entertainment	--	16	400	4.0%	4
Yang ZJ, 2010 [166]	2007-2008	Ruili	Yunnan	Southwest	Entertainment	--	14	751	1.9%	4
Zhu Q, 2009 [167]	2008	Chuxiong	Yunnan	Southwest	Entertainment	--	1	762	0.1%	4
Zi GS, 2009 [168]	2008/01	Weishan	Yunnan	Southwest	Entertainment	--	0	85	0.0%	4
Chen R, 2010 [169]	2009/02-2009/05	N/A	Sichuan	Southwest	Entertainment	Convenience sampling	13	332	3.9%	3
Dong LM, 2010 [163]	2009/04-2009/06	B District Zigong	Sichuan	Southwest	Entertainment	--	0	335	0.0%	6
Han WX, 2012 [164]	2009	Long Chuan	Yunnan	Southwest	--	--	0	128	0.0%	2
He QX, 2011 [154]	2009	Lu Liang County	Yunnan	Southwest	Entertainment	--	4	400	1.0%	3
Lei JH, 2012 [144]	2009	Kai Li	Guizhou	Southwest	--	--	15	400	3.8%	2
Liu CQ, 2010 [156]	2009	Kunming	Yunnan	Southwest	--	--	2	376	0.5%	4
Sun JY, 2012 [146]	2009	Jie Li	Guizhou	Southwest	Entertainment	--	15	400	3.8%	4
Wang QF, 2010 [170]	2009/05	Songming	Yunnan	Southwest	Entertainment	--	2	193	1.0%	3
Yan WZ, 2011 [171]	2009	Jiong Hong	Yunnan	Southwest	Entertainment	--	0	180	0.0%	3
Guo HJ, 2011 [172]	2010/04-2010/07	Zun Yi	Guizhou	Southwest	--	--	1	235	0.4%	4
Lei JH, 2012 [144]	2010	Kai Li	Guizhou	Southwest	--	--	68	400	17.0%	2
Sun JY, 2012 [146]	2010	Jie Li	Guizhou	Southwest	Entertainment	--	68	400	17.0%	4
Yang ZJ, 2012 [173]	2010/03-2010/06	Rui Li	Yunnan	Southwest	Entertainment	--	7	501	1.4%	4

First author, published year	Study period	Location	Province	Region	Recuitment venue	Sampling method	Number of FSW used drug	Total number of FSW	Drug usage (%)	QA Score
Zhou Z, 2012 [174]	2010/04-2010/06	Da Li	Yunnan	Southwest	Entertainment	--	16	2019	0.8%	4
Li Y, 2012 [175]	2010-2011	Lan Cang	Yunnan	Southwest	Entertainment	--	4	701	0.6%	3
Li YK, 2011 [176]	2011/05-2011/09	8 Counties	Sichuan	Southwest	--	--	15	368	4.1%	3
Zhang H, 2012 [177]	2011	Zhen Yuan	Yunnan	Southwest	--	--	0	1301	0.0%	2
Zhang XD, 2012 [178]	2010/07-2011/02	Kunming	Yunnan	Southwest	Entertainment	Snowball and convenience sampling	16	201	8.0%	6

References

1. Wang BF, Chen QJ, Zhang YH, Chen YL, Li SR. [Analysis on Behaviour Characteristics of Prostitute and Epidemiology of HIV and Syphilis]. Disease Surveillance. 2004;19(8):296-7.
2. Xiao Y, Zhang H, Wei X, Hu Y, Yang Z. [A Survey on HIV awareness and Characteristics of Sexual Behavior 201 Women Engaging in Clandestine Prostitution]. Chinese Journal of STD & AIDS Prevention and Control. 2002;8(5):296-8.
3. Xie HY. [Investigation on HIV and syphilis infection among 1996 female sex workers in Quanzhou City, Fujian Province]. Strait Journal of Preventive Medicine. 2004;10(6):44-5.
4. Yang JZ, Zou Y, Pan X, Yao Y, Li X, Guo Z. [Analysis of HIV/AIDS sentinel surveillance in Zhejiang Province, 2003]. Zhejiang Journal of Preventive Medicine. 2004;16(11):19-21.
5. Liu XZ, Liao MZ, Fu JH, Su SL, Huang T. [Analysis on HIV/AIDS Surveillance of Shandong Province in 2004]. Preventive Medicine Tribune. 2006;12(1):80-2.
6. Shen YG, Gu XJ. [Investigation on HIV infection, knowledge, attitude, behavior and practice among female sex workers in rural areas]. Chinese Journal of AIDS & STD. 2006;12(6):560, 6.
7. Yang JZ, Pan XH, Zou Y, Li XT, Yang Q, Xu Y. [Analysis on Behavior Surveillance of AIDS in Zhejiang Province in 2004]. Zhejiang Journal of Preventive Medicine. 2005;17(11):13-5.
8. Zhu X, Kang D, Liu X, Liao M, Fu J. [Analysis of behavioral changes among clandestine prostitutes in Jiaozhou city]. Chinese Journal of AIDS & STD. 2008;14(1):28-30.
9. Zou Y, Yang JZ, Pan XH, Yang Q, Yao YP, Li XT, et al. [Analysis of Sentinel Surveillance of AIDS Infection in Zhejiang Province in 2004]. Zhejiang Journal of Preventive Medicine. 2005;17(08):19-20.
10. Cheng XL, Wang FH, Xiao YK. [Analysis on the Data of HIV Sentinel Surveillance in 20002-2006 in Anhui Province]. Anhui Journal of Preventive Medicine. 2009;15(2):81-4.
11. Liao M, Liu X, Qian Y, Kang D, Fu J. [A Survey on AIDS Knowledge among Seven Population Groups in Shandong Province]. Preventive Medicine Tribune. 2007;13(5):404-6.
12. Liao M, Bi Z, Liu X, Kang D, Fu J, Song Q, et al. Condom use, intervention service utilization and HIV knowledge among female sex workers in China: results of three consecutive cross-sectional surveys in Shandong Province with historically low HIV prevalence. International journal of STD & AIDS. 2012;23(3):e23-9.
13. Luo Y, Chen SC, Ding JM, Cheng J, Xu K, Yuan H, et al. [Analysis of HIV/AIDS sentinel surveillance in Hangzhou]. Disease Surveillance,. 2008;23(11):717-9.
14. Luo Z, Zha YF, Huang ZM. [Analysis on surveillance of secret prostitutes in Songjiang district in year 2006 Shanghai]. Shanghai Journal of Preventive Medicine. 2007;19(4):184-5.
15. Sun Z, Xue F, Lin S, Wen M. [Survey of knowledge and behavior associated with AIDS in prostitutes in educational house]. China Tropical Medicine. 2008;8(03):511-2.
16. Chen SP, Tu BY, Wang X. [Survey and Analysis on HIV/AIDS-Related Behavior and Recognition among HIV/AIDS High-risk Population of Xunyang District Jiujiang City]. Chinese Journal of Evidence-Based Medicine. 2010;10(7):817-21.

17. Chen Y, Cai X, You T, Chen Q. [Analysis on risk behaviors and HIV/syphilis infection among female sex workers (FSWS) in detention home of Longyan city from 2001 to 2007]. Preventive Medicine Tribune. 2009;15(09):827-8.
18. Liao MZ, Liu XZ, Fu JH, Qian YS, Wang TZ. [Analysis of HIV/ AIDS Surveillance Data in Shandong Province in 2007]. Preventive Medicine Tribune 2008;14(12):1143-5.
19. Luo Y, Chen S, Xu K, Yuan H, Chen J, Hu J, et al. [Survey of STD/AIDS-related knowledge, behaviors and infection rates of sex workers in entertainment places in Hangzhou]. Disease Surveillance. 2008;23(10):607-9.
20. Ni YQ, Wang ZY, Shi CL. [Investigation on HIV/AIDS knowledge, attitude and practice (KAP) among female sex workers in three small-sized entertainment venues in Changning District, Shanghai]. Shanghai Journal of Preventive Medicine. 2008;20(11):537-8.
21. Peng B, Jiang M, Wu GZ, Sun YM, Ke CB. [Analysis on HIV epidemic among three high risk populations]. Chinese Journal of Disease Control & Prevention. 2008;12(6):631-2.
22. Qin CM, Li HW. [Analysis on HIV surveillance among 197 commercial sex workers (CSW)]. Medical Information. 2009;22(1):106.
23. Wang W, Xue L, Xia L, Tang Q, Shen L. [Survey of 297 sex workers and the infectious status of STD and HIV/AIDS]. China Tropical Medicine. 2008;8(05):827-8.
24. Chen L. [Analysis on the surveillance in observation sites of AIDS and venereal diseases in year 2008 Cong Ming county Shanghai municipality]. Shanghai Journal of Preventive Medicine. 2009;21(2):83.
25. Liao M, Jiang Z, Zhang X, Kang D, Bi Z, Liu X, et al. Syphilis and methamphetamine use among female sex workers in Shandong Province, China. Sex Transm Dis. 2011;38(1):57-62.
26. Liao M, Nie X, Pan R, Wang C, Ruan S, Zhang C, et al. Consistently low prevalence of syphilis among female sex workers in Jinan, China: findings from two consecutive respondent driven sampling surveys. PLoS One. 2012;7(4):e34085.
27. Tang X, Wu P, Li Y, Zhong Y, Yu X, Pan R, et al. [A study on AIDS related knowledge and behavioral characteristics among mini-type entertainment venues based female sex workers in Hongkou district of Shanghai]. China Preventive Medicine. 2010;11(02):162-5.
28. Wang F, Chen X, Su B, Ji G. [Analysis of the results of the comprehensive HIV/AIDS surveillance among sex workers in Anhui]. Anhui Journal of Preventive Medicine. 2009;15(06):407-8.
29. Xue F. [Survey of AIDS-related knowledge and infection rates of HIV, HBsAg and Syphilis among female sex workers in entertainment places in Lucheng district of Wenzhou city]. Chinese Journal of Health Laboratory Technology. 2009;19(7):1649-51.
30. Yang Y, Yao J, Gao M, Su H, Zhang T, He N. Herpes simplex virus type 2 infection among female sex workers in Shanghai, China. AIDS Care. 2011;23 Suppl 1:37-44.
31. Zhang XJ, Liao MZ, Kang DM, Tao XR, Qian YS, Wang GR, et al. [Condom Use and Correlates Among Female Sex Workers in Shandong Province,2006-2008]. Preventive Medicine Tribune. 2012;18(6):405-7+10.
32. Kang D, Liao M, Jiang Z, Zhang X, Mao W, Zhang N, et al. Commercial sex venues, syphilis and methamphetamine use among female sex workers. AIDS Care. 2011;23 Suppl 1:26-36.

33. Liao MZ, Liu XZ, Kang DM, Fu JH, Wang TZ, Qian YS, et al. [Analysis on the HIV/AIDS Surveillance Data in Shandong Province in 2009]. Preventive Medicine Tribune. 2010;16(5):398-400, 3.
34. Wan LJ, Zhang XX, Gu XM. [A study on HIV/AIDS-related knowledge, attitudes and practices among female sex workers working at different venues]. Zhejiang Journal of Preventive Medicine. 2011;23(5):81-2, 5.
35. Wang F, Chu Y, Wang L, Wang F. [Serological survey and investigation on AIDS knowledge and behavior among CSW in Huaiyuan county]. Anhui Journal of Preventive Medicine. 2010;16(03):190-1+202.
36. Xi SJ, He YF, Zhou XH. [Analysis on the Result of AIDS Test Intervention in CSW, MSM, IDU Population in Xiacheng District]. Zhejiang Journal of Preventive Medicine. 2010;22(9):29-30.
37. Xi SJ, He YF, Zhou XH, Zhou DD, Wang CC. [A Survey on the Status and Wishes of HIV Voluntary Counseling and Testing and Its Influencing Factors among Community Female Commercial Sex Workers]. Zhejiang Journal of Preventive Medicine. 2011;23(1):8-10+6.
38. Xi S, He Y, Zhou X, Zhou D, Wang C. [A Survey on the status and wishes of HIV voluntary counseling and testing and its influencing factors among community female commercial sex workers]. Zhejiang Journal of Preventive Medicine. 2011;23(1):8-10, 6.
39. Zhang QQ, Huan XP, Yin YP, Wang XL, Hu HY, Jiang N, et al. [Incidence rates of sexually transmitted infection and the characteristic analysis of female sex workers failing in follow-up in cohort study]. Acta Universitatis Medicinalis Anhui. 2012;47(9):1050-4.
40. Chen CC, Lin H, Zhang H. [AIDS knowledge Levels and Behavior Characteristics of 225 Female Sex Workers in Entertainment Places of Fuzhou City]. Occupation and Health. 2011;27(2):170-2.
41. Jiang J, Wang HB, Fang WM, Sun JL, Chen BB, Bo DY, et al. [Survey of AIDS prevalence in female sex workers detained in a correctional facility in Ningbo, Zhejiang]. Disease Surveillance. 2012;27(08):634-6.
42. Miao XL, Cheng H, Zhang X, Gu J, Ji YY, He EQ. [Analysis on HIV /AIDS Sentinel Surveillance in Wuxi City in 2010]. Occupation and Health. 2011;27(22):2599-601.
43. Qian ZH, Wang J, Fan XQ. [HIV/AIDS sentinel surveillance results among prostitutes in Suzhou city]. Jiangsu Journal of Preventive Medicine. 2012;23(1):30-1.
44. Sun XQ, Tang GX, Mao TS, Zhang YJ, Mao N. [2010 baseline survey among commercial sex workers of Taihe County]. China Modern Medicine. 2011;18(28):151-3.
45. Tang ZL, Li XF, Dong XP, Wang YF, Chen GZ. [Infection of HIV/STDs through sexual behavior among female sex workers during menstruation]. Chinese Journal of Public Health. 2011;27(12):1510-2.
46. Sun BJ. [Survey on AIDS-related Knowledge, Behavior and HIV-infection Among Commercial Sex Workers in Shizhong District, Zaozhuang City, 2011]. Preventive Medicine Tribune. 2012;18(9):654-6.
47. Yang YH, Gong CT, Wang ZQ, Chen CY. [Analysis of national sentinel monitoring of AIDS infection in Quanzhou City in 2011]. Strait Journal of Preventive Medicine. 2012;18(6):32-3.
48. Ye ZM, Wang DY, Zhang HM, Zhao LN, XUE FH, Jin Q, et al. [Survey on HIV/AIDS -related knowledge and behaviors among FSWs]. Zhejiang Journal of Preventive Medicine. 2012;24(11):74-6.
49. Lin Z, Liu GY, Li ZM, Zhao RL, Zhang XM, Chen Y, et al. [Analysis of HIV surveillance among 364 female sex workers in Tongliao City, Inner Mongolia]. Chinese Journal of Public Health. 2007;23(3):355.

50. Laio GW, Zhong FH, Liang JM, Jiang Y, Ning JR, Tang MJ, et al. [Seroepidemiological study of HIV among targeted population in Yulin City in Guangxi Province]. Journal of Applied Preventive Medicine. 2006;12(4):221-3.
51. Shi W, Bai J, Li J, Qu Y. [A survey on KAB of AIDS among 114 female commercial sex workers in Fengtai District in Beijing]. Chinese Journal of Health Education. 2007;(02):120-2.
52. Bai JM, Shi WY, Xie HY, Qu YM, Zhai CX, Tian ML, et al. [Prevalence of high-risk behavior and HIV infection among bargirls in Fengtai District of Beijing]. The Chinese Journal of Human Sexuality 2007;16(6):45-6, 8.
53. Liu L, Liu M, Lu H, Xia D. [Analysis of HIV/AIDS related risk behaviors among female sex workers at entertainment establishments in two districts of Beijing]. Chinese Journal of AIDS & STD. 2007;13(6):532-5.
54. Shi WY, Xie YY, Liu C. [Behavioral and serological surveillance among female sex workers in Fengtai district, Beijing from 2006-2009]. Chinese Journal of Public Health. 2012;28(01):109-10.
55. Dong XY, Zhou N, Guo Y, Yu MH. [Analysis of HIV/AIDS Sentinel Surveillance among female sex workers and male clients in Tianjin City, 2007]. South China Journal of Preventive Medicine. 2009;35(2):40-1.
56. Jia J, Gao LQ, Xing LY, Han YD, Liu M, Lei XY, et al. [Investigation on HIV-related knowledge and praxeology among commercial sex workers in Beilin District, Xi'an City]. Chinese Journal of Misdiagnostics. 2011;11(32):7940-1.
57. Cao H. [Analysis of high risk AIDS-related behavior characteristics among 186 female sex workers at Xiqing district of Tianjin]. Port Health Control. 2010;15(5):27-9.
58. Li BY, Zhao XH, Wang GR. [Recognition of AIDS among unlicensed prostitutes in Jinnan district of Tianjin]. Occupation and Health. 2012;28(4):465-6.
59. Liu HX, Ma SB, Li F, Wang HS, Hou Z. [Analysis of AIDS sentinel surveillance among commercial sex workers in Changping District from 2005-2010]. Chinese Journal of AIDS & STD. 2011;17(5):579-80.
60. Liu ZJ, Jiang L, Wang CB, Liu TT. [Sentinel surveillance of female sex workers in Luannan County in 2011]. Journal of Capital Medical University. 2012;33(5):621-4.
61. Cui YZ, Zhu L, Yuan LL, Zhao YS, Wang J. [AIDS knowledge, behavior and HIV/syphilis infection survey among FSW in Harin from 2006 to 2009]. Chinese Journal of AIDS & STD. 2011;17(3):351-2.
62. Li Y, Du J. [HIV knowledge and sexual behaviours characteristics among female sex workers in Huanggu district, Shenyang City]. Disease Monitor and Control. 2008;2(4):195-6.
63. Shao B, Yao SP, Wang KL, Yang JQ, Cao B, Wang J, et al. [The survey of AIDS knowledge behaviors and condom use among female sex workers in Heilongjiang Province]. Chinese Journal of Disease Control & Prevention. 2011;v.15(04):318-22.
64. Li Y, Wang KL, Tong X, Yan HM. [Analysis of sentinel monitoring of AIDS among high risk population in Heilongjiang Province in 2010]. Chinese Primary Health Care. 2011;25(5):59-61.
65. Xian XJ. [Analysis of sentinel monitoring on HIV/AIDS in Nongan County of Jilin Province]. Blooming Season. 2011;462(22):261.
66. Ni M, Liu Y, Chen J, Wang D, Dong Y, Gong X, et al. [A comprehensive survey on HIV/AIDS in Kashgar prefecture of Xinjiang]. Chinese Journal of AIDS & STD. 2005;11(5):353-6.
67. Liu Y, Yang X. [HIV knowledge, behavioural characteristics and condom usage among 415 female sex workers]. Modern Preventive Medicine. 2007;34(21):4144-5.

68. Song Y, Muheta, Aziguli, Yimiti, Aibibula, Zhang H, et al. [A survey of sexually transmitted diseases among entertainment-based female sex workers in Turpan City, Xinjiang, in 2006]. Endemic Diseases Bulletin. 2006;21(06):36-7.
69. Zeng K, Lin B, Wang F, Meng Y, Guo J. [Investigation on Risk Behavior of Sexual Transmitted Diseases/ADIS among Commercial Workers in Entertainment Places in Karamay City]. Preventive Medicine Tribune. 2008;14(02):124-6.
70. Chang WH, Xing AH, Wang BS, Li X, Jia H, Zhang L, et al. [Analysis of HIV /AIDS sentinel surveillance among high risk population in Shanxi in 2010]. Occupation and Health. 2012;28(4):399-402.
71. Hu XQ, Yan CY, Yang PR, Tian H, Li Z, Cui XL. [Analysis of monitoring AIDS high-risk populations in 2011]. Journal of Preventive Medicine of Chinese People's Liberation Army. 2011;29(6):437-8.
72. Zhang MN, Zhang ZH, Huang L, Wang XM. [An investigation of sexually transmitted infection and related behavioral feature on unlicensed prostitutes in Shanxi province]. Chinese Remedies & Clinics. 2011;11(9):1051-2.
73. Zhao GD, Zhong L, Li YY. [Analysis of AIDS sentinel surveillance among illicit prostitutes in Shangluo City in 2010]. Journal of Hebei United University(Health Sciences). 2011;13(4):469-70.
74. Guo H, SheN MX, Ma Q, Zhang WX, Pang RP, Li GB. [The HIV Risk Behavior Surveillance Analysis for 820 Slip Women in Longnan Municiple]. Chinese Primary Health Care. 2012;26(4):65-6.
75. Qu S, Liu W, Choi K-H, Li R, Jiang D, Zhou Y, et al. The Potential for Rapid Sexual Transmission of HIV in China: Sexually Transmitted Diseases and Condom Failure Highly Prevalent Among Female Sex Workers. AIDS Behav. 2002;6(3):267-75.
76. Ding Y, Detels R, Zhao Z, Zhu Y, Zhu G, Zhang B, et al. HIV infection and sexually transmitted diseases in female commercial sex workers in China. J Acquir Immune Defic Syndr. 2005;38(3):314-9.
77. He QY, Kuang JS, Pan WL, Huang HZ, Yong CZ, Feng CY, et al. [Investigation on AIDS Related Behaviors in Different Population Groups in Hainan Province]. Chinese Journal of STD & AIDS Prevention and Control. 2002;8(5):274-7.
78. Pei D, Yang B, Lai S, Wang F. [High risk behavior investigation and syphilis infection surveillance among female sex workers and drug users in Hainan]. Chinese Journal of STD & AIDS Prevention and Control. 2002;8(3):160-1.
79. Zhou YJ, Liu W, Guo WG, Li RJ, Lu WJ, Chen L, et al. [Survey on HIV/STIs - related behaviors in female workers in recreation service]. Guangxi Journal of Preventive Medicine 2005;11(3):153-4.
80. Wang Y, Fu X, Lin P, Wu B, Liu Y, Xu R, et al. [Survey of STD/ AIDS related knowledge and behaviors among community female sex workers]. South China Journal of Preventive Medicine. 2004;30(6):18-20.
81. Zhao QQ, Liu YY, Lin P, Wang Y, He Q, Fu XB, et al. [HIV surveillance in Guangdong province, 2001-2002]. South China Journal of Preventive Medicine. 2004;30(3):2.
82. Li N, Sun G. [Analysis of AIDS sentinel surveillance among commercial sex workers in Henan Province]. Disease Surveillance. 2007;22(09):610-1.
83. Peng S, Hong F, LI Y, Wang L, Zhang R, Huang Z. [A survey of Chlamydia Trachoma injection status among 110 female sex workers]. Modern Preventive Medicine. 2004;(03):413-4.

84. Wang X, Zhang Q, Liu Y, Yang T, Chen W, Chen P. [Surveillance reports on AIDS and Syphilis of 103 prostitute women in Dongguan city]. Disease Surveillance. 2005;20(03):121-2.
85. Chen L, Feng TJ, Tan JG, Shi XD, Wang XH, Cai WD, et al. [Analysis of HIV/AIDS sentinel surveillance in Shenzhen]. Chinese Journal of AIDS & STD. 2006;12(2):136-9.
86. Hong Y, Li X, Yang H, Fang X, Zhao R. HIV/AIDS-related sexual risks and migratory status among female sex workers in a rural Chinese county. AIDS Care. 2009;21(2):212-20.
87. Luo J. [Analysis of the results from HIV Surveillance of unlicensed prostitutes]. Disease Surveillance. 2005;20(8):409-12.
88. Wu X. [An analysis of sexual behaviours among 465 female sex workers in Xinyang City]. Journal of Medical Forum. 2005;26(17):5-7.
89. Li W, Li Y, Mai R, Lin P, Yang L, Liu Y, et al. [Svey of STD and AIDS Knowledge and High-risk Behavior of Female Sexual Workers in Urban Area of Yangjiang City]. China Tropical Medicine. 2007;7(10):1921-2+40.
90. Long CW, Ng XH, Xiao SQ, Ke XZ, Chen WS, Deng J. [Analysis of AIDS knowledge and behavoral features of female sex workers in entertainment places]. China Tropical Medicine. 2008;8(4):697-700.
91. Lu F, Jia Y, Sun X, Wang L, Liu W, Xiao Y, et al. Prevalence of HIV infection and predictors for syphilis infection among female sex workers in southern China. Southeast Asian J Trop Med Public Health. 2009;40(2):263-72.
92. Xu YF, Mo XJ, Liang HH, Zhou FH, Li P, Zhou J, et al. [Investigation on STD/AIDS knowledge and risk behaviors among commercial sex women in Nanning City]. Modern Preventive Medicine. 2007;34(21):4007-8, 11.
93. Li N, wang Z, Sun GG, Sun DY. [Analysis of HIV/AIDS sentinel surveillance among high risk population in Henan province in 2006]. Chinese Journal of AIDS & STD. 2007;13(5):427-9.
94. Li X, Li Q, Wei X. [A study of STD/AIDS knowledge, belief and behaviours among 364 female sex workers]. Applied Prev Med. 2007;13(6):1.
95. Zhang JL, Liao YZ, Huang JW, Mai XY. [Investigation on HIV/STD-related knowledge and behaviours among 203 high-risk female in Hezhou city of Guangxi Zhuang Autonomous Region]. Journal of Applied Preventive Medicine. 2008;14(Z1):2.
96. Zhou XL, Yan QR, Liu DA, Ren XL. [Investigation on knowledge, attitude and behavior about HIV/AIDS among female servants of recreational places in Shenzhen]. Shanghai Journal of Preventive Medicine. 2007;19(1):13-5.
97. Chen Y. [Analysis of HIV sentinel surveillance among unlicensed prostitutes in Hezhou city during 2002-2007]. Occupation and Health. 2009;25(16):1738-9.
98. Chen ZJ, Xia WZ. [Investigation on HIV infection among 879 commercial female sex workers in Nanning City in Guangxi Province]. Journal of Guangxi Traditional Chinese Medical University. 2008;11(1):30-1.
99. Li Y, Detels R, Lin P, Fu X, Deng Z, Liu Y, et al. Difference in risk behaviors and STD prevalence between street-based and establishment-based FSWs in Guangdong Province, China. AIDS Behav. 2012;16(4):943-51.
100. Wang XX, Zhang QL, Chen BF, Fang XJ. [Sex criminal HIV / syphilis surveillance report in Dongguan, Guangdong province]. Disease Surveillance. 2008;23(08):490-2.
101. Wen X. [Survey on KAB and serology of AIDS among 360 female commercial sex workers in Guilin]. Modern Preventive Medicine. 2009;36(14):2687-9.

102. Xu YF, Zhou FH, Mo SJ, Li SS, He Y, Huang CH. [Surveillance of Commercial Sex Workers in Nanning, 200 -2008]. Journal of Preventive Medicine Information. 2009;25(8):615-7.
103. Zhang SJ. [Characteristics and trend of HIV/AIDS epidemic in Chongzuo city of Guangxi, 1996-2007]. Internal Medicine of China. 2008;3(06):932-5.
104. Zhang YX, Lin HT, Feng WD, Shan GS, Zhang TJ. [Syphilis and HIV infection status among commercial sexual workers in Liuzhou, Guangxi]. Journal of Tropical Medicine. 2011;11(3):337-9+55.
105. Ba iY. [Investigation on AIDS related knowledge, behaviors and infection among commercial female sex workers in Liuzhou city in 2008]. Preventive Medicine Tribune. 2009;15(12):1224-6.
106. Huang XT, Lin ZW, Mao XT, Chen SN. [Investigation on HIV/AIDS related risk behaviors among female sex workers in entertainment venues in Chaoyang District of Guangdong Province]. South China Journal of Preventive Medicine. 2009;35(1):33-4.
107. Mao A, Shi X, Xiao L, Zhang J, Peng J. [Effectiveness evaluation of HIV knowledge and behavioural interventions among lower-end female sex workers in Jingzhou City, China]. J of Pub Health and Prev Med. 2010;v.21(02):128-9.
108. Weng YQ, Bai Y, Feng WD. [High risk behavior toward HIV/AIDS of female sexual service workers with different marital status in Liuzhou City]. China Tropical Medicine. 2011;11(2):170-1.
109. Zhang L, Xue FH, Zhang XQ, Dong SB. [Study on HIV/AIDS related knowledge and behavior among female sex workers in Xincai County of Henan Province]. Henan Journal of Preventive Medicine. 2010;21(3):235-7.
110. Zheng W, Zhong N, Zhu H, Wang F, Lu Y, Zhang L. [Analysis of the Monitoring Results of 1144 Unlicensed Prostitutes for Infection of Hepatitis B Virus(HBV) , Human Immunodeficiency Virus (HIV) and Syphilis]. The Chinese Journal of Dermatovenereology. 2009;23(8):502-3.
111. Chen L, Tan J, Shi X, Gan Y, Zhang Y, Zhao J, et al. [The comprehensive surveillance of AIDS among unlicensed female sex workers in Shenzhen city]. Journal of Tropical Medicine. 2010;10(6):748-9.
112. Dun ZJ, Ling L, Xia HY, Wang C, Lin AH, Lu CY. [Survey on HIV/AIDS related KABP of four high-risk population in Guangzhou City of Guangdong Province]. Chinese Journal of Health Education. 2011;v.27(11):843-6.
113. Nie ZQ, Lin P, Li Y, Wang Y. [Surveillance of AIDS high-risk people in Guangdong province,2009]. Journal of Tropical Medicine. 2011;11(01):29-31+45.
114. Nie ZQ, Lin P, Li Y, Wang Y. [Surveillance of AIDS high-risk people in Guangdong province, 2009]. Journal of Tropical Medicine. 2011;11(1):29-31+45.
115. Wang J, Wang T, Cen Y, Lai X, Li L, Chen C, et al. [Influencing factors of condom use and HIV infection in female sex workers in Zhongshan City]. South China Journal of Preventive Medicine. 2010;36(4):26-8, 34.
116. Xiang S, Peng J, Tang J, Hu C. [HIV knowledge, attitude and behavioural survey among female sex workers in Hongjiang city]. Practical Preventive Medicine. 2010;17(3):596-7.
117. Xiang Z, Yin YP, Shi MQ, Jiang N, Han Y, Wang HC, et al. Risk factors for Mycoplasma genitalium infection among female sex workers: a cross-sectional study in two cities in southwest China. BMC public health. 2012;12:414.
118. Zhong J, Lin J, Hu YM, Tan LL, Wang G. [HIV/STD infection and risk behaviors among commercial sex workers at various places in Wuzhou City, Guangxi Zhuang Autonomous Region]. Chinese Journal of Health Education. 2011;27(3):177-80.

119. Bai Y, Zhang JP, Ouyang Y. [Analysis on monitoring results of AIDS among female sexual workers in Liuzhou City in 2010]. Chinese Journal of Pest Control. 2012;28(9):964-6.
120. Huang KZ, Chen XH, Li WJ, Chen ZF. [Survey of STD and AIDS knowledge and high risk behavior of female sexual workers in Yangjiang City]. South China Journal of Preventive Medicine. 2010;36(04):41-2.
121. Liang HX, Deng X, Tang GZ, Li Y, Mao LF. [Investigation of AIDS among Female Sex Workers in A District of Shiyan City during 2009-2010]. Occupation and Health. 2011;27(20):2338-9.
122. Wu ZZ, Deng WJ, Zhu X, Huang SJ, Chen XS, Jiang N. [Study of commercial sex workers positive of syphilis serum and its related factors]. Qingdao Medical Journal. 2011;42(5):321-4.
123. Zhu L, Qiu XS, Xie AQ, Gong WS, Wen MX, Yang XZ. [Analysis of AIDS Sentinel Surveillance among prostitutes in Xiangyang in 2010]. Journal of Public Health and Preventive Medicine. 2011;22(3):92-3.
124. Ke XZ, Xiong F, Xie RQ, Peng YH, Song W. [Analysis of sentinel monitoring of HIV, syphilis and HCV in Huangshi City in 2011]. Journal of Public Health and Preventive Medicine. 2012;23(5):80-1.
125. Tang J, Zhang ZK, Zhou Y, Wen XQ, Zhou HJ. [Analysis of sentinel monitoring of AIDS among high risk population in Guilin,2011]. Chinese Journal of AIDS & STD. 2012;18(8):533-6.
126. Li Q, Li X, Stanton B, Wang B. Psychometric properties of a pictorial scale measuring correct condom use. AIDS Behav. 2011;15(2):432-40.
127. Wang S, Yue Z, He L, Lu P, Tang Z, Liu X, et al. [Analyzing demands of FSWs for STD/AIDS prevention and treatment in different high risk sites in Panzhihua]. Chinese Journal of AIDS & STD. 2004;10(5):359-61.
128. Kan HY, Du CH, Long P, He R, Wen RB, Li SY. [Analysis on HIV/AIDS comprehensive intervention among female sex workers in entertainment venues in Yuanjiang County, Yunnan Province]. Soft Science of Health. 2006;20(3):262-3.
129. Jin Y, Yin GG, Bao WS, Liu CB, Mao BB, Deng GY, et al. [Study on HIV/AIDS behavior intervention in the waitresses of inns around roads]. Soft Science of Health. 2006;20(1):55-8.
130. Lai W, Zhou D, Zhang L, Zeng Y, Huang T. [Risk behaviors of AIDS among female CSW and also IDU]. Parasitoses and Infectious Diseases. 2009;7(2):80-3.
131. Lei Z, Du M, Wang Z, Zhong X. [Analysis of serological and behavioral survey results for HIV and TP in 259 female sex workers (FSW) from communities in 2004] Disease Surveillance. 2005;20(09):30-2.
132. Wen Y, Zhang Q, Ren XQ, Fu YF, Yang B, Fang RP, et al. [A study of the role of maternal and child health organisations in HIV/STD prevention among female sex workers]. Soft Science of Health. 2006;20(03):287-90.
133. Xu SM, Qian TX, Peng CL, Yang MF, Lu RR. [Surveillance of HIV and syphilis in Chongqing in 2004]. Modern Preventive Medicine. 2006;33(05):833-4.
134. Cao XT, Ruan YH, Jiang ZQ, Liang S, Qin GM, Chen KL, et al. [Study on sexually transmitted diseases and related risk factors among female sex workers who have regular partners]. Chinese Journal of Preventive Medicine. 2006;40(2):144.
135. Choi SYP. State Control, Female Prostitution and HIV Prevention in China. The China Quarterly. 2011;205:96-114.

136. Ding X, Yi H, Jiang X, Han L, Wu G, Ling H, et al. [Analysis of status of AIDS related knowledge, attitude and risk behavior among 519 female sex workers in Chongqing]. Chinese Journal of AIDS & STD. 2006;12(04):347-9.
137. Lv F, Luan R, Lei Y, Wang L, Huang Y, Zhou C, et al. [Study on HIV/AIDS related behaviors among street-based FSWs and their male clients in a city of Sichuan province]. Chinese Journal of AIDS & STD. 2007;13(2):114-7.
138. Ruan Y, Cao X, Qian HZ, Zhang L, Qin G, Jiang Z, et al. Syphilis among female sex workers in southwestern China: potential for HIV transmission. Sex Transm Dis. 2006;33(12):719-23.
139. Tan X, Yi H, Wang Z, Yu X, Zheng Y, Tang C, et al. [An analysis of knowledge, attitude and the feature of high risk behavior on AIDS among 266 unlicensed prostitutes]. Modern Preventive Medicine. 2007;34(02):253-5+62.
140. Yuzhen LC, Ya X, Ma YG, Gama ZM, Duoji WM, Suolang DJ, et al. [Investigation on HIV infection and related behaviour among female working at entertainment venues in Lhasa City of Tibet Autonomous Region]. Chinese Journal of AIDS & STD. 2006;12(6):545, 2.
141. Du J, Wang G, Wang W, Gu J, Chang D, Zhao M, et al. [Analysis on the survey of HIV/AIDS/STI among the commercial sex workers in Kaiyuan city]. Soft Science of Health. 2008;22(1):84-6.
142. Huang Y, Zhou XW, Fan L, Guo ZH, Qiu XM. [A survey on HIV/AIDS knowledge and sexual behaviour among commercial female sex workers in Leshan City, Sichuan Province]. Journal of Preventive Medicine Information. 2006;22(1):73-4.
143. Ji J. [Results of monitoring of AIDS behavior of female sexual workers in Changjiang County]. China Tropical Medicine. 2007;7(10):1853-4.
144. Lei JH, Xiao YL, Sun JY. [Analysis of the changing trend of AIDS high-risk behaviors among CSWs in Kaili city]. Chinese Journal of AIDS & STD. 2012;18(2):124-6.
145. Li Y, Wen Y, Hu Z, Chen W, He L, Yang F. [Integrated AIDS Behavioral Intervention Among Female Sex Workers in Gejiu City]. Journal of Kunming Medical University. 2009;v.30(09):113-7+21.
146. Sun JY, Xiao YL, Huang GX, Lei JH. [Monitoring on AIDS behavior among prostitutes in Kaili from 2006 to 2010]. Modern Preventive Medicine. 2012;39(14):3593-8+600.
147. Wang H, Chen RY, Ding G, Ma Y, Ma J, Jiao JH, et al. Prevalence and predictors of HIV infection among female sex workers in Kaiyuan City, Yunnan Province, China. Int J Infect Dis. 2009;13(2):162-9.
148. Sun X, Wang N, Li D, Zheng X, Qu S, Wang L, et al. The development of HIV/AIDS surveillance in China. AIDS. 2007;21 Suppl 8:S33-8.
149. Wang HB, Smith K, Brown KS, Wang GX, Chang DF, Xu JJ, et al. Prevalence, incidence, and persistence of syphilis infection in female sex workers in a Chinese province. Epidemiol Infect. 2011;139(9):1401-9.
150. Wang HB, Smith K, Brown KS, Wang GX, Chang DF, Xu JJ, et al. Prevalence, incidence, and persistence of syphilis infection in female sex workers in a Chinese province. Epidemiol Infect. 2010:1-9.
151. Wu Q, He CY, Duo L, He LM, Chen Y, Wang YY. [HIV/AIDS-related Risk Behaviour in Female Sex Workers, Southwest China]. Science & Technology Information. 2011;(13):9-10.
152. Lv CX, Jiang ZX, Zhang XJ, Zhang XF, Fu JH. [Survey of AIDS and syphilis infections among female sex workers in low-grade entertainment venues]. Chinese Journal of AIDS & STD. 2011;17(5):550-2.

153. He QX, Xu ZQ, Wang YB, Ma GS. [Evaluation on comprehensive HIV/AIDS intervention for female sex workers in Luliang City in Yunnan Province]. Soft Science of Health. 2011;25(2):115-8.
154. He QX, Xu QZ, Wang YB, Ma GS. [Analysis of the effect of Comprehensive Analysis on illicit prostitutes in Luliang County, Yuinan]. Soft Science of Health. 2011;25(2):115-8.
155. JIn X, Wang N, Ding G, Chang D, Wang G, Yao Y, et al. [Correlates of and willingness to the participation in HIV routine counseling and testing among female sex workers in a city of Yunnan province]. Chin J Epidemiol. 2009;30(2):205-6.
156. Liu CQ, Hou WJ, Kong XS, Su J, Xi JM, Zhang JX, et al. [Evaluation on HIV prevention project for high risk populations in Chengjiang County in Yunnan Province]. Soft Science of Health. 2010;24(4):335-8.
157. Luo JF. [Analysis of HIV/AIDS surveillance in Wuyishan City in Fujian Province, 2002-2006]. Chinese Journal of Ethnomedicine and Ethnopharmacy. 2009;18(13):71-2.
158. Luo Y, Lai W, Deng B, Xi J, Zhang L, Pan X. [Survey of AIDS-related behavior of sex workers in a certain place in Sichuan province]. Morden Preventive Medicine. 2010;37(2):210-2.
159. Peng H, Fu G, Feng Y, Tian X, Feng J, Zhang Q, et al. [A Survey on CSW's AIDS knowledge, behavior and intervention in an urban district in Nanchong]. Journal of North Sichuan Medical College. 2007;22(05):428-31.
160. Xu JJ, Smith MK, Chu J, Ding GW, Chang DF, Sharp GB, et al. Dynamics of the HIV epidemic in southern China: sexual and drug-using behaviours among female sex workers and male clients in Yunnan. International journal of STD & AIDS. 2012;23(9):670-5.
161. Yao Y, Yang F, Chu J, Siame G, Lim HJ, Jin X, et al. Associations between drug use and risk behaviours for HIV and sexually transmitted infections among female sex workers in Yunnan, China. International journal of STD & AIDS. 2012;23(10):698-703.
162. Chen Y, Xiong H, Xiong F. [AIDS knowledge and behavior of female sexual workers in rural areas]. China Tropical Medicine. 2009;9(8):1645-6.
163. Dong L, Li Q, Chen X, Zhou M, Xie Y. [Surveillance of behavior among female sex workers in Zigong, 2008-2009]. Journal of Preventive Medicine Information. 2010;26(12):982-5.
164. Chen Y, Li X, Zhou Y, Zhang C, Wen X, Guo W. Alcohol consumption in relation to work environment and key sociodemographic characteristics among female sex workers in China. Subst Use Misuse. 2012;47(10):1086-99.
165. Li W. [HIV sentinel surveillance and analysis among female sex workers in Jianshui county, China]. Soft Science of Health. 2009;23(06):711-3.
166. Yang ZJ, Yin ZL, Li ZL, Liu B, Fang KF, Chu XQ. [Investigation on HIV/AIDS epidemic among female sex workers in entertainment establishments in Ruili City, Yunnan Province]. Soft Science of Health. 2010;24(4):373-5.
167. Zhu Q, Wang L, He CY, Zhang XB, Yao XZ. [Analysis on behavioural surveillance among 762 female sex workers]. Journal of Dematology and Venereology. 2009;31(2):45-6.
168. Zi G, Yao H, Cha X, Guo J, Zi Z. [Investigation on the Status Related to AIDS among Female Sex Workers in Weishan County in 2008]. Preventive Medicine Tribune. 2009;15(11):1092-3.

169. Chen R, Yang Y, Hou C, Su HW. [Survey on attitude of female sex workers to voluntary HIV counseling and testing services in one city]. Journal of Luzhou Medical College. 2010;33(4):434-6.

170. Wang QF. [Analysis on HIV/AIDS knowledge and behaviour among prostitutes in Songming County, Yunnan Province]. Medical Information. 2010;23(4):928-9.

171. Yan WZ, Zheng KQ, Feng DL, Cai XY, Liu JR, Long Y, et al. [Investigation on status of STDs and AIDS among female sex workers in JingHong County of Yuinan in 2009]. Journal of Dermatology and Venereology. 2011;33(1):51+4.

172. Guo HJ, Feng D, Chen ZY, Zhou CX, Sun X, Chen ZM. [AIDS Knowledge Levels and Behavior Characteristics of FSWs in Places of Entertainment in Zunyi City]. Occupation and Health. 2011;27(22):2603-4.

173. Yang ZJ, Yin ZL, Li ZL, Liu B, Fang KF, Li RC, et al. [Analysis of AIDS Sentinel Surveillance in Ruili City in 2010]. Soft Science of Health. 2012;26(9):785-7.

174. Sun L, Wen Y, Zhang MH, Liu XX, Huan XP, Yang HT, et al. [Investigation and analysis of the related knowledge and behavior on AIDS of the FSWs in Jiangsu province]. Acta Universitatis Medicinalis Nanjing (Natural Science). 2012;32(1):10-5.

175. Yang Y, Li HL, Yang YH. [Surveillance of HIV/AIDS among FSWs in Lahuzu county, Yuinan]. Soft Science of Health. 2012;26(10):911-2.

176. Nie ZQ, Lin P, Li Y, Wang Y. [Surveillance of AIDS high-risk people in Guangdong province,2009]. Journal of Tropical Medicine. 2011;11(1):29-31, 45.

177. Zhang H, Li HL, Wang C, Yang YH. [Investigation on HIV/AIDS know ledge and related behavior of 1301 female commercial sex workers]. Soft Science of Health. 2012;26(1):47-9.

178. Zhang XD, Temmerman M, Li Y, Luo W, Luchters S. Vulnerabilities, health needs and predictors of high-risk sexual behaviour among female adolescent sex workers in Kunming, China. Sex Transm Infect. 2012.
